# Comparative genomics of transport proteins in seven *Bacteroides* species

**DOI:** 10.1371/journal.pone.0208151

**Published:** 2018-12-05

**Authors:** Hassan Zafar, Milton H. Saier

**Affiliations:** 1 Department of Molecular Biology, Division of Biological Sciences, University of California at San Diego, La Jolla, CA, United States of America; 2 Institute of Microbiology, University of Agriculture, Faisalabad, Punjab, Pakistan; Oswaldo Cruz Foundation, BRAZIL

## Abstract

The communities of beneficial bacteria that live in our intestines, the gut microbiome, are important for the development and function of the immune system. *Bacteroides* species make up a significant fraction of the human gut microbiome, and can be probiotic and pathogenic, depending upon various genetic and environmental factors. These can cause disease conditions such as intra-abdominal sepsis, appendicitis, bacteremia, endocarditis, pericarditis, skin infections, brain abscesses and meningitis. In this study, we identify the transport systems and predict their substrates within seven *Bacteroides* species, all shown to be probiotic; however, four of them (*B*. *thetaiotaomicron*, *B*. *vulgatus*, *B*. *ovatus*, *B*. *fragilis*) can be pathogenic (probiotic and pathogenic; PAP), while *B*. *cellulosilyticus*, *B*. *salanitronis* and *B*. *dorei* are believed to play only probiotic roles (only probiotic; OP). The transport system characteristics of the four PAP and three OP strains were identified and tabulated, and results were compared among the seven strains, and with *E*. *coli* and *Salmonella* strains. The *Bacteroides* strains studied contain similarities and differences in the numbers and types of transport proteins tabulated, but both OP and PAP strains contain similar outer membrane carbohydrate receptors, pore-forming toxins and protein secretion systems, the similarities were noteworthy, but these *Bacteroides* strains showed striking differences with probiotic and pathogenic enteric bacteria, particularly with respect to their high affinity outer membrane receptors and auxiliary proteins involved in complex carbohydrate utilization. The results reveal striking similarities between the PAP and OP species of *Bacteroides*, and suggest that OP species may possess currently unrecognized pathogenic potential.

## Introduction

One of the major bacterial lineages that flourished during the evolution of prokaryotic life is the phylum *Bacteroidetes*. *Bacteroides* species within this phylum are anaerobic, bile resistant, non-spore forming Gram-negative rods [1]. They are numerically the most abundant Gram-negative species to colonize the lower gastrointestinal tract (GIT) of adult humans, with members of the phylum *Firmicutes* being the most abundant Gram-positive bacteria [2]. The probiotic potential of species of *Bacteroides* is well documented, and this mutualistic association is maintained as long as these species remain in a designated bio-geographical location of the gut. The mucosal barrier plays a pivotal role in keeping these organisms in the gut and prevents contact with host tissues. However, in certain situations, such as intestinal surgery, rupture of the appendix, Crohn’s disease, diverticulitis and ulcerative colitis, this mucosal barrier may be compromised [3, 4]. This leads to various pathological conditions such as bacteremia, intra-abdominal abscesses (IAA) and formation of abscesses in various body sites such as the lungs, liver and brain [1]. Recent studies have shown that *Bacteroides* are among the most commonly isolated anaerobes from IAA, with *B*. *fragilis* being the most common isolate, supporting the notion that it is the most pathogenic specie of *Bacteroides* [5].

Studies of *Bacteroides* have revealed various biochemical adaptations allowing them to survive the dynamic environment of the human gut [1]. These species encode a cytochrome bd oxidase that may reduce the oxygen levels in the gut, thus helping themselves and other anaerobes to gain a growth advantage over other organisms [2, 3]. This ability to tolerate oxygen may assist them in not only thriving in the gut but also spreading from one host to another [6]. *Bacteroides* also play pivotal roles in carbohydrate fermentation in the gut, which results in the production of short chain fatty acids (SCFA). These SCFA are reabsorbed in the large intestine, and the host utilizes them as energy sources [7]. *Bacteroides* possess a vast repertoire of enzymes involved in the breakdown of carbohydrates; these enzymes include glycosylhydrolases, glycosidases, polysaccharide lyases and carbohydrate esterases that allow them to breakdown or assist other intestinal bacteria in the breakdown of both host-derived and plant glycans [8]. An interesting facet of the physiology of *Bacteroides* is the absence of transport-linked phosphorylation systems of the phosphotransferase system (PTS) [9]. However, these species have acquired alternative mechanisms to transport sugars into the cells and phosphorylate them [10].

The transporter classification database (TCDB) (www.tcdb.org) is the only internationally recognized database of transport proteins from a diverse range of organisms, and it has been adopted by the International Union of Biochemistry and Molecular Biology (IUBMB). The transporter classification (TC) system includes most transport proteins that have been characterized either structurally or functionally. The research reported in the present communication is a continuation of a series of studies on the transport proteins in gut commensals and pathogens. Previously, Tang and Saier, [11] and Do et al. [12] examined probiotic and pathogenic strains of *E*. *coli* and pathogenic strains of *Salmonella*, finding differences in the levels of pore-forming toxins (PFTs), protein secretion systems, iron acquisition systems and carbohydrate transporters.

The goal of the present study was to analyze the transport proteins in seven *Bacteroides* strains. Three of the strains studied are believed to be only probiotic (OP), while four of them have the dual ability of being both probiotic and pathogenic (PAP). These seven strains were examined for analysis of the distributions of single and multicomponent transport systems and their substrates. Table 1 highlights the basic traits of these seven strains.

**Table 1:** Overview of the seven *Bacteroides* species included in this study. ^a^

Strain	Abbreviation	Accession #	Genome size (Mbp)	Total # proteins identified	Total # transport proteins	Transport proteins (% of total)	Possible disease conditions/relationships
*Bacteroides dorei* CLT03T12C01	BD	NZ_CP011531.1	5.31	4063	458	11.2	None
*Bacteroides cellulosilyticus* WH2	BC	NZ_CP012801.1	7.08	5206	656	12.6	None
*Bacteroides salanitronis* DSM 18170	BS	NC_015164.1	4.24	3514	312	8.8	None
*Bacteroides thetaiotaomicron* 7330	BT	NZ_CP012937.1	6.49	4857	690	14.2	Intra-abdominal sepsis, colitis, endocarditis, peritonitis
*Bacteroides fragilis* YCH 46	BF	NC_006347.1	5.28	4578	515	11.2	Bacteremia, intra-abdominal sepsis, colitis, septic arthritis, meningitis, endocarditis, pericarditis
*Bacteroides vulgatus* ATCC 8482	BV	NC_009614.1	5.16	2995	422	14.1	Increased incidences of Crohn’s disease and Celiac disease (Sprue/Ceoliac)
*Bacteroides ovatus* ATCC 8483	BO	NZ_CP012938.1	6.47	4749	508	10.6	Increased incidences of Crohn’s disease and diabetes

^a^All seven strains are found extracellularly, usually in the colon. The first three strains are only probiotic (OP) and the last four are probiotic and pathogenic (PAP).

## Materials and methods

### 2.1 Genome-BLAST (G-BLAST) searches of *Bacteroides* proteomes

The FASTA formatted protein-coding sequences of *B*. *thetaiotaomicron strain 7330* [13], *B*. *ovatus* ATCC 8483 [13], *B*. *cellulosilyticus* strain WH2 [14], *B*. *dorei* strain CL03T12C01 [14], *B*. *fragilis* strain YCH 46 [14], *B*. *salanitronis* strain DSM 18170 [15] and *B*. *vulgatus* ATCC 8482 [16] were selected and obtained from GenBank. The selection was done on the basis of the draft qualities and completenesses of their sequenced genomes, as well the pathogenic potential of these strains on humans. The proteomes of these seven *Bacteroides* species were screened for homologs of all proteins contained in the Transporter Classification Database (www.tcdb.org) in January 2017 using the program G-BLAST [17]. This program is designed to retrieve information for both the genome query and TC top hit sequences, TC numbers, numbers of amino acyl residues (aas), numbers of predicted TMSs using the HMMTop 2.0 program, both query and hit protein e-value, regions with sequence similarity, and regions of TMS overlap between the query and the hit proteins. For prediction of the number of TMSs, G-BLAST uses the Web-based Hydropathy, Amphipathicity, and Topology (WHAT) program, which aligns the plots of hydrophobicity and amphipathicity throughout the length of the protein [18, 19]. The WHAT program displays amphipathicity and hydropathy profiles of the proteins with a window size of 19 amino acyl residues (aas) and a viewing angle of 100° for α-helices or, a window size of 9 aas and 180° for β strands. Proteins lacking TMSs were not omitted, since multicomponent systems may possess soluble components that can be potential transport protein constituents.

### 2.2 Examination of distant transport protein homologues of *Bacteroides*

For G-BLAST searches, we initially used an arbitrary e-value cut off of 0.0001. Manual examination of the remaining proteins (having e-values of >0.0001) was done using topological data to determine if the proteins were either true homologues or false positives. As two proteins displaying homology in hydrophilic regions can give small e-values, it was necessary to manually examine the regions of overlap to prevent the selection of proteins that had good scores, but were not actually homologous in their transmembrane domains. The hydropathy profiles generated by the WHAT program was used to determine whether the program had missed a TMS or predicted a TMS in an incorrect region. By using the AveHAS program, confirmation of predictions with several homologues was accomplished [19]. Proteins that had moderate e-values, between 0.0001 and e^-8^, indicated a range in which the presence of distant protein homologs was possible, and hence, they were examined more closely using the aforementioned steps.

### 2.3 Identification of substrates transported

Authentic transport protein homologues were assigned substrates according to TCDB hit entries. For entries of unknown function, the genome context of the encoding genes was considered, especially if the encoding genes were within multicistronic operons. Information obtained from the scientific literature was also used to deduce their functions.

### 2.4 Occurrence of multicomponent systems

Our analysis identified various multicomponent transport systems in the seven *Bacteroides* genomes. This identification was primarily based on the presence of the transmembrane (TM) protein of the systems; however, in some instances other constituents were also found. Moreover, if the TM protein was identified, the transport system was considered to be present.

## Results

### 3.1 Overview of transporter types

The Transporter Classification (TC) system includes a plethora of transport proteins, many of which are characterized both structurally and functionally. In TCDB, transporters are organized into five well-defined classes (1–5), and two less well-defined classes (8–9). The five well-defined classes are (1) channels (2) secondary carriers (3) primary active transporters, (4) group translocators and (5) transmembrane electron flow carriers. The latter two classes include (8) auxiliary transport proteins and (9) transporters or putative transporters of unknown function or mechanism of transport.

The seven *Bacteroides* strains examined were analyzed for the occurrence of transporters using G-BLAST [17] and TCDB as noted in the Methods section. The complete results are described in the Supporting information section (S1 Table), whereas Table 2 gives an overview of the subclass distributions of the transporters from each organism. Examination of the transport proteins encoded within the seven genomes revealed that the largest number of transporters are present in the probiotic and pathogenic (PAP) *B*. *thetaiotaomicron* (BT) strain 7330 (690), and the smallest number of transport proteins were found in the only probiotic (OP) strain, *B*. *salanitronis* (BS) DSM 181370 (312), while the remainder of the probiotic and pathogenic (PAP) strains *B*. *ovatus* (BO) ATCC8483, *B*. *fragilis* (BF) YCH 46 and *B*. *vulgatus* (BV) ATCC 8482 had 508, 515 and 422 transport proteins, respectively. The remaining two OP strains, *B*. *cellulosilyticus* (BC) strain (WH2) and *B*. *dorei* (BD) strain CL03T12C01 have 656 and 508 transport proteins, respectively.

**Table 2:** Overview of the *Bacteroides* transport protein numbers based on TC subclass.

TC subclass and description	Number of transport proteins/subclass							Percent (%) of transport proteins						
	BD	BC	BS	BT	BF	BV	BO	BD	BC	BS	BT	BF	BV	BO
1.A, α-type channels	18	19	17	34	19	17	20	3.9	2.9	5.4	4.9	3.7	4.0	3.9
1.B, β-barrel porins	55	69	33	108	61	47	74	12	10.5	10.6	15.7	11.8	11.1	14.6
1.C, Pore-forming toxins	4	5	1	3	4	3	2	0.9	0.8	0.3	0.4	0.8	0.7	0.4
1.E, Holins	3	3	2	5	4	3	4	0.7	0.5	0.6	0.7	0.8	0.7	0.8
2.A, Porters (uniporters, symporters, antiporters)	107	149	90	190	120	102	112	23.3	22.7	29	27.5	23.3	24.2	22
3.A, P-P-bond-hydrolysis-driven transporters	122	157	77	138	133	111	103	26.6	23.9	24.7	20	25.8	26.3	20.3
3.B, Decarboxylation-driven transporters	8	10	5	10	10	8	8	1.7	1.5	1.6	1.5	1.9	1.9	1.6
3.D, Oxidoreduction-driven transporters	22	37	21	39	28	20	25	4.8	5.6	6.7	5.7	5.4	4.7	4.9
4.A, Phosphotransfer-driven group translocator homologs	1	0	1	1	0	1	1	0.2	0	0.3	0.1	0	0.2	0.2
4.B, Nicotinamide ribonucleoside uptake transporters	0	1	0	1	0	0	1	0	0.2	0	0.1	0	0	0.2
4.C, Acyl-CoA ligase-coupled transporters	0	1	0	0	6	0	0	0	0.2	0	0	1.2	0	0
4.D, Polysaccharide synthase exporters	5	26	2	11	6	2	1	1.1	4.0	0.6	1.6	1.2	0.5	0.2
5.A, Transmembrane two-electron transfer carriers	0	1	0	1	0	0	0	0	0.2	0	0.1	0	0	0
8.A, Auxiliary transport proteins	80	102	38	100	66	77	117	17.5	15.5	12.2	14.5	12.8	18.3	23
9.A, Recognized transporters of unknown biochemical mechanism	3	7	1	3	6	4	2	0.7	1.1	0.3	0.4	1.2	0.9	0.4
9.B, Putative transport proteins	30	68	24	46	52	27	38	6.6	10.4	7.7	6.7	10.1	6.5	7.5
Total	458	656	312	690	515	422	508	100	100	100	100	100	100	100

TC subclass 1.A includes α-type channels. These channels catalyze the movement of solutes through transmembrane pores or channels in an energy-independent process. Both OP and PAP strains of *Bacteroides* exhibit a similar distribution of TC subclass 1.A channels, with each of them having 17–34 such channel proteins. The PAP BT strain has the most such channels (34).

TC subclass 1.B includes β-barrel porins. These proteins form transmembrane pores usually permitting the energy-independent passage of solutes across the outer membranes of Gram-negative bacteria [20, 21]. Among the strains, BT has the largest number of such proteins (108). In the case of the remaining PAP strains, BO has 74, while BF and BV contain 61 and 47 such porins, respectively. Among the OP strains, BC has the most (69) such porins followed by BD, with 55. The smallest number of subclass 1.B proteins was found in the OP strain, BS (33).

TC subclass 1.C includes pore-forming toxins (PFTs). PFTs are the most common type of bacterial cytotoxic proteins. Surprisingly, the OP strain, BC, contains 5 of these, while the remaining PAP and OP strains have a range of 1–4 such proteins. There was no apparent correlation between numbers of these toxins and the known pathogenic potential of the different species.

TC subclass 1.E consists of holins. Holins perform a variety of functions in prokaryotes such as biofilm formation, cell lysis, virulence and toxin release, and they may also function as antimicrobials [22]. Within this subclass, the seven strains show similar patterns. The PAP BT strain encodes 5 holins, while the rest have between 2–4.

Secondary carriers (TC 2.A) represent the largest group of transport systems in all nine strains with 22–29% of the transport proteins in each strain falling within this class; these are usually single component systems. The largest number of secondary carriers is found in the PAP BT strain, which contains 190 such carriers, while the remaining strains have 90–149 of these proteins. This reflects the greater metabolic flexibility of BT as compared to the other strains.

TC subclass 3.A represents pyrophosphate hydrolysis driven primary active transporters, usually multi-component systems. The OP strain, BC, has 157 of these types of transport proteins, while the remaining strains have between 77–138. TC subclass 3.B consists of decarboxylation driven transporters, which are involved in the extrusion of Na^+^ from the cytoplasm of the cell; these systems are also multi-component [23]. All the seven strains possess 5–10 such proteins, but none appears to have a complete system. These Na^+^ pumps, when present, may play an important role in ionic homeostasis [24]. TC subclass 3.D includes oxidoreduction driven transporters. BT has the most, 39, followed by BC, which has 37 such constituents. The remaining strains have 20–28.

TC subclass 4.A consists of phosphotransfer-driven sugar transporting group translocators. Both BC and BF lack any such proteins, while the rest each has only one such constituent (TC# 4.A.5.1.4), which, however, cannot function by a PTS- dependent mechanism. TC subclass 4.B contains the nicotinamide ribonucleoside group translocating uptake permease (PnuC) family, which includes several (putative) vitamin transporters. Only BT, BO, and BC encode one such protein (TC# 4.B.1.1.4), and these proteins are likely to be thiamin uptake porters. TC 4.C includes Acyl-CoA-ligase coupled transporters. These proteins activate fatty acids for lipid biosynthesis and may function by group translocation [25]. The only proteins of this class believed to be transporters possess 2–4 TMSs [26]. G-BLAST results revealed more transport proteins of TC 4.C, but only those transporters having at least two TMSs were selected, with BC and BF both having 1 and 6 potential transporters, respectively.

TC subclass 4.D includes integral membrane polysaccharide synthase/exporters (glycosyltransferases); these putative transporters are present in all strains. The most probable group translocating glycosyltransferases are encoded by BC (26). The remaining six strains have 1–11 of these proteins. These glycosyltransferases function in the biosynthesis of polysaccharides and complex oligosaccharides and may play roles in various biological functions such as cell signaling, cellular interactions and pathogenesis [27].

TC subclass 5.A includes transmembrane two-electron carriers. These carriers transfer electron pairs between a donor and acceptor on the two sides of the membrane, thus having an effect on cellular energetics. Of the seven strains, only BT and BC encode such transporters (one each).

TC subclass 8.A includes auxiliary transport proteins that do not participate directly in the transport process but facilitate this process. BO has 117 such transport proteins followed by BC, which has 102, while the rest have 38–100.

Transporters of subclass 9.A are not mechanistically defined, and therefore, cannot be assigned to one of the defined classes in TCDB. Each of the strains contains 1–7 proteins from TC subclass 9.A. TC subclass 9.B contains putative uncharacterized transport proteins. The seven strains contain 24–68 such proteins each.

### 3.2 α-type channel proteins (TC subclass 1.A)

A significant number of α-type channel proteins are present in all seven *Bacteroides* species. A majority of these proteins may play roles in ionic and water homeostasis, while others are associated with ionic stress responses. Transporters of the voltage-gated ion channel (VIC) superfamily (TC#1.A.1) are present in six of the strains (all but the OP BS strain), with a single 6 TMS K^+^ channel (TC#1.A.1.24.3), common to the six strains. The high scores, all matching the same TC entry, are clearly indicative of the orthologous nature of these six proteins.

Three strains, BT, BO and BS, possess representatives of the MIP family (TC#1.A.8) of aquaporins and glycerol facilitators, while the rest of the strains have none. Three of these four proteins have high scores each hitting the same TCDB entry (TC#1.A.8.3.1), indicating that they are orthologous. These proteins are probably concerned with the transport of water, glycerol and dihydroxyacetone [28].

Six of the strains have a single ammonium channel of the Ammonium Transporter Channel (Amt) family (TC#1.A.11), although BF has none. The OP BS strain and the PAP BT and BV strains each contain one protein with hits of the homolog from *Synechococcus* sp (TC#1.A.11.2.3), suggesting that they are orthologous. The *Synechococcus* transporter is a high-affinity ammonia/methyl ammonia transporter [29]. The two strains BS and BO each contain a protein that hits the homolog of *Azospirillum brasilense* (TC#1.A.11.1.4) with a good score.

Three of the PAP strains BT, BO and BF contain one homologue each of an epithelial chloride channel (TC#1.A.13.2.3). It has been suggested that this uncharacterized TC entry is an amyloid protein due to the presence of asparagine and glutamine-rich regions [30]. Although, bacterial homologues have been observed, the functions of these proteins are unknown. These proteins exhibit topologies similar to those of their mammalian counterparts, perhaps indicative of analogous functions. Thus, these proteins may prove to exhibit chloride channel activities.

All of the seven strains have one homologue each of the large mechanosensitive ion channel (MscL) (TC#1.A.22.1.1). These channels catalyze the efflux of small proteins, cations, and osmolytes upon hypoosmotic shock [31]; the seven strains also have paralogs of the small conductance mechanosensitive ion channel family (MscS; 1.A.23), and one of the paralogs in each organism is most similar to (TC#1.A.23.2.1). Like the MscL channels, this MscS channel protein responds to hypoosmotic shock, contributing to osmotic stability. Proteins of both families (TC#1.A.22; TC#1.A.23) have important functions in osmotic adaptation [32, 33]. All seven strains have a single member of the Mg^2+^ Transporter-E (MgtE) Family (TC#1.A.26) of magnesium uptake channels. The seven proteins hit the same TC entry (TC#1.A.26.1.2), clearly indicating that they are orthologous. Two of the OP strains, BD and BC, have two urea transporter channel proteins, which are close homologs of the urea transporter (TC#1.A.28.1.4) of *Desulfovibrio vulgaris*.

All of the strains have multiple paralogs of the H^+^/Na^+^-translocating MotAB/ExbBD/TolQR channel-forming proteins (TC#1.A.30). The MotAB proteins energize flagellar motility, while the ExbBD proteins energize the uptake of large molecules across the membrane, molecules including vitamin B_12_, siderophores and colicins, but also phage DNA [34]. The TolQR channels stabilize the outer membrane and also assist in the uptake of certain colicins. Out of all the strains, only the OP strain, BD has MotAB energizers. BD has components of the systems (TC#1.A.30.1.2) and (TC#1.A.30.1.6), which are sodium motive force (SMF) dependent energizers. The occurrence of ExbBD/TolQR energizers is variable in the seven *Bacteroides* strains, with all seven strains having homologues of a TolQR energizer (TC# 1.A.30.2.9). Out of all the seven strains, only BC has homologues of (TC#1.A.30.3.2), resembling the *Myxococcus xanthus* adventurous gliding motility proteins (AglR and AglS, homologues of TolQ and TolR respectively). Four strains, BT, BF, BD and BO, have a homologue of (TC#1.A.30.2.7), another system of *Myxococcus xanthus* which functions in gliding motility. Hence, several of the *Bacteroides* strains have potential energizers resembling those used for gliding and flagellar motility in other bacteria.

All strains show paralogs of the potential cation channel-forming heat shock protein-70 (HSP-70) family, (TC#1.A.33). Heat Shock Proteins have been identified from various organisms, and in eukaryotes they can insert into membranes, forming channels [35]. However, this has never been shown for a prokaryote. Six of the strains have a homolog of (TC#1.A.33.1.5), an actin-like ATPase involved in cell morphogenesis. All of the strains have homologues of the chaperone protein DnaK (TC#1.A.33.1.2), involved in chromosomal DNA replication, but that also participates in hyperosmotic shock adaptation [36].

The remaining channel protein families are present only in select *Bacteroides* strains. Only the BT strain has homologues of the *Bacillus* gap junction-like-channel-forming complex (GJ-CC) (TC#1.A.30.5). Members of the CorA Metal Ion Transporter Family (MIT) (TC#1.A.35) are present in BS, BO and BT. All of the strains have at least one member of the Camphor Resistance or Fluoride Exporter Family (TC#1.A.35). These fluoride ion (F^-^) export channels protect the bacterium from the toxic effects of F^-^ by lowering its cytoplasmic levels [37]. All of the strains contain a 7-TMS homologue (TC#1.A.62.2.1) of the Homotrimeric Cation Channel Family (TC#1.A.62). These proteins are related to a protein found in another Bacteroidetes species, *Gramella forsetti*. In prokaryotes, members of this family play roles in the efflux of metabolites (amino acids/nucleotides) [38].

### 3.3 β- type porins (TC subclass 1.B)

β-type porins constitute a major fraction of the transporters encoded by *Bacteroides* species. The outer membranes of *Bacteroides* contain numerous receptors and porins for the utilization, degradation, and transport of oligosaccharides and polysaccharides [39]. The polysaccharide end products are further used by other resident microbiota including pathogens [40]. Each *Bacteroides* strain has outer membrane porins from at least seven different families. Four strains, BD, BO, BC and BV, each contains a member of the *Pseudomonas* OprP Porin (POP) Family (TC#1.B.5), concerned with the transport of anions. All of the strains contain homologues of the OmpA-OmpF Porin (OOP) Family; six of the strains (all except BS) have a member of the, (FadL) Family, (TC# 1.B.9), concerned with the transport of hydrocarbons and fatty acids [41]. Most strikingly, all seven strains contain numerous homologues of a SusC like receptor/porin for oligosaccharides, (TC#1.B.14.6.1), with the OP strain, BO, having the most (8). The SusC receptor/porin in TCDB is involved in starch utilization and binds maltooligosaccharides. It functions in complex with another protein (TC#8.A.46.1.1), a SusD oligosaccharide binding protein. A class of metabolic corrinoid cofactors (one of which is vitamin B_12_) is a central component of the fitness landscape in the gut. These cofactors are required for methionine synthesis and other metabolic pathways [42]. *Bacteroides* lack the machinery to synthesize these large and complex cofactors, and instead rely on corrinoid transporters to extract them from the medium. Four of the strains, BD, BO, BF and BT have one homologue each of the vitamin B_12_ transporter (TC#1.B.14.3.1). With the exception of BS, the remaining six strains also encode the TonB-dependent transmembrane thiamine receptor (TC#1.B.14.14.1). However, striking differences occur in some of the other families; for example, each organism exhibits different sets of outer membrane receptors (OMR) (TC#1.B.14). This fact can be explained by the different specificities of these receptors as illustrated in the Supporting information (S1 Table). Six of the strains contain homologues of the Outer Membrane Factor (OMF) Family (TC#1.B.17), but BS and BV lack homologues of (TC#1.B.17.2.6), present in the remaining five strains. This protein is a putative OM macrolide efflux protein. The complement of OMR’s and OMF’s of *Bacteroides* must suit the landscape of the gut where these microbes reside.

All seven *Bacteroides* strains have members of the Outer Membrane Protein Insertion Porin (Bam complex) (OmpIP) Family (TC#1.B.33). Homologues of the Omp85 translocase (TC#1.B.33.1.1) of *Neisseria meningitidis* are present in all seven strains. This protein is part of the outer membrane protein assembly complex, which is involved in assembly and insertion of β-barrel proteins into the outer membrane. BO, BS and BT strains contain components of, (TC#1.B.42.1.2), a lipopolysaccharide export complex LptBCFG-A-DE. This seven-component system translocates LPS from the outer surface of the inner membrane to the outer surface of the OM using ATP hydrolysis [43]. Remaining families of this subclass represented in the *Bacteroides* strains include members of the Probable Protein Translocating *Porphyromonas gingivalis* Porin (PorT) Family (TC#1.B.44), the Curli Fiber Subunit CsgA, Porin (CsgG) family (TC#1.B.48), the Intimin/Invasion Autotransporter-3 (AT-3) Family (TC#1.B.54), the *Legionella* Major-Outer Membrane Protein (LM-OMP) Family (TC#1.B.57), the Outer Membrane channel (OMC) Family (TC#1.B.70) and the proteobacterial/verrucomicrobial porin (PVP) Family (TC#1.B.71).

### 3.4 Pore-forming toxins (TC subclass 1.C)

Pore-forming toxins (PFTs) are the most common bacterial cytotoxic proteins and are required for virulence in a large number of important pathogens, including both Gram positive and Gram-negative bacteria [44]. PFTs generally disrupt host cell membranes, but they can have additional effects independently of pore formation. Substantial effort has been devoted to understanding the molecular mechanisms underlying the functions of certain model PFTs. There are disparate patterns for the PFT’s of the strains examined here (details given in Table 3). Some of them are members of the membrane attack complex/perforin (MACPF) family (TC#1.C.39) [45]. None of the toxins were OP specific, while one of the toxins was PAP specific. BV contains a homologue of the bacteriocin cerein 7b (TC#1.C.102.1.1), which is a member of Ennahar’s class IId bacteriocins that are exported via a Sec-independent pathway [46]. Of the OP strains, only BD encodes an Hly-III homologue, indicative of hidden pathogenic potential. With the exception of BO, the other three PAP strains have one homologue each of hemolysin, (Hly-III).

**Table 3:** Occurrence of pore-forming toxins in the seven *Bacteroides* strains. Toxins marked in yellow are present in PAP strains; those marked in pink are present in both PAP and OP strains. No probiotic specific toxins were identified.

TCID	Family	Function	BD	BS	BC	BT	BF	BV	BO
1. C.39.13.1	Membrane attack complex/perforin (MACPF)	Pore formation	0	0	1	1	0	1	1
1.C.39.13.2	Membrane attack complex/perforin (MACPF)	Pore formation	2	0	0	0	1	0	0
1.C.39.13.3	Membrane attack complex/perforin (MACPF)	Pore formation	0	0	1	0	1	0	0
1.C.82.1.1	(HP2-20)	Pore formation	1	1	0	1	0	1	1
1.C.102.1.2	Cerein	Pore formation	0	0	0	0	0	1	0
1.C.113.1.1	Hly III	Pore formation	1	0	0	1	1	1	0

### 3.5 Holins (TC subclass 1.E)

Holins are encoded within the genomes of Gram-negative and Gram-positive bacteria as well as phages. Their primary function appears to be secretion of murein hydrolases across the cytoplasmic membrane to the cell wall, where these enzymes hydrolyze the peptidoglycan as a prelude to cell lysis [47]. As these muralytic enzymes are chromosomally encoded within the cell that is also the target, they are referred to as “autolysins” [48]. Both CidAB and LrgAB affect biofilm formation, oxidative stress, stationary phase survival, and antibiotic tolerance in a reciprocal fashion in other bacteria such as *Staphylococcus aureus*, where their genes are regulated by a 2-component regulatory system, LytSR [49]. All seven strains contain a homologue of the murein hydrolase transporter LrgA (TC#1.E.14.1.13). Other holins identified in the seven strains include members of the Putative Actinobacterial Holin-X (Hol-X) Family (TC#1.E.34), and the Putative 3–4 TMS Transglycosylase-associated Holin (T-A) Family (TC#1.E.43); however, the functions of these holins are unknown.

### 3.6 Secondary carriers (TC subclass 2.A)

The largest family of secondary carriers found in nature, the Major Facilitator Superfamily (MFS; TC#2.A.1.), is also the best-represented secondary carriers superfamily in all seven strains. These strains contain probable drug exporters of the Drug:H^+^ Antiporter-1 DHA1 (TC#2.A.1.2) and the Drug:H^+^ Antiporter-2 DHA2 (TC#2.A.1.3) families. However, only BF, BD, and BC have a transporter of the DHA-3 Family (TC#2.A.1.21.22), which includes macrolide efflux pumps that in one case can be induced by erythromycin and the antimicrobial peptide LL-37 [50]. BT has a homologue of (TC#2.A.1.4), a transporter that is concerned with sugar-phosphate uptake. In addition, all of the strains have members of the Fucose:H^+^ Symporter (FHS) Family (TC#2.A.1.7), the Nucleoside:H^+^ Symporter (NHS) Family (TC# 2.A.1.10) and the Anion-Cation Symporter (ACS) Family (TC# 2.A.1.14). Only one OP strain, BC, and three PAP strains, BF, BT and BO, have proteins of both the Fosmidomycin Resistance (Fsr) Family (TC#2.A.1.35) and the Acriflavin-Sensitivity (YnfM) Family (TC#2.A.1.36), while BS, BV and BD have only one member of one or the other family. The protein (TC#2.A.1.35.1) is a fosmidiomycin resistance protein, and homologues are present in BS, BC, BF, BT and BO. This *E*. *coli* protein confers resistance against trimethoprim and carbonyl cyanide m-chlorophenyl hydrazine, as well as fosmidiomycin, so it is a multidrug exporter [51]. All of the strains have one protein of the Uncharacterized Major Facilitator-5 (UMF-5) Family, the members of which are likely to be multi-drug resistance pumps based on sequence similarity analysis.

With the exception of BT, all strains have members of the Glycoside-Pentoside-Hexuronide (GPH):Cation Symporter Family (TC# 2.A.2). These porters catalyze uptake of sugars (mostly, but not exclusively, glycosides) together with a monovalent cation (H^+^ or Na^+^). The export of heavy metals is mediated by the Cation Diffusion Facilitator (CDF) family (TC#2.A.4), and proteins of this family are found in all strains except BF.

The superfamily of secondary carriers, that is second most prevalent after the MFS in the *Bacteroides* strains, is the Resistance-Nodulation Cell Division (RND) Superfamily (TC#2.A.6). RND pumps export small molecules (heavy metals and antibacterial compounds) and some organic molecules such as lipids out of the cell or from the periplasm of Gram-negative bacteria into the extracellular medium, thus contributing to antibiotic and heavy metal resistance [52]. The PAP BT strain of BT has the most members with 45, and BS has the smallest number of such transporters with 11 members. Most of these transporters, found in both PAP and OP strains, are members of the Largely Gram-Negative Hydrophobe/Amphiphile Efflux (HAE-1) Family (TC#2.A.6.2) and are responsible for drug efflux [53]. All of the strains except BO contain homologues of a three-component system (TC#2.A.6.2.22), for bile-inducible drug efflux. This system in *Campylobacter jejuni* contributes to biofilm formation on mucosal surfaces, thus assisting the bacteria to thrive in the harsh environment of the gut [54]. Members of the Heavy Metal Efflux (HME) family (TC#2.A.6.1) are present in all strains. Five strains have a homologue of the protein translocase subunits SecDF, (TC#2.A.6.4.3), which functions as a RND pump to facilitate proton-driven protein secretion via the General Secretory Pathway (Sec; TC#3.A.5). Only the PAP BT possesses a member of the poorly characterized putative Hydrophobe/Amphiphile Efflux-3 (HAE-3) family (TC#2.A.6.7).

The Drug Metabolite Transporter (DMT) Superfamily (TC#2.A.7) is the third largest superfamily of secondary carriers in the *Bacteroides* strains, with BT having 14, BC, 10, BD, 9 and the rest having eight members. Members of this family are involved in drug export and small metabolite import [55]. Most of the top hits in TCDB have not been characterized, so only limited substrate assignments for these hits were possible.

All of the strains have proteins of the Monovalent Cation:Proton Antiporter-1 (CPA1) Family (TC#2.A.36) and the Monovalent Cation:Proton Antiporter-2 (CPA-2) Family (TC#2.A.37), both of the CPA Superfamily (TC#2.A.37). All seven strains seem to possess a functional Tat protein secretory system pathway having TatA and TatC homologues of the TatABCE translocase (TC#2.A.64) of *E*. *coli*. As bacteria require one TatC and one of the three proteins; TatA, TatB or TatE for transport, the systems identified in the seven strains seem functional. The Tat system exports across the cytoplasmic membrane large folded proteins containing characteristic twin-arginine motifs in their signal peptides [56].

Two different constituent families of the Multidrug/Oligosaccharidyl-lipid/Polysaccharide (MOP superfamily (TC#2.A.66) are present in all of the seven strains. These are included in the Multi Antimicrobial Extrusion (MATE) family (TC#2.A.66.1), and the Polysaccharide Transport (PST) Family (TC#2.A.66.2). The MATE family includes a functionally characterized multidrug efflux system from *Vibrio parahaemolyticus*, NorM, and several homologues from other closely related bacteria that function by a drug:Na^+^ antiport mechanism. However, only BV contains a transporter of the LPS Precursor Flippase (LPS-F) Family, and only BT has a member of the Uncharacterized MOP-12 (U-MOP12) Family (TC#2.A.66.12).

### 3.7 Primary pyrophosphate hydrolysis-driven active transporters (TC subclass 3.A)

In all of the strains, the ABC Superfamily (TC#3.A.1) is the best represented. This superfamily is represented in all domains of life and is known to transport a wide variety of large and small substrates for both uptake and export [57]. The strains contain variable numbers of proteins within this superfamily, with BD having 70, BS 30, BC 91, BT 76, BF 77, BO 57 and BV 55. Overall, the seven strains have more ABC efflux systems than uptake systems. Substrates of the ABC efflux systems include 1) drugs, 2) proteins and peptides, 3) lipids and lipoproteins and 4) secondary metabolites. Proteins of the Carbohydrate Uptake Transporter (CUT-2) Family (TC#3.A.1.2) are present in six of the strains with PAP BV being the only exception. The substrates of these multicomponent transporters include allose, arabinose and glucose. Protein systems of the Polar Amino Acid Uptake Transporter (PAAT) Family (TC#3.A.1.3) are found in all strains except BS. In the remaining six strains, a complete system of the Polyamine/Opine/Phosphate Uptake Transporter (POPT) Family (TC#3.A.1.11) for the transport of polyamines, especially spermidine and putrescine, is present in each species. It has been reported that polyamines play important roles in chromosomal stabilization, bacterial metabolism (including biosynthesis of siderophores) and stimulation of growth, but they can also act as scavengers of free radicals [58].

All strains possess orthologous sets of the integral membrane components of the ATP synthases in the F-ATPase Superfamily (TC#3.A.2). A pivotal characteristic of these systems is the reversibility of the enzyme for either the establishment of a proton motive force or ATP synthesis [59]. With the exception of BS, all strains contain a multicomponent high affinity K^+^ uptake system, KdpABC (TC#3.A.3.7.2), which is regulated by the direct interaction of the IIA^Ntr^ protein with the sensor kinase/response regulator, KdpDE in *E*. *coli* [60]. This system will not be included in the following descriptions of P-type ATPases. These ATPases are present in all of the strains analyzed, but they have different substrate specificities. There are two such transporters in BD: one is an efflux pump for Cu^2+^, Fe^3+,^ and Pb^2+^; the other is specific for mono and divalent heavy metals such as Cu^+^, Ag^+^, Zn^2+^ and Cd^2+^. *B*. *cellulosilyticus* has four P-ATPases; one is for the export of Ca^2+^, another for the uptake of Ni^2+^/Mg^2+^, a third for the uptake of Cu^+^, and a fourth for the efflux of Cd^2+^, Co^2+^ and Zn^2+^. BS has two transporters, one for the efflux of Ca^2+^, and the other for the uptake of Cu^2+^, respectively. Three ATPases in BF have different substrate specificities, with one involved in the uptake of Mg^2+^ and Ni^2+^, the second for Cu^2+^ uptake, the third for the uptake of Ag^+,^ Cu^+^ Zn^2+^ and Cd^2+^ (monovalent and divalent metals). BT, has two transporters, one specific for Cu2^+^ and Ag^+^ and the other for Zn^2+^. BV has one transporter for the uptake of Cu^2+^. The diversity in these transporters probably reflects the types of stress faced by these organisms. Thus, the functions of most of the P-type ATPases in prokaryotes involve protection from environmental stress conditions [61]. All strains have proteins homologous to components of the general secretory pathway (Sec) (TC#3.A.5). Detailed descriptions of the components identified are given in Table 4.

**Table 4:** Components of the General Secretory Pathway (Sec-SRP) complex identified in the seven *Bacteroides* strains (×, at least one homologue was identified; -, no homologue was identified).

Components	BD	BS	BC	BT	BF	BV	BO
SecY	×	×	×	×	×	×	×
SecE	×	×	×	×	×	×	×
SecG	×	×	×	×	×	×	×
Ffh	×	×	×	×	×	×	×
FtsY	×	×	×	×	×	×	×
SecA	×	-	×	-	×	-	×
ybaA	-	-	-	-	-	-	-
YajC	×	×	×	×	×	×	×
SecD/F	×	×	×	-	-	×	×

The seven strains seem to possess incomplete T4SS (TC#3.A.7), although all strains contain constituents of the complete systems. These systems in Gram-negative bacteria are often involved in bacterial conjugation and are pivotal to the transport of protein-DNA complexes and virulence factors [62]. In addition, all strains possess components of Bacterial Competence-Related DNA Transformation (DNA-T) systems (TC#3.A.11). These systems are important for the uptake of DNA under normal physiological conditions [63]. Details of secretion systems are given in Table 5. All OP strains and BV contain a single member of the H^+^, Na^+^-translocating pyrophosphatase (TC#3.A.10), and the hit protein (TC#3.A.10.1.10) is a putative K^+^-stimulated pyrophosphate-energized Na ^+^pump [64].

**Table 5:** Occurrence of secretion system components in *Bacteroide*s strains with numbers of transport proteins in both PAP and OP strains^a^.

Family	TCID	Function	BD	BS	BC	BT	BF	BV	BO
T1SS	3.A.1.105.4	Drug (pyoluteorin) exporter (5 components)	1	1	2	2	2	2	3
	3.A.1.105.15	Unknown exporter (4 components)	3	3	1	2	2	0	1
	3.A.1.105.16	Unknown exporter (3 components)	3	0	3	2	2	1	3
	3.A.1.121.4	Drug (fluoroquinolones) exporter (2 components)	2	1	2	3	2	2	3
	3.A.1.122.1	Drug (macrolides) exporter (2 components)	5	7	6	3	7	5	2
	3.A.1.122.2	Peptide (antimicrobials) exporter (4 components)	8	2	7	8	19	7	6
	3.A.1.122.3	Peptide (enterocin) exporter (3 components)	0	0	1	0	1	1	1
	3.A.1.122.12	Drug (arthrofactin) exporter (2 components)	1	1	2	4	2	1	1
	3.A.1.122.14	Unknown exporter (2 components)	0	1	2	1	1	1	1
	3.A.1.122.16	Drug (macrolides) exporter (2 components)	1	0	1	1	0	0	1
	3.A.1.125.1	Protein (lipoproteins) exporter (3 components)	2	1	2	2	2	2	2
	3.A.1.125.5	Unknown exporter (2 components)	1	0	0	0	0	1	0
	3.A.1.128.6	Unknown exporter (2 components)	2	1	0	1	1	1	1
	3.A.1.131.1	Drug exporter (bacitracin) (2 components)	1	0	1	1	1	1	1
	3.A.1.132.3	Unknown exporter (2 components)	2	1	1	1	1	0	1
	3.A.1.132.6	Unknown exporter (2 components)	1	0	2	1	0	1	1
T4SS	3.A.7.7.1	Conjugal DNA protein transfer system or ViRB (15 components)	4	5	6	4	4	4	3
	3.A.7.11.1	Type IV betaproteobacterial DNA secretion system (20 components)	1	5	2	5	5	6	4
	3.A.7.13.2	Conjugal DNA protein transfer system or VIRB (12 components)	1	0	0	0	1	0	0
	3.A.7.14.2	Type IV (conjugal DNA-protein transfer) (11 components)	1	4	2	5	2	3	5
	3.A.7.16.1	Conjugal DNA protein transfer system or VIRB (13 components)	3	2	1	3	1	3	1
	3.A.7.19.1	Conjugal DNA protein transfer system or VIRB (19 components)	4	6	9	6	5	6	5
T6SS	3.A.23.1.1	T6SS VasA-L (14 components)	8	7	9	6	5	6	5

^a^ No PAP or OP specific secretory system was found in the seven *Bacteroides* strains.

All strains have components of the Na^+^-Transporting Carboxylic Acid Decarboxylase (NaT-DC) Family (TC#3.B), which catalyze decarboxylation of a substrate carboxylic acid and use the resultant energy released to drive extrusion of one or two Na^+^ ions from the cytoplasm of the cell [23, 24]. However the strains do not possess the membrane protein of these systems, which in combination with the decarboxylase, is necessary for transport, in case of the *Bacteroides* strains these decarboxylases are not part of a transport system.

### 3.8 Oxidoreduction driven transporters (TC subclass 3.D)

*Bacteroides* as anaerobes can employ either anaerobic respiration or fermentation for the generation of energy. They can form rather primitive electron transport chains for their energy needs, for example with fumarate as the final electron acceptor [65]. Few constituents of the proton pumping electron transfer complexes present in many aerobic bacteria, such as proton-translocating NADH dehydrogenase (TC#3.D.1), and proton-translocating cytochrome oxidase (TC#3.D.4) are present in the seven strains. Interestingly, all the seven strains have components of a cytochrome bd complex. It seems strange that these anaerobes possess aerobic machineries for respiration, but it has been shown that BF encodes a cytochrome bd that is essential for the consumption of O_2_ and is pivotal for growth stimulation in the presence of nanomolar concentrations of O_2_ [66]_._ The strains also possess components similar to the Na^+^-translocating NADH-quinone oxidoreductase, NqrABCDEF (TC# 3.D.5.1.1) of *Vibrio alginolyticus*; the OP strain BD contains six characterized components of this seven-component system. This NQR complex catalyzes the reduction of ubiquinone to ubiquinol in two successive reactions, coupled with the transport of Na^+^ from the cytoplasm to the periplasm [67]. BO, BT, BC and BV have components of succinate dehydrogenase, SdhABC, a type E succinate:quinone oxidoreductase (SQR) (TC#3.D.10.1.4). Additionally, intact subunits of a three-subunit menaquinone:fumarate oxidoreductase (QfrABC) (TC# 3.D.10.1.5) are present in six of the strains. It has been proposed in an electron transport chain (ETC) model that *Bacteroides* consist of two main enzyme complexes, NADH dehydrogenase and fumarate reductase, which are responsible for shuttling electrons along the membrane [65]. As mentioned above, our results indicate the presence of fumarate reductase in the ETCs of all seven *Bacteroides* strains.

### 3.9 Possible group translocators (TC class 4)

Five of the strains have a single homologue of a galactitol IIC protein (TC#4.A.5.1.4). However, this homologue cannot function by a PTS dependent mechanism as the seven strains lack PTS phosphoryl transfer proteins. The encoding genes are in monocistronic operons with good promoters, indicating that these IIC homologues may function as secondary carriers. In accordance with the observations of Barabote and Saier, (2005) [9], BF lacks identifiable genes that encode PTS homologues, and according to our results, BC lacks them too. Three strains, BT, BO and BC, have a thiamine uptake permease (TC# 4.B.1.1.4); the gene encoding this transporter, *pnuT*, is regulated by a TPP riboswitch, clearly suggesting that it is a thiamine carrier [68]. Each strain has 2–11 polysaccharide synthase/exporters (TC#4.D). The proposed function of these transporters is to catalyze vectorial glycosyl polymerization.

### 3.10 Auxiliary transporters (TC subclass 8.A)

TC subclass 8.A contains transporters that facilitate transport across the membrane in some way, but do not participate directly in the transport process. They function in conjunction with one or several established transport systems. In all the strains, the Glycan-Binding Protein (SusD) Family (TC#8.A.46) is well represented with numerous homologues. All seven strains have homologues of (TC#8.A.46.1.1), a starch and maltooligosaccharide binding protein of 551 aas that functions in complex with a TonB-dependent SusC outer membrane receptor (TC#1.B.14.6.1); the SusC protein transports the oligosaccharides into the periplasm where they are broken down into monosaccharides and disaccharides by the α-amylase SusA and an α-glucosidase SusB [6, 69, 70]. Another glycan binding protein homologue, present in all of the strains, is a high affinity sialic acid binding protein; NanU (extracellular neuraminate uptake protein) of 516 aas (TC#8.A.46.1.4). This protein, along with NanO, has been predicted to be part of the NanOU transport system in various bacterial species. However, NanU shares more sequence similarity with the SusD family than with NanO [71].

### 3.11 Poorly characterized transporters (TC subclass 9.A)

TC subclass 9.A includes known transport systems that function by an unknown mechanism of action. Six of the strains (with the exception of BO) have a single member of the FeoB family of ferrous iron uptake transporters (TC#9.A.8). The TC ferrous iron transport protein B (TC#9.A.8.1.3) has 827 aas with 9 TMSs, and it is a Fe^2+^ uptake system either driven by GTP hydrolysis or positively regulated by GTP [72].

### 3.12 Putative transport proteins (TC subclass 9.B)

Uncharacterized putative transport proteins are grouped into this subclass and will either be classified in TCDB when the transport function of a member becomes established, or will be eliminated from the TC classification system if the proposed transport function is disproven. Numerous putative transporters are present in all seven strains. This includes a glycosyl transferase GumD (TC# 9.B.18.1.1) that is absent in BF but present in the remaining six strains. The functions of this putative transporter have been suggested to be gum polysaccharide synthesis and export [73]. BF uniquely also lacks transporters of the Putative Mg^2+^ Transporter-C (MgtC) Family (TC#9.B.20) and the DedA Family (TC#9.B.27).

### 3.13. Differences in substrates transported by PAP and OP strains

To gain a better understanding of the transport systems of PAP and OP *Bacteroides*, the probable substrate specificities of the transporters were predicted as presented in the Supporting information section (S1 Table) and Table 6. By looking at Table 6, it can be concluded that on the average, PAP strains contain more transport proteins (250–360) of unknown function as compared to the OP strains (142–321). Similarly, the PAP strains contain more protein and peptide transporters (8–13) in comparison to the OP strains (4–13). Most of the peptides transported are bacteriocins including homologs of enterocin and colicins. However, the OP strains are more varied in their cation transport capacities than their PAP counterparts (44–78 versus 28–76). This indicates a better ability of the OP strains to maintain ionic homeostasis, osmotic regulation and heavy metal resistance. In all the strains, there are fewer anion transporters than cationic porters. Anion transporters are found primarily in TC subclass 2.A, taking up or exporting bicarbonate, phosphate, arsenate, arsenite, tellurite, chromate, chloride and fluoride. Sulfate uptake, on the other hand, is mediated primarily by homologs of the CysPTWA ATP-dependent ABC system. All seven strains have a carboxylate transporter range of 1–10 with BV having the former and BC the latter. These proteins are probably concerned with the transport of malate, succinate, fumarate, aspartate, methylmalonyl-CoA and D-mannuronate. This fact is in agreement with the anaerobic lifestyle of these organisms. The numbers of sugar transporters are disproportionally distributed among both PAP and OP strains (25–67 versus 26–90). The most commonly transported sugars by both types include maltooligosaccharide, arabinose, glucosamine, glucose, mannose and xylose. As *Bacteroides* live in the body extracellularly, they take up nutrients that are readily available in the extracellular environment.

**Table 6:** Overview of predicted substrate specificities of transport proteins in the seven *Bacteroides* species.

Substrate category	BD	BC	BS	BT	BF	BV	BO
Inorganic Anions	7	11	9	16	13	7	7
Inorganic Cations	52	78	44	76	56	28	31
Amines	4	4	0	4	4	5	6
Amino acids	12	18	6	13	13	11	7
Carboxylates	10	5	4	7	4	1	4
Non-Selective	16	8	8	12	9	5	7
Drugs	45	49	30	61	38	38	30
Nucleobases, Nucleosides, Nucleotides	21	36	29	33	21	29	28
Proteins, Peptides	13	12	4	13	11	8	8
Siderophores	0	1	0	2	1	0	1
Sugars	38	90	26	67	46	29	25
Sugar Derivatives	3	6	6	7	1	6	5
Lipids	0	5	0	3	8	0	1
Sugar Alcohols	1	0	1	2	3	0	2
Vitamins	3	12	3	14	8	5	8
Unknown	233	321	142	360	279	250	338
Total	458	656	312	690	515	422	508

The PAP strain BT has the most (61) transport proteins involved in drug export. This may indicate that out of all the strains, BT is the most antibiotic resistant. In the remaining six strains, both PAP and OP, the numbers of drug transport proteins are less than BT with a range of 30–49 proteins. Interestingly, both PAP and OP strains contain large numbers of transport proteins for nucleotides, nucleobases and nucleosides; this includes a range of 21–36 in the OP strains and 21–33 in the PAP strains.

### 3.14 Transporters that are likely to contribute to pathogenesis are found in both PAP and OP strains

Both types of strains contain components of a T6SS, and interestingly, the OP strains contain more components of T6SS than the PAP strains. T6SSs are hypothesized to contribute to the virulence of some bacterial pathogens, both through the delivery of protein substrates to host cells, and by secreting substrates into neighboring bacteria that may compete to exploit specific host niches [74]. However, they are also known to allow competition between bacteria, favoring the ones bearing a functional T6SS. Consequently, the T6SS in the OP strains may be employed as an advantageous mechanism to allow successful competition with pathogens.

Components hitting at least six types of T4SS in TCDB are present equally in all seven *Bacteroides* strains. While T4SSs are capable of transferring both DNA and proteins, they can serve a variety of functions, including the conjugative transfer of DNA, and translocation of effector proteins or DNA/protein complexes directly into recipient cells. These systems, similar to T3SS, can play pivotal roles in pathogenesis [75]. There are also disparate patterns for the distribution of toxins among the strains, with the presence of hemolysins, membrane attack complex/perforins and pore-forming amphipathic helical peptide (HP2-20) in both types of strains.

### 3.15 Major families/superfamilies found in the seven *Bacteroides* species

Six families are the most highly represented in the seven *Bacteroides* strains examined as shown in Table 7. Of particular interest is the high numbers of transport proteins of the Outer Membrane Receptor (OMR) Family (TC#1.B.14) and the Glycan-binding Protein (SusD) Family (TC#8.A.46). The number of transport proteins of the OMR family is between 22–57 with a percentage of 5.9–8.5% of the total transport proteins. While the range of proteins of the SusD family is 30–112, and the percentages of these proteins relative to the total transport proteins is 9.6–22% in the seven strains. The high representation of both families in all seven *Bacteroides* strains points towards their ability to work as very effective probiotic machines. In comparison to the number of these families observed in the probiotic strains of *E*. *coli* in the study by Do et al [12], the numbers are much higher in the species of this study. This leads to the prediction that *Bacteroides* have developed sophisticated mechanisms to bind, degrade and transport sugars into the cytoplasms and their external environments. This enhanced ability to metabolize sugar can also be attributed to the presence of high numbers of polysaccharide utilization loci (PUL) in the genomes of *Bacteroides* species. The PUL can be defined as a set of physically linked genes organized around SusCD gene pairs. The presence of high numbers of PUL gives *Bacteroides* species an evolutionary advantage over other microbes, in orchestrating the breakdown of a wide array of glycans in the gut [1]

**Table 7:** Tabulation of the largest transport protein families encoded within the genomes of the genomes of the seven *Bacteroides* species. Both the total number of proteins and average percentage of major families are shown.

Family name, abbreviation and TC#	BD	BC	BS	BT	BF	BV	BO	Total #	BD	BC	BS	BT	BF	BV	BO	Average %
The Outer Membrane Receptor (OMR) Family (TC# 1.B.14)	28	39	22	57	38	28	43	255	6.1	5.9	7.1	8.3	7.4	6.6	3.4	7.1
The Major Facilitator Superfamily (MFS) (TC#2.A.1)	20	27	20	30	28	20	22	167	4.4	4.1	6.4	4.3	5.4	4.7	4.3	4.8
The Resistance-nodulation-Cell-Division (RND) Superfamily (TC#2.A.6)	22	27	11	45	18	19	14	156	4.8	4.1	3.5	6.5	3.5	4.5	2.8	4.2
The Multi drug/Oligosaccharidyl-lipid/Polysaccharide (MOP) Flippase Superfamily (TC#2.A.66)	15	22	13	18	19	15	16	118	3.3	3.4	4.2	2.6	3.7	3.6	3.1	3.4
The ATP-binding Cassette (ABC) Superfamily (TC#3.A.1)	70	91	30	76	77	55	57	456	15.3	13.9	9.6	11.0	15.0	13	11.2	12.7
The Glycan-binding protein Family (TC#8.A.46)	75	94	30	97	62	77	112	547	16.3	14.3	9.6	14.1	12.0	18.2	22	15.2
Totals	230	300	126	323	242	214	264	1699/3561	50.3	45.7	40.4	46.8	47	50.6	52	47.4

### 3.16 Comparison of sequence similarity distributions between orthologous protein sets of transporter and non-transporters proteins

The proteome of each genome was divided into two sets: transporters and non-transporters. Transporters were defined as all proteins identified by the methodology described in this paper, plus all proteins with at least 4 predicted TMSs (using HMMTOP), even if they had no sequence similarity with proteins in TCDB. All other proteins were regarded as non-transporters. Sets of candidate orthologous proteins were identified between pairs of genomes as reciprocal best hits (E-value < 1×10^−5^ and coverage ≥ 80% of the shorter protein). We recorded the percentage of sequence identity between pairs of orthologous proteins among all seven *Bacteroides* genomes. The identity distributions are bimodal (see S1 and S2 Figs), with the smaller peak around 35–40% identity and the higher peak around 80–85% identity. Since these genomes are closely related, it is not surprising that most orthologs show high degrees of sequence identity (mean > 65%). However, notice that non-transporters have a larger proportion of orthologues with identities less than 55% as compared to transporters. Such a difference in proportions is large enough to render the two distributions of identities significantly different both between pairs of genomes and when comparing the global distributions of identities across all genomes (Kolmogorov–Smirnov test P-value < 1×10^−55^ and Mann–Whitney *U* test P-value < 1×10^−26^). Notice that although the means of both distributions are similar (μ = 70.9 vs μ = 67.2), the differences in the shape of the distributions are apparent for lower identities (see S1 and S2 Figs).

## Discussion

Over many millennia, the human gut has co-evolved with various bacteria, viruses, archaea and eukaryotes, collectively termed “the gut microbiome’, to form an intricate association that is usually beneficial for both the host and the microbes [76]. Members of this association can be mutualists, commensals and pathobionts, depending upon their metabolic repertoires and bio-geographical locations [1]. These microbes, present in the human gut, can be regarded as a separate, fully developed and functional metabolic organ that contributes to nutrient absorption, metabolism, susceptibility and resistance to various kinds of diseases, xenobiotic responses and immune system modulation [77, 78]. Recent advances in sequencing techniques, metagenomics and proteomics have given us a plethora of information about the interactions taking place between the host cells and microbial residents.

*Bacteroides* is a prominent genus of the gut microbiome, and its members are well-established probiotic strains, but they have the tendency to be pathogenic when they escape from the gut and reach other locations in the human body [79]. Clinically, *Bacteroides* species are the most commonly isolated anaerobic pathogens for certain types of infection, and in some cases, they cause life-threatening conditions such as intra-abdominal, brain and lung abscesses and sepsis [80]. Members of this genus are also considered to be the most antibiotic resistant anaerobic pathogens known, and they exhibit resistance to a wide range of antibiotics including fluoroquinolones, carbapenems, metronidazole, cefoxitin and clindamycin. In some instances, these bacteria show 100% resistance to β-lactam derivatives and tetracycline [81].

The genomes of seven *Bacteroides* strains (four PAP and three OP) were screened against TCDB to identify homologues of (putative) transport proteins. Based on our current knowledge of the systems, complete or nearly complete sets of transport systems were identified. As previously reported by Tang and Saier, and Do et al, [11, 12], probiotic species often live extracellularly in the gut and express transport system genes that give them advantages for extracellular survival. The strains in our study follow the probiotic lifestyle, utilizing many metabolites such as polysaccharides, oligosaccharides and vitamins, in some cases converting them to compounds useful to the hosts.

All of the PAP and OP strains contain homologues of pore-forming toxins (PFTs) including members of the MACPF family. These toxins may be utilized by the strains for the killing of other resident gut microbes, and they may target pathogens trying to colonize the gut, thus proving to be an attribute of their probiotic nature. MACPF domains have been reported to be present in the Bacteriodales secreted antimicrobial protein (BSAP-1) of *B*. *fragilis* (BF). It has been shown that this protein is involved in the killing of closely related strains *in vivo* and *in vitro* [82]. The presence of these PFTs may contribute directly to the pathogenicity of these organisms when present outside of the gut.

Studies have shown that BF can evade the immune response of peritoneal macrophages (PMs) after extrusion from the gut into the peritoneal cavity using PFTs [83]. The main mechanism by which PMs kill bacteria involves the production of nitric oxide (NO). NO is produced from the inducible nitrate synthase (iNOS) on exposure of PMs to different cytokines. However, interaction of the PMs with BF results in decreased production of both NO and iNOS. Also, in the cytoskeleton of the PMs, a colocalization of iNOS and actin filaments occurs. The ability of BF to utilize these PFTs to cause pore formation in the PMs can lead to the formation of actin filament and iNOS extrusion through these pores, which in turn can assist BF to evade the host immune response. Also, the BF strain in our study has a homologue of the Hemolysin III of *Bacillus cereus*. These types of toxins are considered to be powerful virulence determinants in bacteria, and offer a competitive edge to these bacteria by killing leukocytes, promoting survival and providing assistance in nutrient acquisition [84].

All of the *Bacteroides* strains examined contain components of the different types of protein secretory systems. Noteworthy, is the finding that all of the strains contain components of T6SS, but according to the information in TCDB, these systems may be incomplete. This may be due to: 1) some of the components could not be identified using our approaches, 2) the components differ in different bacteria leading to the possibility that these systems are in fact complete, and 3) the common ancestor may have possessed more than one such system, but their components were partially lost or shuffled by the progeny. T6SSs are of particular interest in relation to the fitness of the strains in the intestine, as these systems have been shown to antagonize both commensals and competitors, thus proving to be advantageous in hostile environments. Upon direct contact with other bacteria, the T6SS may inject antibacterial toxins into the target cell [85]. Interestingly, a T6SS is not only beneficial for the bacterium expressing it but can also be of benefit to the host, as it can provide protection against pathogens. It has been shown by Hecht et al. that the T6SS can be used by a probiotic non-toxigenic strain of BF to antagonize a toxigenic strain of BF in an *in vitro* experiment using mice [86].

In the anaerobic environment of the human intestine there is intense competition among microbial residents to acquire iron. The process of iron sequestration provides an important mechanism to limit colonization by pathogens. *Bacteroides* species seem to lack siderophores [87], but they exist in a densely populated and competitive environment in which other organisms do produce siderophores. Notable is the fact that iron and heme are regarded as essential nutrients for *Bacteroides*. To counteract their lack of siderophore production, these bacteria have the ability to utilize xenosiderophores (siderophores from other organisms) [88]. Recent studies have shown that the acquisition of Fe^3+^ via siderophores plays an important role in colonization of the gut by bacteria. Interestingly, in our study, transporters for Fe^3+^-siderophores are present in the three PAP strains (BT, BV and BO), although the OP strains lack recognizable iron siderophore transporters. This may be a distinguishing feature, allowing pathogenicity in non-gastrointestinal tract tissues.

As mentioned earlier, *Bacteroides* are opportunistic pathogens, and their pathogenicity depends on their locations in the body. However, recent studies have indicated that these bacteria may assist other pathogens to cause infection in the gut. The huge repertoire of sugar transport systems (see Results section) at their disposal makes this reasonably easy to accomplish. For instance, *Bacteroides* can liberate fucose, sialic acid and other sugars from glycoproteins, and these in turn can be utilized by enteric pathogens such as the entero-hemorrhagic *E*. *coli* (EHEC), facilitating the expression of their virulence genes [89].

Comparison of the transport proteins of the seven strains included in this study with the strains of *E*. *coli* (two extracellular pathogens, two intracellular pathogens, two probiotics and a commensal), as well two intracellular pathogens of *Salmonella* from our previous study [12], reveals similarities and striking differences. For instance, the percent of α -type channels in *E*. *coli* and *Salmonella* strains ranges from 3.4 to 4.2% similar to that in the *Bacteroides* strains (2.9–5.4%), but in *Bacteroides*, the range of β-barrel proteins is 10.5–15.7%, which is considerably more than the *E*. *coli* and *Salmonella* strains (7.2–9.9%). The reason for this is the presence of numerous proteins of the OMR family in *Bacteroides* that are concerned with the binding and uptake of sugars, mostly oligosaccharides. There is no major difference among the PFTs (0.3–0.9% for *Bacteroides* and 0.2–1.4% for *E*. *coli* and *Salmonella*). Only the *Bacteroides* strains contain members of the Membrane Attack Complex/Perforin Family (MACPF), since *E*. *coli* and *Salmonella* lack members of this family. No probiotic specific PFTs were found in *Bacteroides*, but the *E*. *coli* strain Nissle has one. *E*. *coli* and *Salmonella* have more holins (0.8–1.3%) than the *Bacteroides* strains (0.5–0.8%). A larger percent of porters are present in the *E*. *coli* and *Salmonella* strains (27.4–32.5%) as compared to the *Bacteroides* strains (20–26.6%). This indicates a better metabolic repertoire in terms of secondary carriers in *E*. *coli* and *Salmonella*, and may correlate with the preferred carbon metabolic pathways used by these organisms. Higher percentages of primary pyrophosphate hydrolysis driven transporters are found in *E*. *coli* and *Salmonella* strains (26.9–32.5%) while the *Bacteroides* strains have 20.3–26.6%. The former two species have more uptake and efflux systems as compared to *Bacteroides*, but the *Bacteroides* have more components of the T1SS and T4SS, while components of the T3SS and T6SS are better characterized in the *E*. *coli* and *Salmonella* strains, consistent with the two latter species having strong pathogenic character. The percent of oxidoreduction-driven transporters is similar (*E*. *coli* and *Salmonella*, 4.1–5.9% and *Bacteroides* 4.7–6.7%*)*. However, the *Bacteroides* strains contain more auxiliary transport proteins (12.2–23%) in comparison to the 1.7–2.6% of the *E*. *coli* and *Salmonella* strains. This includes numerous homologues of the Glycan Binding Protein family (SusD) (TC#8.A.46), thus highlighting superior mechanisms of polysaccharide binding and utilization in *Bacteroides*. Low percentages of poorly characterized but known transporters (subclass 9.A) are similar across all three groups of organisms (*Bacteroides* 0.3–1.1%; *E*. *coli* and *Salmonella* 0.8–1.2%).

The occurrence of high numbers of homologues of SusD and SusC observed in this study is interesting and can be related to the presence of PUL in these seven strains as mentioned in the Results section. [1]. However, of interest is the finding that these seven *Bacteroides* strains contain larger numbers of proteins of the SusD family (ranging from 30–112), with the PAP strain BO surprisingly having the most number (112) of these proteins, while the range of SusC proteins (OMR family) is much less than that of the SusD proteins (3–5). This unequal correlation of both SusD and SusC proteins is intriguing and will the focus of our future research on *Bacteroides*, as future studies on other strains could provide more information about the presence of the above mentioned proteins.

In S1 and S2 Figs, we compared putative orthologous transport-related proteins with all remaining putative orthologous (non-transporter) proteins encoded within the seven genomes. The results revealed that orthologous transporter proteins from *Bacteroides* strains showed noticeably lower percentages of distantly related proteins (identity < 55%) as compared to all other proteins encoded within the genomes. This suggests that transport proteins have diverged in sequence at lower rates than non-transport proteins, which correlates with the fact that TM segments of integral membrane proteins generally diverge less rapidly than the hydrophilic portions of the same proteins [90]. This observation can be explained, at least in part, by the physicochemical constraints in the membrane environment that limit the type of substituting residues in TMSs [91]

In the present study, the transport proteins comprised more than 10% of the proteomes of all seven *Bacteroides* strains. The overall transportome (total transport proteins) revealed key characteristics of metabolism, pathogenicity and mutualism. Both OP and PAP strains of *Bacteroides* contain virulence factors, indicating yet undisclosed hidden pathogenic potential of the OP strains. Virulence factors include the holins, PFTs, T6SS, T3SS and various multidrug efflux pumps. Recent studies have shown that most of the factors in *Bacteroides* related to pathogenesis were acquired though horizontal gene transfer (HGT) [92, 93]. However, the G-Blast program lacks the ability to detect HGT, and future additions will be made. Further analysis of the PAP strains will be necessary to detect the environmental and immunological triggers that cause these strains to have a duel nature. Further analysis of drug transporters will also be beneficial, allowing improvements to our understanding of the antibiotic resistances in these strains. Further efforts may help to identify disease conditions that could be caused by the OP strains. Based on the data presented here, the transporters identified in these strains should be of value in the development of therapeutic treatments for *Bacteroides* pathogens.

## Supporting information

S1 Fig Distribution of percent identities between putative orthologs of “transporters”. “Transporter” proteins refer to all proteins retrieved using the methodology described in this paper plus any other integral membrane protein with at least 4 predicted TMSs. The histogram shows the distribution of percent identities between putative orthologous “Transporter” proteins (μ = 70.9 and σ = 15.7).
(JPG)

S2 Fig Distribution of percent identities between putative orthologs of “non-transporter” proteins. “Transporter” proteins refer to all proteins retrieved using the methodology described in this paper plus any other integral membrane protein with at least 4 predicted TMSs. “Non-transporter” proteins are defined as all remaining proteins encoded by the genome. The histogram shows the distribution of percent identities between putative orthologous “Non-transporter” proteins (μ = 67.2 and σ = 19.3). Notice the higher proportion of “non-transporter” orthologous proteins with identities < 55% as compared to the “transporter” proteins in S1 Fig.
(JPG)

S1 Table Transport proteins and substrates of all seven *Bacteroides* strains. Homologues of transport proteins are marked in blue.
(DOCX)
