# Supplementary figures and images for: Comparative genomics of transport proteins in seven Bacteroides species
Source: PLoS One. 2018 Dec 5;13(12):e0208151. doi: 10.1371/journal.pone.0208151 (PMC6281302; doi:10.1371/journal.pone.0208151)

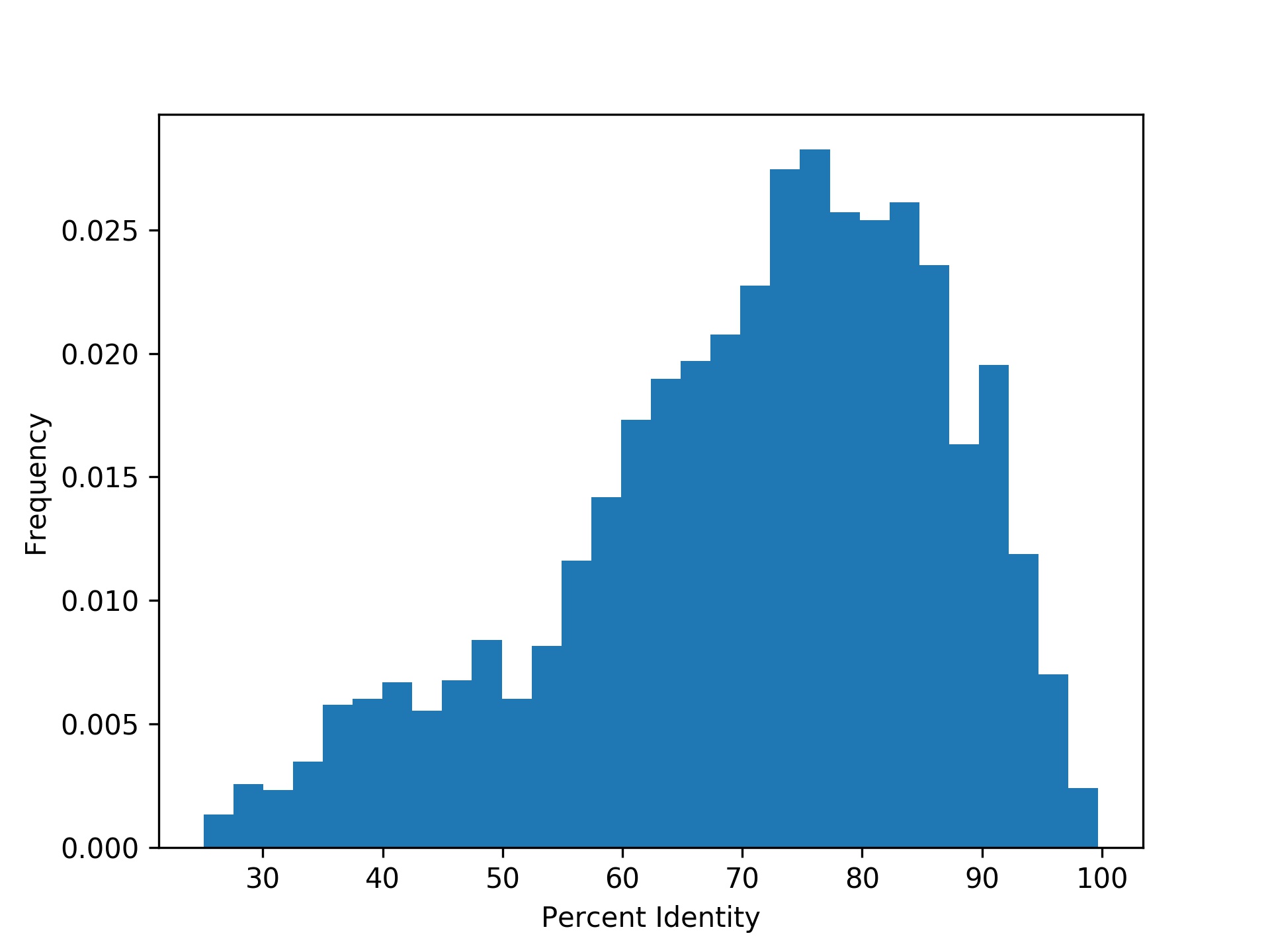

Supplement: S1 Fig — “Transporter” proteins refer to all proteins retrieved using the methodology described in this paper plus any other integral membrane protein with at least 4 predicted TMSs. The histogram shows the distribution of percent identities between putative orthologous “Transporter” proteins (μ = 70.9 and σ = 15.7). (JPG) [file pone.0208151.s001.jpg]

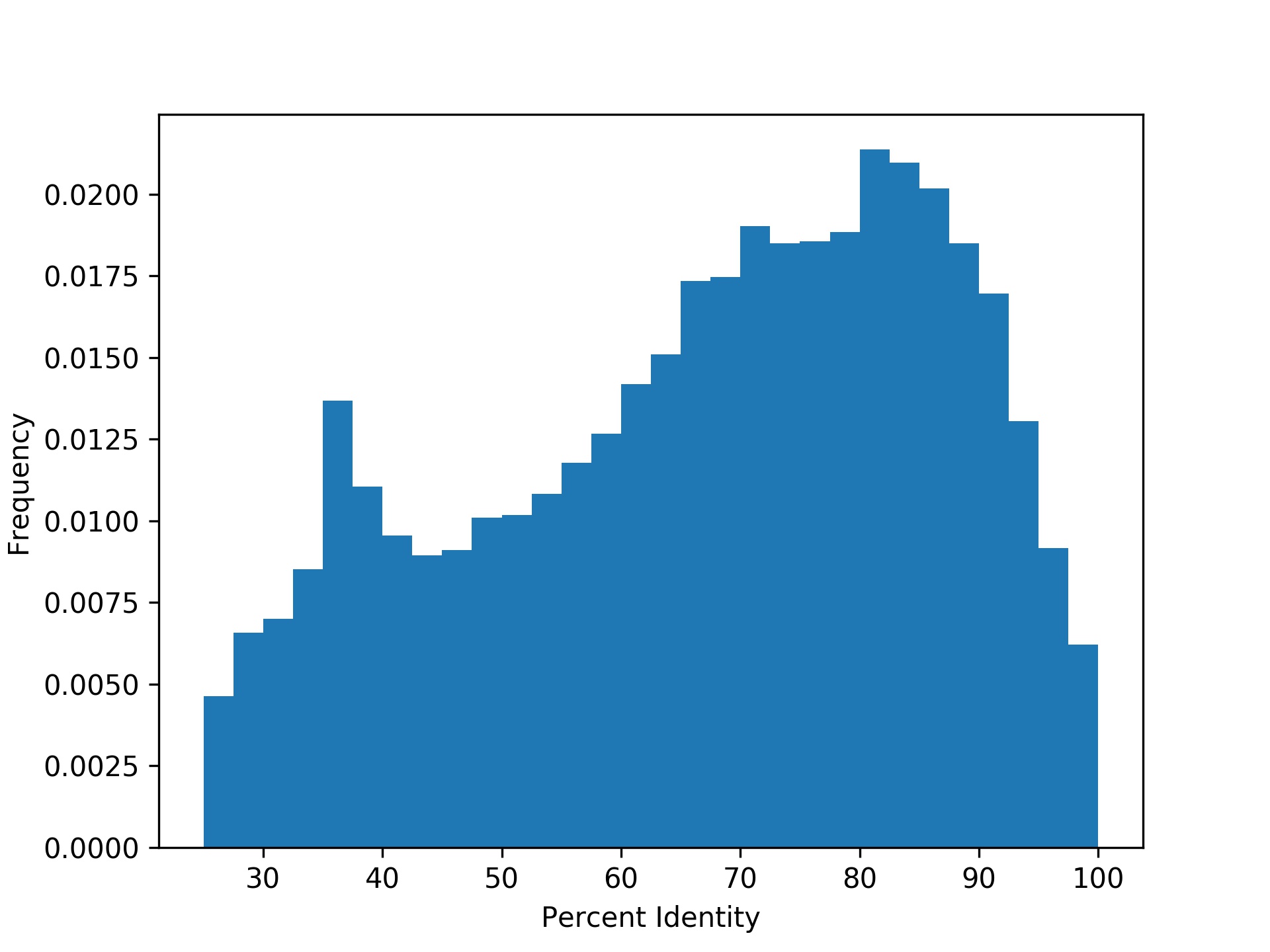

Supplement: S2 Fig — “Transporter” proteins refer to all proteins retrieved using the methodology described in this paper plus any other integral membrane protein with at least 4 predicted TMSs. “Non-transporter” proteins are defined as all remaining proteins encoded by the genome. The histogram shows the distribution of percent identities between putative orthologous “Non-transporter” proteins (μ = 67.2 and σ = 19.3). Notice the higher proportion of “non-transporter” orthologous proteins with identities < 55% as compared to the “transporter” proteins in S1 Fig. (JPG) [file pone.0208151.s002.jpg]
