# Supplementary material for: Comparative genomics of transport proteins in seven Bacteroides species
Source: PLoS One. 2018 Dec 5;13(12):e0208151. doi: 10.1371/journal.pone.0208151 (PMC6281302; doi:10.1371/journal.pone.0208151)
Supplement: S1 Table — Homologues of transport proteins are marked in blue. (DOCX) [file pone.0208151.s003.docx]

| **NCBI accession #** | **BD** | **BS** | **BC** | **BT** | **BF** | **BO** | **BV** | **TCID** | **E-value** | **Uniprot accession #** | **General substrate** | **Specific substrate** |
| --- | --- | --- | --- | --- | --- | --- | --- | --- | --- | --- | --- | --- |
| WP_008765568.1 |  |  |  | + |  |  |  | 1.A.1.5.25 | -09 | D6ZSR9 | Unknown | Unknown |
| WP_011109206.1 |  |  |  | + |  |  |  | 1.A.1.5.25 | -07 | D6ZSR9 | Unknown | Unknown |
| YP_099429.1 |  |  |  |  | + |  |  | [1.A.1.5.25](http://tcdb.org/search/result.php?tc=1.A.1.5.25) | -07 | D6ZSR9 | Unknown | Unknown |
| WP_011108359.1 |  |  |  | + |  |  |  | 1.A.1.17.2 | -11 | Q8Y5K1 | Cations | K^+^ |
| YP_097823.1 |  |  |  |  | + |  |  | [1.A.1.17.2](http://tcdb.org/search/result.php?tc=1.A.1.17.2) | -13 | Q8Y5K1 | Anions | K^-^ |
| [WP_004296272.1](file:///Volumes/ESD-USB/Summer%202018/Hassan%20Submission%20PLosOne/content.html#lcl|NZ_CP012938.1_prot_WP_004296272.1_2584) |  |  |  |  |  | + |  | [1.A.1.17.2](http://tcdb.org/search/result.php?tc=1.A.1.17.2) | -11 | Q8Y5K1 | Unknown | Unknown |
| WP_022209091.1 |  |  | + |  |  |  |  | [1.A.1.24.3](http://tcdb.org/search/result.php?tc=1.A.1.24.3) | -86 | Q1D027 | Cations | K^+^ |
| [WP_004301970.1](file:///Volumes/ESD-USB/Summer%202018/Hassan%20Submission%20PLosOne/content.html#lcl|NZ_CP012938.1_prot_WP_004301970.1_4522) |  |  |  |  |  | + |  | [1.A.1.24.3](http://tcdb.org/search/result.php?tc=1.A.33.1.2) | -89 | Q1D027 | Unknown | Unknown |
| WP_048694701.1 |  |  |  | + |  |  |  | 1.A.1.24.3 | -87 | Q1D027 | Cations | K^+^ |
| YP_100843.1 |  |  |  |  | + |  |  | [1.A.1.24.3](http://tcdb.org/search/result.php?tc=1.A.1.24.3) | -85 | Q1D027 | Anions | K^-^ |
| [WP_005850728.1](file:///Volumes/ESD-USB/Summer%202018/Library/Application%20Support/Microsoft/Office/Office%202011%20AutoRecovery/content.html#lcl|NC_009614.1_prot_WP_005850728.1_597) |  |  |  |  |  |  | + | [1.A.1.24.3](http://tcdb.org/search/result.php?tc=1.A.1.24.3) | -82 | Q1D027 | Unknown | Unknown |
| [WP_013617521.1](file:///Volumes/ESD-USB/Summer%202018/Hassan%20Submission%20PLosOne/content.html#lcl|NC_015164.1_prot_WP_013617521.1_1487) |  | + |  |  |  |  |  | [1.A.8.3.1](http://tcdb.org/search/result.php?tc=1.A.8.3.1) | -51 | P60844 | [Water](https://www.ebi.ac.uk/chebi/searchId.do;jsessionid=A9D16DCB24C6F74339FC28A4941EFBB6?chebiId=CHEBI:15377) | Water |
| WP_032841205.1 |  |  |  | + |  |  |  | 1.A.8.3.1 | -52 | P60844 | Unknown | Unknown |
| [WP_004302280.1](file:///Volumes/ESD-USB/Summer%202018/Hassan%20Submission%20PLosOne/content.html#lcl|NZ_CP012938.1_prot_WP_004302280.1_4765) |  |  |  |  |  | + |  | [1.A.8.3.1](http://tcdb.org/search/result.php?tc=1.A.33.1.2) | -52 | P60844 | [Water](https://www.ebi.ac.uk/chebi/searchId.do;jsessionid=A9D16DCB24C6F74339FC28A4941EFBB6?chebiId=CHEBI:15377) | Water |
| [WP_004296155.1](file:///Volumes/ESD-USB/Summer%202018/Hassan%20Submission%20PLosOne/content.html#lcl|NZ_CP012938.1_prot_WP_004296155.1_2485) |  |  |  |  |  | + |  | [1.A.8.13.3](http://tcdb.org/search/result.php?tc=1.A.33.1.2) | -46 | D7VF10 | Sugar alcohols | Glycerol/Dihydroxyacetone |
| [WP_041584404.1](file:///Volumes/ESD-USB/Summer%202018/Hassan%20Submission%20PLosOne/content.html#lcl|NC_015164.1_prot_WP_041584404.1_2443) |  | + |  |  |  |  |  | [1.A.11.1.4](http://tcdb.org/search/result.php?tc=1.A.11.1.4) | -132 | O67997 | Water | NH_4_^+^ |
| WP_008765058.1 |  |  |  | + |  |  |  | 1.A.11.2.3 | -111 | Q93IP6 | Cations | NH_4_^+^ |
| ABI39_14260 | + |  |  |  |  |  |  | 1.A.11.2.3 | -105 | Q93IP6 | Cations | NH_4_^+^ |
| [WP_012055979.1](file:///Volumes/ESD-USB/Summer%202018/Library/Application%20Support/Microsoft/Office/Office%202011%20AutoRecovery/content.html#lcl|NC_009614.1_prot_WP_012055979.1_3903) |  |  |  |  |  |  | + | [1.A.11.2.3](http://tcdb.org/search/result.php?tc=1.A.11.2.3) | -107 | Q93IP6 | Cations | NH_4_^+^ |
| [WP_029428195.1](file:///Volumes/ESD-USB/Summer%202018/Hassan%20Submission%20PLosOne/content.html#lcl|NZ_CP012801.1_prot_WP_029428195.1_5024) |  |  | + |  |  |  |  | [1.A.11.2.7](http://tcdb.org/search/result.php?tc=1.A.11.2.7) | -107 | O28528 | Cations | NH_4_^+^ |
| WP_011109265.1 |  |  |  | + |  |  |  | 1.A.13.2.3 | -134 | P76481 | Anions | Cl^-^ |
| YP_100866.1 |  |  |  |  | + |  |  | [1.A.13.2.3](http://tcdb.org/search/result.php?tc=1.A.13.2.3) | -139 | P76481 | Anions | K^-^ |
| [WP_004301951.1](file:///Volumes/ESD-USB/Summer%202018/Hassan%20Submission%20PLosOne/content.html#lcl|NZ_CP012938.1_prot_WP_004301951.1_4506) |  |  |  |  |  | + |  | [1.A.13.2.3](http://tcdb.org/search/result.php?tc=1.A.33.1.2) | -138 | P76481 | [Anions](https://www.ebi.ac.uk/chebi/searchId.do;jsessionid=A9D16DCB24C6F74339FC28A4941EFBB6?chebiId=CHEBI:17996) | [Anions](https://www.ebi.ac.uk/chebi/searchId.do;jsessionid=A9D16DCB24C6F74339FC28A4941EFBB6?chebiId=CHEBI:17996) |
| [WP_013619174.1](file:///Volumes/ESD-USB/Summer%202018/Hassan%20Submission%20PLosOne/content.html#lcl|NC_015164.1_prot_WP_013619174.1_3227) |  | + |  |  |  |  |  | [1.A.14.2.2](http://tcdb.org/search/result.php?tc=1.A.14.2.2) | -69 | P0AAC4 | Anions | CH3COO^−^ |
| ABI39_17975 | + |  |  |  |  |  |  | 1.A.22.1.1 | -57 | P0A742 | Osmolytes | Osmolytes |
| [WP_007215416.1](file:///Volumes/ESD-USB/Summer%202018/Hassan%20Submission%20PLosOne/content.html#lcl|NZ_CP012801.1_prot_WP_007215416.1_4027) |  |  |  |  |  |  |  | [1.A.22.1.1](http://tcdb.org/search/result.php?tc=1.A.22.1.1) | -53 | P0A742 | Unknown | Unknown |
| [WP_013618527.1](file:///Volumes/ESD-USB/Summer%202018/Hassan%20Submission%20PLosOne/content.html#lcl|NC_015164.1_prot_WP_013618527.1_2574) |  | + |  |  |  |  |  | [1.A.22.1.1](http://tcdb.org/search/result.php?tc=1.A.22.1.1) | -47 | P0A742 | Unknown | Unknown |
| WP_008759941.1 |  |  |  | + |  |  |  | 1.A.22.1.1 | -52 | P0A742 | Unknown | Unknown |
| YP_098252.1 |  |  |  |  | + |  |  | [1.A.22.1.1](http://tcdb.org/search/result.php?tc=1.A.22.1.1) | -54 | P0A742 | Osmolytes | Osmolytes |
| [WP_004301777.1](file:///Volumes/ESD-USB/Summer%202018/Hassan%20Submission%20PLosOne/content.html#lcl|NZ_CP012938.1_prot_WP_004301777.1_4354) |  |  |  |  |  | + |  | [1.A.22.1.1](http://tcdb.org/search/result.php?tc=1.A.33.1.2) | -54 | P0A742 | Unknown | Unknown |
| [WP_005839284.1](file:///Volumes/ESD-USB/Summer%202018/Library/Application%20Support/Microsoft/Office/Office%202011%20AutoRecovery/content.html#lcl|NC_009614.1_prot_WP_005839284.1_3505) |  |  |  |  |  |  | + | [1.A.22.1.1](http://tcdb.org/search/result.php?tc=1.A.22.1.1) | -56 | P0A742 | Osmolytes | Osmolytes |
| [WP_029426456.1](file:///Volumes/ESD-USB/Summer%202018/Hassan%20Submission%20PLosOne/content.html#lcl|NZ_CP012801.1_prot_WP_029426456.1_2871) |  |  | + |  |  |  |  | [1.A.23.1.4](http://tcdb.org/search/result.php?tc=1.A.23.1.4) | -120 | M4V7W7 | Unknown | Unknown |
| WP_011108587.1 |  |  |  | + |  |  |  | 1.A.23.1.4 | -122 | M4V7W7 | Unknown | Unknown |
| YP_101813.1 |  |  |  |  | + |  |  | [1.A.23.1.4](http://tcdb.org/search/result.php?tc=1.A.23.1.4) | -129 | M4V7W7 | Unknown | Unknown |
| [WP_004298383.1](file:///Volumes/ESD-USB/Summer%202018/Hassan%20Submission%20PLosOne/content.html#lcl|NZ_CP012938.1_prot_WP_004298383.1_2890) |  |  |  |  |  | + |  | [1.A.23.1.4](http://tcdb.org/search/result.php?tc=1.A.33.1.2) | -123 | M4V7W7 | Unknown | Unknown |
| ABI39_10115 | + |  |  |  |  |  |  | 1.A.23.2.1 | -59 | P0C0S1 | Ions | Ions |
| [WP_007209478.1](file:///Volumes/ESD-USB/Summer%202018/Hassan%20Submission%20PLosOne/content.html#lcl|NZ_CP012801.1_prot_WP_007209478.1_3450) |  |  | + |  |  |  |  | [1.A.23.2.1](http://tcdb.org/search/result.php?tc=1.A.23.2.1) | -65 | P0C0S1 | Unknown | Unknown |
| [WP_041584098.1](file:///Volumes/ESD-USB/Summer%202018/Hassan%20Submission%20PLosOne/content.html#lcl|NC_015164.1_prot_WP_041584098.1_3245) |  | + |  |  |  |  |  | [1.A.23.2.1](http://tcdb.org/search/result.php?tc=1.A.23.2.1) | -62 | P0C0S1 | Cations | Cations |
| WP_011108275.1 |  |  |  | + |  |  |  | 1.A.23.2.1 | -65 | P0C0S1 | Unknown | Unknown |
| YP_097899.1 |  |  |  |  | + |  |  | [1.A.23.2.1](http://tcdb.org/search/result.php?tc=1.A.23.2.1) | -63 | P0C0S1 | Ions | Ions |
| [WP_004299133.1](file:///Volumes/ESD-USB/Summer%202018/Hassan%20Submission%20PLosOne/content.html#lcl|NZ_CP012938.1_prot_WP_004299133.1_2270) |  |  |  |  |  | + |  | [1.A.23.2.1](http://tcdb.org/search/result.php?tc=1.A.33.1.2) | -65 | P0C0S1 | Unknown | Unknown |
| [WP_005843474.1](file:///Volumes/ESD-USB/Summer%202018/Library/Application%20Support/Microsoft/Office/Office%202011%20AutoRecovery/content.html#lcl|NC_009614.1_prot_WP_005843474.1_3148) |  |  |  |  |  |  | + | [1.A.23.2.1](http://tcdb.org/search/result.php?tc=1.A.23.2.1) | -61 | P0C0S1 | Unknown | Unknown |
| [WP_011965404.1](file:///Volumes/ESD-USB/Summer%202018/Library/Application%20Support/Microsoft/Office/Office%202011%20AutoRecovery/content.html#lcl|NC_009614.1_prot_WP_011965404.1_1968) |  |  |  |  |  |  | + | [1.A.23.2.1](http://tcdb.org/search/result.php?tc=1.A.23.2.1) | -59 | P0C0S1 | Unknown | Unknown |
| WP_008766794.1 |  |  |  | + |  |  |  | 1.A.23.4.2 | -54 | Q57634 | Unknown | Unknown |
| ABI39_01685 | + |  |  |  |  |  |  | 1.A.23.4.5 | -101 | P0AAT4 | Ions | Ions |
| ABI39_13865 | + |  |  |  |  |  |  | 1.A.23.4.5 | -81 | P0AAT4 | Ions | Ions |
| [WP_007212444.1](file:///Volumes/ESD-USB/Summer%202018/Hassan%20Submission%20PLosOne/content.html#lcl|NZ_CP012801.1_prot_WP_007212444.1_3839) |  |  | + |  |  |  |  | [1.A.23.4.5](http://tcdb.org/search/result.php?tc=1.A.23.4.5) | -114 | P0AAT4 | [Cations](https://www.ebi.ac.uk/chebi/searchId.do;jsessionid=A9D16DCB24C6F74339FC28A4941EFBB6?chebiId=CHEBI:24870) | Cations |
| [WP_007217471.1](file:///Volumes/ESD-USB/Summer%202018/Hassan%20Submission%20PLosOne/content.html#lcl|NZ_CP012801.1_prot_WP_007217471.1_1157) |  |  | + |  |  |  |  | [1.A.23.4.5](http://tcdb.org/search/result.php?tc=1.A.23.4.5) | -76 | P0AAT4 | [Cations](https://www.ebi.ac.uk/chebi/searchId.do;jsessionid=A9D16DCB24C6F74339FC28A4941EFBB6?chebiId=CHEBI:24870) | Cations |
| [WP_013618516.1](file:///Volumes/ESD-USB/Summer%202018/Hassan%20Submission%20PLosOne/content.html#lcl|NC_015164.1_prot_WP_013618516.1_2562) |  | + |  |  |  |  |  | [1.A.23.4.5](http://tcdb.org/search/result.php?tc=1.A.23.4.5) | -100 | P0AAT4 | [Cations](https://www.ebi.ac.uk/chebi/searchId.do;jsessionid=A9D16DCB24C6F74339FC28A4941EFBB6?chebiId=CHEBI:24870) | Ions |
| WP_032814496.1 |  |  |  | + |  |  |  | 1.A.23.4.5 | -119 | P0AAT4 | Cations | Cations |
| WP_008762107.1 |  |  |  | + |  |  |  | 1.A.23.4.5 | -80 | P0AAT4 | Cations | Cations |
| YP_098078.1 |  |  |  |  | + |  |  | [1.A.23.4.5](http://tcdb.org/search/result.php?tc=1.A.23.4.5) | -116 | P0AAT4 | Ions | Ions |
| YP_100520.1 |  |  |  |  | + |  |  | [1.A.23.4.5](http://tcdb.org/search/result.php?tc=1.A.23.4.5) | -76 | P0AAT4 | Ions | Ions |
| [WP_004311434.1](file:///Volumes/ESD-USB/Summer%202018/Hassan%20Submission%20PLosOne/content.html#lcl|NZ_CP012938.1_prot_WP_004311434.1_1625) |  |  |  |  |  | + |  | [1.A.23.4.5](http://tcdb.org/search/result.php?tc=1.A.33.1.2) | -77 | P0AAT4 | [Cations](https://www.ebi.ac.uk/chebi/searchId.do;jsessionid=A9D16DCB24C6F74339FC28A4941EFBB6?chebiId=CHEBI:24870) | [Cations](https://www.ebi.ac.uk/chebi/searchId.do;jsessionid=A9D16DCB24C6F74339FC28A4941EFBB6?chebiId=CHEBI:24870) |
| [WP_005845174.1](file:///Volumes/ESD-USB/Summer%202018/Library/Application%20Support/Microsoft/Office/Office%202011%20AutoRecovery/content.html#lcl|NC_009614.1_prot_WP_005845174.1_314) |  |  |  |  |  |  | + | [1.A.23.4.5](http://tcdb.org/search/result.php?tc=1.A.23.4.5) | -101 | P0AAT4 | [Cations](https://www.ebi.ac.uk/chebi/searchId.do;jsessionid=A9D16DCB24C6F74339FC28A4941EFBB6?chebiId=CHEBI:24870) | Ions |
| [WP_005843823.1](file:///Volumes/ESD-USB/Summer%202018/Library/Application%20Support/Microsoft/Office/Office%202011%20AutoRecovery/content.html#lcl|NC_009614.1_prot_WP_005843823.1_2665) |  |  |  |  |  |  | + | [1.A.23.4.5](http://tcdb.org/search/result.php?tc=1.A.23.4.5) | -82 | P0AAT4 | [Cations](https://www.ebi.ac.uk/chebi/searchId.do;jsessionid=A9D16DCB24C6F74339FC28A4941EFBB6?chebiId=CHEBI:24870) | Ions |
| ABI39_09765 | + |  |  |  |  |  |  | 1.A.26.1.2 | -71 | Q5SMG8 | Cations | Mg^2+^ |
| [WP_007212358.1](file:///Volumes/ESD-USB/Summer%202018/Hassan%20Submission%20PLosOne/content.html#lcl|NZ_CP012801.1_prot_WP_007212358.1_3770) |  |  | + |  |  |  |  | [1.A.26.1.2](http://tcdb.org/search/result.php?tc=1.A.26.1.2) | -71 | Q5SMG8 | Unknown | Unknown |
| [WP_013617629.1](file:///Volumes/ESD-USB/Summer%202018/Hassan%20Submission%20PLosOne/content.html#lcl|NC_015164.1_prot_WP_013617629.1_1597) |  | + |  |  |  |  |  | [1.A.26.1.2](http://tcdb.org/search/result.php?tc=1.A.26.1.2) | -73 | Q5SMG8 | Cations | Mg^2+^ |
| WP_008760927.1 |  |  |  | + |  |  |  | 1.A.26.1.2 | -70 | Q5SMG8 | Cations | Mg^2+^ |
| YP_098062.1 |  |  |  |  | + |  |  | [1.A.26.1.2](http://tcdb.org/search/result.php?tc=1.A.26.1.2) | -71 | Q5SMG8 | Cations | Mg^2+^ |
| [WP_004301409.1](file:///Volumes/ESD-USB/Summer%202018/Hassan%20Submission%20PLosOne/content.html#lcl|NZ_CP012938.1_prot_WP_004301409.1_4122) |  |  |  |  |  | + |  | [1.A.26.1.2](http://tcdb.org/search/result.php?tc=1.A.33.1.2) | -67 | Q5SMG8 | Cations | Mg^2+^ |
| [WP_011965381.1](file:///Volumes/ESD-USB/Summer%202018/Library/Application%20Support/Microsoft/Office/Office%202011%20AutoRecovery/content.html#lcl|NC_009614.1_prot_WP_011965381.1_1897) |  |  |  |  |  |  | + | [1.A.26.1.2](http://tcdb.org/search/result.php?tc=1.A.26.1.2) | -71 | Q5SMG8 | Cations | Mg^2+^ |
| ABI39_11305 | + |  |  |  |  |  |  | 1.A.28.1.4 | -39 | A1VEP3 | Carbamides | Urea |
| [WP_029428984.1](file:///Volumes/ESD-USB/Summer%202018/Hassan%20Submission%20PLosOne/content.html#lcl|NZ_CP012801.1_prot_WP_029428984.1_1887) |  |  | + |  |  |  |  | [1.A.28.1.4](http://tcdb.org/search/result.php?tc=1.A.28.1.4) | -39 | A1VEP3 | Carbamides | Urea |
| ABI39_00325 | + |  |  |  |  |  |  | 1.A.30.1.2 | -06 | Q93R25 | Cations | Na^+^ |
| ABI39_16580 | + |  |  |  |  |  |  | 1.A.30.1.6 | -09 | Q8EAG6 | Cations | Na^+^ |
| [WP_007214888.1](file:///Volumes/ESD-USB/Summer%202018/Hassan%20Submission%20PLosOne/content.html#lcl|NZ_CP012801.1_prot_WP_007214888.1_1310) |  |  | + |  |  |  |  | [1.A.30.1.3](http://tcdb.org/search/result.php?tc=1.A.30.1.3) | -17 | P28612 | Unknown | Unknown |
| [WP_007210072.1](file:///Volumes/ESD-USB/Summer%202018/Hassan%20Submission%20PLosOne/content.html#lcl|NZ_CP012801.1_prot_WP_007210072.1_1682) |  |  | + |  |  |  |  | [1.A.30.2.4](http://tcdb.org/search/result.php?tc=1.A.30.2.4) | -09 | Q1D3D7 | Unknown | Unknown |
| [WP_013617751.1](file:///Volumes/ESD-USB/Summer%202018/Hassan%20Submission%20PLosOne/content.html#lcl|NC_015164.1_prot_WP_013617751.1_1724) |  | + |  |  |  |  |  | [1.A.30.2.4](http://tcdb.org/search/result.php?tc=1.A.30.2.4) | -16 | Q1D3D8 | Anions | H^+^/Na^+^ |
| [WP_013618697.1](file:///Volumes/ESD-USB/Summer%202018/Hassan%20Submission%20PLosOne/content.html#lcl|NC_015164.1_prot_WP_013618697.1_2736) |  | + |  |  |  |  |  | [1.A.30.2.4](http://tcdb.org/search/result.php?tc=1.A.30.2.4) | -07 | Q1D3D7 | Anions | H^+^/Na^+^ |
| WP_008760840.1 |  |  |  | + |  |  |  | 1.A.30.2.4 | -09 | Q1D3D7 | Unknown | Unknown |
| ABI39_09875 | + |  |  |  |  |  |  | 1.A.30.2.7 | -05 | Q1D0D2 | Unknown | Unknown |
| WP_008760582.1 |  |  |  | + |  |  |  | 1.A.30.2.7 | -12 | Q1D0D2 | Unknown | Unknown |
| YP_101241.1 |  |  |  |  | + |  |  | [1.A.30.2.7](http://tcdb.org/search/result.php?tc=1.A.30.2.7) | -08 | Q1D0D2 | Unknown | Unknown |
| [WP_004305499.1](file:///Volumes/ESD-USB/Summer%202018/Hassan%20Submission%20PLosOne/content.html#lcl|NZ_CP012938.1_prot_WP_004305499.1_3867) |  |  |  |  |  | + |  | [1.A.30.2.7](http://tcdb.org/search/result.php?tc=1.A.33.1.2) | -08 | Q1D0D2 | Unknown | Unknown |
| [WP_005845862.1](file:///Volumes/ESD-USB/Summer%202018/Library/Application%20Support/Microsoft/Office/Office%202011%20AutoRecovery/content.html#lcl|NC_009614.1_prot_WP_005845862.1_1918) |  |  |  |  |  |  | + | [1.A.30.2.7](http://tcdb.org/search/result.php?tc=1.A.30.2.7) | -05 | Q1D0D2 | Unknown | Unknown |
| ABI39_02495 | + |  |  |  |  |  |  | 1.A.30.2.8 | -07 | Q8EXJ5 | Cations | H^+^ |
| [WP_008781468.1](file:///Volumes/ESD-USB/Summer%202018/Library/Application%20Support/Microsoft/Office/Office%202011%20AutoRecovery/content.html#lcl|NC_009614.1_prot_WP_008781468.1_469) |  |  |  |  |  |  | + | [1.A.30.2.8](http://tcdb.org/search/result.php?tc=1.A.30.2.8) | -08 | Q8EXJ5 | Unknown | Unknown |
| ABI39_09860 | + |  |  |  |  |  |  | 1.A.30.2.9 | -19 | Q8F192 | Unknown | Unknown |
| ABI39_07990 | + |  |  |  |  |  |  | 1.A.30.2.9 | -05 | Q8F191 | Unknown | Unknown |
| [WP_022391859.1](file:///Volumes/ESD-USB/Summer%202018/Hassan%20Submission%20PLosOne/content.html#lcl|NZ_CP012801.1_prot_WP_022391859.1_1683) |  |  | + |  |  |  |  | [1.A.30.2.9](http://tcdb.org/search/result.php?tc=1.A.30.2.9) | -25 | Q8F192 | Unknown | Unknown |
| [WP_007210397.1](file:///Volumes/ESD-USB/Summer%202018/Hassan%20Submission%20PLosOne/content.html#lcl|NZ_CP012801.1_prot_WP_007210397.1_1454) |  |  | + |  |  |  |  | [1.A.30.2.9](http://tcdb.org/search/result.php?tc=1.A.30.2.9) | -19 | Q8F192 | Unknown | Unknown |
| [WP_007209411.1](file:///Volumes/ESD-USB/Summer%202018/Hassan%20Submission%20PLosOne/content.html#lcl|NZ_CP012801.1_prot_WP_007209411.1_3330) |  |  | + |  |  |  |  | [1.A.30.2.9](http://tcdb.org/search/result.php?tc=1.A.30.2.9) | -05 | Q8F191 | Cations | H^+^ |
| [WP_013618696.1](file:///Volumes/ESD-USB/Summer%202018/Hassan%20Submission%20PLosOne/content.html#lcl|NC_015164.1_prot_WP_013618696.1_2735) |  | + |  |  |  |  |  | [1.A.30.2.9](http://tcdb.org/search/result.php?tc=1.A.30.2.9) | -25 | Q8F192 | Unknown | Unknown |
| [WP_013619227.1](file:///Volumes/ESD-USB/Summer%202018/Hassan%20Submission%20PLosOne/content.html#lcl|NC_015164.1_prot_WP_013619227.1_3283) |  | + |  |  |  |  |  | [1.A.30.2.9](http://tcdb.org/search/result.php?tc=1.A.30.2.9) | -17 | Q8F192 | Unknown | Unknown |
| WP_008760839.1 |  |  |  | + |  |  |  | 1.A.30.2.9 | -23 | Q8F192 | Unknown | Unknown |
| WP_008766172.1 |  |  |  | + |  |  |  | 1.A.30.2.9 | -19 | Q8F192 | Unknown | Unknown |
| WP_008766463.1 |  |  |  | + |  |  |  | 1.A.30.2.9 | -18 | Q8F192 | Unknown | Unknown |
| WP_062694593.1 |  |  |  | + |  |  |  | 1.A.30.2.9 | -06 | Q8F191 | Unknown | Unknown |
| YP_101242.1 |  |  |  |  | + |  |  | [1.A.30.2.9](http://tcdb.org/search/result.php?tc=1.A.30.2.9) | -25 | Q8F192 | Unknown | Unknown |
| YP_101018.1 |  |  |  |  | + |  |  | [1.A.30.2.9](http://tcdb.org/search/result.php?tc=1.A.30.2.9) | -19 | Q8F192 | Unknown | Unknown |
| YP_101409.1 |  |  |  |  | + |  |  | [1.A.30.2.9](http://tcdb.org/search/result.php?tc=1.A.30.2.9) | -06 | Q8F191 | Unknown | Unknown |
| [WP_004297930.1](file:///Volumes/ESD-USB/Summer%202018/Hassan%20Submission%20PLosOne/content.html#lcl|NZ_CP012938.1_prot_WP_004297930.1_1999) |  |  |  |  |  | + |  | [1.A.30.2.9](http://tcdb.org/search/result.php?tc=1.A.33.1.2) | -07 | Q8F191 | Unknown | Unknown |
| [WP_004296287.1](file:///Volumes/ESD-USB/Summer%202018/Hassan%20Submission%20PLosOne/content.html#lcl|NZ_CP012938.1_prot_WP_004296287.1_2596) |  |  |  |  |  | + |  | [1.A.30.2.9](http://tcdb.org/search/result.php?tc=1.A.33.1.2) | -05 | Q8F191 | Unknown | Unknown |
| [WP_008781467.1](file:///Volumes/ESD-USB/Summer%202018/Library/Application%20Support/Microsoft/Office/Office%202011%20AutoRecovery/content.html#lcl|NC_009614.1_prot_WP_008781467.1_470) |  |  |  |  |  |  | + | [1.A.30.2.9](http://tcdb.org/search/result.php?tc=1.A.30.2.9) | -25 | Q8F192 | Unknown | Unknown |
| [WP_005845856.1](file:///Volumes/ESD-USB/Summer%202018/Library/Application%20Support/Microsoft/Office/Office%202011%20AutoRecovery/content.html#lcl|NC_009614.1_prot_WP_005845856.1_1915) |  |  |  |  |  |  | + | [1.A.30.2.9](http://tcdb.org/search/result.php?tc=1.A.30.2.9) | -20 | Q8F192 | Unknown | Unknown |
| [WP_005839160.1](file:///Volumes/ESD-USB/Summer%202018/Library/Application%20Support/Microsoft/Office/Office%202011%20AutoRecovery/content.html#lcl|NC_009614.1_prot_WP_005839160.1_1600) |  |  |  |  |  |  | + | [1.A.30.2.9](http://tcdb.org/search/result.php?tc=1.A.30.2.9) | -05 | Q8F191 | Unknown | Unknown |
| [WP_007667673.1](file:///Volumes/ESD-USB/Summer%202018/Hassan%20Submission%20PLosOne/content.html#lcl|NZ_CP012801.1_prot_WP_007667673.1_3329) |  |  | + |  |  |  |  | [1.A.30.3.2](http://tcdb.org/search/result.php?tc=1.A.30.3.2) | -18 | Q84FB8 | Unknown | Unknown |
| WP_008763543.1 |  |  |  | + |  |  |  | 1.A.30.3.2 | -18 | Q84FB8 | Unknown | Unknown |
| [WP_007215905.1](file:///Volumes/ESD-USB/Summer%202018/Hassan%20Submission%20PLosOne/content.html#lcl|NZ_CP012801.1_prot_WP_007215905.1_4455) |  |  | + |  |  |  |  | [1.A.33.1.2](http://tcdb.org/search/result.php?tc=1.A.33.1.2) | 0 | P0A6Y8 | [Cations](https://www.ebi.ac.uk/chebi/searchId.do;jsessionid=A9D16DCB24C6F74339FC28A4941EFBB6?chebiId=CHEBI:24870) | [Cations](https://www.ebi.ac.uk/chebi/searchId.do;jsessionid=A9D16DCB24C6F74339FC28A4941EFBB6?chebiId=CHEBI:24870) |
| [WP_013617574.1](file:///Volumes/ESD-USB/Summer%202018/Hassan%20Submission%20PLosOne/content.html#lcl|NC_015164.1_prot_WP_013617574.1_1541) |  | + |  |  |  |  |  | [1.A.33.1.2](http://tcdb.org/search/result.php?tc=1.A.33.1.2) | 0 | P0A6Y8 | [Cations](https://www.ebi.ac.uk/chebi/searchId.do;jsessionid=A9D16DCB24C6F74339FC28A4941EFBB6?chebiId=CHEBI:24870) | Ions |
| WP_008764765.1 |  |  |  | + |  |  |  | 1.A.33.1.2 | 0 | P0A6Y8 | Cations | [Cations](https://www.ebi.ac.uk/chebi/searchId.do;jsessionid=A9D16DCB24C6F74339FC28A4941EFBB6?chebiId=CHEBI:24870) |
| YP_098509.1 |  |  |  |  | + |  |  | [1.A.33.1.2](http://tcdb.org/search/result.php?tc=1.A.33.1.2) | 0 | P0A6Y8 | Cations | Ions |
| [WP_004302085.1](file:///Volumes/ESD-USB/Summer%202018/Hassan%20Submission%20PLosOne/content.html#lcl|NZ_CP012938.1_prot_WP_004302085.1_4620) |  |  |  |  |  | + |  | [1.A.33.1.2](http://tcdb.org/search/result.php?tc=1.A.33.1.2) | 0 | P0A6Y8 | [Cations](https://www.ebi.ac.uk/chebi/searchId.do;jsessionid=A9D16DCB24C6F74339FC28A4941EFBB6?chebiId=CHEBI:24870) | Ions |
| [WP_005846379.1](file:///Volumes/ESD-USB/Summer%202018/Library/Application%20Support/Microsoft/Office/Office%202011%20AutoRecovery/content.html#lcl|NC_009614.1_prot_WP_005846379.1_2334) |  |  |  |  |  |  | + | [1.A.33.1.2](http://tcdb.org/search/result.php?tc=1.A.33.1.2) | 0 | P0A6Y8 | [Cations](https://www.ebi.ac.uk/chebi/searchId.do;jsessionid=A9D16DCB24C6F74339FC28A4941EFBB6?chebiId=CHEBI:24870) | Ions |
| [WP_013617692.1](file:///Volumes/ESD-USB/Summer%202018/Hassan%20Submission%20PLosOne/content.html#lcl|NC_015164.1_prot_WP_013617692.1_1661) |  | + |  |  |  |  |  | [1.A.33.1.4](http://tcdb.org/search/result.php?tc=1.A.33.1.4) | -55 | G4NXA5 | [Cations](https://www.ebi.ac.uk/chebi/searchId.do;jsessionid=A9D16DCB24C6F74339FC28A4941EFBB6?chebiId=CHEBI:24870) | Ions |
| YP_097974.1 |  |  |  |  | + |  |  | [1.A.33.1.4](http://tcdb.org/search/result.php?tc=1.A.33.1.4) | -67 | G4NXA5 | Unknown | Unknown |
| ABI39_09195 | + |  |  |  |  |  |  | 1.A.33.1.5 | -90 | Q8F2L2 | Cations | Ions |
| [WP_007209637.1](file:///Volumes/ESD-USB/Summer%202018/Hassan%20Submission%20PLosOne/content.html#lcl|NZ_CP012801.1_prot_WP_007209637.1_3402) |  |  | + |  |  |  |  | [1.A.33.1.5](http://tcdb.org/search/result.php?tc=1.A.33.1.5) | -94 | Q8F2L2 | Unknown | Unknown |
| WP_008762747.1 |  |  |  | + |  |  |  | 1.A.33.1.5 | -92 | Q8F2L2 | Unknown | Unknown |
| YP_101376.1 |  |  |  |  | + |  |  | [1.A.33.1.5](http://tcdb.org/search/result.php?tc=1.A.33.1.5) | -92 | Q8F2L2 | Unknown | Unknown |
| [WP_005842742.1](file:///Volumes/ESD-USB/Summer%202018/Library/Application%20Support/Microsoft/Office/Office%202011%20AutoRecovery/content.html#lcl|NC_009614.1_prot_WP_005842742.1_1800) |  |  |  |  |  |  | + | [1.A.33.1.5](http://tcdb.org/search/result.php?tc=1.A.33.1.5) | -90 | Q8F2L2 | Unknown | Unknown |
| WP_008760846.1 |  |  |  | + |  |  |  | 1.A.34.1.2 | -15 | B7LEG0 | Unknown | Unknown |
| WP_008760060.1 |  |  |  | + |  |  |  | 1.A.34.1.2 | -14 | B7LEG0 | Unknown | Unknown |
| WP_062695432.1 |  |  |  | + |  |  |  | 1.A.34.1.2 | -06 | B7LEG0 | Unknown | Unknown |
| WP_011108845.1 |  |  |  | + |  |  |  | 1.A.34.1.2 | -05 | B7LEG0 | Unknown | Unknown |
| WP_008760911.1 |  |  |  | + |  |  |  | 1.A.34.1.3 | -17 | B7L5V2 | Unknown | Unknown |
| WP_005944242.1 |  |  |  | + |  |  |  | 1.A.34.1.3 | -12 | B7L5V2 | Unknown | Unknown |
| WP_062695420.1 |  |  |  | + |  |  |  | 1.A.34.1.3 | -11 | B7L5V2 | Unknown | Unknown |
| [WP_013617976.1](file:///Volumes/ESD-USB/Summer%202018/Hassan%20Submission%20PLosOne/content.html#lcl|NC_015164.1_prot_WP_013617976.1_1953) |  | + |  |  |  |  |  | [1.A.35.3.1](http://tcdb.org/search/result.php?tc=1.A.35.3.1) | -18 | Q58439 | Cations | Mg^2+/^Ca^2+/^Ni^2+^ |
| [WP_013617428.1](file:///Volumes/ESD-USB/Summer%202018/Hassan%20Submission%20PLosOne/content.html#lcl|NC_015164.1_prot_WP_013617428.1_1394) |  | + |  |  |  |  |  | [1.A.35.3.2](http://tcdb.org/search/result.php?tc=1.A.35.3.2) | -62 | Q9WZ31 | Cations | Co^2+/^Mg^2+^ |
| WP_008766857.1 |  |  |  | + |  |  |  | 1.A.35.3.2 | -69 | Q9WZ31 | Cations | Mg^2+/^Co^2+^ |
| WP_008763680.1 |  |  |  | + |  |  |  | 1.A.35.3.2 | -14 | Q9WZ31 | Cations | Mg^2+/^Co^2+^ |
| [WP_008776058.1](file:///Volumes/ESD-USB/Summer%202018/Hassan%20Submission%20PLosOne/content.html#lcl|NZ_CP012938.1_prot_WP_008776058.1_3397) |  |  |  |  |  | + |  | [1.A.35.3.2](http://tcdb.org/search/result.php?tc=1.A.35.3.2) | -15 | Q9WZ31 | Cations | Mg^2+/^Co^2+^ |
| WP_007215538.1 |  |  | + |  |  |  |  | [1.A.43.1.1](http://tcdb.org/search/result.php?tc=1.A.43.1.1) | -20 | P37002 | [Unknown](https://www.ebi.ac.uk/chebi/searchId.do;jsessionid=A9D16DCB24C6F74339FC28A4941EFBB6?chebiId=CHEBI:17996) | Unknown |
| WP_008764721.1 |  |  |  | + |  |  |  | 1.A.43.1.6 | -25 | Q3B199 | Anions | F^-^ |
| ABI39_19010 | + |  |  |  |  |  |  | 1.A.43.1.13 | -25 | I3YSC9 | Anions | F^-^ |
| [WP_013616800.1](file:///Volumes/ESD-USB/Summer%202018/Hassan%20Submission%20PLosOne/content.html#lcl|NC_015164.1_prot_WP_013616800.1_740) |  | + |  |  |  |  |  | [1.A.43.1.13](http://tcdb.org/search/result.php?tc=1.A.43.1.13) | -24 | I3YSC9 | Cations | F- |
| YP_098474.1 |  |  |  |  | + |  |  | [1.A.43.1.13](http://tcdb.org/search/result.php?tc=1.A.43.1.13) | -20 | I3YSC9 | Anions | F^-^ |
| [WP_004302048.1](file:///Volumes/ESD-USB/Summer%202018/Hassan%20Submission%20PLosOne/content.html#lcl|NZ_CP012938.1_prot_WP_004302048.1_4588) |  |  |  |  |  | + |  | [1.A.43.1.13](http://tcdb.org/search/result.php?tc=1.A.43.1.13) | -17 | I3YSC9 | Anions | F^-^ |
| [WP_005841677.1](file:///Volumes/ESD-USB/Summer%202018/Library/Application%20Support/Microsoft/Office/Office%202011%20AutoRecovery/content.html#lcl|NC_009614.1_prot_WP_005841677.1_3744) |  |  |  |  |  |  | + | [1.A.43.1.13](http://tcdb.org/search/result.php?tc=1.A.43.1.13) | -26 | I3YSC9 | Anions | F^-^ |
| ABI39_17450 | + |  |  |  |  |  |  | 1.A.62.2.1 | -50 | A0M015 | Cations | Cations |
| [WP_013616810.1](file:///Volumes/ESD-USB/Summer%202018/Hassan%20Submission%20PLosOne/content.html#lcl|NC_015164.1_prot_WP_013616810.1_750) |  | + |  |  |  |  |  | [1.A.62.2.1](http://tcdb.org/search/result.php?tc=1.A.62.2.1) | -53 | A0M015 | Cations | Cations |
| [WP_007213626.1](file:///Volumes/ESD-USB/Summer%202018/Hassan%20Submission%20PLosOne/content.html#lcl|NZ_CP012801.1_prot_WP_007213626.1_599) |  |  | + |  |  |  |  | [1.A.62.2.1](http://tcdb.org/search/result.php?tc=1.A.62.2.1) | -54 | A0M015 | Cations | Cations |
| WP_048694308.1 |  |  |  | + |  |  |  | 1.A.62.2.1 | -53 | A0M015 | Cations | Cations |
| YP_099169.1 |  |  |  |  | + |  |  | [1.A.62.2.1](http://tcdb.org/search/result.php?tc=1.A.62.2.1) | -50 | A0M015 | Cations | Cations |
| [WP_004300342.1](file:///Volumes/ESD-USB/Summer%202018/Hassan%20Submission%20PLosOne/content.html#lcl|NZ_CP012938.1_prot_WP_004300342.1_447) |  |  |  |  |  | + |  | [1.A.62.2.1](http://tcdb.org/search/result.php?tc=1.A.62.2.1) | -51 | A0M015 | Cations | [Cations](https://www.ebi.ac.uk/chebi/searchId.do;jsessionid=A9D16DCB24C6F74339FC28A4941EFBB6?chebiId=CHEBI:36916) |
| [WP_005841975.1](file:///Volumes/ESD-USB/Summer%202018/Library/Application%20Support/Microsoft/Office/Office%202011%20AutoRecovery/content.html#lcl|NC_009614.1_prot_WP_005841975.1_3280) |  |  |  |  |  |  | + | [1.A.62.2.1](http://tcdb.org/search/result.php?tc=1.A.62.2.1) | -50 | A0M015 | Cations | Cations |
| ABI39_02780 | + |  |  |  |  |  |  | 1.B.5.1.6 | 0 | E6SSR4 | Molecules | Small molecules |
| ABI39_15015 | + |  |  |  |  |  |  | 1.B.5.1.6 | -44 | E6SSR4 | Molecules | Small molecules |
| [WP_029428122.1](file:///Volumes/ESD-USB/Summer%202018/Hassan%20Submission%20PLosOne/content.html#lcl|NZ_CP012801.1_prot_WP_029428122.1_5150) |  |  | + |  |  |  |  | [1.B.5.1.6](http://tcdb.org/search/result.php?tc=1.B.5.1.6) | -50 | E6SSR4 | Unknown | Unknown |
| [WP_004302334.1](file:///Volumes/ESD-USB/Summer%202018/Hassan%20Submission%20PLosOne/content.html#lcl|NZ_CP012938.1_prot_WP_004302334.1_4798) |  |  |  |  |  | + |  | [1.B.5.1.6](http://tcdb.org/search/result.php?tc=1.B.5.1.6) | -46 | E6SSR4 | Unknown | Unknown |
| [WP_011964787.1](file:///Volumes/ESD-USB/Summer%202018/Library/Application%20Support/Microsoft/Office/Office%202011%20AutoRecovery/content.html#lcl|NC_009614.1_prot_WP_011964787.1_524) |  |  |  |  |  |  | + | [1.B.5.1.6](http://tcdb.org/search/result.php?tc=1.B.5.1.6) | 0 | E6SSR4 | Unknown | Unknown |
| [WP_032856151.1](file:///Volumes/ESD-USB/Summer%202018/Hassan%20Submission%20PLosOne/content.html#lcl|NZ_CP012938.1_prot_WP_032856151.1_1678) |  |  |  |  |  | + |  | [1.B.5.1.12](http://tcdb.org/search/result.php?tc=1.B.5.1.12) | -05 | Q2S5G8 | Unknown | Unknown |
| WP_008760779.1 |  |  |  | + |  |  |  | 1.B.6.1.6 | -39 | Q9S3R8 | Sugars | Polysaccharides |
| WP_062694809.1 |  |  |  | + |  |  |  | 1.B.6.1.6 | -37 | Q9S3R8 | Sugars | Polysaccharides |
| WP_062695910.1 |  |  |  | + |  |  |  | 1.B.6.1.6 | -36 | Q9S3R8 | Sugars | Polysaccharides |
| WP_062694813.1 |  |  |  | + |  |  |  | 1.B.6.1.6 | -36 | Q9S3R8 | Sugars | Polysaccharides |
| WP_008760698.1 |  |  |  | + |  |  |  | 1.B.6.1.6 | -36 | Q9S3R8 | Sugars | Polysaccharides |
| WP_008764794.1 |  |  |  | + |  |  |  | 1.B.6.1.6 | -35 | Q9S3R8 | Sugars | Polysaccharides |
| WP_008767726.1 |  |  |  | + |  |  |  | 1.B.6.1.6 | -34 | Q9S3R8 | Sugars | Polysaccharides |
| WP_062695530.1 |  |  |  | + |  |  |  | 1.B.6.1.6 | -34 | Q9S3R8 | Sugars | Polysaccharides |
| WP_008765146.1 |  |  |  | + |  |  |  | 1.B.6.1.6 | -31 | Q9S3R8 | Sugars | Polysaccharides |
| WP_008763632.1 |  |  |  | + |  |  |  | 1.B.6.1.6 | -29 | Q9S3R8 | Sugars | Polysaccharides |
| WP_011107749.1 |  |  |  | + |  |  |  | 1.B.6.1.6 | -16 | Q9S3R8 | Sugars | Polysaccharides |
| WP_004309918.1 |  |  |  | + |  |  |  | 1.B.6.1.6 | -12 | Q9S3R8 | Sugars | Polysaccharides |
| WP_062695446.1 |  |  |  | + |  |  |  | 1.B.6.1.6 | -11 | Q9S3R8 | Sugars | Polysaccharides |
| WP_062695668.1 |  |  |  | + |  |  |  | 1.B.6.1.6 | -09 | Q9S3R8 | Sugars | Polysaccharides |
| YP_098582.1 |  |  |  |  | + |  |  | 1.B.6.1.6 | -52 | Q9S3R8 | Sugars | Polysaccharides |
| YP_101313.1 |  |  |  |  | **+** |  |  | 1.B.6.1.6 | -38 | Q9S3R8 | Sugars | Polysaccharides |
| YP_098955.1 |  |  |  |  | **+** |  |  | 1.B.6.1.6 | -36 | Q9S3R8 | Sugars | Polysaccharides |
| YP_098963.1 |  |  |  |  | **+** |  |  | 1.B.6.1.6 | -33 | Q9S3R8 | Sugars | Polysaccharides |
| [WP_004297176.1](file:///Volumes/ESD-USB/Summer%202018/Hassan%20Submission%20PLosOne/content.html#lcl|NZ_CP012938.1_prot_WP_004297176.1_3809) |  |  |  |  |  | + |  | [1.B.6.1.6](http://tcdb.org/search/result.php?tc=1.B.6.1.6) | -43 | Q9S3R8 | Unknown | Unknown |
| [WP_004297527.1](file:///Volumes/ESD-USB/Summer%202018/Hassan%20Submission%20PLosOne/content.html#lcl|NZ_CP012938.1_prot_WP_004297527.1_259) |  |  |  |  |  | + |  | [1.B.6.1.6](http://tcdb.org/search/result.php?tc=1.B.6.1.6) | -37 | Q9S3R8 | Unknown | Unknown |
| [WP_004296157.1](file:///Volumes/ESD-USB/Summer%202018/Hassan%20Submission%20PLosOne/content.html#lcl|NZ_CP012938.1_prot_WP_004296157.1_2487) |  |  |  |  |  | + |  | [1.B.6.1.6](http://tcdb.org/search/result.php?tc=1.B.6.1.6) | -35 | Q9S3R8 | Unknown | Unknown |
| [WP_004301238.1](file:///Volumes/ESD-USB/Summer%202018/Hassan%20Submission%20PLosOne/content.html#lcl|NZ_CP012938.1_prot_WP_004301238.1_4007) |  |  |  |  |  | + |  | [1.B.6.1.6](http://tcdb.org/search/result.php?tc=1.B.6.1.6) | -10 | Q9S3R8 | Unknown | Unknown |
| [WP_004302328.1](file:///Volumes/ESD-USB/Summer%202018/Hassan%20Submission%20PLosOne/content.html#lcl|NZ_CP012938.1_prot_WP_004302328.1_4796) |  |  |  |  |  | + |  | [1.B.6.1.6](http://tcdb.org/search/result.php?tc=1.B.6.1.6) | -06 | Q9S3R8 | Unknown | Unknown |
| ABI39_10660 | + |  |  |  |  |  |  | 1.B.6.1.14 | -13 | K1LZ89 | Molecules | Small molecules |
| ABI39_18325 | + |  |  |  |  |  |  | 1.B.6.1.14 | -06 | K1LZ89 | Molecules | Small molecules |
| [WP_029427708.1](file:///Volumes/ESD-USB/Summer%202018/Hassan%20Submission%20PLosOne/content.html#lcl|NZ_CP012801.1_prot_WP_029427708.1_4410) |  |  | + |  |  |  |  | [1.B.6.1.14](http://tcdb.org/search/result.php?tc=1.B.6.1.14) | -08 | K1LZ89 | Unknown | Unknown |
| [WP_013616229.1](file:///Volumes/ESD-USB/Summer%202018/Hassan%20Submission%20PLosOne/content.html#lcl|NC_015164.1_prot_WP_013616229.1_151) |  | + |  |  |  |  |  | [1.B.6.1.14](http://tcdb.org/search/result.php?tc=1.B.6.1.14) | -19 | K1LZ89 | Unknown | Unknown |
| YP_099201.1 |  |  |  |  | + |  |  | 1.B.6.1.14 | -15 | K1LZ89 | Molecules | Small molecules |
| YP_101044.1 |  |  |  |  | + |  |  | 1.B.6.1.14 | -09 | K1LZ89 | Molecules | Small molecules |
| ABI39_08355 | + |  |  |  |  |  |  | 1.B.6.6.7 | -15 | O30487 | Molecules | Small molecules |
| YP_099819.1 |  |  |  |  | + |  |  | 1.B.6.6.7 | -10 | O30487 | Molecules | Small molecules |
| [WP_004295700.1](file:///Volumes/ESD-USB/Summer%202018/Hassan%20Submission%20PLosOne/content.html#lcl|NZ_CP012938.1_prot_WP_004295700.1_948) |  |  |  |  |  | + |  | [1.B.6.6.7](http://tcdb.org/search/result.php?tc=1.B.6.6.7) | -05 | O30487 | Unknown | Unknown |
| [WP_008667901.1](file:///Volumes/ESD-USB/Summer%202018/Library/Application%20Support/Microsoft/Office/Office%202011%20AutoRecovery/content.html#lcl|NC_009614.1_prot_WP_008667901.1_1673) |  |  |  |  |  |  | + | [1.B.6.6.7](http://tcdb.org/search/result.php?tc=1.B.6.6.7) | -17 | O30487 | Unknown | Unknown |
| WP_029427700.1 |  |  | + |  |  |  |  | [1.B.6.7.1](http://tcdb.org/search/result.php?tc=1.B.6.7.1) | -148 | Q64QZ2 | Unknown | Unknown |
| [WP_029426031.1](file:///Volumes/ESD-USB/Summer%202018/Hassan%20Submission%20PLosOne/content.html#lcl|NZ_CP012801.1_prot_WP_029426031.1_2137) |  |  | + |  |  |  |  | [1.B.6.7.1](http://tcdb.org/search/result.php?tc=1.B.6.7.1) | -10 | Q64QZ2 | Unknown | Unknown |
| WP_048693721.1 |  |  |  | + |  |  |  | 1.B.6.7.1 | -08 | Q64QZ2 | Unknown | Unknown |
| YP_098478.1 |  |  |  |  | + |  |  | 1.B.6.7.1 | 0 | Q64QZ2 | Molecules | Small molecules |
| YP_100623.1 |  |  |  |  | + |  |  | 1.B.6.7.1 | 0 | Q64QZ2 | Molecules | Small molecules |
| [WP_004321670.1](file:///Volumes/ESD-USB/Summer%202018/Hassan%20Submission%20PLosOne/content.html#lcl|NZ_CP012938.1_prot_WP_004321670.1_1860) |  |  |  |  |  | + |  | [1.B.6.7.1](http://tcdb.org/search/result.php?tc=1.B.6.7.1) | -09 | Q64QZ2 | Unknown | Unknown |
| ABI39_11365 | + |  |  |  |  |  |  | 1.B.6.9.3 | -07 | G8R543 | Molecules | Small molecules |
| ABI39_12900 | + |  |  |  |  |  |  | 1.B.6.9.3 | -06 | G8R543 | Molecules | Small molecules |
| WP_008763636.1 |  |  |  | + |  |  |  | 1.B.6.9.3 | -07 | G8R543 | Sugars | Polysaccharides |
| WP_008760037.1 |  |  |  | + |  |  |  | 1.B.9.2.3 | -05 | Q9RBW8 | Unknown | Unknown |
| ABI39_10840 | + |  |  |  |  |  |  | 1.B.9.2.4 | -13 | F2NY23 | Hydrocarbons | Aromatic hydrocarbons |
| WP_007213366.1 |  |  | + |  |  |  |  | [1.B.9.2.4](http://tcdb.org/search/result.php?tc=1.B.9.2.4) | -17 | F2NY23 | Unknown | Unknown |
| WP_008767945.1 |  |  |  | + |  |  |  | 1.B.9.2.4 | -06 | F2NY23 | Unknown | Unknown |
| [WP_004300771.1](file:///Volumes/ESD-USB/Summer%202018/Hassan%20Submission%20PLosOne/content.html#lcl|NZ_CP012938.1_prot_WP_004300771.1_1589) |  |  |  |  |  | + |  | [1.B.9.2.4](http://tcdb.org/search/result.php?tc=1.B.9.2.4) | -15 | F2NY23 | Unknown | Unknown |
| ABI39_11540 | + |  |  |  |  |  |  | 1.B.9.4.2 | -105 | E7RSN2 | Unknown | Unknown |
| WP_029428488.1 |  |  | + |  |  |  |  | [1.B.9.4.2](http://tcdb.org/search/result.php?tc=1.B.9.4.2) | -89 | E7RSN2 | Unknown | Unknown |
| YP_098915.1 |  |  |  |  | + |  |  | 1.B.9.4.2 | -92 | E7RSN2 | Unknown | Unknown |
| WP_005846158.1 |  |  |  |  |  |  | + | [1.B.9.4.2](http://tcdb.org/search/result.php?tc=1.B.9.4.2) | -105 | E7RSN2 | Unknown | Unknown |
| WP_022471012.1 |  |  |  | + |  |  |  | 1.B.13.3.1 | -10 | B9XJ69 | Sugar alcohols | Alginate |
| YP_101571.1 |  |  |  |  | + |  |  | 1.B.14.1.4 | -18 | P17315 | Cations | Fe^3+^ |
| YP_100244.1 |  |  |  |  | + |  |  | 1.B.14.1.4 | -13 | P17315 | Cations | Fe^3+^ |
| YP_099795.1 |  |  |  |  | + |  |  | 1.B.14.1.7 | -32 | A4LBD1 | Cations | Fe^3+^ |
| WP_032840677.1 |  |  |  | + |  |  |  | 1.B.14.3.1 | -12 | P06129 | Vitamins | Cobalamin |
| WP_062694585.1 |  |  |  | + |  |  |  | 1.B.14.3.1 | -05 | P06129 | Vitamins | Cobalamin |
| [WP_004319328.1](file:///Volumes/ESD-USB/Summer%202018/Hassan%20Submission%20PLosOne/content.html#lcl|NZ_CP012938.1_prot_WP_004319328.1_157) |  |  |  |  |  | + |  | [1.B.9.4.2](http://tcdb.org/search/result.php?tc=1.B.9.4.2) | -74 | E7RSN2 | Unknown | Unknown |
| [WP_004318258.1](file:///Volumes/ESD-USB/Summer%202018/Hassan%20Submission%20PLosOne/content.html#lcl|NZ_CP012938.1_prot_WP_004318258.1_681) |  |  |  |  |  | + |  | [1.B.14.1.13](http://tcdb.org/search/result.php?tc=1.B.14.1.13) | -19 | A3ZKG8 | Unknown | Unknown |
| YP_099205.1 |  |  |  |  | + |  |  | 1.B.14.1.20 | -22 | P13036 | Cations | Fe^3+^ |
| [WP_052588034.1](file:///Volumes/ESD-USB/Summer%202018/Hassan%20Submission%20PLosOne/content.html#lcl|NZ_CP012938.1_prot_WP_052588034.1_4015) |  |  |  |  |  | + |  | [1.B.14.1.25](http://tcdb.org/search/result.php?tc=1.B.14.1.25) | -07 | Q7VJ32 | Unknown | Unknown |
| YP_097601.1 |  |  |  |  | + |  |  | 1.B.14.2.9 | -22 | Q9JZN9 | Unknown | Unknown |
| YP_100711.1 |  |  |  |  | + |  |  | 1.B.14.2.9 | -09 | Q9JZN9 | Unknown | Unknown |
| [WP_004297095.1](file:///Volumes/ESD-USB/Summer%202018/Hassan%20Submission%20PLosOne/content.html#lcl|NZ_CP012938.1_prot_WP_004297095.1_1396) |  |  |  |  |  | + |  | [1.B.14.2.9](http://tcdb.org/search/result.php?tc=1.B.14.2.9) | -13 | Q9JZN9 | Unknown | Unknown |
| ABI39_10465 | + |  |  |  |  |  |  | 1.B.14.3.1 | -09 | P06129 | Vitamins | Cobalamin |
| YP_098853.1 |  |  |  |  | + |  |  | 1.B.14.3.1 | -08 | P06129 | Vitamins | Cobalamin |
| [WP_004300635.1](file:///Volumes/ESD-USB/Summer%202018/Hassan%20Submission%20PLosOne/content.html#lcl|NZ_CP012938.1_prot_WP_004300635.1_1507) |  |  |  |  |  | + |  | [1.B.14.3.1](http://tcdb.org/search/result.php?tc=1.B.14.3.1) | -06 | P06129 | Unknown | Unknown |
| WP_008765027.1 |  |  |  | + |  |  |  | 1.B.14.3.7 | -29 | Q0HZ17 | Unknown | Unknown |
| WP_008765025.1 |  |  |  | + |  |  |  | 1.B.14.3.7 | -27 | Q0HZ17 | Unknown | Unknown |
| WP_062695124.1 |  |  |  | + |  |  |  | 1.B.14.3.7 | -14 | Q0HZ17 | Unknown | Unknown |
| YP_099959.1 |  |  |  |  | + |  |  | 1.B.14.3.7 | -23 | Q0HZ17 | Molecules | Small molecules |
| YP_099092.1 |  |  |  |  | + |  |  | 1.B.14.3.7 | -29 | Q0HZ17 | Molecules | Small molecules |
| WP_062695881.1 |  |  |  | + |  |  |  | 1.B.14.4.3 | -05 | 660511454 | Unknown | Unknown |
| [ABI39_04455](file:///Volumes/ESD-USB/Summer%202018/Hassan%20Submission%20PLosOne/content.html#lcl|CP011531.1_prot_ABI39_04455_832) | + |  |  |  |  |  |  | [1.B.14.6.1](http://tcdb.org/search/result.php?tc=1.B.14.6.1) | 0 | Q45780 | Sugars | Maltooligosaccharides |
| [ABI39_03920](file:///Volumes/ESD-USB/Summer%202018/Hassan%20Submission%20PLosOne/content.html#lcl|CP011531.1_prot_ABI39_03920_732) | + |  |  |  |  |  |  | [1.B.14.6.1](http://tcdb.org/search/result.php?tc=1.B.14.6.1) | -81 | Q45780 | Sugars | Maltooligosaccharides |
| [ABI39_07675](file:///Volumes/ESD-USB/Summer%202018/Hassan%20Submission%20PLosOne/content.html#lcl|CP011531.1_prot_ABI39_07675_1446) | + |  |  |  |  |  |  | [1.B.14.6.1](http://tcdb.org/search/result.php?tc=1.B.14.6.1) | -57 | Q45780 | Sugars | Maltooligosaccharides |
| [ABI39_08910](file:///Volumes/ESD-USB/Summer%202018/Hassan%20Submission%20PLosOne/content.html#lcl|CP011531.1_prot_ABI39_08910_1668) | + |  |  |  |  |  |  | [1.B.14.6.1](http://tcdb.org/search/result.php?tc=1.B.14.6.1) | -53 | Q45780 | Sugars | Maltooligosaccharides |
| [ABI39_16605](file:///Volumes/ESD-USB/Summer%202018/Hassan%20Submission%20PLosOne/content.html#lcl|CP011531.1_prot_ABI39_16605_3141) | + |  |  |  |  |  |  | [1.B.14.6.1](http://tcdb.org/search/result.php?tc=1.B.14.6.1) | -43 | Q45780 | Sugars | Maltooligosaccharides |
| [WP_013616237.1](file:///Volumes/ESD-USB/Summer%202018/Hassan%20Submission%20PLosOne/content.html#lcl|NC_015164.1_prot_WP_013616237.1_159) |  | + |  |  |  |  |  | [1.B.14.6.1](http://tcdb.org/search/result.php?tc=1.B.14.6.1) | 0 | Q45780 | Sugars | Maltooligosaccharides |
| [WP_041584099.1](file:///Volumes/ESD-USB/Summer%202018/Hassan%20Submission%20PLosOne/content.html#lcl|NC_015164.1_prot_WP_041584099.1_3274) |  | + |  |  |  |  |  | [1.B.14.6.1](http://tcdb.org/search/result.php?tc=1.B.14.6.1) | -32 | Q45780 | Sugars | Maltooligosaccharides |
| [WP_013619186.1](file:///Volumes/ESD-USB/Summer%202018/Hassan%20Submission%20PLosOne/content.html#lcl|NC_015164.1_prot_WP_013619186.1_3239) |  | + |  |  |  |  |  | [1.B.14.6.1](http://tcdb.org/search/result.php?tc=1.B.14.6.1) | -06 | Q45780 | Sugars | Maltooligosaccharides |
| [WP_029426066.1](file:///Volumes/ESD-USB/Summer%202018/Hassan%20Submission%20PLosOne/content.html#lcl|NZ_CP012801.1_prot_WP_029426066.1_2200) |  |  | + |  |  |  |  | [1.B.14.6.1](http://tcdb.org/search/result.php?tc=1.B.14.6.1) | 0 | Q45780 | Sugars | Maltooligosaccharides |
| [WP_029428320.1](file:///Volumes/ESD-USB/Summer%202018/Hassan%20Submission%20PLosOne/content.html#lcl|NZ_CP012801.1_prot_WP_029428320.1_1224) |  |  | + |  |  |  |  | [1.B.14.6.1](http://tcdb.org/search/result.php?tc=1.B.14.6.1) | 0 | Q45780 | Sugars | Maltooligosaccharides |
| [WP_029427749.1](file:///Volumes/ESD-USB/Summer%202018/Hassan%20Submission%20PLosOne/content.html#lcl|NZ_CP012801.1_prot_WP_029427749.1_1773) |  |  | + |  |  |  |  | [1.B.14.6.1](http://tcdb.org/search/result.php?tc=1.B.14.6.1) | 0 | Q45780 | Sugars | Maltooligosaccharides |
| [WP_022209205.1](file:///Volumes/ESD-USB/Summer%202018/Hassan%20Submission%20PLosOne/content.html#lcl|NZ_CP012801.1_prot_WP_022209205.1_1739) |  |  | + |  |  |  |  | [1.B.14.6.1](http://tcdb.org/search/result.php?tc=1.B.14.6.1) | -59 | Q45780 | Sugars | Maltooligosaccharides |
| [WP_026367260.](file:///Volumes/ESD-USB/Summer%202018/Hassan%20Submission%20PLosOne/content.html#lcl|NZ_CP012801.1_prot_WP_026367260.1_3321) 1 |  |  | + |  |  |  |  | [1.B.14.6.1](http://tcdb.org/search/result.php?tc=1.B.14.6.1) | -50 | Q45780 | Sugars | Maltooligosaccharides |
| [WP_029428513.1](file:///Volumes/ESD-USB/Summer%202018/Hassan%20Submission%20PLosOne/content.html#lcl|NZ_CP012801.1_prot_WP_029428513.1_853) |  |  | + |  |  |  |  | [1.B.14.6.1](http://tcdb.org/search/result.php?tc=1.B.14.6.1) | -47 | Q45780 | Sugars | Maltooligosaccharides |
| WP_008767004.1 |  |  |  | + |  |  |  | [1.B.14.6.1](http://tcdb.org/search/result.php?tc=1.B.14.6.1) | 0 | Q45780 | Sugars | Maltooligosaccharides |
| WP_062694220.1 |  |  |  | + |  |  |  | [1.B.14.6.1](http://tcdb.org/search/result.php?tc=1.B.14.6.1) | -53 | Q45780 | Sugars | Maltooligosaccharides |
| WP_022471962.1 |  |  |  | + |  |  |  | [1.B.14.6.1](http://tcdb.org/search/result.php?tc=1.B.14.6.1) | -46 | Q45780 | Sugars | Maltooligosaccharides |
| WP_008767691.1 |  |  |  | + |  |  |  | [1.B.14.6.1](http://tcdb.org/search/result.php?tc=1.B.14.6.1) | -43 | Q45780 | Sugars | Maltooligosaccharides |
| WP_004327093.1 |  |  |  | + |  |  |  | [1.B.14.6.1](http://tcdb.org/search/result.php?tc=1.B.14.6.1) | -34 | Q45780 | Sugars | Maltooligosaccharides |
| WP_008764618.1 |  |  |  | + |  |  |  | [1.B.14.6.1](http://tcdb.org/search/result.php?tc=1.B.14.6.1) | -18 | Q45780 | Sugars | Maltooligosaccharides |
| WP_062695586.1 |  |  |  | + |  |  |  | [1.B.14.6.1](http://tcdb.org/search/result.php?tc=1.B.14.6.1) | -06 | Q45780 | Sugars | Maltooligosaccharides |
| [YP_100585.1](file:///Volumes/ESD-USB/Summer%202018/Library/Application%20Support/Microsoft/Office/Office%202011%20AutoRecovery/content.html#lcl|NC_006347.1_prot_YP_100585.1_3302) |  |  |  |  | + |  |  | [1.B.14.6.1](http://tcdb.org/search/result.php?tc=1.B.14.6.1) | 0 | Q45780 | Sugars | Maltooligosaccharides |
| [YP_098994.1](file:///Volumes/ESD-USB/Summer%202018/Library/Application%20Support/Microsoft/Office/Office%202011%20AutoRecovery/content.html#lcl|NC_006347.1_prot_YP_098994.1_1711) |  |  |  |  | + |  |  | [1.B.14.6.1](http://tcdb.org/search/result.php?tc=1.B.14.6.1) | -57 | Q45780 | Sugars | Maltooligosaccharides |
| YP_098993.1 |  |  |  |  | + |  |  | [1.B.14.6.1](http://tcdb.org/search/result.php?tc=1.B.14.6.1) | -14 | Q45780 | Sugars | Maltooligosaccharides |
| YP_098184.1 |  |  |  |  | + |  |  | [1.B.14.6.1](http://tcdb.org/search/result.php?tc=1.B.14.6.1) | -07 | Q45780 | Sugars | Maltooligosaccharides |
| WP_004300556.1 |  |  |  |  |  | + |  | [1.B.14.6.1](http://tcdb.org/search/result.php?tc=1.B.14.6.1) | -56 | Q45780 | Sugars | Maltooligosaccharides |
| [WP_004302350.1](file:///Volumes/ESD-USB/Summer%202018/Hassan%20Submission%20PLosOne/content.html#lcl|NZ_CP012938.1_prot_WP_004302350.1_4804) |  |  |  |  |  | + |  | [1.B.14.6.1](http://tcdb.org/search/result.php?tc=1.B.14.6.1) | -43 | Q45780 | Sugars | Maltooligosaccharides |
| [WP_004301946.1](file:///Volumes/ESD-USB/Summer%202018/Hassan%20Submission%20PLosOne/content.html#lcl|NZ_CP012938.1_prot_WP_004301946.1_4501) |  |  |  |  |  | + |  | [1.B.14.6.1](http://tcdb.org/search/result.php?tc=1.B.14.6.1) | -15 | Q45780 | Sugars | Maltooligosaccharides |
| [WP_011965160.1](file:///Volumes/ESD-USB/Summer%202018/Library/Application%20Support/Microsoft/Office/Office%202011%20AutoRecovery/content.html#lcl|NC_009614.1_prot_WP_011965160.1_1363) |  |  |  |  |  |  | + | [1.B.14.6.1](http://tcdb.org/search/result.php?tc=1.B.14.6.1) | 0 | Q45780 | Sugars | Maltooligosaccharides |
| [WP_011964600.1](file:///Volumes/ESD-USB/Summer%202018/Library/Application%20Support/Microsoft/Office/Office%202011%20AutoRecovery/content.html#lcl|NC_009614.1_prot_WP_011964600.1_40) |  |  |  |  |  |  | + | [1.B.14.6.1](http://tcdb.org/search/result.php?tc=1.B.14.6.1) | -76 | Q45780 | Sugars | Maltooligosaccharides |
| [WP_011965651.1](file:///Volumes/ESD-USB/Summer%202018/Library/Application%20Support/Microsoft/Office/Office%202011%20AutoRecovery/content.html#lcl|NC_009614.1_prot_WP_011965651.1_2530) |  |  |  |  |  |  | + | [1.B.14.6.1](http://tcdb.org/search/result.php?tc=1.B.14.6.1) | -56 | Q45780 | Sugars | Maltooligosaccharides |
| [WP_005843090.1](file:///Volumes/ESD-USB/Summer%202018/Library/Application%20Support/Microsoft/Office/Office%202011%20AutoRecovery/content.html#lcl|NC_009614.1_prot_WP_005843090.1_128) |  |  |  |  |  |  | + | [1.B.14.6.1](http://tcdb.org/search/result.php?tc=1.B.14.6.1) | -54 | Q45780 | Sugars | Maltooligosaccharides |
| [WP_011965363.1](file:///Volumes/ESD-USB/Summer%202018/Library/Application%20Support/Microsoft/Office/Office%202011%20AutoRecovery/content.html#lcl|NC_009614.1_prot_WP_011965363.1_1825) |  |  |  |  |  |  | + | [1.B.14.6.1](http://tcdb.org/search/result.php?tc=1.B.14.6.1) | -51 | Q45780 | Sugars | Maltooligosaccharides |
| ABI39_02955 | + |  |  |  |  |  |  | 1.B.14.6.2 | 0 | Q93TH9 | Sugars | Maltooligosaccharides |
| ABI39_02945 | + |  |  |  |  |  |  | 1.B.14.6.2 | -146 | Q93TH9 | Sugars | Maltooligosaccharides |
| ABI39_03305 | + |  |  |  |  |  |  | 1.B.14.6.2 | -138 | Q93TH9 | Sugars | Maltooligosaccharides |
| ABI39_21175 | + |  |  |  |  |  |  | 1.B.14.6.2 | -67 | Q93TH9 | Unknown | Unknown |
| ABI39_02665 | + |  |  |  |  |  |  | 1.B.14.6.2 | -54 | Q93TH9 | Unknown | Unknown |
| ABI39_10260 | + |  |  |  |  |  |  | 1.B.14.6.2 | -48 | Q93TH9 | Unknown | Unknown |
| ABI39_05195 | + |  |  |  |  |  |  | 1.B.14.6.3 | -14 | P38370 | Unknown | Unknown |
| [WP_013617900.1](file:///Volumes/ESD-USB/Summer%202018/Hassan%20Submission%20PLosOne/content.html#lcl|NC_015164.1_prot_WP_013617900.1_1875) |  | + |  |  |  |  |  | [1.B.14.6.2](http://tcdb.org/search/result.php?tc=1.B.14.6.2) | -127 | Q93TH9 | Unknown | Unknown |
| [WP_013617590.1](file:///Volumes/ESD-USB/Summer%202018/Hassan%20Submission%20PLosOne/content.html#lcl|NC_015164.1_prot_WP_013617590.1_1561) |  | + |  |  |  |  |  | [1.B.14.6.2](http://tcdb.org/search/result.php?tc=1.B.14.6.2) | -63 | Q93TH9 | Unknown | Unknown |
| [WP_013617633.1](file:///Volumes/ESD-USB/Summer%202018/Hassan%20Submission%20PLosOne/content.html#lcl|NC_015164.1_prot_WP_013617633.1_1601) |  | + |  |  |  |  |  | [1.B.14.6.2](http://tcdb.org/search/result.php?tc=1.B.14.6.2) | -06 | Q93TH9 | Unknown | Unknown |
| [WP_013618482.1](file:///Volumes/ESD-USB/Summer%202018/Hassan%20Submission%20PLosOne/content.html#lcl|NC_015164.1_prot_WP_013618482.1_2478) |  | + |  |  |  |  |  | [1.B.14.6.2](http://tcdb.org/search/result.php?tc=1.B.14.6.2) | 0.000124022 | Q93TH9 | Unknown | Unknown |
| [WP_033160454.1](file:///Volumes/ESD-USB/Summer%202018/Hassan%20Submission%20PLosOne/content.html#lcl|NZ_CP012801.1_prot_WP_033160454.1_3309) |  |  | + |  |  |  |  | [1.B.14.6.2](http://tcdb.org/search/result.php?tc=1.B.14.6.2) | -142 | Q93TH9 | Unknown | Unknown |
| [WP_052331990.1](file:///Volumes/ESD-USB/Summer%202018/Hassan%20Submission%20PLosOne/content.html#lcl|NZ_CP012801.1_prot_WP_052331990.1_2109) |  |  | + |  |  |  |  | [1.B.14.6.2](http://tcdb.org/search/result.php?tc=1.B.14.6.2) | -125 | Q93TH9 | Unknown | Unknown |
| [WP_029427549.1](file:///Volumes/ESD-USB/Summer%202018/Hassan%20Submission%20PLosOne/content.html#lcl|NZ_CP012801.1_prot_WP_029427549.1_3345) |  |  | + |  |  |  |  | [1.B.14.6.2](http://tcdb.org/search/result.php?tc=1.B.14.6.2) | -87 | Q93TH9 | Unknown | Unknown |
| [WP_029426083.1](file:///Volumes/ESD-USB/Summer%202018/Hassan%20Submission%20PLosOne/content.html#lcl|NZ_CP012801.1_prot_WP_029426083.1_2228) |  |  | + |  |  |  |  | [1.B.14.6.2](http://tcdb.org/search/result.php?tc=1.B.14.6.2) | -76 | Q93TH9 | Unknown | Unknown |
| [WP_007210164.1](file:///Volumes/ESD-USB/Summer%202018/Hassan%20Submission%20PLosOne/content.html#lcl|NZ_CP012801.1_prot_WP_007210164.1_1617) |  |  | + |  |  |  |  | [1.B.14.6.2](http://tcdb.org/search/result.php?tc=1.B.14.6.2) | -73 | Q93TH9 | Unknown | Unknown |
| [WP_029428422.1](file:///Volumes/ESD-USB/Summer%202018/Hassan%20Submission%20PLosOne/content.html#lcl|NZ_CP012801.1_prot_WP_029428422.1_1046) |  |  | + |  |  |  |  | [1.B.14.6.2](http://tcdb.org/search/result.php?tc=1.B.14.6.2) | -67 | Q93TH9 | Unknown | Unknown |
| [WP_029426057.1](file:///Volumes/ESD-USB/Summer%202018/Hassan%20Submission%20PLosOne/content.html#lcl|NZ_CP012801.1_prot_WP_029426057.1_2188) |  |  | + |  |  |  |  | [1.B.14.6.2](http://tcdb.org/search/result.php?tc=1.B.14.6.2) | -57 | Q93TH9 | Unknown | Unknown |
| WP_029428853.1 |  |  | + |  |  |  |  | [1.B.14.6.2](http://tcdb.org/search/result.php?tc=1.B.14.6.2) | -52 | Q93TH9 | Unknown | Unknown |
| [WP_029427313.1](file:///Volumes/ESD-USB/Summer%202018/Hassan%20Submission%20PLosOne/content.html#lcl|NZ_CP012801.1_prot_WP_029427313.1_3636) |  |  | + |  |  |  |  | [1.B.14.6.2](http://tcdb.org/search/result.php?tc=1.B.14.6.2) | -38 | Q93TH9 | Unknown | Unknown |
| [WP_033160486.1](file:///Volumes/ESD-USB/Summer%202018/Hassan%20Submission%20PLosOne/content.html#lcl|NZ_CP012801.1_prot_WP_033160486.1_332) |  |  | + |  |  |  |  | [1.B.14.6.2](http://tcdb.org/search/result.php?tc=1.B.14.6.2) | -17 | Q93TH9 | Unknown | Unknown |
| [WP_029427683.1](file:///Volumes/ESD-USB/Summer%202018/Hassan%20Submission%20PLosOne/content.html#lcl|NZ_CP012801.1_prot_WP_029427683.1_4463) |  |  | + |  |  |  |  | 1.B.14.6.2 | -06 | Q93TH9 | Unknown | Unknown |
| WP_008766148.1 |  |  |  | + |  |  |  | 1.B.14.6.2 | -128 | Q93TH9 | Unknown | Unknown |
| WP_062694532.1 |  |  |  | + |  |  |  | 1.B.14.6.2 | -106 | Q93TH9 | Unknown | Unknown |
| WP_008767769.1 |  |  |  | + |  |  |  | 1.B.14.6.2 | -102 | Q93TH9 | Unknown | Unknown |
| WP_062694493.1 |  |  |  | + |  |  |  | 1.B.14.6.2 | -58 | Q93TH9 | Unknown | Unknown |
| WP_008767502.1 |  |  |  | + |  |  |  | 1.B.14.6.2 | -55 | Q93TH9 | Unknown | Unknown |
| WP_008766446.1 |  |  |  | + |  |  |  | 1.B.14.6.2 | -11 | Q93TH9 | Unknown | Unknown |
| YP_099176.1 |  |  |  |  | + |  |  | 1.B.14.6.2 | 0 | Q93TH9 | Molecules | Small molecules |
| YP_098037.1 |  |  |  |  | + |  |  | 1.B.14.6.2 | -138 | Q93TH9 | Molecules | Small molecules |
| YP_100463.1 |  |  |  |  | + |  |  | 1.B.14.6.2 | -96 | Q93TH9 | Molecules | Small molecules |
| YP_098537.1 |  |  |  |  | + |  |  | 1.B.14.6.2 | -92 | Q93TH9 | Molecules | Small molecules |
| YP_098340.1 |  |  |  |  | + |  |  | 1.B.14.6.2 | -92 | Q93TH9 | Molecules | Small molecules |
| YP_099003.1 |  |  |  |  | + |  |  | 1.B.14.6.2 | -72 | Q93TH9 | Molecules | Small molecules |
| YP_098991.1 |  |  |  |  | + |  |  | 1.B.14.6.2 | -71 | Q93TH9 | Molecules | Small molecules |
| YP_097836.1 |  |  |  |  | + |  |  | 1.B.14.6.2 | -66 | Q93TH9 | Molecules | Small molecules |
| [WP_004296737.1](file:///Volumes/ESD-USB/Summer%202018/Hassan%20Submission%20PLosOne/content.html#lcl|NZ_CP012938.1_prot_WP_004296737.1_1915) |  |  |  |  |  | + |  | 1.B.14.6.2 | -133 | Q93TH9 | Unknown | Unknown |
| [WP_004302254.1](file:///Volumes/ESD-USB/Summer%202018/Hassan%20Submission%20PLosOne/content.html#lcl|NZ_CP012938.1_prot_WP_004302254.1_4740) |  |  |  |  |  | + |  | 1.B.14.6.2 | -128 | Q93TH9 | Unknown | Unknown |
| [WP_004298558.1](file:///Volumes/ESD-USB/Summer%202018/Hassan%20Submission%20PLosOne/content.html#lcl|NZ_CP012938.1_prot_WP_004298558.1_2994) |  |  |  |  |  | + |  | 1.B.14.6.2 | -122 | Q93TH9 | Unknown | Unknown |
| [WP_004298393.1](file:///Volumes/ESD-USB/Summer%202018/Hassan%20Submission%20PLosOne/content.html#lcl|NZ_CP012938.1_prot_WP_004298393.1_2894) |  |  |  |  |  | + |  | 1.B.14.6.2 | -67 | Q93TH9 | Unknown | Unknown |
| [WP_004300602.1](file:///Volumes/ESD-USB/Summer%202018/Hassan%20Submission%20PLosOne/content.html#lcl|NZ_CP012938.1_prot_WP_004300602.1_1479) |  |  |  |  |  | + |  | 1.B.14.6.2 | -59 | Q93TH9 | Unknown | Unknown |
| [WP_004320878.10](file:///Volumes/ESD-USB/Summer%202018/Hassan%20Submission%20PLosOne/content.html#lcl|NZ_CP012938.1_prot_WP_004320878.1_4400) |  |  |  |  |  | + |  | 1.B.14.6.2 | -51 | Q93TH9 | Unknown | Unknown |
| [WP_004299453.1](file:///Volumes/ESD-USB/Summer%202018/Hassan%20Submission%20PLosOne/content.html#lcl|NZ_CP012938.1_prot_WP_004299453.1_2045) |  |  |  |  |  | + |  | 1.B.14.6.2 | -44 | Q93TH9 | Unknown | Unknown |
| [WP_004297975.1](file:///Volumes/ESD-USB/Summer%202018/Hassan%20Submission%20PLosOne/content.html#lcl|NZ_CP012938.1_prot_WP_004297975.1_1970) |  |  |  |  |  | + |  | 1.B.14.6.2 | -11 | Q93TH9 | Unknown | Unknown |
| [WP_004296734.1](file:///Volumes/ESD-USB/Summer%202018/Hassan%20Submission%20PLosOne/content.html#lcl|NZ_CP012938.1_prot_WP_004296734.1_1918) |  |  |  |  |  | + |  | 1.B.14.6.2 | -05 | Q93TH9 | Unknown | Unknown |
| [WP_008667530.1](file:///Volumes/ESD-USB/Summer%202018/Library/Application%20Support/Microsoft/Office/Office%202011%20AutoRecovery/content.html#lcl|NC_009614.1_prot_WP_008667530.1_554) |  |  |  |  |  |  | + | 1.B.14.6.2 | 0 | Q93TH9 | Unknown | Unknown |
| [WP_011964803.1](file:///Volumes/ESD-USB/Summer%202018/Library/Application%20Support/Microsoft/Office/Office%202011%20AutoRecovery/content.html#lcl|NC_009614.1_prot_WP_011964803.1_552) |  |  |  |  |  |  | + | 1.B.14.6.2 | 0 | Q93TH9 | Unknown | Unknown |
| [WP_011964806.1](file:///Volumes/ESD-USB/Summer%202018/Library/Application%20Support/Microsoft/Office/Office%202011%20AutoRecovery/content.html#lcl|NC_009614.1_prot_WP_011964806.1_559) |  |  |  |  |  |  | + | 1.B.14.6.2 | 0 | Q93TH9 | Unknown | Unknown |
| [WP_005850778.1](file:///Volumes/ESD-USB/Summer%202018/Library/Application%20Support/Microsoft/Office/Office%202011%20AutoRecovery/content.html#lcl|NC_009614.1_prot_WP_005850778.1_562) |  |  |  |  |  |  | + | 1.B.14.6.2 | -139 | Q93TH9 | Unknown | Unknown |
| [WP_011964836.1](file:///Volumes/ESD-USB/Summer%202018/Library/Application%20Support/Microsoft/Office/Office%202011%20AutoRecovery/content.html#lcl|NC_009614.1_prot_WP_011964836.1_614) |  |  |  |  |  |  | + | 1.B.14.6.2 | -138 | Q93TH9 | Unknown | Unknown |
| [WP_011965108.1](file:///Volumes/ESD-USB/Summer%202018/Library/Application%20Support/Microsoft/Office/Office%202011%20AutoRecovery/content.html#lcl|NC_009614.1_prot_WP_011965108.1_1235) |  |  |  |  |  |  | + | 1.B.14.6.2 | -97 | Q93TH9 | Unknown | Unknown |
| [WP_005850665.1](file:///Volumes/ESD-USB/Summer%202018/Library/Application%20Support/Microsoft/Office/Office%202011%20AutoRecovery/content.html#lcl|NC_009614.1_prot_WP_005850665.1_752) |  |  |  |  |  |  | + | 1.B.14.6.2 | -74 | Q93TH9 | Unknown | Unknown |
| [WP_012056019.1](file:///Volumes/ESD-USB/Summer%202018/Library/Application%20Support/Microsoft/Office/Office%202011%20AutoRecovery/content.html#lcl|NC_009614.1_prot_WP_012056019.1_4015) |  |  |  |  |  |  | + | 1.B.14.6.2 | -67 | Q93TH9 | Unknown | Unknown |
| [WP_011965410.1](file:///Volumes/ESD-USB/Summer%202018/Library/Application%20Support/Microsoft/Office/Office%202011%20AutoRecovery/content.html#lcl|NC_009614.1_prot_WP_011965410.1_1990) |  |  |  |  |  |  | + | 1.B.14.6.2 | -48 | Q93TH9 | Unknown | Unknown |
| [WP_011965523.1](file:///Volumes/ESD-USB/Summer%202018/Library/Application%20Support/Microsoft/Office/Office%202011%20AutoRecovery/content.html#lcl|NC_009614.1_prot_WP_011965523.1_2173) |  |  |  |  |  |  | + | 1.B.14.6.2 | -46 | Q93TH9 | Unknown | Unknown |
| [WP_005843060.1](file:///Volumes/ESD-USB/Summer%202018/Library/Application%20Support/Microsoft/Office/Office%202011%20AutoRecovery/content.html#lcl|NC_009614.1_prot_WP_005843060.1_115) |  |  |  |  |  |  | + | 1.B.14.6.2 | -15 | Q93TH9 | Unknown | Unknown |
| [WP_011964689.1](file:///Volumes/ESD-USB/Summer%202018/Library/Application%20Support/Microsoft/Office/Office%202011%20AutoRecovery/content.html#lcl|NC_009614.1_prot_WP_011964689.1_277) |  |  |  |  |  |  | + | 1.B.14.6.2 | -07 | Q93TH9 | Unknown | Unknown |
| [WP_052305972.1](file:///Volumes/ESD-USB/Summer%202018/Hassan%20Submission%20PLosOne/content.html#lcl|NC_015164.1_prot_WP_052305972.1_1481) |  | + |  |  |  |  |  | [1.B.14.6.3](http://tcdb.org/search/result.php?tc=1.B.14.6.3) | -17 | P38370 | Unknown | Unknown |
| WP_008765900.1 |  |  |  | + |  |  |  | 1.B.14.6.4 | -19 | B2FKT9 | Sugars | Monosaccharides/Oligo/Poly |
| WP_062695866.1 |  |  |  | + |  |  |  | 1.B.14.6.4 | -05 | B2FKT9 | Sugars | Monosaccharides/Oligo/Poly |
| ABI39_12510 | + |  |  |  |  |  |  | 1.B.14.6.5 | -06 | A5ZGZ4 | Unknown | Unknown |
| [WP_013618192.1](file:///Volumes/ESD-USB/Summer%202018/Hassan%20Submission%20PLosOne/content.html#lcl|NC_015164.1_prot_WP_013618192.1_2168) |  | + |  |  |  |  |  | [1.B.14.6.5](http://tcdb.org/search/result.php?tc=1.B.14.6.5) | 0 | A5ZGZ4 | Unknown | Unknown |
| [WP_013616299.1](file:///Volumes/ESD-USB/Summer%202018/Hassan%20Submission%20PLosOne/content.html#lcl|NC_015164.1_prot_WP_013616299.1_221) |  | + |  |  |  |  |  | [1.B.14.6.5](http://tcdb.org/search/result.php?tc=1.B.14.6.5) | 0.000554046 | A5ZGZ4 | Unknown | Unknown |
| [WP_029429127.1](file:///Volumes/ESD-USB/Summer%202018/Hassan%20Submission%20PLosOne/content.html#lcl|NZ_CP012801.1_prot_WP_029429127.1_4921) |  |  | + |  |  |  |  | [1.B.14.6.5](http://tcdb.org/search/result.php?tc=1.B.14.6.5) | -39 | A5ZGZ4 | Unknown | Unknown |
| [WP_029427467.1](file:///Volumes/ESD-USB/Summer%202018/Hassan%20Submission%20PLosOne/content.html#lcl|NZ_CP012801.1_prot_WP_029427467.1_3453) |  |  | + |  |  |  |  | [1.B.14.6.5](http://tcdb.org/search/result.php?tc=1.B.14.6.5) | -38 | A5ZGZ4 | Unknown | Unknown |
| [WP_029428606.1](file:///Volumes/ESD-USB/Summer%202018/Hassan%20Submission%20PLosOne/content.html#lcl|NZ_CP012801.1_prot_WP_029428606.1_4320) |  |  | + |  |  |  |  | [1.B.14.6.5](http://tcdb.org/search/result.php?tc=1.B.14.6.5) | -09 | A5ZGZ4 | Unknown | Unknown |
| [WP_029426233.1](file:///Volumes/ESD-USB/Summer%202018/Hassan%20Submission%20PLosOne/content.html#lcl|NZ_CP012801.1_prot_WP_029426233.1_2484) |  |  | + |  |  |  |  | [1.B.14.6.5](http://tcdb.org/search/result.php?tc=1.B.14.6.5) | -07 | A5ZGZ4 | Unknown | Unknown |
| WP_062695252.1 |  |  |  | + |  |  |  | 1.B.14.6.5 | 0 | A5ZGZ4 | Unknown | Unknown |
| WP_022470982.1 |  |  |  | + |  |  |  | 1.B.14.6.5 | -40 | A5ZGZ4 | Unknown | Unknown |
| WP_062694324.1 |  |  |  | + |  |  |  | 1.B.14.6.5 | -06 | A5ZGZ4 | Unknown | Unknown |
| YP_098686.1 |  |  |  |  | + |  |  | 1.B.14.6.5 | 0 | A5ZGZ4 | Molecules | Small molecules |
| YP_100514.1 |  |  |  |  | + |  |  | 1.B.14.6.5 | -12 | A5ZGZ4 | Molecules | Small molecules |
| YP_099185.1 |  |  |  |  | + |  |  | 1.B.14.6.5 | -05 | A5ZGZ4 | Molecules | Small molecules |
| [WP_004296961.1](file:///Volumes/ESD-USB/Summer%202018/Hassan%20Submission%20PLosOne/content.html#lcl|NZ_CP012938.1_prot_WP_004296961.1_46) |  |  |  |  |  | + |  | [1.B.14.6.5](http://tcdb.org/search/result.php?tc=1.B.14.6.5) | 0 | A5ZGZ4 | Unknown | Unknown |
| [WP_004296011.1](file:///Volumes/ESD-USB/Summer%202018/Hassan%20Submission%20PLosOne/content.html#lcl|NZ_CP012938.1_prot_WP_004296011.1_676) |  |  |  |  |  | + |  | [1.B.14.6.5](http://tcdb.org/search/result.php?tc=1.B.14.6.5) | -43 | A5ZGZ4 | Unknown | Unknown |
| [WP_008782093.1](file:///Volumes/ESD-USB/Summer%202018/Library/Application%20Support/Microsoft/Office/Office%202011%20AutoRecovery/content.html#lcl|NC_009614.1_prot_WP_008782093.1_2459) |  |  |  |  |  |  | + | [1.B.14.6.5](http://tcdb.org/search/result.php?tc=1.B.14.6.5) | -06 | A5ZGZ4 | Unknown | Unknown |
| [WP_011965068.1](file:///Volumes/ESD-USB/Summer%202018/Library/Application%20Support/Microsoft/Office/Office%202011%20AutoRecovery/content.html#lcl|NC_009614.1_prot_WP_011965068.1_1126) |  |  |  |  |  |  | + | [1.B.14.6.5](http://tcdb.org/search/result.php?tc=1.B.14.6.5) | -06 | A5ZGZ4 | Unknown | Unknown |
| WP_029428655.1 |  |  | + |  |  |  |  | [1.B.14.6.7](http://tcdb.org/search/result.php?tc=1.B.14.6.7) | -157 | C6XVY5 | Unknown | Unknown |
| ABI39_13440 | + |  |  |  |  |  |  | 1.B.14.6.8 | -13 | Q64VG5 | Unknown | Unknown |
| ABI39_06995 | + |  |  |  |  |  |  | 1.B.14.6.8 | -10 | Q64VG5 | Unknown | Unknown |
| ABI39_10770 | + |  |  |  |  |  |  | 1.B.14.6.8 | -07 | Q64VG5 | Unknown | Unknown |
| ABI39_06860 | + |  |  |  |  |  |  | 1.B.14.6.8 | -07 | Q64VG5 | Unknown | Unknown |
| ABI39_07540 | + |  |  |  |  |  |  | 1.B.14.6.8 | -05 | Q64VG5 | Unknown | Unknown |
| [WP_013617475.1](file:///Volumes/ESD-USB/Summer%202018/Hassan%20Submission%20PLosOne/content.html#lcl|NC_015164.1_prot_WP_013617475.1_1440) |  | + |  |  |  |  |  | [1.B.14.6.8](http://tcdb.org/search/result.php?tc=1.B.14.6.8) | -12 | Q64VG5 | Unknown | Unknown |
| [WP_013617042.1](file:///Volumes/ESD-USB/Summer%202018/Hassan%20Submission%20PLosOne/content.html#lcl|NC_015164.1_prot_WP_013617042.1_993) |  | + |  |  |  |  |  | [1.B.14.6.8](http://tcdb.org/search/result.php?tc=1.B.14.6.8) | -09 | Q64VG5 | Unknown | Unknown |
| [WP_041584200.1](file:///Volumes/ESD-USB/Summer%202018/Hassan%20Submission%20PLosOne/content.html#lcl|NC_015164.1_prot_WP_041584200.1_586) |  | + |  |  |  |  |  | [1.B.14.6.8](http://tcdb.org/search/result.php?tc=1.B.14.6.8) | -09 | Q64VG5 | Unknown | Unknown |
| [WP_013618413.1](file:///Volumes/ESD-USB/Summer%202018/Hassan%20Submission%20PLosOne/content.html#lcl|NC_015164.1_prot_WP_013618413.1_2408) |  | + |  |  |  |  |  | [1.B.14.6.8](http://tcdb.org/search/result.php?tc=1.B.14.6.8) | -08 | Q64VG5 | Unknown | Unknown |
| [WP_041584517.1](file:///Volumes/ESD-USB/Summer%202018/Hassan%20Submission%20PLosOne/content.html#lcl|NC_015164.1_prot_WP_041584517.1_3543) |  | + |  |  |  |  |  | [1.B.14.6.8](http://tcdb.org/search/result.php?tc=1.B.14.6.8) | -08 | Q64VG5 | Unknown | Unknown |
| [WP_013619145.1](file:///Volumes/ESD-USB/Summer%202018/Hassan%20Submission%20PLosOne/content.html#lcl|NC_015164.1_prot_WP_013619145.1_3198) |  | + |  |  |  |  |  | [1.B.14.6.8](http://tcdb.org/search/result.php?tc=1.B.14.6.8) | -07 | Q64VG5 | Unknown | Unknown |
| [WP_013617672.1](file:///Volumes/ESD-USB/Summer%202018/Hassan%20Submission%20PLosOne/content.html#lcl|NC_015164.1_prot_WP_013617672.1_1641) |  | + |  |  |  |  |  | [1.B.14.6.8](http://tcdb.org/search/result.php?tc=1.B.14.6.8) | -06 | Q64VG5 | Unknown | Unknown |
| [WP_007219254.1](file:///Volumes/ESD-USB/Summer%202018/Hassan%20Submission%20PLosOne/content.html#lcl|NZ_CP012801.1_prot_WP_007219254.1_2987) |  |  | + |  |  |  |  | [1.B.14.6.8](http://tcdb.org/search/result.php?tc=1.B.14.6.8) | -11 | Q64VG5 | Unknown | Unknown |
| [WP_033160463.1](file:///Volumes/ESD-USB/Summer%202018/Hassan%20Submission%20PLosOne/content.html#lcl|NZ_CP012801.1_prot_WP_033160463.1_429) |  |  | + |  |  |  |  | [1.B.14.6.8](http://tcdb.org/search/result.php?tc=1.B.14.6.8) | -07 | Q64VG5 | Unknown | Unknown |
| WP_008764436.1 |  |  |  | + |  |  |  | 1.B.14.6.8 | -11 | Q64VG5 | Unknown | Unknown |
| WP_008761919.1 |  |  |  | + |  |  |  | 1.B.14.6.8 | -11 | Q64VG5 | Unknown | Unknown |
| WP_055299495.1 |  |  |  | + |  |  |  | 1.B.14.6.8 | -09 | Q64VG5 | Unknown | Unknown |
| WP_062695756.1 |  |  |  | + |  |  |  | 1.B.14.6.8 | -08 | Q64VG5 | Unknown | Unknown |
| WP_032840999.1 |  |  |  | + |  |  |  | 1.B.14.6.8 | -05 | Q64VG5 | Unknown | Unknown |
| YP_099045.1 |  |  |  |  | + |  |  | 1.B.14.6.8 | 0 | Q64VG5 | Molecules | Small molecules |
| YP_100680.1 |  |  |  |  | + |  |  | 1.B.14.6.8 | -132 | Q64VG5 | Molecules | Small molecules |
| YP_098891.1 |  |  |  |  | + |  |  | 1.B.14.6.8 | -11 | Q64VG5 | Molecules | Small molecules |
| YP_101756.1 |  |  |  |  | + |  |  | 1.B.14.6.8 | -09 | Q64VG5 | Molecules | Small molecules |
| YP_099406.1 |  |  |  |  | + |  |  | 1.B.14.6.8 | -07 | Q64VG5 | Molecules | Small molecules |
| [WP_004296527.1](file:///Volumes/ESD-USB/Summer%202018/Hassan%20Submission%20PLosOne/content.html#lcl|NZ_CP012938.1_prot_WP_004296527.1_2809) |  |  |  |  |  | + |  | [1.B.14.6.8](http://tcdb.org/search/result.php?tc=1.B.14.6.8) | -12 | Q64VG5 | Unknown | Unknown |
| [WP_004322478.1](file:///Volumes/ESD-USB/Summer%202018/Hassan%20Submission%20PLosOne/content.html#lcl|NZ_CP012938.1_prot_WP_004322478.1_3406) |  |  |  |  |  | + |  | [1.B.14.6.8](http://tcdb.org/search/result.php?tc=1.B.14.6.8) | -11 | Q64VG5 | Unknown | Unknown |
| [WP_004321329.1](file:///Volumes/ESD-USB/Summer%202018/Hassan%20Submission%20PLosOne/content.html#lcl|NZ_CP012938.1_prot_WP_004321329.1_1436) |  |  |  |  |  | + |  | [1.B.14.6.8](http://tcdb.org/search/result.php?tc=1.B.14.6.8) | -10 | Q64VG5 | Unknown | Unknown |
| [WP_004296167.1](file:///Volumes/ESD-USB/Summer%202018/Hassan%20Submission%20PLosOne/content.html#lcl|NZ_CP012938.1_prot_WP_004296167.1_2494) |  |  |  |  |  | + |  | [1.B.14.6.8](http://tcdb.org/search/result.php?tc=1.B.14.6.8) | -07 | Q64VG5 | Unknown | Unknown |
| [WP_004300439.1](file:///Volumes/ESD-USB/Summer%202018/Hassan%20Submission%20PLosOne/content.html#lcl|NZ_CP012938.1_prot_WP_004300439.1_533) |  |  |  |  |  | + |  | [1.B.14.6.8](http://tcdb.org/search/result.php?tc=1.B.14.6.8) | -06 | Q64VG5 | Unknown | Unknown |
| [WP_004296041.1](file:///Volumes/ESD-USB/Summer%202018/Hassan%20Submission%20PLosOne/content.html#lcl|NZ_CP012938.1_prot_WP_004296041.1_646) |  |  |  |  |  | + |  | [1.B.14.6.8](http://tcdb.org/search/result.php?tc=1.B.14.6.8) | -05 | Q64VG5 | Unknown | Unknown |
| [WP_005844474.1](file:///Volumes/ESD-USB/Summer%202018/Library/Application%20Support/Microsoft/Office/Office%202011%20AutoRecovery/content.html#lcl|NC_009614.1_prot_WP_005844474.1_862) |  |  |  |  |  |  | + | [1.B.14.6.8](http://tcdb.org/search/result.php?tc=1.B.14.6.8) | -10 | Q64VG5 | Unknown | Unknown |
| [WP_005844424.1](file:///Volumes/ESD-USB/Summer%202018/Library/Application%20Support/Microsoft/Office/Office%202011%20AutoRecovery/content.html#lcl|NC_009614.1_prot_WP_005844424.1_890) |  |  |  |  |  |  | + | [1.B.14.6.8](http://tcdb.org/search/result.php?tc=1.B.14.6.8) | -06 | Q64VG5 | Unknown | Unknown |
| ABI39_10155 | + |  |  |  |  |  |  | 1.B.14.6.9 | -06 | A3UAU5 | Unknown | Unknown |
| [WP_065757456.1](file:///Volumes/ESD-USB/Summer%202018/Hassan%20Submission%20PLosOne/content.html#lcl|NC_015164.1_prot_WP_065757456.1_1591) |  | + |  |  |  |  |  | [1.B.14.6.9](http://tcdb.org/search/result.php?tc=1.B.14.6.9) | -12 | A3UAU5 | Unknown | Unknown |
| [WP_029427746.1](file:///Volumes/ESD-USB/Summer%202018/Hassan%20Submission%20PLosOne/content.html#lcl|NZ_CP012801.1_prot_WP_029427746.1_1779) |  |  | + |  |  |  |  | [1.B.14.6.9](http://tcdb.org/search/result.php?tc=1.B.14.6.9) | -61 | A3UAU5 | Unknown | Unknown |
| [WP_044128974.1](file:///Volumes/ESD-USB/Summer%202018/Hassan%20Submission%20PLosOne/content.html#lcl|NZ_CP012801.1_prot_WP_044128974.1_4162) |  |  | + |  |  |  |  | [1.B.14.6.9](http://tcdb.org/search/result.php?tc=1.B.14.6.9) | -18 | A3UAU5 | Unknown | Unknown |
| WP_011109207.1 |  |  |  | + |  |  |  | 1.B.14.6.9 | -11 | A3UAU5 | Unknown | Unknown |
| [WP_004301839.1](file:///Volumes/ESD-USB/Summer%202018/Hassan%20Submission%20PLosOne/content.html#lcl|NZ_CP012938.1_prot_WP_004301839.1_4407) |  |  |  |  |  | + |  | [1.B.14.6.9](http://tcdb.org/search/result.php?tc=1.B.14.6.9) | -10 | A3UAU5 | Unknown | Unknown |
| [WP_012055766.1](file:///Volumes/ESD-USB/Summer%202018/Library/Application%20Support/Microsoft/Office/Office%202011%20AutoRecovery/content.html#lcl|NC_009614.1_prot_WP_012055766.1_3497) |  |  |  |  |  |  | + | [1.B.14.6.9](http://tcdb.org/search/result.php?tc=1.B.14.6.9) | -06 | A3UAU5 | Unknown | Unknown |
| ABI39_01480 | + |  |  |  |  |  |  | 1.B.14.6.10 | -07 | F9YSZ3 | Unknown | Unknown |
| ABI39_10140 | + |  |  |  |  |  |  | 1.B.14.6.10 | -06 | F9YSZ3 | Unknown | Unknown |
| WP_008760358.1 |  |  |  | + |  |  |  | 1.B.14.6.10 | -07 | F9YSZ3 | Unknown | Unknown |
| [WP_004302170.1_](file:///Volumes/ESD-USB/Summer%202018/Hassan%20Submission%20PLosOne/content.html#lcl|NZ_CP012938.1_prot_WP_004302170.1_4663) |  |  |  |  |  | + |  | [1.B.14.6.10](http://tcdb.org/search/result.php?tc=1.B.14.6.10) | -05 | F9YSZ3 | Unknown | Unknown |
| [WP_013617365.1](file:///Volumes/ESD-USB/Summer%202018/Hassan%20Submission%20PLosOne/content.html#lcl|NC_015164.1_prot_WP_013617365.1_1336) |  | + |  |  |  |  |  | [1.B.14.6.11](http://tcdb.org/search/result.php?tc=1.B.14.6.11) | -18 | G0LBU8 | Unknown | Unknown |
| WP_062695294.1 |  |  |  | + |  |  |  | 1.B.14.6.11 | -08 | G0LBU8 | Unknown | Unknown |
| YP_099311.1 |  |  |  |  | + |  |  | 1.B.14.6.11 | -13 | G0LBU8 | Molecules | Small molecules |
| YP_100349.1 |  |  |  |  | + |  |  | 1.B.14.6.11 | -05 | G0LBU8 | Molecules | Small molecules |
| ABI39_00110 | + |  |  |  |  |  |  | 1.B.14.6.12 | -143 | H6L5X3 | Unknown | Unknown |
| ABI39_00135 | + |  |  |  |  |  |  | 1.B.14.6.12 | -135 | H6L5X3 | Unknown | Unknown |
| [WP_029428761.1](file:///Volumes/ESD-USB/Summer%202018/Hassan%20Submission%20PLosOne/content.html#lcl|NZ_CP012801.1_prot_WP_029428761.1_4048) |  |  | + |  |  |  |  | [1.B.14.6.12](http://tcdb.org/search/result.php?tc=1.B.14.6.12) | -149 | H6L5X3 | Unknown | Unknown |
| [WP_029427866.1](file:///Volumes/ESD-USB/Summer%202018/Hassan%20Submission%20PLosOne/content.html#lcl|NZ_CP012801.1_prot_WP_029427866.1_1599) |  |  | + |  |  |  |  | [1.B.14.6.12](http://tcdb.org/search/result.php?tc=1.B.14.6.12) | -149 | H6L5X3 | Unknown | Unknown |
| [WP_029427530.1](file:///Volumes/ESD-USB/Summer%202018/Hassan%20Submission%20PLosOne/content.html#lcl|NZ_CP012801.1_prot_WP_029427530.1_3362) |  |  | + |  |  |  |  | [1.B.14.6.12](http://tcdb.org/search/result.php?tc=1.B.14.6.12) | -06 | H6L5X3 | Unknown | Unknown |
| WP_062694567.1 |  |  |  | + |  |  |  | 1.B.14.6.12 | -165 | H6L5X3 | Unknown | Unknown |
| WP_005829323.1 |  |  |  | + |  |  |  | 1.B.14.6.12 |  | H6L5X3 | Unknown | Unknown |
| [WP_004299318.1](file:///Volumes/ESD-USB/Summer%202018/Hassan%20Submission%20PLosOne/content.html#lcl|NZ_CP012938.1_prot_WP_004299318.1_2145) |  |  |  |  |  | + |  | [1.B.14.6.12](http://tcdb.org/search/result.php?tc=1.B.14.6.12) | -158 | H6L5X3 | Unknown | Unknown |
| [WP_005844649.1](file:///Volumes/ESD-USB/Summer%202018/Library/Application%20Support/Microsoft/Office/Office%202011%20AutoRecovery/content.html#lcl|NC_009614.1_prot_WP_005844649.1_29) |  |  |  |  |  |  | + | [1.B.14.6.12](http://tcdb.org/search/result.php?tc=1.B.14.6.12) | -134 | H6L5X3 | Unknown | Unknown |
| ABI39_06305 | + |  |  |  |  |  |  | 1.B.14.6.13 | 0 | Q8A8X1 | Unknown | Unknown |
| ABI39_10805 | + |  |  |  |  |  |  | 1.B.14.6.13 | -174 | Q8A8X1 | Unknown | Unknown |
| [WP_013616870.1](file:///Volumes/ESD-USB/Summer%202018/Hassan%20Submission%20PLosOne/content.html#lcl|NC_015164.1_prot_WP_013616870.1_812) |  | + |  |  |  |  |  | [1.B.14.6.13](http://tcdb.org/search/result.php?tc=1.B.14.6.13) | 0 | Q8A8X1 | Unknown | Unknown |
| [WP_026367853.1](file:///Volumes/ESD-USB/Summer%202018/Hassan%20Submission%20PLosOne/content.html#lcl|NZ_CP012801.1_prot_WP_026367853.1_4075) |  |  | + |  |  |  |  | [1.B.14.6.13](http://tcdb.org/search/result.php?tc=1.B.14.6.13) | 0 | Q8A8X1 | Unknown | Unknown |
| [WP_029426611.1](file:///Volumes/ESD-USB/Summer%202018/Hassan%20Submission%20PLosOne/content.html#lcl|NZ_CP012801.1_prot_WP_029426611.1_3142) |  |  | + |  |  |  |  | [1.B.14.6.13](http://tcdb.org/search/result.php?tc=1.B.14.6.13) | 0 | Q8A8X1 | Unknown | Unknown |
| [WP_029426780.1](file:///Volumes/ESD-USB/Summer%202018/Hassan%20Submission%20PLosOne/content.html#lcl|NZ_CP012801.1_prot_WP_029426780.1_434) |  |  | + |  |  |  |  | [1.B.14.6.13](http://tcdb.org/search/result.php?tc=1.B.14.6.13) | 0 | Q8A8X1 | Unknown | Unknown |
| WP_008762688.1 |  |  |  | + |  |  |  | 1.B.14.6.13 | 0 | Q8A8X1 | Unknown | Unknown |
| WP_008764985.1 |  |  |  | + |  |  |  | 1.B.14.6.13 | 0 | Q8A8X1 | Unknown | Unknown |
| WP_008765833.1 |  |  |  | + |  |  |  | 1.B.14.6.13 | 0 | Q8A8X1 | Unknown | Unknown |
| WP_008766779.1 |  |  |  | + |  |  |  | 1.B.14.6.13 | 0 | Q8A8X1 | Unknown | Unknown |
| WP_008767141.1 |  |  |  | + |  |  |  | 1.B.14.6.13 | 0 | Q8A8X1 | Unknown | Unknown |
| WP_011109268.1 |  |  |  | + |  |  |  | 1.B.14.6.13 | 0 | Q8A8X1 | Unknown | Unknown |
| WP_022470100.1 |  |  |  | + |  |  |  | 1.B.14.6.13 | 0 | Q8A8X1 | Unknown | Unknown |
| WP_048695006.1 |  |  |  | + |  |  |  | 1.B.14.6.13 | 0 | Q8A8X1 | Unknown | Unknown |
| WP_055217007.1 |  |  |  | + |  |  |  | 1.B.14.6.13 | 0 | Q8A8X1 | Unknown | Unknown |
| WP_062695119.1 |  |  |  | + |  |  |  | 1.B.14.6.13 | 0 | Q8A8X1 | Unknown | Unknown |
| WP_062695755.1 |  |  |  | + |  |  |  | 1.B.14.6.13 | 0 | Q8A8X1 | Unknown | Unknown |
| WP_062694534.1 |  |  |  | + |  |  |  | 1.B.14.6.13 | -125 | Q8A8X1 | Unknown | Unknown |
| WP_062694601.1 |  |  |  | + |  |  |  | 1.B.14.6.13 | -74 | Q8A8X1 | Unknown | Unknown |
| YP_098609.1 |  |  |  |  | + |  |  | 1.B.14.6.13 | 0 | Q8A8X1 | Molecules | Small molecules |
| YP_100888.1 |  |  |  |  | + |  |  | 1.B.14.6.13 | 0 | Q8A8X1 | Molecules | Small molecules |
| YP_101601.1 |  |  |  |  | + |  |  | 1.B.14.6.13 | 0 | Q8A8X1 | Molecules | Small molecules |
| YP_097948.1 |  |  |  |  | + |  |  | 1.B.14.6.13 | -78 | Q8A8X1 | Molecules | Small molecules |
| [WP_004296888.1](file:///Volumes/ESD-USB/Summer%202018/Hassan%20Submission%20PLosOne/content.html#lcl|NZ_CP012938.1_prot_WP_004296888.1_3171) |  |  |  |  |  | + |  | [1.B.14.6.13](http://tcdb.org/search/result.php?tc=1.B.14.6.13) | 0 | Q8A8X1 | Unknown | Unknown |
| [WP_004299792.1](file:///Volumes/ESD-USB/Summer%202018/Hassan%20Submission%20PLosOne/content.html#lcl|NZ_CP012938.1_prot_WP_004299792.1_3683) |  |  |  |  |  | + |  | [1.B.14.6.13](http://tcdb.org/search/result.php?tc=1.B.14.6.13) | 0 | Q8A8X1 | Unknown | Unknown |
| [WP_004301370.1](file:///Volumes/ESD-USB/Summer%202018/Hassan%20Submission%20PLosOne/content.html#lcl|NZ_CP012938.1_prot_WP_004301370.1_4097) |  |  |  |  |  | + |  | [1.B.14.6.13](http://tcdb.org/search/result.php?tc=1.B.14.6.13) | 0 | Q8A8X1 | Unknown | Unknown |
| [WP_032853833.1](file:///Volumes/ESD-USB/Summer%202018/Hassan%20Submission%20PLosOne/content.html#lcl|NZ_CP012938.1_prot_WP_032853833.1_4509) |  |  |  |  |  | + |  | [1.B.14.6.13](http://tcdb.org/search/result.php?tc=1.B.14.6.13) | 0 | Q8A8X1 | Unknown | Unknown |
| [WP_052588029.1](file:///Volumes/ESD-USB/Summer%202018/Hassan%20Submission%20PLosOne/content.html#lcl|NZ_CP012938.1_prot_WP_052588029.1_4756) |  |  |  |  |  | + |  | [1.B.14.6.13](http://tcdb.org/search/result.php?tc=1.B.14.6.13) | 0 | Q8A8X1 | Unknown | Unknown |
| [WP_055167753.1](file:///Volumes/ESD-USB/Summer%202018/Hassan%20Submission%20PLosOne/content.html#lcl|NZ_CP012938.1_prot_WP_055167753.1_557) |  |  |  |  |  | + |  | [1.B.14.6.13](http://tcdb.org/search/result.php?tc=1.B.14.6.13) | 0 | Q8A8X1 | Unknown | Unknown |
| [WP_059365491.1](file:///Volumes/ESD-USB/Summer%202018/Hassan%20Submission%20PLosOne/content.html#lcl|NZ_CP012938.1_prot_WP_059365491.1_554) |  |  |  |  |  | + |  | [1.B.14.6.13](http://tcdb.org/search/result.php?tc=1.B.14.6.13) | 0 | Q8A8X1 | Unknown | Unknown |
| [WP_044919503.1](file:///Volumes/ESD-USB/Summer%202018/Hassan%20Submission%20PLosOne/content.html#lcl|NZ_CP012938.1_prot_WP_044919503.1_2227) |  |  |  |  |  | + |  | [1.B.14.6.13](http://tcdb.org/search/result.php?tc=1.B.14.6.13) | -125 | Q8A8X1 | Unknown | Unknown |
| [WP_052587917.1](file:///Volumes/ESD-USB/Summer%202018/Hassan%20Submission%20PLosOne/content.html#lcl|NZ_CP012938.1_prot_WP_052587917.1_1971) |  |  |  |  |  | + |  | [1.B.14.6.13](http://tcdb.org/search/result.php?tc=1.B.14.6.13) | -76 | Q8A8X1 | Unknown | Unknown |
| [WP_011964833.1](file:///Volumes/ESD-USB/Summer%202018/Library/Application%20Support/Microsoft/Office/Office%202011%20AutoRecovery/content.html#lcl|NC_009614.1_prot_WP_011964833.1_610) |  |  |  |  |  |  | + | [1.B.14.6.13](http://tcdb.org/search/result.php?tc=1.B.14.6.13) | 0 | Q8A8X1 | Unknown | Unknown |
| [WP_011965024.1](file:///Volumes/ESD-USB/Summer%202018/Library/Application%20Support/Microsoft/Office/Office%202011%20AutoRecovery/content.html#lcl|NC_009614.1_prot_WP_011965024.1_1036) |  |  |  |  |  |  | + | [1.B.14.6.13](http://tcdb.org/search/result.php?tc=1.B.14.6.13) | -179 | Q8A8X1 | Unknown | Unknown |
| [WP_041174449.1](file:///Volumes/ESD-USB/Summer%202018/Library/Application%20Support/Microsoft/Office/Office%202011%20AutoRecovery/content.html#lcl|NC_009614.1_prot_WP_041174449.1_2158) |  |  |  |  |  |  | + | [1.B.14.6.13](http://tcdb.org/search/result.php?tc=1.B.14.6.13) | -174 | Q8A8X1 | Unknown | Unknown |
| [WP_041584477.1](file:///Volumes/ESD-USB/Summer%202018/Hassan%20Submission%20PLosOne/content.html#lcl|NC_015164.1_prot_WP_041584477.1_3223) |  | + |  |  |  |  |  | [1.B.14.6.14](http://tcdb.org/search/result.php?tc=1.B.14.6.14) | -96 | I3YT95 | Unknown | Unknown |
| [WP_013616080.1](file:///Volumes/ESD-USB/Summer%202018/Hassan%20Submission%20PLosOne/content.html#lcl|NC_015164.1_prot_WP_013616080.1_3) |  | + |  |  |  |  |  | [1.B.14.6.14](http://tcdb.org/search/result.php?tc=1.B.14.6.14) | -89 | I3YT95 | Unknown | Unknown |
| [WP_029428906.1](file:///Volumes/ESD-USB/Summer%202018/Hassan%20Submission%20PLosOne/content.html#lcl|NZ_CP012801.1_prot_WP_029428906.1_602) |  |  | + |  |  |  |  | [1.B.14.6.14](http://tcdb.org/search/result.php?tc=1.B.14.6.14) | -93 | I3YT95 | Unknown | Unknown |
| [WP_029429122.1](file:///Volumes/ESD-USB/Summer%202018/Hassan%20Submission%20PLosOne/content.html#lcl|NZ_CP012801.1_prot_WP_029429122.1_4931) |  |  | + |  |  |  |  | [1.B.14.6.14](http://tcdb.org/search/result.php?tc=1.B.14.6.14) | -83 | I3YT95 | Unknown | Unknown |
| [WP_029428452.1](file:///Volumes/ESD-USB/Summer%202018/Hassan%20Submission%20PLosOne/content.html#lcl|NZ_CP012801.1_prot_WP_029428452.1_1008) |  |  | + |  |  |  |  | [1.B.14.6.14](http://tcdb.org/search/result.php?tc=1.B.14.6.14) | -81 | I3YT95 | Unknown | Unknown |
| WP_008765914.1 |  |  |  | + |  |  |  | 1.B.14.6.14 | -94 | I3YT95 | Unknown | Unknown |
| WP_062694798.1 |  |  |  | + |  |  |  | 1.B.14.6.14 | -86 | I3YT95 | Unknown | Unknown |
| [WP_004300339.1](file:///Volumes/ESD-USB/Summer%202018/Hassan%20Submission%20PLosOne/content.html#lcl|NZ_CP012938.1_prot_WP_004300339.1_444) |  |  |  |  |  | + |  | [1.B.14.6.14](http://tcdb.org/search/result.php?tc=1.B.14.6.14) | -93 | I3YT95 | Unknown | Unknown |
| [WP_044918747.1](file:///Volumes/ESD-USB/Summer%202018/Hassan%20Submission%20PLosOne/content.html#lcl|NZ_CP012938.1_prot_WP_044918747.1_1544) |  |  |  |  |  | + |  | [1.B.14.6.14](http://tcdb.org/search/result.php?tc=1.B.14.6.14) | -86 | I3YT95 | Unknown | Unknown |
| [WP_029427500.1](file:///Volumes/ESD-USB/Summer%202018/Hassan%20Submission%20PLosOne/content.html#lcl|NZ_CP012801.1_prot_WP_029427500.1_3415) |  |  | + |  |  |  |  | [1.B.14.8.2](http://tcdb.org/search/result.php?tc=1.B.14.8.2) | -23 | Q2M5P4 | Unknown | Unknown |
| WP_032840464.1 |  |  |  | + |  |  |  | 1.B.14.8.2 | -22 | Q2M5P4 | Siderophores | Pyridine-2,6-bis(thiocarboxylic acid) |
| [WP_004300147.1](file:///Volumes/ESD-USB/Summer%202018/Hassan%20Submission%20PLosOne/content.html#lcl|NZ_CP012938.1_prot_WP_004300147.1_1207) |  |  |  |  |  | + |  | [1.B.14.10.1](http://tcdb.org/search/result.php?tc=1.B.14.10.1) | -104 | P95494 | Unknown | Unknown |
| [WP_033160745.1](file:///Volumes/ESD-USB/Summer%202018/Hassan%20Submission%20PLosOne/content.html#lcl|NZ_CP012801.1_prot_WP_033160745.1_1434) |  |  | + |  |  |  |  | [1.B.14.10.2](http://tcdb.org/search/result.php?tc=1.B.14.10.2) | -09 | Q1DCQ0 | Unknown | Unknown |
| WP_011107990.1 |  |  |  | + |  |  |  | 1.B.14.10.3 | -16 | Q1CZJ4 | Unknown | Unknown |
| [WP_004301024.1](file:///Volumes/ESD-USB/Summer%202018/Hassan%20Submission%20PLosOne/content.html#lcl|NZ_CP012938.1_prot_WP_004301024.1_1775) |  |  |  |  |  | + |  | [1.B.14.10.3](http://tcdb.org/search/result.php?tc=1.B.14.10.3) | -17 | Q1CZJ4 | Unknown | Unknown |
| [WP_004301021.1](file:///Volumes/ESD-USB/Summer%202018/Hassan%20Submission%20PLosOne/content.html#lcl|NZ_CP012938.1_prot_WP_004301021.1_1772) |  |  |  |  |  | + |  | [1.B.14.10.3](http://tcdb.org/search/result.php?tc=1.B.14.10.3) | -16 | Q1CZJ4 | Unknown | Unknown |
| WP_011108585.1 |  |  |  | + |  |  |  | 1.B.14.12.2 | -19 | Q9AAZ6 | Unknown | Unknown |
| WP_032840500.1 |  |  |  | + |  |  |  | 1.B.14.12.3 | -48 | Q1DFM0 | Unknown | Unknown |
| WP_062694463.1 |  |  |  | + |  |  |  | 1.B.14.12.3 | -07 | Q1DFM0 | Unknown | Unknown |
| [WP_004301446.1](file:///Volumes/ESD-USB/Summer%202018/Hassan%20Submission%20PLosOne/content.html#lcl|NZ_CP012938.1_prot_WP_004301446.1_4136) |  |  |  |  |  | + |  | [1.B.14.12.3](http://tcdb.org/search/result.php?tc=1.B.14.12.3) | -49 | Q1DFM0 | Unknown | Unknown |
| ABI39_14080 | + |  |  |  |  |  |  | 1.B.14.14.1 | -18 | Q8A552 | Unknown | Unknown |
| [WP_007219835.1](file:///Volumes/ESD-USB/Summer%202018/Hassan%20Submission%20PLosOne/content.html#lcl|NZ_CP012801.1_prot_WP_007219835.1_3449) |  |  | + |  |  |  |  | [1.B.14.14.1](http://tcdb.org/search/result.php?tc=1.B.14.14.1) | 0 | Q8A552 | Vitamins | Thiamine |
| [WP_007213600.1](file:///Volumes/ESD-USB/Summer%202018/Hassan%20Submission%20PLosOne/content.html#lcl|NZ_CP012801.1_prot_WP_007213600.1_610) |  |  | + |  |  |  |  | [1.B.14.14.1](http://tcdb.org/search/result.php?tc=1.B.14.14.1) | -19 | Q8A552 | Vitamins | Thiamine |
| WP_062694443.1 |  |  |  | + |  |  |  | 1.B.14.14.1 | 0 | Q8A552 | Vitamins | Thiamine |
| YP_097898.1 |  |  |  |  | + |  |  | 1.B.14.14.1 | 0 | Q8A552 | Vitamins | Thiamine |
| YP_099273.1 |  |  |  |  | + |  |  | 1.B.14.14.1 | -17 | Q8A552 | Vitamins | Thiamine |
| [WP_004299131.1](file:///Volumes/ESD-USB/Summer%202018/Hassan%20Submission%20PLosOne/content.html#lcl|NZ_CP012938.1_prot_WP_004299131.1_2271) |  |  |  |  |  | + |  | [1.B.14.14.1](http://tcdb.org/search/result.php?tc=1.B.14.14.1) | 0 | Q8A552 | Unknown | Unknown |
| [WP_008667283.1](file:///Volumes/ESD-USB/Summer%202018/Library/Application%20Support/Microsoft/Office/Office%202011%20AutoRecovery/content.html#lcl|NC_009614.1_prot_WP_008667283.1_2698) |  |  |  |  |  |  | + | [1.B.14.14.1](http://tcdb.org/search/result.php?tc=1.B.14.14.1) | -18 | Q8A552 | Vitamins | Thiamine |
| ABI39_11565 | + |  |  |  |  |  |  | 1.B.14.15.2 | -106 | D5ESR8 | Unknown | Unknown |
| [WP_029428499.1](file:///Volumes/ESD-USB/Summer%202018/Hassan%20Submission%20PLosOne/content.html#lcl|NZ_CP012801.1_prot_WP_029428499.1_890) |  |  | + |  |  |  |  | [1.B.14.15.2](http://tcdb.org/search/result.php?tc=1.B.14.15.2) | -167 | D5ESR8 | Unknown | Unknown |
| WP_011107202.1 |  |  |  | + |  |  |  | 1.B.14.15.2 | -92 | D5ESR8 | Unknown | Unknown |
| [WP_004297414.1](file:///Volumes/ESD-USB/Summer%202018/Hassan%20Submission%20PLosOne/content.html#lcl|NZ_CP012938.1_prot_WP_004297414.1_172) |  |  |  |  |  | + |  | [1.B.14.15.2](http://tcdb.org/search/result.php?tc=1.B.14.15.2) | -96 | D5ESR8 | Unknown | Unknown |
| WP_062695219.1 |  |  |  | + |  |  |  | 1.B.14.16.4 | -07 | D8EPC3 | Unknown | Unknown |
| [WP_011965296.1](file:///Volumes/ESD-USB/Summer%202018/Library/Application%20Support/Microsoft/Office/Office%202011%20AutoRecovery/content.html#lcl|NC_009614.1_prot_WP_011965296.1_1712) |  |  |  |  |  |  | + | [1.B.14.16.4](http://tcdb.org/search/result.php?tc=1.B.14.16.4) | -08 | D8EPC3 | Unknown | Unknown |
| WP_008767137.1 |  |  |  | + |  |  |  | 1.B.16.1.4 | -10 | Q2IMR5 | Carboxylates | Amides |
| WP_011107936.1 |  |  |  | + |  |  |  | 1.B.16.1.4 | -06 | Q2IMR5 | Carboxylates | Amides |
| [WP_007212214.1](file:///Volumes/ESD-USB/Summer%202018/Hassan%20Submission%20PLosOne/content.html#lcl|NZ_CP012801.1_prot_WP_007212214.1_4665) |  |  | + |  |  |  |  | [1.B.16.2.3](http://tcdb.org/search/result.php?tc=1.B.16.2.3) | -11 | B9M986 | Anions | PO_4_^3-^ |
| [WP_022209911.1](file:///Volumes/ESD-USB/Summer%202018/Hassan%20Submission%20PLosOne/content.html#lcl|NZ_CP012801.1_prot_WP_022209911.1_1209) |  |  | + |  |  |  |  | [1.B.16.2.3](http://tcdb.org/search/result.php?tc=1.B.16.2.3) | -08 | B9M986 | Anions | PO_4_^3-^ |
| ABI39_17930 | + |  |  |  |  |  |  | 1.B.17.2.5 | -17 | C9LSB9 | Unknown | Unknown |
| ABI39_01070 | + |  |  |  |  |  |  | 1.B.17.2.5 | -17 | C9LSB9 | Unknown | Unknown |
| ABI39_21005 | + |  |  |  |  |  |  | 1.B.17.2.5 | -16 | C9LSB9 | Unknown | Unknown |
| [WP_025725773.1](file:///Volumes/ESD-USB/Summer%202018/Hassan%20Submission%20PLosOne/content.html#lcl|NZ_CP012801.1_prot_WP_025725773.1_1943) |  |  | + |  |  |  |  | [1.B.17.2.5](http://tcdb.org/search/result.php?tc=1.B.17.2.5) | -20 | C9LSB9 | Unknown | Unknown |
| [WP_029426683.1](file:///Volumes/ESD-USB/Summer%202018/Hassan%20Submission%20PLosOne/content.html#lcl|NZ_CP012801.1_prot_WP_029426683.1_3293) |  |  | + |  |  |  |  | [1.B.17.2.5](http://tcdb.org/search/result.php?tc=1.B.17.2.5) | -17 | C9LSB9 | Unknown | Unknown |
| [lWP_029428352.1](file:///Volumes/ESD-USB/Summer%202018/Hassan%20Submission%20PLosOne/content.html#lcl|NZ_CP012801.1_prot_WP_029428352.1_1160) |  |  | + |  |  |  |  | [1.B.17.2.5](http://tcdb.org/search/result.php?tc=1.B.17.2.5) | -12 | C9LSB9 | Unknown | Unknown |
| [WP_029428308.1](file:///Volumes/ESD-USB/Summer%202018/Hassan%20Submission%20PLosOne/content.html#lcl|NZ_CP012801.1_prot_WP_029428308.1_1238) |  |  | + |  |  |  |  | [1.B.17.2.5](http://tcdb.org/search/result.php?tc=1.B.17.2.5) | -09 | C9LSB9 | Unknown | Unknown |
| YP_097840.1 |  |  |  |  | + |  |  | 1.B.17.2.5 | -22 | C9LSB9 | Molecules | Small molecules |
| YP_101260.1 |  |  |  |  | + |  |  | 1.B.17.2.5 | -14 | C9LSB9 | Molecules | Small molecules |
| YP_100591.1 |  |  |  |  | + |  |  | 1.B.17.2.5 | -12 | C9LSB9 | Molecules | Small molecules |
| YP_101791.1 |  |  |  |  | + |  |  | 1.B.17.2.5 | -11 | C9LSB9 | Molecules | Small molecules |
| [WP_012056004.1](file:///Volumes/ESD-USB/Summer%202018/Library/Application%20Support/Microsoft/Office/Office%202011%20AutoRecovery/content.html#lcl|NC_009614.1_prot_WP_012056004.1_3978) |  |  |  |  |  |  | + | [1.B.17.2.5](http://tcdb.org/search/result.php?tc=1.B.17.2.5) | -17 | C9LSB9 | Unknown | Unknown |
| [WP_005843625.1](file:///Volumes/ESD-USB/Summer%202018/Library/Application%20Support/Microsoft/Office/Office%202011%20AutoRecovery/content.html#lcl|NC_009614.1_prot_WP_005843625.1_198) |  |  |  |  |  |  | + | [1.B.17.2.5](http://tcdb.org/search/result.php?tc=1.B.17.2.5) | -17 | C9LSB9 | Unknown | Unknown |
| [WP_008669562.1](file:///Volumes/ESD-USB/Summer%202018/Library/Application%20Support/Microsoft/Office/Office%202011%20AutoRecovery/content.html#lcl|NC_009614.1_prot_WP_008669562.1_3496) |  |  |  |  |  |  | + | [1.B.17.2.5](http://tcdb.org/search/result.php?tc=1.B.17.2.5) | -15 | C9LSB9 | Unknown | Unknown |
| ABI39_01265 | + |  |  |  |  |  |  | 1.B.17.2.6 | -19 | Q1D4Q0 | Drugs | Macrolides |
| ABI39_09420 | + |  |  |  |  |  |  | 1.B.17.2.6 | -14 | Q1D4Q0 | Drugs | Macrolides |
| ABI39_01365 | + |  |  |  |  |  |  | 1.B.17.2.6 | -13 | Q1D4Q0 | Drugs | Macrolides |
| ABI39_21240 | + |  |  |  |  |  |  | 1.B.17.2.6 | -13 | Q1D4Q0 | Drugs | Macrolides |
| ABI39_05840 | + |  |  |  |  |  |  | 1.B.17.2.6 | -11 | Q1D4Q0 | Drugs | Macrolides |
| ABI39_17670 | + |  |  |  |  |  |  | 1.B.17.2.6 | -10 | Q1D4Q0 | Drugs | Macrolides |
| ABI39_10340 | + |  |  |  |  |  |  | 1.B.17.2.6 | -05 | Q1D4Q0 | Drugs | Macrolides |
| [WP_033160591.1](file:///Volumes/ESD-USB/Summer%202018/Hassan%20Submission%20PLosOne/content.html#lcl|NZ_CP012801.1_prot_WP_033160591.1_1559) |  |  | + |  |  |  |  | [1.B.17.2.6](http://tcdb.org/search/result.php?tc=1.B.17.2.6) | -17 | Q1D4Q0 | Drugs | Macrolides |
| [WP_007219635.1](file:///Volumes/ESD-USB/Summer%202018/Hassan%20Submission%20PLosOne/content.html#lcl|NZ_CP012801.1_prot_WP_007219635.1_3659) |  |  | + |  |  |  |  | [1.B.17.2.6](http://tcdb.org/search/result.php?tc=1.B.17.2.6) | -13 | Q1D4Q0 | Drugs | Macrolides |
| [WP_007210523.1](file:///Volumes/ESD-USB/Summer%202018/Hassan%20Submission%20PLosOne/content.html#lcl|NZ_CP012801.1_prot_WP_007210523.1_1719) |  |  | + |  |  |  |  | [1.B.17.2.6](http://tcdb.org/search/result.php?tc=1.B.17.2.6) | -13 | Q1D4Q0 | Drugs | Macrolides |
| [WP_022209306.1](file:///Volumes/ESD-USB/Summer%202018/Hassan%20Submission%20PLosOne/content.html#lcl|NZ_CP012801.1_prot_WP_022209306.1_324) |  |  | + |  |  |  |  | [1.B.17.2.6](http://tcdb.org/search/result.php?tc=1.B.17.2.6) | -11 | Q1D4Q0 | Drugs | Macrolides |
| WP_008762063.1 |  |  |  | + |  |  |  | 1.B.17.2.6 | -25 | Q1D4Q0 | Drugs | Macrolides |
| WP_011108315.1 |  |  |  | + |  |  |  | 1.B.17.2.6 | -20 | Q1D4Q0 | Drugs | Macrolides |
| WP_008764161.1 |  |  |  | + |  |  |  | 1.B.17.2.6 | -16 | Q1D4Q0 | Drugs | Macrolides |
| WP_008761968.1 |  |  |  | + |  |  |  | 1.B.17.2.6 | -15 | Q1D4Q0 | Drugs | Macrolides |
| WP_008766603.1 |  |  |  | + |  |  |  | 1.B.17.2.6 | -14 | Q1D4Q0 | Drugs | Macrolides |
| WP_062694899.1 |  |  |  | + |  |  |  | 1.B.17.2.6 | -12 | Q1D4Q0 | Drugs | Macrolides |
| WP_011107543.1 |  |  |  | + |  |  |  | 1.B.17.2.6 | -11 | Q1D4Q0 | Drugs | Macrolides |
| WP_062694823.1 |  |  |  | + |  |  |  | 1.B.17.2.6 | -10 | Q1D4Q0 | Drugs | Macrolides |
| WP_032840472.1 |  |  |  | + |  |  |  | 1.B.17.2.6 | -06 | Q1D4Q0 | Drugs | Macrolides |
| [WP_004296312.1](file:///Volumes/ESD-USB/Summer%202018/Hassan%20Submission%20PLosOne/content.html#lcl|NZ_CP012938.1_prot_WP_004296312.1_2614) |  |  |  |  |  | + |  | [1.B.17.2.6](http://tcdb.org/search/result.php?tc=1.B.17.2.6) | -23 | Q1D4Q0 | Unknown | Unknown |
| [WP_052587999.1](file:///Volumes/ESD-USB/Summer%202018/Hassan%20Submission%20PLosOne/content.html#lcl|NZ_CP012938.1_prot_WP_052587999.1_438) |  |  |  |  |  | + |  | [1.B.17.2.6](http://tcdb.org/search/result.php?tc=1.B.17.2.6) | -17 | Q1D4Q0 | Unknown | Unknown |
| [WP_004299384.1](file:///Volumes/ESD-USB/Summer%202018/Hassan%20Submission%20PLosOne/content.html#lcl|NZ_CP012938.1_prot_WP_004299384.1_2086) |  |  |  |  |  | + |  | [1.B.17.2.6](http://tcdb.org/search/result.php?tc=1.B.17.2.6) | -16 | Q1D4Q0 | Unknown | Unknown |
| [WP_004295999.1](file:///Volumes/ESD-USB/Summer%202018/Hassan%20Submission%20PLosOne/content.html#lcl|NZ_CP012938.1_prot_WP_004295999.1_687) |  |  |  |  |  | + |  | [1.B.17.2.6](http://tcdb.org/search/result.php?tc=1.B.17.2.6) | -12 | Q1D4Q0 | Unknown | Unknown |
| [WP_032855778.1](file:///Volumes/ESD-USB/Summer%202018/Hassan%20Submission%20PLosOne/content.html#lcl|NZ_CP012938.1_prot_WP_032855778.1_2221) |  |  |  |  |  | + |  | [1.B.17.2.6](http://tcdb.org/search/result.php?tc=1.B.17.2.6) | -06 | Q1D4Q0 | Unknown | Unknown |
| WP_062695905.1 |  |  |  | + |  |  |  | 1.B.17.3.5 | -47 | P77211 | Cations | Cu^2+^/ Ag^+^ |
| WP_055229599.1 |  |  |  | + |  |  |  | 1.B.18.1.2 | -18 | Q03961 | Sugar derivatives | Polysialic acid |
| WP_062694853.1 |  |  |  | + |  |  |  | 1.B.18.1.2 | -18 | Q03961 | Sugar derivatives | Polysialic acid |
| WP_062694753.1 |  |  |  | + |  |  |  | 1.B.18.1.2 | -18 | Q03961 | Sugar derivatives | Polysialic acid |
| ABI39_13415 | + |  |  |  |  |  |  | 1.B.18.3.3 | -36 | A5FN23 | unknown | unknown |
| ABI39_11140 | + |  |  |  |  |  |  | 1.B.18.3.3 | -28 | A5FN23 | unknown | unknown |
| [WP_013616312.1](file:///Volumes/ESD-USB/Summer%202018/Hassan%20Submission%20PLosOne/content.html#lcl|NC_015164.1_prot_WP_013616312.1_236) |  | + |  |  |  |  |  | [1.B.18.3.3](http://tcdb.org/search/result.php?tc=1.B.18.3.3) | -30 | A5FN23 | Sugars | Polysaccharides |
| [WP_013618645.1](file:///Volumes/ESD-USB/Summer%202018/Hassan%20Submission%20PLosOne/content.html#lcl|NC_015164.1_prot_WP_013618645.1_2684) |  | + |  |  |  |  |  | [1.B.18.3.3](http://tcdb.org/search/result.php?tc=1.B.18.3.3) | -29 | A5FN23 | Sugars | Polysaccharides |
| WP_008766005.1 |  |  |  | + |  |  |  | 1.B.18.3.3 | -32 | A5FN23 | Unknown | Unknown |
| WP_062695184.1 |  |  |  | + |  |  |  | 1.B.18.3.3 | -31 | A5FN23 | Unknown | Unknown |
| WP_062695929.1 |  |  |  | + |  |  |  | 1.B.18.3.3 | -30 | A5FN23 | Unknown | Unknown |
| YP_100052.1 |  |  |  |  | + |  |  | 1.B.18.3.3 | -36 | A5FN23 | Sugars | Polysaccharides |
| [WP_004297470.1](file:///Volumes/ESD-USB/Summer%202018/Hassan%20Submission%20PLosOne/content.html#lcl|NZ_CP012938.1_prot_WP_004297470.1_215) |  |  |  |  |  | + |  | [1.B.18.3.3](http://tcdb.org/search/result.php?tc=1.B.18.3.3) | -31 | A5FN23 | Unknown | Unknown |
| [WP_052587988.1](file:///Volumes/ESD-USB/Summer%202018/Hassan%20Submission%20PLosOne/content.html#lcl|NZ_CP012938.1_prot_WP_052587988.1_1085) |  |  |  |  |  | + |  | [1.B.18.3.3](http://tcdb.org/search/result.php?tc=1.B.18.3.3) | -31 | A5FN23 | Unknown | Unknown |
| [WP_004297482.1](file:///Volumes/ESD-USB/Summer%202018/Hassan%20Submission%20PLosOne/content.html#lcl|NZ_CP012938.1_prot_WP_004297482.1_222) |  |  |  |  |  | + |  | [1.B.18.3.3](http://tcdb.org/search/result.php?tc=1.B.18.3.3) | -30 | A5FN23 | Unknown | Unknown |
| [WP_004300023.1](file:///Volumes/ESD-USB/Summer%202018/Hassan%20Submission%20PLosOne/content.html#lcl|NZ_CP012938.1_prot_WP_004300023.1_1123) |  |  |  |  |  | + |  | [1.B.18.3.3](http://tcdb.org/search/result.php?tc=1.B.18.3.3) | -29 | A5FN23 | Unknown | Unknown |
| [WP_050428273.1](file:///Volumes/ESD-USB/Summer%202018/Hassan%20Submission%20PLosOne/content.html#lcl|NZ_CP012938.1_prot_WP_050428273.1_847) |  |  |  |  |  | + |  | [1.B.18.3.3](http://tcdb.org/search/result.php?tc=1.B.18.3.3) | -29 | A5FN23 | Unknown | Unknown |
| [WP_005848112.1](file:///Volumes/ESD-USB/Summer%202018/Library/Application%20Support/Microsoft/Office/Office%202011%20AutoRecovery/content.html#lcl|NC_009614.1_prot_WP_005848112.1_2624) |  |  |  |  |  |  | + | [1.B.18.3.3](http://tcdb.org/search/result.php?tc=1.B.18.3.3) | -35 | A5FN23 | Unknown | Unknown |
| [WP_005636845.1](file:///Volumes/ESD-USB/Summer%202018/Library/Application%20Support/Microsoft/Office/Office%202011%20AutoRecovery/content.html#lcl|NC_009614.1_prot_WP_005636845.1_1519) |  |  |  |  |  |  | + | [1.B.18.3.3](http://tcdb.org/search/result.php?tc=1.B.18.3.3) | -29 | A5FN23 | Unknown | Unknown |
| [WP_005840559.1](file:///Volumes/ESD-USB/Summer%202018/Library/Application%20Support/Microsoft/Office/Office%202011%20AutoRecovery/content.html#lcl|NC_009614.1_prot_WP_005840559.1_2341) |  |  |  |  |  |  | + | [1.B.18.3.3](http://tcdb.org/search/result.php?tc=1.B.18.3.3) | -28 | A5FN23 | Unknown | Unknown |
| [WP_007219036.1](file:///Volumes/ESD-USB/Summer%202018/Library/Application%20Support/Microsoft/Office/Office%202011%20AutoRecovery/content.html#lcl|NC_009614.1_prot_WP_007219036.1_697) |  |  |  |  |  |  | + | [1.B.18.3.3](http://tcdb.org/search/result.php?tc=1.B.18.3.3) | -28 | A5FN23 | Unknown | Unknown |
| ABI39_07050 | + |  |  |  |  |  |  | 1.B.33.1.1 | -42 | Q9K1H0 | Unknown | Unknown |
| ABI39_15410 | + |  |  |  |  |  |  | 1.B.33.1.1 | -06 | Q9K1H0 | Unknown | Unknown |
| [WP_013618071.1](file:///Volumes/ESD-USB/Summer%202018/Hassan%20Submission%20PLosOne/content.html#lcl|NC_015164.1_prot_WP_013618071.1_2048) |  | + |  |  |  |  |  | [1.B.33.1.1](http://tcdb.org/search/result.php?tc=1.B.33.1.1) | -37 | Q9K1H0 | Unknown | Unknown |
| [WP_041584119.1](file:///Volumes/ESD-USB/Summer%202018/Hassan%20Submission%20PLosOne/content.html#lcl|NC_015164.1_prot_WP_041584119.1_3520) |  | + |  |  |  |  |  | [1.B.33.1.1](http://tcdb.org/search/result.php?tc=1.B.33.1.1) | -07 | Q9K1H0 | Unknown | Unknown |
| WP_007214119.1 |  |  | + |  |  |  |  | 1.B.33.1.1 | -08 | Q9K1H0 | Unknown | Unknown |
| WP_007209618.1 |  |  | + |  |  |  |  | 1.B.33.1.1 | -05 | Q9K1H0 | Unknown | Unknown |
| WP_008766990.1 |  |  |  | + |  |  |  | 1.B.33.1.1 | -32 | Q9K1H0 | Unknown | Unknown |
| WP_062695073.1 |  |  |  | + |  |  |  | 1.B.33.1.1 | -07 | Q9K1H0 | Unknown | Unknown |
| [WP_041174474.1](file:///Volumes/ESD-USB/Summer%202018/Library/Application%20Support/Microsoft/Office/Office%202011%20AutoRecovery/content.html#lcl|NC_009614.1_prot_WP_041174474.1_3009) |  |  |  |  |  |  | + | [1.B.33.1.1](http://tcdb.org/search/result.php?tc=1.B.33.1.1) | -06 | Q9K1H0 | Unknown | Unknown |
| WP_029426052.1 |  |  | + |  |  |  |  | 1.B.33.1.3 | -16 | P77774 | Unknown | Unknown |
| WP_025725687.1 |  |  | + |  |  |  |  | 1.B.33.1.3 | -14 | P77774 | Unknown | Unknown |
| ABI39_02175 | + |  |  |  |  |  |  | 1.B.33.1.4 | -05 | Q9A7R7 | Unknown | Unknown |
| ABI39_02055 | + |  |  |  |  |  |  | 1.B.33.2.3 | -09 | C9LSC3 | Unknown | Unknown |
| [WP_004300383.1](file:///Volumes/ESD-USB/Summer%202018/Hassan%20Submission%20PLosOne/content.html#lcl|NZ_CP012938.1_prot_WP_004300383.1_483) |  |  |  |  |  | + |  | [1.B.33.2.3](http://tcdb.org/search/result.php?tc=1.B.33.2.3) | -06 | C9LSC3 | Unknown | Unknown |
| YP_101384.1 |  |  |  |  | + |  |  | 1.B.33.2.4 | -05 | P0ADE4 | Proteins | Proteins |
| [WP_004299936.1](file:///Volumes/ESD-USB/Summer%202018/Hassan%20Submission%20PLosOne/content.html#lcl|NZ_CP012938.1_prot_WP_004299936.1_3771) |  |  |  |  |  | + |  | [1.B.33.2.4](http://tcdb.org/search/result.php?tc=1.B.33.2.4) | -09 | P0ADE4 | Unknown | Unknown |
| [WP_013618965.1](file:///Volumes/ESD-USB/Summer%202018/Hassan%20Submission%20PLosOne/content.html#lcl|NC_015164.1_prot_WP_013618965.1_3005) |  | + |  |  |  |  |  | [1.B.42.1.2](http://tcdb.org/search/result.php?tc=1.B.42.1.2) | -83 | P0A9V1 | Sugars | Lipopolysaccharide |
| [WP_005852797.1](file:///Volumes/ESD-USB/Summer%202018/Library/Application%20Support/Microsoft/Office/Office%202011%20AutoRecovery/content.html#lcl|NC_009614.1_prot_WP_005852797.1_445) |  |  |  |  |  |  | + | [1.B.42.1.2](http://tcdb.org/search/result.php?tc=1.B.42.1.2) | -81 | P0A9V1 | Unknown | Unknown |
| [WP_013616129.1](file:///Volumes/ESD-USB/Summer%202018/Hassan%20Submission%20PLosOne/content.html#lcl|NC_015164.1_prot_WP_013616129.1_51) |  | + |  |  |  |  |  | [1.B.42.1.8](http://tcdb.org/search/result.php?tc=1.B.42.1.8) | -126 | L7WGX6 | Sugars | Lipopolysaccharide |
| WP_062694577.1 |  |  |  | + |  |  |  | 1.B.42.1.8 | -130 | L7WGX6 | Unknown | Unknown |
| [WP_008671498.1](file:///Volumes/ESD-USB/Summer%202018/Library/Application%20Support/Microsoft/Office/Office%202011%20AutoRecovery/content.html#lcl|NC_009614.1_prot_WP_008671498.1_4050) |  |  |  |  |  |  | + | [1.B.42.1.8](http://tcdb.org/search/result.php?tc=1.B.42.1.8) | -117 | L7WGX6 | Sugar derivatives | Lipopolysaccharides |
| [WP_029429098.1](file:///Volumes/ESD-USB/Summer%202018/Hassan%20Submission%20PLosOne/content.html#lcl|NZ_CP012801.1_prot_WP_029429098.1_1849) |  |  | + |  |  |  |  | [1.B.42.1.13](http://tcdb.org/search/result.php?tc=1.B.42.1.13) | -09 | B8CYZ1 | Lipids | Lipopolysaccharides |
| WP_061473265.1 |  |  |  | + |  |  |  | 1.B.42.1.13 | -08 | B8CYZ1 | Unknown | Unknown |
| WP_008763745.1 |  |  |  | + |  |  |  | 1.B.42.1.13 | -06 | B8CYZ1 | Unknown | Unknown |
| [WP_025725828.1](file:///Volumes/ESD-USB/Summer%202018/Hassan%20Submission%20PLosOne/content.html#lcl|NZ_CP012801.1_prot_WP_025725828.1_1845) |  |  | + |  |  |  |  | [1.B.44.1.2](http://tcdb.org/search/result.php?tc=1.B.44.1.2) | -70 | E6K3Q6 | Unknown | Unknown |
| WP_008760767.1 |  |  |  | + |  |  |  | 1.B.42.1.2 | -82 | P0A9V1 | Sugars | Lipopolysaccharides |
| WP_062694563.1 |  |  |  | + |  |  |  | 1.B.42.1.2 | -18 | P0A9V1 | Sugars | Lipopolysaccharides |
| [WP_004297152.1](file:///Volumes/ESD-USB/Summer%202018/Hassan%20Submission%20PLosOne/content.html#lcl|NZ_CP012938.1_prot_WP_004297152.1_3794) |  |  |  |  |  | + |  | [1.B.42.1.2](http://tcdb.org/search/result.php?tc=1.B.42.1.2) | -82 | P0A9V1 | Unknown | Unknown |
| [WP_004296378.1](file:///Volumes/ESD-USB/Summer%202018/Hassan%20Submission%20PLosOne/content.html#lcl|NZ_CP012938.1_prot_WP_004296378.1_2680) |  |  |  |  |  | + |  | [1.B.44.2.1](http://tcdb.org/search/result.php?tc=1.B.44.2.1) | -16 | A5FNR0 | Unknown | Unknown |
| [WP_012055603.1](file:///Volumes/ESD-USB/Summer%202018/Library/Application%20Support/Microsoft/Office/Office%202011%20AutoRecovery/content.html#lcl|NC_009614.1_prot_WP_012055603.1_3149) |  |  |  |  |  |  | + | [1.B.44.2.1](http://tcdb.org/search/result.php?tc=1.B.44.2.1) | -09 | A5FNR0 | Unknown | Unknown |
| [WP_004295789.1](file:///Volumes/ESD-USB/Summer%202018/Hassan%20Submission%20PLosOne/content.html#lcl|NZ_CP012938.1_prot_WP_004295789.1_871) |  |  |  |  |  | + |  | [1.B.44.2.3](http://tcdb.org/search/result.php?tc=1.B.44.2.3) | -09 | L7WBF8 | Unknown | Unknown |
| [WP_004296220.1](file:///Volumes/ESD-USB/Summer%202018/Hassan%20Submission%20PLosOne/content.html#lcl|NZ_CP012938.1_prot_WP_004296220.1_2538) |  |  |  |  |  | + |  | [1.B.44.2.9](http://tcdb.org/search/result.php?tc=1.B.44.2.9) | -14 | I3YP34 | Unknown | Unknown |
| ABI39_07385 | + |  |  |  |  |  |  | 1.B.44.2.11 | -06 | F0R8C3 | Unknown | Unknown |
| ABI39_05490 | + |  |  |  |  |  |  | 1.B.44.2.11 | -05 | F0R8C3 | Unknown | Unknown |
| [WP_013619462.1](file:///Volumes/ESD-USB/Summer%202018/Hassan%20Submission%20PLosOne/content.html#lcl|NC_015164.1_prot_WP_013619462.1_3509) |  | + |  |  |  |  |  | [1.B.44.2.11](http://tcdb.org/search/result.php?tc=1.B.44.2.11) | -149 | F0R8C3 | Unknown | Unknown |
| [WP_013618808.1](file:///Volumes/ESD-USB/Summer%202018/Hassan%20Submission%20PLosOne/content.html#lcl|NC_015164.1_prot_WP_013618808.1_2849) |  | + |  |  |  |  |  | [1.B.44.2.11](http://tcdb.org/search/result.php?tc=1.B.44.2.11) | -69 | F0R8C3 | Unknown | Unknown |
| [WP_022209002.1](file:///Volumes/ESD-USB/Summer%202018/Hassan%20Submission%20PLosOne/content.html#lcl|NZ_CP012801.1_prot_WP_022209002.1_2192) |  |  | + |  |  |  |  | [1.B.44.2.11](http://tcdb.org/search/result.php?tc=1.B.44.2.11) | -22 | F0R8C3 | Unknown | Unknown |
| [WP_022210508.1](file:///Volumes/ESD-USB/Summer%202018/Hassan%20Submission%20PLosOne/content.html#lcl|NZ_CP012801.1_prot_WP_022210508.1_3718) |  |  | + |  |  |  |  | [1.B.44.2.11](http://tcdb.org/search/result.php?tc=1.B.44.2.11) | -14 | F0R8C3 | Unknown | Unknown |
| [WP_007213914.1](file:///Volumes/ESD-USB/Summer%202018/Hassan%20Submission%20PLosOne/content.html#lcl|NZ_CP012801.1_prot_WP_007213914.1_353) |  |  | + |  |  |  |  | [1.B.44.2.11](http://tcdb.org/search/result.php?tc=1.B.44.2.11) | -12 | F0R8C3 | Unknown | Unknown |
| [WP_007214465.1](file:///Volumes/ESD-USB/Summer%202018/Hassan%20Submission%20PLosOne/content.html#lcl|NZ_CP012801.1_prot_WP_007214465.1_972) |  |  | + |  |  |  |  | [1.B.44.2.11](http://tcdb.org/search/result.php?tc=1.B.44.2.11) | -11 | F0R8C3 | Unknown | Unknown |
| [WP_022210713.1](file:///Volumes/ESD-USB/Summer%202018/Hassan%20Submission%20PLosOne/content.html#lcl|NZ_CP012801.1_prot_WP_022210713.1_971) |  |  | + |  |  |  |  | [1.B.44.2.11](http://tcdb.org/search/result.php?tc=1.B.44.2.11) | -09 | F0R8C3 | Unknown | Unknown |
| WP_008764158.1 |  |  |  | + |  |  |  | 1.B.44.2.11 | -46 | F0R8C3 | Unknown | Unknown |
| WP_055218186.1 |  |  |  | + |  |  |  | 1.B.44.2.11 | -27 | F0R8C3 | Unknown | Unknown |
| WP_008762074.1 |  |  |  | + |  |  |  | 1.B.44.2.11 | -05 | F0R8C3 | Unknown | Unknown |
| [WP_004314733.1](file:///Volumes/ESD-USB/Summer%202018/Hassan%20Submission%20PLosOne/content.html#lcl|NZ_CP012938.1_prot_WP_004314733.1_3849) |  |  |  |  |  | + |  | [1.B.44.2.11](http://tcdb.org/search/result.php?tc=1.B.44.2.11) | -42 | F0R8C3 | Unknown | Unknown |
| [WP_004295701.1](file:///Volumes/ESD-USB/Summer%202018/Hassan%20Submission%20PLosOne/content.html#lcl|NZ_CP012938.1_prot_WP_004295701.1_947) |  |  |  |  |  | + |  | [1.B.44.2.11](http://tcdb.org/search/result.php?tc=1.B.44.2.11) | -38 | F0R8C3 | Unknown | Unknown |
| [WP_004296878.1](file:///Volumes/ESD-USB/Summer%202018/Hassan%20Submission%20PLosOne/content.html#lcl|NZ_CP012938.1_prot_WP_004296878.1_3163) |  |  |  |  |  | + |  | [1.B.44.2.11](http://tcdb.org/search/result.php?tc=1.B.44.2.11) | -05 | F0R8C3 | Unknown | Unknown |
| [WP_008669072.1](file:///Volumes/ESD-USB/Summer%202018/Library/Application%20Support/Microsoft/Office/Office%202011%20AutoRecovery/content.html#lcl|NC_009614.1_prot_WP_008669072.1_3151) |  |  |  |  |  |  | + | [1.B.44.2.11](http://tcdb.org/search/result.php?tc=1.B.44.2.11) | -22 | F0R8C3 | Unknown | Unknown |
| [WP_011965265.1](file:///Volumes/ESD-USB/Summer%202018/Library/Application%20Support/Microsoft/Office/Office%202011%20AutoRecovery/content.html#lcl|NC_009614.1_prot_WP_011965265.1_1677) |  |  |  |  |  |  | + | [1.B.44.2.11](http://tcdb.org/search/result.php?tc=1.B.44.2.11) | -20 | F0R8C3 | Unknown | Unknown |
| YP_099898.1 |  |  |  |  | + |  |  | 1.B.44.2.12 | -09 | D0LVA6 | Unknown | Unknown |
| [WP_005846633.1](file:///Volumes/ESD-USB/Summer%202018/Library/Application%20Support/Microsoft/Office/Office%202011%20AutoRecovery/content.html#lcl|NC_009614.1_prot_WP_005846633.1_2988) |  |  |  |  |  |  | + | [1.B.44.2.12](http://tcdb.org/search/result.php?tc=1.B.44.2.12) | -14 | D0LVA6 | Unknown | Unknown |
| [WP_013617871.1](file:///Volumes/ESD-USB/Summer%202018/Hassan%20Submission%20PLosOne/content.html#lcl|NC_015164.1_prot_WP_013617871.1_1844) |  | + |  |  |  |  |  | [1.B.48.2.5](http://tcdb.org/search/result.php?tc=1.B.48.2.5) | -17 | D6CYH4 | Unknown | Unknown |
| [WP_007210930.1](file:///Volumes/ESD-USB/Summer%202018/Hassan%20Submission%20PLosOne/content.html#lcl|NZ_CP012801.1_prot_WP_007210930.1_2304) |  |  | + |  |  |  |  | [1.B.48.2.5](http://tcdb.org/search/result.php?tc=1.B.48.2.5) | -88 | D6CYH4 | Unknown | Unknown |
| [WP_004298503.1](file:///Volumes/ESD-USB/Summer%202018/Hassan%20Submission%20PLosOne/content.html#lcl|NZ_CP012938.1_prot_WP_004298503.1_2946) |  |  |  |  |  | + |  | [1.B.48.2.5](http://tcdb.org/search/result.php?tc=1.B.48.2.5) | 0 | D6CYH4 | Unknown | Unknown |
| [WP_008666620.1](file:///Volumes/ESD-USB/Summer%202018/Library/Application%20Support/Microsoft/Office/Office%202011%20AutoRecovery/content.html#lcl|NC_009614.1_prot_WP_008666620.1_1019) |  |  |  |  |  |  | + | [1.B.48.2.5](http://tcdb.org/search/result.php?tc=1.B.48.2.5) | -24 | D6CYH4 | Unknown | Unknown |
| WP_008766733.1 |  |  |  | + |  |  |  | 1.B.54.1.1 | -05 | P43261 | Proteins | Intimins/Invasins |
| WP_008765856.1 |  |  |  | + |  |  |  | 1.B.57.4.3 | -44 | F9Z6A9 | Unknown | Unknown |
| [WP_007210479.1](file:///Volumes/ESD-USB/Summer%202018/Hassan%20Submission%20PLosOne/content.html#lcl|NZ_CP012801.1_prot_WP_007210479.1_1775) |  |  | + |  |  |  |  | [1.B.57.4.6](http://tcdb.org/search/result.php?tc=1.B.57.4.6) | 0 | D9RR74 | Unknown | Unknown |
| [WP_013619278.1](file:///Volumes/ESD-USB/Summer%202018/Hassan%20Submission%20PLosOne/content.html#lcl|NC_015164.1_prot_WP_013619278.1_3330) |  | + |  |  |  |  |  | [1.B.70.1.1](http://tcdb.org/search/result.php?tc=1.B.70.1.1) | -07 | D0SAV4 | Unknown | Unknown |
| [WP_029427588.1](file:///Volumes/ESD-USB/Summer%202018/Hassan%20Submission%20PLosOne/content.html#lcl|NZ_CP012801.1_prot_WP_029427588.1_4600) |  |  | + |  |  |  |  | [1.B.70.1.6](http://tcdb.org/search/result.php?tc=1.B.70.1.6) | -25 | B2FIM5 | Unknown | Unknown |
| [WP_022208851.1](file:///Volumes/ESD-USB/Summer%202018/Hassan%20Submission%20PLosOne/content.html#lcl|NZ_CP012801.1_prot_WP_022208851.1_1909) |  |  | + |  |  |  |  | [1.B.71.1.6](http://tcdb.org/search/result.php?tc=1.B.71.1.6) | -07 | M1PDZ7 | Unknown | Unknown |
| WP_008764091.1 |  |  |  | + |  |  |  | 1.B.71.1.6 | -06 | M1PDZ7 | Unknown | Unknown |
| [WP_029428297.1](file:///Volumes/ESD-USB/Summer%202018/Hassan%20Submission%20PLosOne/content.html#lcl|NZ_CP012801.1_prot_WP_029428297.1_1267) |  |  | + |  |  |  |  | [1.C.39.13.1](http://tcdb.org/search/result.php?tc=1.C.39.13.1) | -59 | Q8A335 | Unknown | Unknown |
| [WP_033160337.1](file:///Volumes/ESD-USB/Summer%202018/Hassan%20Submission%20PLosOne/content.html#lcl|NZ_CP012801.1_prot_WP_033160337.1_2330) |  |  | + |  |  |  |  | [1.C.39.13.1](http://tcdb.org/search/result.php?tc=1.C.39.13.1) | -44 | Q8A335 | Unknown | Unknown |
| WP_008767383.1 |  |  |  | + |  |  |  | 1.C.39.13.1 | 0 | Q8A335 | Unknown | Unknown |
| ABI39_06200 | + |  |  |  |  |  |  | 1.C.39.13.2 | -42 | Q64VU4 | Unknown | Unknown |
| ABI39_06205 | + |  |  |  |  |  |  | 1.C.39.13.2 | -43 | Q64VU4 | Unknown | Unknown |
| YP_098916.1 |  |  |  |  | + |  |  | 1.C.39.13.2 | 0 | Q64VU4 | Unknown | Unknown |
| [WP_022208367.1](file:///Volumes/ESD-USB/Summer%202018/Hassan%20Submission%20PLosOne/content.html#lcl|NZ_CP012801.1_prot_WP_022208367.1_2954) |  |  | + |  |  |  |  | [1.C.39.13.3](http://tcdb.org/search/result.php?tc=1.C.39.13.3) | -41 | Q64W10 | Unknown | Unknown |
| YP_098850.1 |  |  |  |  | + |  |  | 1.C.39.13.3 | 0 | Q64W10 | Unknown | Unknown |
| YP_099969.1 |  |  |  |  | + |  |  | 1.C.39.13.3 | -39 | Q64W10 | Unknown | Unknown |
| ABI39_03780 | + |  |  |  |  |  |  | 1.C.82.1.1 | -75 | Q9ZK21 | Unknown | Unknown |
| [WP_013618476.1](file:///Volumes/ESD-USB/Summer%202018/Hassan%20Submission%20PLosOne/content.html#lcl|NC_015164.1_prot_WP_013618476.1_2471) |  | + |  |  |  |  |  | [1.C.82.1.1](http://tcdb.org/search/result.php?tc=1.C.82.1.1) | -75 | Q9ZK21 | Unknown | Unknown |
| [WP_007219563.1](file:///Volumes/ESD-USB/Summer%202018/Hassan%20Submission%20PLosOne/content.html#lcl|NZ_CP012801.1_prot_WP_007219563.1_3245) |  |  | + |  |  |  |  | [1.C.82.1.1](http://tcdb.org/search/result.php?tc=1.C.82.1.1) | -74 | Q9ZK21 | Unknown | Unknown |
| WP_004310894.1 |  |  |  | + |  |  |  | 1.C.82.1.1 | -75 | Q9ZK21 | Unknown | Unknown |
| WP_004296360.1 |  |  |  |  |  | + |  | [1.C.82.1.1](http://tcdb.org/search/result.php?tc=1.C.82.1.1) | -75 | Q9ZK21 | Unknown | Unknown |
| [WP_005844828.1](file:///Volumes/ESD-USB/Summer%202018/Library/Application%20Support/Microsoft/Office/Office%202011%20AutoRecovery/content.html#lcl|NC_009614.1_prot_WP_005844828.1_809) |  |  |  |  |  |  | + | [1.C.82.1.1](http://tcdb.org/search/result.php?tc=1.C.82.1.1) | -75 | Q9ZK21 | Unknown | Unknown |
| [WP_049764443.](file:///Volumes/ESD-USB/Summer%202018/Library/Application%20Support/Microsoft/Office/Office%202011%20AutoRecovery/content.html#lcl|NC_009614.1_prot_WP_049764443.1_2406) 1 |  |  |  |  |  |  | + | [1.C.102.1.2](http://tcdb.org/search/result.php?tc=1.C.102.1.2) | -20 | C6SS71 | Peptides | Bacteriocins |
| ABI39_08190 | + |  |  |  |  |  |  | 1.C.113.1.1 | -32 | P54176 | Unknown | Unknown |
| WP_032850333.1 |  |  |  | + |  |  |  | 1.C.113.1.1 | -35 | P54176 | Unknown | Unknown |
| YP_100968.1 |  |  |  |  | + |  |  | 1.C.113.1.1 | -32 | P54176 | Molecules | Small molecules |
| [WP_004297979.](file:///Volumes/ESD-USB/Summer%202018/Hassan%20Submission%20PLosOne/content.html#lcl|NZ_CP012938.1_prot_WP_004297979.1_1968) 1 |  |  |  |  |  | + |  | [1.C.113.1.1](http://tcdb.org/search/result.php?tc=1.C.113.1.1) | -37 | P54176 | Unknown | Unknown |
| [WP_005838692.1](file:///Volumes/ESD-USB/Summer%202018/Library/Application%20Support/Microsoft/Office/Office%202011%20AutoRecovery/content.html#lcl|NC_009614.1_prot_WP_005838692.1_1640) |  |  |  |  |  |  | + | [1.C.113.1.1](http://tcdb.org/search/result.php?tc=1.C.113.1.1) | -33 | P54176 | Unknown | Unknown |
| [WP_007217932.1](file:///Volumes/ESD-USB/Summer%202018/Hassan%20Submission%20PLosOne/content.html#lcl|NZ_CP012801.1_prot_WP_007217932.1_554) |  |  | + |  |  |  |  | [1.C.113.1.3](http://tcdb.org/search/result.php?tc=1.C.113.1.3) | -34 | A4VGV1 | Unknown | Unknown |
| ABI39_02730 | + |  |  |  |  |  |  | 1.E.14.1.13 | -34 | C7M4K8 | Unknown | Unknown |
| [WP_013617721.1](file:///Volumes/ESD-USB/Summer%202018/Hassan%20Submission%20PLosOne/content.html#lcl|NC_015164.1_prot_WP_013617721.1_1691) |  | + |  |  |  |  |  | [1.E.14.1.13](http://tcdb.org/search/result.php?tc=1.E.14.1.13) | -30 | C7M4K8 | Unknown | Unknown |
| [WP_007210661.1](file:///Volumes/ESD-USB/Summer%202018/Hassan%20Submission%20PLosOne/content.html#lcl|NZ_CP012801.1_prot_WP_007210661.1_2077) |  |  | + |  |  |  |  | [1.E.14.1.13](http://tcdb.org/search/result.php?tc=1.E.14.1.13) | -34 | C7M4K8 | Unknown | Unknown |
| WP_022472045.1 |  |  |  | + |  |  |  | 1.E.14.1.13 | -34 | C7M4K8 | Unknown | Unknown |
| YP_097757.1 |  |  |  |  | + |  |  | 1.E.14.1.13 | -33 | C7M4K8 | Unknown | Unknown |
| [WP_004299736.1](file:///Volumes/ESD-USB/Summer%202018/Hassan%20Submission%20PLosOne/content.html#lcl|NZ_CP012938.1_prot_WP_004299736.1_3628) |  |  |  |  |  | + |  | [1.E.14.1.13](http://tcdb.org/search/result.php?tc=1.E.14.1.13) | -33 | C7M4K8 | Unknown | Unknown |
| [WP_005840692.1](file:///Volumes/ESD-USB/Summer%202018/Library/Application%20Support/Microsoft/Office/Office%202011%20AutoRecovery/content.html#lcl|NC_009614.1_prot_WP_005840692.1_515) |  |  |  |  |  |  | + | [1.E.14.1.13](http://tcdb.org/search/result.php?tc=1.E.14.1.13) | -34 | C7M4K8 | Unknown | Unknown |
| [WP_013617685.1](file:///Volumes/ESD-USB/Summer%202018/Hassan%20Submission%20PLosOne/content.html#lcl|NC_015164.1_prot_WP_013617685.1_1654) |  | + |  |  |  |  |  | [1.E.34.4.2](http://tcdb.org/search/result.php?tc=1.E.34.4.2) | -17 | E0NRR4 | Unknown | Unknown |
| [WP_025726505.1](file:///Volumes/ESD-USB/Summer%202018/Hassan%20Submission%20PLosOne/content.html#lcl|NZ_CP012801.1_prot_WP_025726505.1_860) |  |  | + |  |  |  |  | [1.E.34.4.2](http://tcdb.org/search/result.php?tc=1.E.34.4.2) | -20 | E0NRR4 | Unknown | Unknown |
| WP_008760687.1 |  |  |  | + |  |  |  | 1.E.34.4.2 | -19 | E0NRR4 | Unknown | Unknown |
| ABI39_20975 | + |  |  |  |  |  |  | 1.E.34.4.2 | -23 | E0NRR4 | Unknown | Unknown |
| [WP_004297511.1](file:///Volumes/ESD-USB/Summer%202018/Hassan%20Submission%20PLosOne/content.html#lcl|NZ_CP012938.1_prot_WP_004297511.1_248) |  |  |  |  |  | + |  | [1.E.34.4.2](http://tcdb.org/search/result.php?tc=1.E.34.4.2) | -18 | E0NRR4 | Unknown | Unknown |
| [WP_005847087.1](file:///Volumes/ESD-USB/Summer%202018/Library/Application%20Support/Microsoft/Office/Office%202011%20AutoRecovery/content.html#lcl|NC_009614.1_prot_WP_005847087.1_3972) |  |  |  |  |  |  | + | [1.E.34.4.2](http://tcdb.org/search/result.php?tc=1.E.34.4.2) | -22 | E0NRR4 | Unknown | Unknown |
| ABI39_18015 | + |  |  |  |  |  |  | 1.E.43.1.10 | -46 | I9U3B8 | Unknown | Unknown |
| WP_008764857.1 |  |  |  | + |  |  |  | 1.E.43.1.10 | -20 | I9U3B8 | Unknown | Unknown |
| YP_101465.1 |  |  |  |  | + |  |  | 1.E.43.1.10 | -19 | I9U3B8 | Unknown | Unknown |
| [WP_004297125.1](file:///Volumes/ESD-USB/Summer%202018/Hassan%20Submission%20PLosOne/content.html#lcl|NZ_CP012938.1_prot_WP_004297125.1_1421) |  |  |  |  |  | + |  | [1.E.43.1.10](http://tcdb.org/search/result.php?tc=1.E.43.1.10) | -20 | I9U3B8 | Unknown | Unknown |
| [WP_005839298.1](file:///Volumes/ESD-USB/Summer%202018/Library/Application%20Support/Microsoft/Office/Office%202011%20AutoRecovery/content.html#lcl|NC_009614.1_prot_WP_005839298.1_3513) |  |  |  |  |  |  | + | [1.E.43.1.10](http://tcdb.org/search/result.php?tc=1.E.43.1.10) | -47 | I9U3B8 | Unknown | Unknown |
| WP_008761998.1 |  |  |  | + |  |  |  | 1.E.43.3.3 | -21 | A0ZK04 | Amino acids | Serine/Threonine |
| [WP_029426725.1](file:///Volumes/ESD-USB/Summer%202018/Hassan%20Submission%20PLosOne/content.html#lcl|NZ_CP012801.1_prot_WP_029426725.1_4814) |  |  | + |  |  |  |  | [1.E.43.4.1](http://tcdb.org/search/result.php?tc=1.E.43.4.1) | -27 | L7WG79 | Unknown | Unknown |
| WP_008759611.1 |  |  |  | + |  |  |  | 1.E.43.4.1 | -28 | L7WG79 | Unknown | Unknown |
| YP_097996.1 |  |  |  |  | + |  |  | 1.E.43.4.1 | -35 | L7WG79 | Unknown | Unknown |
| [WP_004299251.1](file:///Volumes/ESD-USB/Summer%202018/Hassan%20Submission%20PLosOne/content.html#lcl|NZ_CP012938.1_prot_WP_004299251.1_2178) |  |  |  |  |  | + |  | [1.E.43.4.1](http://tcdb.org/search/result.php?tc=1.E.43.4.1) | -27 | L7WG79 | Unknown | Unknown |
| ABI39_20990 | + |  |  |  |  |  |  | 2.A.1.1.3 | -158 | P0AGF4 | Sugars | Xylose |
| ABI39_03480 | + |  |  |  |  |  |  | 2.A.1.1.3 | -96 | P0AGF4 | Sugars | Xylose |
| [WP_013618898.1](file:///Volumes/ESD-USB/Summer%202018/Hassan%20Submission%20PLosOne/content.html#lcl|NC_015164.1_prot_WP_013618898.1_2936) |  | + |  |  |  |  |  | [2.A.1.1.3](http://tcdb.org/search/result.php?tc=2.A.1.1.3) | -144 | P0AGF4 | Sugars | Xylose |
| [WP_013619196.1](file:///Volumes/ESD-USB/Summer%202018/Hassan%20Submission%20PLosOne/content.html#lcl|NC_015164.1_prot_WP_013619196.1_3249) |  | + |  |  |  |  |  | [2.A.1.1.3](http://tcdb.org/search/result.php?tc=2.A.1.1.3) | -101 | P0AGF4 | Sugars | Xylose |
| [WP_007218457.1](file:///Volumes/ESD-USB/Summer%202018/Hassan%20Submission%20PLosOne/content.html#lcl|NZ_CP012801.1_prot_WP_007218457.1_5241) |  |  | + |  |  |  |  | [2.A.1.1.3](http://tcdb.org/search/result.php?tc=2.A.1.1.3) | -154 | P0AGF4 | Sugars | Xylose |
| [WP_007219285.1](file:///Volumes/ESD-USB/Summer%202018/Hassan%20Submission%20PLosOne/content.html#lcl|NZ_CP012801.1_prot_WP_007219285.1_3022) |  |  | + |  |  |  |  | [2.A.1.1.3](http://tcdb.org/search/result.php?tc=2.A.1.1.3) | -106 | P0AGF4 | Sugars | Xylose |
| [WP_022209475.1](file:///Volumes/ESD-USB/Summer%202018/Hassan%20Submission%20PLosOne/content.html#lcl|NZ_CP012801.1_prot_WP_022209475.1_708) |  |  | + |  |  |  |  | [2.A.1.1.3](http://tcdb.org/search/result.php?tc=2.A.1.1.3) | -93 | P0AGF4 | Sugars | Xylose |
| WP_008765653.1 |  |  |  | + |  |  |  | 2.A.1.1.3 | -155 | P0AGF4 | Sugars | Xylose |
| WP_011109239.1 |  |  |  | + |  |  |  | 2.A.1.1.3 | -102 | P0AGF4 | Sugars | Xylose |
| WP_032813836.1 |  |  |  | + |  |  |  | 2.A.1.1.3 | -95 | P0AGF4 | Sugars | Xylose |
| WP_008767094.1 |  |  |  | + |  |  |  | 2.A.1.1.3 | -89 | P0AGF4 | Sugars | Xylose |
| YP_099544.1 |  |  |  |  | + |  |  | 2.A.1.1.3 | -156 | P0AGF4 | Sugars | Xylose |
| YP_098222.1 |  |  |  |  | + |  |  | 2.A.1.1.3 | -102 | P0AGF4 | Sugars | Xylose |
| [WP_004295877.1](file:///Volumes/ESD-USB/Summer%202018/Hassan%20Submission%20PLosOne/content.html#lcl|NZ_CP012938.1_prot_WP_004295877.1_795) |  |  |  |  |  | + |  | [2.A.1.1.3](http://tcdb.org/search/result.php?tc=2.A.1.1.3) | -153 | P0AGF4 | Sugars | Xylose |
| [WP_004297558.1](file:///Volumes/ESD-USB/Summer%202018/Hassan%20Submission%20PLosOne/content.html#lcl|NZ_CP012938.1_prot_WP_004297558.1_279) |  |  |  |  |  | + |  | [2.A.1.1.3](http://tcdb.org/search/result.php?tc=2.A.1.1.3) | -96 | P0AGF4 | Sugars | Xylose |
| [WP_012056002.1](file:///Volumes/ESD-USB/Summer%202018/Library/Application%20Support/Microsoft/Office/Office%202011%20AutoRecovery/content.html#lcl|NC_009614.1_prot_WP_012056002.1_3975) |  |  |  |  |  |  | + | [2.A.1.1.3](http://tcdb.org/search/result.php?tc=2.A.1.1.3) | -157 | P0AGF4 | Sugars | Xylose |
| WP_011964891.1 |  |  |  |  |  |  | + | [2.A.1.1.3](http://tcdb.org/search/result.php?tc=2.A.1.1.3) | -96 | P0AGF4 | Sugars | Xylose |
| [WP_026367882.1](file:///Volumes/ESD-USB/Summer%202018/Hassan%20Submission%20PLosOne/content.html#lcl|NZ_CP012801.1_prot_WP_026367882.1_4125) |  |  | + |  |  |  |  | [2.A.1.1.106](http://tcdb.org/search/result.php?tc=2.A.1.1.106) | -68 | P46333 | Unknown | Unknown |
| YP_101800.1 |  |  |  |  | + |  |  | 2.A.1.1.106 | -85 | P46333 | Metabolites | Metabolites |
| ABI39_04620 | + |  |  |  |  |  |  | 2.A.1.2.14 | -115 | P23910 | Sugars | Arabinose |
| [WP_041584072.1](file:///Volumes/ESD-USB/Summer%202018/Hassan%20Submission%20PLosOne/content.html#lcl|NC_015164.1_prot_WP_041584072.1_2980) |  | + |  |  |  |  |  | [2.A.1.2.14](http://tcdb.org/search/result.php?tc=2.A.1.2.14) | -111 | P23910 | Sugars | Arabinose |
| [WP_007213793.1](file:///Volumes/ESD-USB/Summer%202018/Hassan%20Submission%20PLosOne/content.html#lcl|NZ_CP012801.1_prot_WP_007213793.1_458) |  |  | + |  |  |  |  | [2.A.1.2.14](http://tcdb.org/search/result.php?tc=2.A.1.2.14) | -115 | P23910 | Sugars | Arabinose |
| WP_008767378.1 |  |  |  | + |  |  |  | 2.A.1.2.14 | -119 | P23910 | Sugars | Arabinose |
| [WP_004298658.1](file:///Volumes/ESD-USB/Summer%202018/Hassan%20Submission%20PLosOne/content.html#lcl|NZ_CP012938.1_prot_WP_004298658.1_3050) |  |  |  |  |  | + |  | [2.A.1.2.14](http://tcdb.org/search/result.php?tc=2.A.1.2.14) | -118 | P23910 | Sugars | Arabinose |
| [WP_004297086.1](file:///Volumes/ESD-USB/Summer%202018/Hassan%20Submission%20PLosOne/content.html#lcl|NZ_CP012938.1_prot_WP_004297086.1_1387) |  |  |  |  |  | + |  | [2.A.1.2.14](http://tcdb.org/search/result.php?tc=2.A.1.2.14) | -32 | P23910 | Sugars | Arabinose |
| [WP_011965152.1](file:///Volumes/ESD-USB/Summer%202018/Library/Application%20Support/Microsoft/Office/Office%202011%20AutoRecovery/content.html#lcl|NC_009614.1_prot_WP_011965152.1_1335) |  |  |  |  |  |  | + | [2.A.1.2.14](http://tcdb.org/search/result.php?tc=2.A.1.2.14) | -113 | P23910 | Sugars | Arabinose |
| [WP_029426740.1](file:///Volumes/ESD-USB/Summer%202018/Hassan%20Submission%20PLosOne/content.html#lcl|NZ_CP012801.1_prot_WP_029426740.1_4791) |  |  | + |  |  |  |  | [2.A.1.2.65](http://tcdb.org/search/result.php?tc=2.A.1.2.65) | -34 | P77389 | Unknown | Unknown |
| WP_008764888.1 |  |  |  | + |  |  |  | 2.A.1.2.65 | -33 | P77389 | Unknown | Unknown |
| [WP_004297070.1](file:///Volumes/ESD-USB/Summer%202018/Hassan%20Submission%20PLosOne/content.html#lcl|NZ_CP012938.1_prot_WP_004297070.1_1375) |  |  |  |  |  | + |  | [2.A.1.2.65](http://tcdb.org/search/result.php?tc=2.A.1.2.65) | -32 | P77389 | Unknown | Unknown |
| ABI39_16625 | + |  |  |  |  |  |  | 2.A.1.2.97 | -55 | I7FD23 | Drugs | Fluoroquinolones |
| [WP_013618446.1](file:///Volumes/ESD-USB/Summer%202018/Hassan%20Submission%20PLosOne/content.html#lcl|NC_015164.1_prot_WP_013618446.1_2442) |  | + |  |  |  |  |  | [2.A.1.2.97](http://tcdb.org/search/result.php?tc=2.A.1.2.97) | -66 | I7FD23 | Sugar derivatives | Unknown |
| WP_029427281.1 |  |  | + |  |  |  |  | [2.A.1.2.97](http://tcdb.org/search/result.php?tc=2.A.1.2.97) | -63 | I7FD23 | Drugs | Fluoroquinolones |
| YP_098044.1 |  |  |  |  | + |  |  | 2.A.1.2.97 | -63 | I7FD23 | Drugs | Fluoroquinolones |
| YP_100371.1 |  |  |  |  | + |  |  | 2.A.1.2.97 | -58 | I7FD23 | Drugs | Fluoroquinolones |
| YP_099962.1 |  |  |  |  | + |  |  | 2.A.1.3.2 | -67 | P0AEJ0 | Drugs | Nalidixic acid/Rhodamine |
| ABI39_05560 | + |  |  |  |  |  |  | 2.A.1.3.17 | 0 | P76269 | Drugs | Trimethoprim |
| [WP_029428686.1](file:///Volumes/ESD-USB/Summer%202018/Hassan%20Submission%20PLosOne/content.html#lcl|NZ_CP012801.1_prot_WP_029428686.1_4194) |  |  | + |  |  |  |  | [2.A.1.3.17](http://tcdb.org/search/result.php?tc=2.A.1.3.17) | -172 | P76269 | [Drugs](https://www.ebi.ac.uk/chebi/searchId.do;jsessionid=A9D16DCB24C6F74339FC28A4941EFBB6?chebiId=CHEBI:45924) | Trimethoprim |
| WP_008766368.1 |  |  |  | + |  |  |  | 2.A.1.3.17 | 0 | P76269 | Drugs | Trimethoprim |
| YP_100971.1 |  |  |  |  | + |  |  | 2.A.1.3.17 | -174 | P76269 | Drugs | Trimethoprim |
| [WP_005849911.1_](file:///Volumes/ESD-USB/Summer%202018/Library/Application%20Support/Microsoft/Office/Office%202011%20AutoRecovery/content.html#lcl|NC_009614.1_prot_WP_005849911.1_1165) |  |  |  |  |  |  | + | [2.A.1.3.17](http://tcdb.org/search/result.php?tc=2.A.1.3.17) | 0 | P76269 | [Sugars](http://tcdb.org/search/result.php?tc=2.A.25.1.5) | Trimethoprim |
| [WP_013618926.1](file:///Volumes/ESD-USB/Summer%202018/Hassan%20Submission%20PLosOne/content.html#lcl|NC_015164.1_prot_WP_013618926.1_2964) |  | + |  |  |  |  |  | [2.A.1.3.18](http://tcdb.org/search/result.php?tc=2.A.1.3.18) | -10 | Q9KIH3 | Drugs | Phytoalexins, flavenoids and salicylate |
| [WP_013618032.1](file:///Volumes/ESD-USB/Summer%202018/Hassan%20Submission%20PLosOne/content.html#lcl|NC_015164.1_prot_WP_013618032.1_2008) |  | + |  |  |  |  |  | [2.A.1.3.18](http://tcdb.org/search/result.php?tc=2.A.1.3.18) | -07 | Q9KIH3 | Drugs | Phytoalexins, flavenoids and salicylate |
| WP_022471470.1 |  |  |  | + |  |  |  | 2.A.1.3.24 | -07 | O51919 | Drugs | Fluoroquinolones/Chloramphenicol |
| WP_022471471.1 |  |  |  | + |  |  |  | 2.A.1.3.36 | -36 | C5W790 | Amino acids | Cysteine |
| YP_100575.1 |  |  |  |  | + |  |  | 2.A.1.3.36 | -33 | C5W790 | Amino acids | Cysteine |
| YP_099963.1 |  |  |  |  | + |  |  | 2.A.1.3.36 | -21 | C5W790 | Amino acids | Cysteine |
| [WP_004300158.1](file:///Volumes/ESD-USB/Summer%202018/Hassan%20Submission%20PLosOne/content.html#lcl|NZ_CP012938.1_prot_WP_004300158.1_1217) |  |  |  |  |  | + |  | [2.A.1.3.36](http://tcdb.org/search/result.php?tc=2.A.1.3.36) | -38 | C5W790 | Amino Acids | Cysteine |
| WP_011107160.1 |  |  |  | + |  |  |  | 2.A.1.4.6 | -66 | Q9Z7N9 | Sugars | Glucose |
| ABI39_01180 | + |  |  |  |  |  |  | 2.A.1.7.1 | -86 | P11551 | Sugars | Fucose |
| [WP_007213271.1](file:///Volumes/ESD-USB/Summer%202018/Hassan%20Submission%20PLosOne/content.html#lcl|NZ_CP012801.1_prot_WP_007213271.1_4530) |  |  | + |  |  |  |  | [2.A.1.7.1](http://tcdb.org/search/result.php?tc=2.A.1.7.1) | -86 | P11551 | [Drugs](https://www.ebi.ac.uk/chebi/searchId.do;jsessionid=A9D16DCB24C6F74339FC28A4941EFBB6?chebiId=CHEBI:33984) | Fucose |
| [WP_022210477.1](file:///Volumes/ESD-USB/Summer%202018/Hassan%20Submission%20PLosOne/content.html#lcl|NZ_CP012801.1_prot_WP_022210477.1_2912) |  |  | + |  |  |  |  | [2.A.1.7.1](http://tcdb.org/search/result.php?tc=2.A.1.7.1) | -81 | P11551 | [Sugars](https://www.ebi.ac.uk/chebi/searchId.do;jsessionid=A9D16DCB24C6F74339FC28A4941EFBB6?chebiId=CHEBI:33984) | Fucose |
| [WP_029428183.1](file:///Volumes/ESD-USB/Summer%202018/Hassan%20Submission%20PLosOne/content.html#lcl|NZ_CP012801.1_prot_WP_029428183.1_5051) |  |  | + |  |  |  |  | [2.A.1.7.1](http://tcdb.org/search/result.php?tc=2.A.1.7.1) | -75 | P11551 | [Sugars](https://www.ebi.ac.uk/chebi/searchId.do;jsessionid=A9D16DCB24C6F74339FC28A4941EFBB6?chebiId=CHEBI:33984) | Fucose |
| WP_008767090.1 |  |  |  | + |  |  |  | 2.A.1.7.1 | -86 | P11551 | Sugars | Maltooligosaccharide |
| WP_008764988.1 |  |  |  | + |  |  |  | 2.A.1.7.1 | -72 | P11551 | Sugars | Maltooligosaccharide |
| YP_097540.1 |  |  |  |  | + |  |  | 2.A.1.7.1 | -77 | P11551 | Sugars | L-fucose |
| [WP_004305136.1](file:///Volumes/ESD-USB/Summer%202018/Hassan%20Submission%20PLosOne/content.html#lcl|NZ_CP012938.1_prot_WP_004305136.1_3553) |  |  |  |  |  | + |  | [2.A.1.7.1](http://tcdb.org/search/result.php?tc=2.A.1.7.1) | -84 | P11551 | [Sugars](https://www.ebi.ac.uk/chebi/searchId.do;jsessionid=A9D16DCB24C6F74339FC28A4941EFBB6?chebiId=CHEBI:33984) | Fucose |
| [WP_005843574.1](file:///Volumes/ESD-USB/Summer%202018/Library/Application%20Support/Microsoft/Office/Office%202011%20AutoRecovery/content.html#lcl|NC_009614.1_prot_WP_005843574.1_222) |  |  |  |  |  |  | + | [2.A.1.7.1](http://tcdb.org/search/result.php?tc=2.A.1.7.1) | -86 | P11551 | [Sugars](http://tcdb.org/search/result.php?tc=2.A.25.1.5) | Fucose |
| [WP_008668518.1](file:///Volumes/ESD-USB/Summer%202018/Library/Application%20Support/Microsoft/Office/Office%202011%20AutoRecovery/content.html#lcl|NC_009614.1_prot_WP_008668518.1_1355) |  |  |  |  |  |  | + | [2.A.1.7.1](http://tcdb.org/search/result.php?tc=2.A.1.7.1) | -74 | P11551 | [Sugars](http://tcdb.org/search/result.php?tc=2.A.25.1.5) | Fucose |
| ABI39_12975 | + |  |  |  |  |  |  | 2.A.1.7.2 | -80 | P0C105 | Sugars | Glucose/Galactose |
| ABI39_11645 | + |  |  |  |  |  |  | 2.A.1.7.2 | -38 | P0C105 | Sugars | Glucose/Galactose |
| [WP_013616306.1](file:///Volumes/ESD-USB/Summer%202018/Hassan%20Submission%20PLosOne/content.html#lcl|NC_015164.1_prot_WP_013616306.1_229) |  | + |  |  |  |  |  | [2.A.1.7.2](http://tcdb.org/search/result.php?tc=2.A.1.7.2) | -81 | P0C105 | Sugars | Glucose/Galactose |
| [WP_013616962.1](file:///Volumes/ESD-USB/Summer%202018/Hassan%20Submission%20PLosOne/content.html#lcl|NC_015164.1_prot_WP_013616962.1_907) |  | + |  |  |  |  |  | [2.A.1.7.2](http://tcdb.org/search/result.php?tc=2.A.1.7.2) | -17 | P0C105 | Sugars | Glucose/Galactose |
| [WP_007216273.1](file:///Volumes/ESD-USB/Summer%202018/Hassan%20Submission%20PLosOne/content.html#lcl|NZ_CP012801.1_prot_WP_007216273.1_4785) |  |  | + |  |  |  |  | [2.A.1.7.2](http://tcdb.org/search/result.php?tc=2.A.1.7.2) | -74 | P0C105 | Sugars | Glucose/Galactose |
| [WP_007214525.1](file:///Volumes/ESD-USB/Summer%202018/Hassan%20Submission%20PLosOne/content.html#lcl|NZ_CP012801.1_prot_WP_007214525.1_864) |  |  | + |  |  |  |  | [2.A.1.7.2](http://tcdb.org/search/result.php?tc=2.A.1.7.2) | -39 | P0C105 | Sugars | Glucose/Galactose |
| WP_008760657.1 |  |  |  | + |  |  |  | 2.A.1.7.2 | -46 | P0C105 | Sugars | Maltooligosaccharide |
| YP_100628.1 |  |  |  |  | + |  |  | 2.A.1.7.2 | -46 | P0C105 | Sugars | Glucose/Galactose |
| YP_098936.1 |  |  |  |  | + |  |  | 2.A.1.7.2 | -40 | P0C105 | Sugars | Glucose/Galactose |
| [WP_004297464.1](file:///Volumes/ESD-USB/Summer%202018/Hassan%20Submission%20PLosOne/content.html#lcl|NZ_CP012938.1_prot_WP_004297464.1_210) |  |  |  |  |  | + |  | [2.A.1.7.2](http://tcdb.org/search/result.php?tc=2.A.1.7.2) | -38 | P0C105 | Sugars | Glucose/Galactose |
| [WP_005847347.1](file:///Volumes/ESD-USB/Summer%202018/Library/Application%20Support/Microsoft/Office/Office%202011%20AutoRecovery/content.html#lcl|NC_009614.1_prot_WP_005847347.1_2559) |  |  |  |  |  |  | + | [2.A.1.7.2](http://tcdb.org/search/result.php?tc=2.A.1.7.2) | -78 | P0C105 | Sugars | Glucose/Galactose |
| [WP_005846203.1](file:///Volumes/ESD-USB/Summer%202018/Library/Application%20Support/Microsoft/Office/Office%202011%20AutoRecovery/content.html#lcl|NC_009614.1_prot_WP_005846203.1_2277) |  |  |  |  |  |  | + | [2.A.1.7.2](http://tcdb.org/search/result.php?tc=2.A.1.7.2) | -37 | P0C105 | Sugars | Glucose/Galactose |
| ABI39_18290 | + |  |  |  |  |  |  | 2.A.1.7.8 | -73 | Q8EBL0 | Sugar derivatives | N-acetylglucosamine |
| WP_008764497.1 |  |  |  | + |  |  |  | 2.A.1.7.8 | -76 | Q8EBL0 | Sugars | Maltooligosaccharide |
| YP_098290.1 |  |  |  |  | + |  |  | 2.A.1.7.8 | -69 | Q8EBL0 | Sugars | Glucose/Galactose |
| [WP_012055794.1](file:///Volumes/ESD-USB/Summer%202018/Library/Application%20Support/Microsoft/Office/Office%202011%20AutoRecovery/content.html#lcl|NC_009614.1_prot_WP_012055794.1_3553) |  |  |  |  |  |  | + | [2.A.1.7.8](http://tcdb.org/search/result.php?tc=2.A.1.7.8) | -74 | Q8EBL0 | Sugars | Glucose/Galactose |
| [WP_029426689.1](file:///Volumes/ESD-USB/Summer%202018/Hassan%20Submission%20PLosOne/content.html#lcl|NZ_CP012801.1_prot_WP_029426689.1_3304) |  |  | + |  |  |  |  | [2.A.1.7.9](http://tcdb.org/search/result.php?tc=2.A.1.7.9) | -99 | A1S4V0 | Sugars | Glucose/Galactose |
| WP_062695798.1 |  |  |  | + |  |  |  | 2.A.1.7.9 | -114 | A1S4V0 | Sugars | Maltooligosaccharide |
| [WP_004299605.1](file:///Volumes/ESD-USB/Summer%202018/Hassan%20Submission%20PLosOne/content.html#lcl|NZ_CP012938.1_prot_WP_004299605.1_3539) |  |  |  |  |  | + |  | [2.A.1.7.9](http://tcdb.org/search/result.php?tc=2.A.1.7.9) | -114 | A1S4V0 | Sugars | N-acetylglucosamine |
| [WP_013616841.1](file:///Volumes/ESD-USB/Summer%202018/Hassan%20Submission%20PLosOne/content.html#lcl|NC_015164.1_prot_WP_013616841.1_784) |  | + |  |  |  |  |  | [2.A.1.7.10](http://tcdb.org/search/result.php?tc=2.A.1.7.10) | -73 | A1S5F4 | Sugars | Glucose/Galactose |
| [WP_029428233.1](file:///Volumes/ESD-USB/Summer%202018/Hassan%20Submission%20PLosOne/content.html#lcl|NZ_CP012801.1_prot_WP_029428233.1_1373) |  |  | + |  |  |  |  | [2.A.1.7.11](http://tcdb.org/search/result.php?tc=2.A.1.7.11) | -57 | A1S297 | Sugars | Mannose |
| [WP_004299239.1](file:///Volumes/ESD-USB/Summer%202018/Hassan%20Submission%20PLosOne/content.html#lcl|NZ_CP012938.1_prot_WP_004299239.1_2183) |  |  |  |  |  | + |  | [2.A.1.7.11](http://tcdb.org/search/result.php?tc=2.A.1.7.11) | -57 | A1S297 | Sugars | Mannose |
| WP_008766592.1 |  |  |  | + |  |  |  | 2.A.1.7.16 | 0 | Q8A5Y0 | Sugars | Glucose/Glalactoses/Mannose |
| [WP_004299403.1](file:///Volumes/ESD-USB/Summer%202018/Hassan%20Submission%20PLosOne/content.html#lcl|NZ_CP012938.1_prot_WP_004299403.1_2069) |  |  |  |  |  | + |  | [2.A.1.7.16](http://tcdb.org/search/result.php?tc=2.A.1.7.16) | -108 | Q8A5Y0 | Sugars | Mannose |
| ABI39_08235 | + |  |  |  |  |  |  | 2.A.1.7.17 | 0 | Q8A6W8 | Sugars | Fructose |
| [WP_013618744.1](file:///Volumes/ESD-USB/Summer%202018/Hassan%20Submission%20PLosOne/content.html#lcl|NC_015164.1_prot_WP_013618744.1_2786) |  | + |  |  |  |  |  | [2.A.1.7.17](http://tcdb.org/search/result.php?tc=2.A.1.7.17) | 0 | Q8A6W8 | Sugars | Fructose |
| [WP_007217377.1](file:///Volumes/ESD-USB/Summer%202018/Hassan%20Submission%20PLosOne/content.html#lcl|NZ_CP012801.1_prot_WP_007217377.1_1269) |  |  | + |  |  |  |  | [2.A.1.7.17](http://tcdb.org/search/result.php?tc=2.A.1.7.17) | 0 | Q8A6W8 | Sugars | Fucose |
| WP_008763289.1 |  |  |  | + |  |  |  | 2.A.1.7.17 | 0 | Q8A6W8 | Sugars | Fructose |
| YP_100619.1 |  |  |  |  | + |  |  | 2.A.1.7.17 | 0 | Q8A6W8 | Sugars | Fructose |
| [WP_008667877.1](file:///Volumes/ESD-USB/Summer%202018/Library/Application%20Support/Microsoft/Office/Office%202011%20AutoRecovery/content.html#lcl|NC_009614.1_prot_WP_008667877.1_1649) |  |  |  |  |  |  | + | [2.A.1.7.17](http://tcdb.org/search/result.php?tc=2.A.1.7.17) | 0 | Q8A6W8 | Sugars | Glucose/Galactose |
| [WP_029427853.1](file:///Volumes/ESD-USB/Summer%202018/Hassan%20Submission%20PLosOne/content.html#lcl|NZ_CP012801.1_prot_WP_029427853.1_1624) |  |  | + |  |  |  |  | [2.A.1.7.18](http://tcdb.org/search/result.php?tc=2.A.1.7.18) | 0 | Q89YS8 | Sugars | Glucose/Galactose |
| WP_062695292.1 |  |  |  | + |  |  |  | 2.A.1.7.18 | 0 | Q89YS8 | Sugars | Glucose, Galactose |
| ABI39_18155 | + |  |  |  |  |  |  | 2.A.1.10.1 | -120 | P0AFF4 | Nucleosides | Guanosine/inosine/cytidine/ thymidine |
| [WP_041583816.1](file:///Volumes/ESD-USB/Summer%202018/Hassan%20Submission%20PLosOne/content.html#lcl|NC_015164.1_prot_WP_041583816.1_197) |  | + |  |  |  |  |  | [2.A.1.10.1](http://tcdb.org/search/result.php?tc=2.A.1.10.1) | -122 | P0AFF4 | Nucleosides | Guanosine/inosine/cytidine/ thymidine |
| [WP_012055782.1](file:///Volumes/ESD-USB/Summer%202018/Library/Application%20Support/Microsoft/Office/Office%202011%20AutoRecovery/content.html#lcl|NC_009614.1_prot_WP_012055782.1_3528) |  |  |  |  |  |  | + | [2.A.1.10.1](http://tcdb.org/search/result.php?tc=2.A.1.10.1) | -119 | P0AFF4 | Nucleosides | Guanosine, inosine, cytidine |
| [WP_007212810.1](file:///Volumes/ESD-USB/Summer%202018/Hassan%20Submission%20PLosOne/content.html#lcl|NZ_CP012801.1_prot_WP_007212810.1_4147) |  |  | + |  |  |  |  | [2.A.1.10.2](http://tcdb.org/search/result.php?tc=2.A.1.10.2) | -157 | P45562 | Nucleosides | Xanthosine, inosine, adenosine, cytidine and thymidine |
| WP_008760003.1 |  |  |  | + |  |  |  | 2.A.1.10.2 | -155 | P45562 | Nucleosides | Xantosine |
| YP_098311.1 |  |  |  |  | + |  |  | 2.A.1.10.2 | -153 | P45562 | Nucleoside | Nucleoside |
| [WP_004301812.1](file:///Volumes/ESD-USB/Summer%202018/Hassan%20Submission%20PLosOne/content.html#lcl|NZ_CP012938.1_prot_WP_004301812.1_4385) |  |  |  |  |  | + |  | [2.A.1.10.2](http://tcdb.org/search/result.php?tc=2.A.1.10.2) | -154 | P45562 | Nucleosides | Xantosine |
| ABI39_09035 | + |  |  |  |  |  |  | 2.A.1.14.25 | -55 | Q07YH1 | Carboxylates | D-Mannuronate |
| [WP_013617387.1](file:///Volumes/ESD-USB/Summer%202018/Hassan%20Submission%20PLosOne/content.html#lcl|NC_015164.1_prot_WP_013617387.1_1356) |  | + |  |  |  |  |  | [2.A.1.14.25](http://tcdb.org/search/result.php?tc=2.A.1.14.25) | -55 | Q07YH1 | Sugar derivatives | D-mannuronate |
| [WP_041584407.1](file:///Volumes/ESD-USB/Summer%202018/Hassan%20Submission%20PLosOne/content.html#lcl|NC_015164.1_prot_WP_041584407.1_2554) |  | + |  |  |  |  |  | [2.A.1.14.25](http://tcdb.org/search/result.php?tc=2.A.1.14.25) | -47 | Q07YH1 | Sugar derivatives | D-mannuronate |
| [WP_007212474.1](file:///Volumes/ESD-USB/Summer%202018/Hassan%20Submission%20PLosOne/content.html#lcl|NZ_CP012801.1_prot_WP_007212474.1_3865) |  |  | + |  |  |  |  | [2.A.1.14.25](http://tcdb.org/search/result.php?tc=2.A.1.14.25) | -56 | Q07YH1 | Sugars | mannuronate |
| WP_008764329.1 |  |  |  | + |  |  |  | 2.A.1.14.25 | -58 | Q07YH1 | Sugars | D-Mannuronate |
| YP_098170.1 |  |  |  |  | + |  |  | 2.A.1.14.25 | -56 | Q07YH1 | Sugars | D-mannuronate |
| [WP_004301500.1](file:///Volumes/ESD-USB/Summer%202018/Hassan%20Submission%20PLosOne/content.html#lcl|NZ_CP012938.1_prot_WP_004301500.1_4180) |  |  |  |  |  | + |  | [2.A.1.14.25](http://tcdb.org/search/result.php?tc=2.A.1.14.25) | -58 | Q07YH1 | Sugar derivatives | D-mannuronate |
| [WP_005842798.1](file:///Volumes/ESD-USB/Summer%202018/Library/Application%20Support/Microsoft/Office/Office%202011%20AutoRecovery/content.html#lcl|NC_009614.1_prot_WP_005842798.1_1772) |  |  |  |  |  |  | + | [2.A.1.14.25](http://tcdb.org/search/result.php?tc=2.A.1.14.25) | -55 | Q07YH1 | Sugar derivatives | D-mannuronate |
| [WP_007215307.1](file:///Volumes/ESD-USB/Summer%202018/Hassan%20Submission%20PLosOne/content.html#lcl|NZ_CP012801.1_prot_WP_007215307.1_3893) |  |  | + |  |  |  |  | [2.A.1.15.7](http://tcdb.org/search/result.php?tc=2.A.1.15.7) | -09 | Q6FBB3 | Aromatic | Benzoate |
| ABI39_12300 | + |  |  |  |  |  |  | 2.A.1.21.22 | -67 | Q7BKK4 | Drugs | Macrolides |
| [WP_029328648.1](file:///Volumes/ESD-USB/Summer%202018/Hassan%20Submission%20PLosOne/content.html#lcl|NZ_CP012801.1_prot_WP_029328648.1_5218) |  |  | + |  |  |  |  | [2.A.1.21.22](http://tcdb.org/search/result.php?tc=2.A.1.21.22) | -91 | Q7BKK4 | Drugs | Macrolides |
| YP_099571.1 |  |  |  |  | + |  |  | 2.A.1.21.22 | -98 | Q7BKK4 | Drugs | Macrolides |
| ABI39_03275 | + |  |  |  |  |  |  | 2.A.1.25.2 | -13 | P0AE16 | Drugs | Penicillin |
| [WP_013616877.1](file:///Volumes/ESD-USB/Summer%202018/Hassan%20Submission%20PLosOne/content.html#lcl|NC_015164.1_prot_WP_013616877.1_818) |  | + |  |  |  |  |  | [2.A.1.25.2](http://tcdb.org/search/result.php?tc=2.A.1.25.2) | -11 | P0AE16 | Unknown | Unknown |
| WP_008762567.1 |  |  |  | + |  |  |  | 2.A.1.25.2 | -24 | P0AE16 | Unknown | Unknown |
| WP_008765822.1 |  |  |  | + |  |  |  | 2.A.1.25.2 | -18 | P0AE16 | Unknown | Unknown |
| [WP_011964827.1](file:///Volumes/ESD-USB/Summer%202018/Library/Application%20Support/Microsoft/Office/Office%202011%20AutoRecovery/content.html#lcl|NC_009614.1_prot_WP_011964827.1_604) |  |  |  |  |  |  | + | [2.A.1.25.2](http://tcdb.org/search/result.php?tc=2.A.1.25.2) | -13 | P0AE16 | Drugs | Penicillin |
| [WP_011965657.1](file:///Volumes/ESD-USB/Summer%202018/Library/Application%20Support/Microsoft/Office/Office%202011%20AutoRecovery/content.html#lcl|NC_009614.1_prot_WP_011965657.1_2548) |  |  |  |  |  |  | + | [2.A.1.25.3](http://tcdb.org/search/result.php?tc=2.A.1.25.3) | -23 | Q5F6G0 | unknown | unknown |
| [WP_013617543.1](file:///Volumes/ESD-USB/Summer%202018/Hassan%20Submission%20PLosOne/content.html#lcl|NC_015164.1_prot_WP_013617543.1_1509) |  | + |  |  |  |  |  | [2.A.1.33.2](http://tcdb.org/search/result.php?tc=2.A.1.33.2) | 0 | A7LYG9 | Unknown | Unknown |
| [WP_013616126.1](file:///Volumes/ESD-USB/Summer%202018/Hassan%20Submission%20PLosOne/content.html#lcl|NC_015164.1_prot_WP_013616126.1_48) |  | + |  |  |  |  |  | [2.A.1.35.1](http://tcdb.org/search/result.php?tc=2.A.1.35.1) | -132 | P52067 | Drugs | Fosidomycin/Trimethoprim |
| [WP_007212222.1](file:///Volumes/ESD-USB/Summer%202018/Hassan%20Submission%20PLosOne/content.html#lcl|NZ_CP012801.1_prot_WP_007212222.1_4673) |  |  | + |  |  |  |  | [2.A.1.35.1](http://tcdb.org/search/result.php?tc=2.A.1.35.1) | -141 | P52067 | Drugs | Fosidomycin |
| WP_062695919.1 |  |  |  | + |  |  |  | 2.A.1.35.1 | -144 | P52067 | Drugs | Fosidomycin |
| YP_099529.1 |  |  |  |  | + |  |  | 2.A.1.35.1 | -146 | P52067 | Drugs | Fosmidomycin |
| [WP_004297126.1](file:///Volumes/ESD-USB/Summer%202018/Hassan%20Submission%20PLosOne/content.html#lcl|NZ_CP012938.1_prot_WP_004297126.1_1422) |  |  |  |  |  | + |  | [2.A.1.35.1](http://tcdb.org/search/result.php?tc=2.A.1.35.1) | -147 | P52067 | Unknown | Unknown |
| ABI39_12470 | + |  |  |  |  |  |  | 2.A.1.36.3 | -74 | P76628 | Unknown | Unknown |
| [WP_029428581.1](file:///Volumes/ESD-USB/Summer%202018/Hassan%20Submission%20PLosOne/content.html#lcl|NZ_CP012801.1_prot_WP_029428581.1_4368) |  |  | + |  |  |  |  | [2.A.1.36.3](http://tcdb.org/search/result.php?tc=2.A.1.36.3) | -73 | P76628 | Unknown | Unknown |
| WP_062695953.1 |  |  |  | + |  |  |  | 2.A.1.36.3 | -73 | P76628 | Unknown | Unknown |
| YP_098698.1 |  |  |  |  | + |  |  | 2.A.1.36.3 | -70 | P76628 | Unknown | Unknown |
| YP_098698.1 |  |  |  |  | + |  |  | 2.A.1.36.3 | -70 | P76628 | Unknown | Unknown |
| [WP_004296951.1](file:///Volumes/ESD-USB/Summer%202018/Hassan%20Submission%20PLosOne/content.html#lcl|NZ_CP012938.1_prot_WP_004296951.1_56) |  |  |  |  |  | + |  | [2.A.1.36.3](http://tcdb.org/search/result.php?tc=2.A.1.36.3) | -68 | P76628 | Unknown | Unknown |
| [WP_005847437.1](file:///Volumes/ESD-USB/Summer%202018/Library/Application%20Support/Microsoft/Office/Office%202011%20AutoRecovery/content.html#lcl|NC_009614.1_prot_WP_005847437.1_2455) |  |  |  |  |  |  | + | [2.A.1.36.3](http://tcdb.org/search/result.php?tc=2.A.1.36.3) | -76 | P76628 | Unknown | Unknown |
| ABI39_11240 | + |  |  |  |  |  |  | 2.A.1.46.5 | -65 | C2UR80 | Unknown | Unknown |
| ABI39_14015 | + |  |  |  |  |  |  | 2.A.1.46.5 | -14 | C2UR80 | Unknown | Unknown |
| [WP_013617029.1](file:///Volumes/ESD-USB/Summer%202018/Hassan%20Submission%20PLosOne/content.html#lcl|NC_015164.1_prot_WP_013617029.1_980) |  | + |  |  |  |  |  | [2.A.1.46.5](http://tcdb.org/search/result.php?tc=2.A.1.46.5) | -58 | C2UR80 | Drugs | Fluoroquinolones |
| [WP_013617408.1](file:///Volumes/ESD-USB/Summer%202018/Hassan%20Submission%20PLosOne/content.html#lcl|NC_015164.1_prot_WP_013617408.1_1377) |  | + |  |  |  |  |  | [2.A.1.46.5](http://tcdb.org/search/result.php?tc=2.A.1.46.5) | -11 | C2UR80 | Drugs | Fluoroquinolones |
| [WP_029428328.1](file:///Volumes/ESD-USB/Summer%202018/Hassan%20Submission%20PLosOne/content.html#lcl|NZ_CP012801.1_prot_WP_029428328.1_1206) |  |  | + |  |  |  |  | [2.A.1.46.5](http://tcdb.org/search/result.php?tc=2.A.1.46.5) | -63 | C2UR80 | Unknown | Unknown |
| [WP_007213966.1](file:///Volumes/ESD-USB/Summer%202018/Hassan%20Submission%20PLosOne/content.html#lcl|NZ_CP012801.1_prot_WP_007213966.1_304) |  |  | + |  |  |  |  | [2.A.1.46.5](http://tcdb.org/search/result.php?tc=2.A.1.46.5) | -17 | C2UR80 | Unknown | Unknown |
| WP_008763377.1 |  |  |  | + |  |  |  | 2.A.1.46.5 | -60 | C2UR80 | Drugs | Fluoroquinolones |
| WP_008761375.1 |  |  |  | + |  |  |  | 2.A.1.46.5 | -17 | C2UR80 | Drugs | Fluoroquinolones |
| YP_100545.1 |  |  |  |  | + |  |  | 2.A.1.46.5 | -58 | C2UR80 | Drugs | Quinolones |
| YP_099445.1 |  |  |  |  | + |  |  | 2.A.1.46.5 | -18 | C2UR80 | Drugs | Quinolones |
| [WP_004300864.1](file:///Volumes/ESD-USB/Summer%202018/Hassan%20Submission%20PLosOne/content.html#lcl|NZ_CP012938.1_prot_WP_004300864.1_1674) |  |  |  |  |  | + |  | [2.A.1.46.5](http://tcdb.org/search/result.php?tc=2.A.1.46.5) | -60 | C2UR80 | Drugs | Quinolones |
| [WP_004295744.1](file:///Volumes/ESD-USB/Summer%202018/Hassan%20Submission%20PLosOne/content.html#lcl|NZ_CP012938.1_prot_WP_004295744.1_907) |  |  |  |  |  | + |  | [2.A.1.46.5](http://tcdb.org/search/result.php?tc=2.A.1.46.5) | -17 | C2UR80 | Drugs | Quinolones |
| [WP_005846035.1](file:///Volumes/ESD-USB/Summer%202018/Library/Application%20Support/Microsoft/Office/Office%202011%20AutoRecovery/content.html#lcl|NC_009614.1_prot_WP_005846035.1_2189) |  |  |  |  |  |  | + | [2.A.1.46.5](http://tcdb.org/search/result.php?tc=2.A.1.46.5) | -65 | C2UR80 | Drugs | Fluoroquinolones |
| [WP_005843278.1](file:///Volumes/ESD-USB/Summer%202018/Library/Application%20Support/Microsoft/Office/Office%202011%20AutoRecovery/content.html#lcl|NC_009614.1_prot_WP_005843278.1_2695) |  |  |  |  |  |  | + | [2.A.1.46.5](http://tcdb.org/search/result.php?tc=2.A.1.46.5) | -13 | C2UR80 | Drugs | Fluoroquinolones |
| ABI39_21205 | + |  |  |  |  |  |  | 2.A.1.49.7 | -23 | Q1D0C6 | Sugar alcohol | Sphingosine |
| [WP_004300603.1](file:///Volumes/ESD-USB/Summer%202018/Hassan%20Submission%20PLosOne/content.html#lcl|NZ_CP012938.1_prot_WP_004300603.1_1480) |  |  |  |  |  | + |  | [2.A.1.49.7](http://tcdb.org/search/result.php?tc=2.A.1.49.7) | 27 | Q1D0C6 | Amines | Sphingosine |
| YP_101211.1 |  |  |  |  | + |  |  | 2.A.1.49.7 | -24 | Q1D0C6 | Sugar alcohols | Sphingosine |
| YP_098989.1 |  |  |  |  | + |  |  | 2.A.1.49.7 | -22 | Q1D0C6 | Sugar alcohols | Sphingosine |
| YP_101521.1 |  |  |  |  | + |  |  | 2.A.1.49.7 | -21 | Q1D0C6 | Sugar alcohols | Sphingosine |
| [WP_005839713.1](file:///Volumes/ESD-USB/Summer%202018/Library/Application%20Support/Microsoft/Office/Office%202011%20AutoRecovery/content.html#lcl|NC_009614.1_prot_WP_005839713.1_4021) |  |  |  |  |  |  | + | [2.A.1.49.7](http://tcdb.org/search/result.php?tc=2.A.1.49.7) | -23 | Q1D0C6 | Amines | Sphingosine |
| [WP_004296058.1](file:///Volumes/ESD-USB/Summer%202018/Hassan%20Submission%20PLosOne/content.html#lcl|NZ_CP012938.1_prot_WP_004296058.1_631) |  |  |  |  |  | + |  | [2.A.1.49.8](http://tcdb.org/search/result.php?tc=2.A.1.49.8) | -24 | E8UWW5 | Amines | Sphingosine |
| ABI39_08305 | + |  |  |  |  |  |  | 2.A.1.53.5 | -41 | Q9VS47 | Amino Acids | Amino Acids |
| [WP_013617878.1](file:///Volumes/ESD-USB/Summer%202018/Hassan%20Submission%20PLosOne/content.html#lcl|NC_015164.1_prot_WP_013617878.1_1851) |  | + |  |  |  |  |  | [2.A.1.53.5](http://tcdb.org/search/result.php?tc=2.A.1.53.5) | -36 | Q9VS47 | Amino Acids | Amino Acids |
| [WP_029428635.1](file:///Volumes/ESD-USB/Summer%202018/Hassan%20Submission%20PLosOne/content.html#lcl|NZ_CP012801.1_prot_WP_029428635.1_4285) |  |  | + |  |  |  |  | [2.A.1.53.5](http://tcdb.org/search/result.php?tc=2.A.1.53.5) | -41 | Q9VS47 | Amino acids | Amino Acids |
| WP_008764538.1 |  |  |  | + |  |  |  | 2.A.1.53.5 | -41 | Q9VS47 | Unknown | Unknown |
| YP_098369.1_1086 |  |  |  |  | + |  |  | 2.A.1.53.5 | -43 | Q9VS47 | Amino acids | Amino acids |
| [WP_005838636.1](file:///Volumes/ESD-USB/Summer%202018/Library/Application%20Support/Microsoft/Office/Office%202011%20AutoRecovery/content.html#lcl|NC_009614.1_prot_WP_005838636.1_1663) |  |  |  |  |  |  | + | [2.A.1.53.5](http://tcdb.org/search/result.php?tc=2.A.1.53.5) | -40 | Q9VS47 | Amino Acids | Amino Acids |
| YP_099197.1 |  |  |  |  | + |  |  | 2.A.1.57.4 | -39 | Q1D1G6 | Siderophores | Siderophores |
| [WP_013618209.1](file:///Volumes/ESD-USB/Summer%202018/Hassan%20Submission%20PLosOne/content.html#lcl|NC_015164.1_prot_WP_013618209.1_2187) |  | + |  |  |  |  |  | [2.A.1.60.3](http://tcdb.org/search/result.php?tc=2.A.1.60.3) | -73 | F2WVP9 | Drugs | Fluoroquinolones/Tetracylcines |
| WP_062695103.1 |  |  |  | + |  |  |  | 2.A.1.60.3 | -34 | F2WVP9 | Drugs | Tetracycline/Fluoroquinolones |
| [WP_004296024.1](file:///Volumes/ESD-USB/Summer%202018/Hassan%20Submission%20PLosOne/content.html#lcl|NZ_CP012938.1_prot_WP_004296024.1_663) |  |  |  |  |  | + |  | [2.A.1.60.3](http://tcdb.org/search/result.php?tc=2.A.1.60.3) | -33 | F2WVP9 | Drugs | Tetracyclines/Fluoroquinolones |
| ABI39_20125 | + |  |  |  |  |  |  | 2.A.1.68.1 | -17 | Q04DP6 | Sugars | Glucose |
| [WP_007209676.1](file:///Volumes/ESD-USB/Summer%202018/Hassan%20Submission%20PLosOne/content.html#lcl|NZ_CP012801.1_prot_WP_007209676.1_4909) |  |  | + |  |  |  |  | [2.A.1.68.1](http://tcdb.org/search/result.php?tc=2.A.1.68.1) | -17 | Q04DP6 | Unknown | Unknown |
| WP_011108356.1 |  |  |  | + |  |  |  | 2.A.1.68.1 | -13 | Q04DP6 | Sugars | Glucose |
| YP_097735.1_452 |  |  |  |  | + |  |  | 2.A.1.68.1 | -14 | Q04DP6 | Sugars | Glucose |
| [WP_004296267.1](file:///Volumes/ESD-USB/Summer%202018/Hassan%20Submission%20PLosOne/content.html#lcl|NZ_CP012938.1_prot_WP_004296267.1_2581) |  |  |  |  |  | + |  | [2.A.1.68.1](http://tcdb.org/search/result.php?tc=2.A.1.68.1) | -12 | Q04DP6 | Sugars | Glucose |
| [WP_005851602.1](file:///Volumes/ESD-USB/Summer%202018/Library/Application%20Support/Microsoft/Office/Office%202011%20AutoRecovery/content.html#lcl|NC_009614.1_prot_WP_005851602.1_3797) |  |  |  |  |  |  | + | [2.A.1.68.1](http://tcdb.org/search/result.php?tc=2.A.1.68.1) | -18 | Q04DP6 | Sugars | Glucose |
| [WP_004297806.1](file:///Volumes/ESD-USB/Summer%202018/Hassan%20Submission%20PLosOne/content.html#lcl|NZ_CP012938.1_prot_WP_004297806.1_2397) |  |  |  |  |  | + |  | [2.A.2.3.2](http://tcdb.org/search/result.php?tc=2.A.2.3.2) | -131 | P94488 | Unknown | Unknown |
| ABI39_00195 | + |  |  |  |  |  |  | 2.A.2.3.4 | -78 | P31435 | Sugar derivatives | Xylosides |
| [WP_007212789.1](file:///Volumes/ESD-USB/Summer%202018/Hassan%20Submission%20PLosOne/content.html#lcl|NZ_CP012801.1_prot_WP_007212789.1_4133) |  |  | + |  |  |  |  | [2.A.2.3.4](http://tcdb.org/search/result.php?tc=2.A.2.3.4) | -83 | P31435 | Sugar derivatives | Xyloside |
| [WP_008782290.1](file:///Volumes/ESD-USB/Summer%202018/Library/Application%20Support/Microsoft/Office/Office%202011%20AutoRecovery/content.html#lcl|NC_009614.1_prot_WP_008782290.1_45) |  |  |  |  |  |  | + | [2.A.2.3.4](http://tcdb.org/search/result.php?tc=2.A.2.3.4) | -77 | P31435 | Sugar Derivatives | Xyloside |
| [WP_013618704.1](file:///Volumes/ESD-USB/Summer%202018/Hassan%20Submission%20PLosOne/content.html#lcl|NC_015164.1_prot_WP_013618704.1_2744) |  | + |  |  |  |  |  | [2.A.2.3.5](http://tcdb.org/search/result.php?tc=2.A.2.3.5) | -73 | P75683 | Sugar Derivatives | Xyloside |
| [WP_029429004.1](file:///Volumes/ESD-USB/Summer%202018/Hassan%20Submission%20PLosOne/content.html#lcl|NZ_CP012801.1_prot_WP_029429004.1_1917) |  |  | + |  |  |  |  | [2.A.2.3.5](http://tcdb.org/search/result.php?tc=2.A.2.3.5) | -83 | P75683 | Sugar Derivatives | Xyloside |
| YP_098133.1_850 |  |  |  |  | + |  |  | 2.A.2.3.5 | -97 | P75683 | Sugar Derivatives | Xylosides |
| [WP_013617710.1](file:///Volumes/ESD-USB/Summer%202018/Hassan%20Submission%20PLosOne/content.html#lcl|NC_015164.1_prot_WP_013617710.1_1680) |  | + |  |  |  |  |  | [2.A.2.3.6](http://tcdb.org/search/result.php?tc=2.A.2.3.6) | -87 | A1S5F2 | Sugar Derivatives | Cellobiose |
| [WP_004298410.1](file:///Volumes/ESD-USB/Summer%202018/Hassan%20Submission%20PLosOne/content.html#lcl|NZ_CP012938.1_prot_WP_004298410.1_2902) |  |  |  |  |  | + |  | [2.A.2.3.6](http://tcdb.org/search/result.php?tc=2.A.2.3.6) | -98 | A1S5F2 | Sugar Derivatives | Cellobiose |
| [WP_004296492.1](file:///Volumes/ESD-USB/Summer%202018/Hassan%20Submission%20PLosOne/content.html#lcl|NZ_CP012938.1_prot_WP_004296492.1_2778) |  |  |  |  |  | + |  | [2.A.2.3.6](http://tcdb.org/search/result.php?tc=2.A.2.3.6) | -97 | A1S5F2 | Sugar Derivatives | Cellobiose |
| ABI39_04405 | + |  |  |  |  |  |  | 2.A.2.6.2 | -72 | Q9A612 | Sugars | Maltose |
| [WP_013616372.1](file:///Volumes/ESD-USB/Summer%202018/Hassan%20Submission%20PLosOne/content.html#lcl|NC_015164.1_prot_WP_013616372.1_297) |  | + |  |  |  |  |  | [2.A.2.6.2](http://tcdb.org/search/result.php?tc=2.A.2.6.2) | -72 | Q9A612 | Sugars | Maltose |
| [WP_029428312.1](file:///Volumes/ESD-USB/Summer%202018/Hassan%20Submission%20PLosOne/content.html#lcl|NZ_CP012801.1_prot_WP_029428312.1_1234) |  |  | + |  |  |  |  | [2.A.2.6.2](http://tcdb.org/search/result.php?tc=2.A.2.6.2) | -74 | Q9A612 | Sugars | Maltose |
| [WP_005849623.1](file:///Volumes/ESD-USB/Summer%202018/Library/Application%20Support/Microsoft/Office/Office%202011%20AutoRecovery/content.html#lcl|NC_009614.1_prot_WP_005849623.1_1369) |  |  |  |  |  |  | + | [2.A.2.6.2](http://tcdb.org/search/result.php?tc=2.A.2.6.2) | -73 | Q9A612 | Sugars | Maltose |
| ABI39_06105 | + |  |  |  |  |  |  | 2.A.4.7.9 | -51 | Q1LHU8 | Cations | Zn^2+/^Co^2+^/Cd^2+^ |
| [WP_005845172.1](file:///Volumes/ESD-USB/Summer%202018/Library/Application%20Support/Microsoft/Office/Office%202011%20AutoRecovery/content.html#lcl|NC_009614.1_prot_WP_005845172.1_315) |  |  |  |  |  |  | + | [2.A.6.2.16](http://tcdb.org/search/result.php?tc=2.A.6.2.16) | 0 | Q9I0Y8 | Drugs | Fluoroquinolones, chloramphenicol, biocides, xenobiotics |
| WP_011108360.1 |  |  |  | + |  |  |  | 2.A.3.7.1 | -66 | O30417 | Amino acids | Glutamic acid |
| [WP_041584069.1](file:///Volumes/ESD-USB/Summer%202018/Hassan%20Submission%20PLosOne/content.html#lcl|NC_015164.1_prot_WP_041584069.1_2962) |  | + |  |  |  |  |  | [2.A.3.3.22](http://tcdb.org/search/result.php?tc=2.A.3.3.22) | -126 | Q8R706 | Amino Acids | Amino Acids |
| [WP_013616756.1](file:///Volumes/ESD-USB/Summer%202018/Hassan%20Submission%20PLosOne/content.html#lcl|NC_015164.1_prot_WP_013616756.1_696) |  | + |  |  |  |  |  | [2.A.4.1.3](http://tcdb.org/search/result.php?tc=2.A.4.1.3) | -56 | O07084 | Cations | Cd^2+^/Zn^2+^ |
| [WP_007214988.1](file:///Volumes/ESD-USB/Summer%202018/Hassan%20Submission%20PLosOne/content.html#lcl|NZ_CP012801.1_prot_WP_007214988.1_1395) |  |  | + |  |  |  |  | [2.A.4.1.3](http://tcdb.org/search/result.php?tc=2.A.4.1.3) | -63 | O07084 | Cations | Cd^2+^/Zn^2+^ |
| WP_008765023.1 |  |  |  | + |  |  |  | 2.A.4.1.3 | -59 | O07084 | Cations | Cd^2+^/Zn^2+^/H^+^/K^+^ |
| [WP_004300142.1](file:///Volumes/ESD-USB/Summer%202018/Hassan%20Submission%20PLosOne/content.html#lcl|NZ_CP012938.1_prot_WP_004300142.1_1202) |  |  |  |  |  | + |  | [2.A.4.1.3](http://tcdb.org/search/result.php?tc=2.A.4.1.3) | -57 | O07084 | Cations | Mn^2+^ |
| WP_008763494.1 |  |  |  | + |  |  |  | 2.A.4.7.6 | -35 | Q97PQ1 | Cations | Mn^2+^ |
| [WP_004300135.1](file:///Volumes/ESD-USB/Summer%202018/Hassan%20Submission%20PLosOne/content.html#lcl|NZ_CP012938.1_prot_WP_004300135.1_1196) |  |  |  |  |  | + |  | [2.A.4.7.6](http://tcdb.org/search/result.php?tc=2.A.4.7.6) | -35 | Q97PQ1 | Cations | Mn^2+^ |
| [WP_013618422.1](file:///Volumes/ESD-USB/Summer%202018/Hassan%20Submission%20PLosOne/content.html#lcl|NC_015164.1_prot_WP_013618422.1_2418) |  | + |  |  |  |  |  | [2.A.4.7.9](http://tcdb.org/search/result.php?tc=2.A.4.7.9) | -52 | Q1LHU8 | Unknown | Unknown |
| [WP_007211562.1](file:///Volumes/ESD-USB/Summer%202018/Hassan%20Submission%20PLosOne/content.html#lcl|NZ_CP012801.1_prot_WP_007211562.1_2811) |  |  | + |  |  |  |  | [2.A.4.7.9](http://tcdb.org/search/result.php?tc=2.A.4.7.9) | -52 | Q1LHU8 | Cations | Cd^2+^/Zn^2+^ |
| [WP_005850066.](file:///Volumes/ESD-USB/Summer%202018/Library/Application%20Support/Microsoft/Office/Office%202011%20AutoRecovery/content.html#lcl|NC_009614.1_prot_WP_005850066.1_1071) 1 |  |  |  |  |  |  | + | [2.A.4.7.9](http://tcdb.org/search/result.php?tc=2.A.4.7.9) | -51 | Q1LHU8 | Cations | Zn^2+^/Cd^2+^/Pb^2+^ |
| WP_004309915.1 |  |  |  | + |  |  |  | 2.A.5.5.5 | -81 | B2UL32 | Cations | Zn^2+^ |
| WP_004309915.1 |  |  |  | + |  |  |  | 2.A.5.5.5 | -81 | B2UL32 | Cations | Zn^2+^ |
| WP_004309915.1 |  |  |  | + |  |  |  | 2.A.5.5.5 | -81 | B2UL32 | Cations | Zn^2+^ |
| WP_062694353.1 |  |  |  | + |  |  |  | 2.A.6.1.2 | 0 | P13511 | Heavy metals | Co, Zn, Cd |
| YP_101164.1 |  |  |  |  | + |  |  | 2.A.6.1.2 | 0 | P13511 | Metals | Zinc/Cobalt |
| ABI39_10335 | + |  |  |  |  |  |  | 2.A.6.1.5 | 0 | Q88RT6 | Cations | Zn^2+/^Cd^2+^/Pb^2+^ |
| ABI39_05385 | + |  |  |  |  |  |  | 2.A.6.1.5 | -14 | Q88RT4 | Cations | Zn^2+^/Cd^2+^ |
| [WP_013617326.1](file:///Volumes/ESD-USB/Summer%202018/Hassan%20Submission%20PLosOne/content.html#lcl|NC_015164.1_prot_WP_013617326.1_1294) |  | + |  |  |  |  |  | [2.A.6.1.5](http://tcdb.org/search/result.php?tc=2.A.6.1.5) | 0 | Q88RT6 | Cations | Co^2+^/Zn^2+^/Cd^2+^ |
| [WP_029429154.1](file:///Volumes/ESD-USB/Summer%202018/Hassan%20Submission%20PLosOne/content.html#lcl|NZ_CP012801.1_prot_WP_029429154.1_1431) |  |  | + |  |  |  |  | [2.A.6.1.5](http://tcdb.org/search/result.php?tc=2.A.6.1.5) | 0 | Q88RT6 | Cations | Zn^2+/^Cd^2+/^Pb^2+^ |
| [WP_029428059.1](file:///Volumes/ESD-USB/Summer%202018/Hassan%20Submission%20PLosOne/content.html#lcl|NZ_CP012801.1_prot_WP_029428059.1_5237) |  |  | + |  |  |  |  | [2.A.6.1.5](http://tcdb.org/search/result.php?tc=2.A.6.1.5) | -26 | Q88RT4 | Cations | Zn^2+^/Cd^2+^/Pb^2+^ |
| [WP_007211957.1](file:///Volumes/ESD-USB/Summer%202018/Hassan%20Submission%20PLosOne/content.html#lcl|NZ_CP012801.1_prot_WP_007211957.1_1944) |  |  | + |  |  |  |  | [2.A.6.1.5](http://tcdb.org/search/result.php?tc=2.A.6.1.5) | -12 | Q88RT5 | Cations | Zn^2+^/Cd^2+^/Pb^2+^ |
| WP_062694599.1 |  |  |  | + |  |  |  | 2.A.6.1.5 | 0 | Q88RT6 | Cations | Zn^2+^/Cd^2+^/Pb^2+^ |
| WP_008766787.1 |  |  |  | + |  |  |  | 2.A.6.1.5 | -17 | Q88RT4 | Cations | Zn2+/Cd2+Pb^2+^ |
| WP_008765561.1 |  |  |  | + |  |  |  | 2.A.6.1.5 | -12 | Q88RT4 | Cations | Zn^2+^/Cd^2+^/Pb^2+^ |
| YP_099373.1 |  |  |  |  | + |  |  | 2.A.6.1.5 | 0 | Q88RT6 | Cations | Zn^2+^/Cd^2+^/Pb^2+^ |
| YP_100999.1 |  |  |  |  | + |  |  | 2.A.6.1.5 | 0 | Q88RT6 | Cations | Zn^2+^/Cd^2+^/Pb^2+^ |
| YP_099463.1 |  |  |  |  | + |  |  | 2.A.6.1.5 | -12 | Q88RT4 | Cations | Zn^2+^/Cd^2+^/Pb^2+^ |
| [WP_011965418.1](file:///Volumes/ESD-USB/Summer%202018/Library/Application%20Support/Microsoft/Office/Office%202011%20AutoRecovery/content.html#lcl|NC_009614.1_prot_WP_011965418.1_2004) |  |  |  |  |  |  | + | [2.A.6.1.5](http://tcdb.org/search/result.php?tc=2.A.6.1.5) | 0 | Q88RT6 | Cations | Zn2+/Cd2+/Pb^2+^ |
| [WP_029428060.1](file:///Volumes/ESD-USB/Summer%202018/Hassan%20Submission%20PLosOne/content.html#lcl|NZ_CP012801.1_prot_WP_029428060.1_5236) |  |  | + |  |  |  |  | [2.A.6.1.6](http://tcdb.org/search/result.php?tc=2.A.6.1.6) | 0 | Q1LCD8 | Cations | Zn^2+^ |
| WP_008766788.1 |  |  |  | + |  |  |  | 2.A.6.1.6 | 0 | Q1LCD8 | Cations | Zn^2+^ |
| WP_008766789.1 |  |  |  | + |  |  |  | 2.A.6.1.6 | -37 | Q1LCD7 | Cations | Zn^2+^ |
| [WP_004297968.1](file:///Volumes/ESD-USB/Summer%202018/Hassan%20Submission%20PLosOne/content.html#lcl|NZ_CP012938.1_prot_WP_004297968.1_1976) |  |  |  |  |  | + |  | [2.A.6.1.6](http://tcdb.org/search/result.php?tc=2.A.6.1.6) | -12 | Q1LCD7 | Nucleosides | Xantosine |
| WP_062694351.1 |  |  |  | + |  |  |  | 2.A.6.1.7 | -18 | Q1DDM8 | Cations | Zn^2+^ |
| WP_008766452.1 |  |  |  | + |  |  |  | 2.A.6.1.7 | -13 | Q1DDM8 | Cations | Zn^2+^ |
| [WP_004301142.1](file:///Volumes/ESD-USB/Summer%202018/Hassan%20Submission%20PLosOne/content.html#lcl|NZ_CP012938.1_prot_WP_004301142.1_3946) |  |  |  |  |  | + |  | [2.A.6.1.7](http://tcdb.org/search/result.php?tc=2.A.6.1.7) | -16 | Q1DDM8 | Cations | Mn^2+^ |
| [WP_033160444.1](file:///Volumes/ESD-USB/Summer%202018/Hassan%20Submission%20PLosOne/content.html#lcl|NZ_CP012801.1_prot_WP_033160444.1_3169) |  |  | + |  |  |  |  | [2.A.6.1.8](http://tcdb.org/search/result.php?tc=2.A.6.1.8) | -29 | Q1DDM3 | Cations | Cu^2+^ |
| WP_008765562.1 |  |  |  | + |  |  |  | 2.A.6.1.8 | -27 | Q1DDM3 | Unknown | Unknown |
| YP_100838.1 |  |  |  |  | + |  |  | 2.A.6.1.8 | -78 | Q1DDM3 | Cations | Cu^2+^ |
| YP_099464.1_ |  |  |  |  | + |  |  | 2.A.6.1.8 | -32 | Q1DDM3 | Cations | Cu^2+^ |
| [WP_004295723.1](file:///Volumes/ESD-USB/Summer%202018/Hassan%20Submission%20PLosOne/content.html#lcl|NZ_CP012938.1_prot_WP_004295723.1_927) |  |  |  |  |  | + |  | [2.A.6.1.8](http://tcdb.org/search/result.php?tc=2.A.6.1.8) | -32 | Q1DDM3 | Unknown | Unknown |
| WP_011107845.1 |  |  |  | + |  |  |  | 2.A.6.1.13 | -09 | Q9RLI9 | Unknown | Unknown |
| ABI39_05375 | + |  |  |  |  |  |  | 2.A.6.1.17 | 0 | Q1LCE8 | Cations | Zn^2+^/Cd^2+^ |
| [WP_029426627.1](file:///Volumes/ESD-USB/Summer%202018/Hassan%20Submission%20PLosOne/content.html#lcl|NZ_CP012801.1_prot_WP_029426627.1_3170) |  |  | + |  |  |  |  | [2.A.6.1.17](http://tcdb.org/search/result.php?tc=2.A.6.1.17) | 0 | Q1LCE8 | Cations | Zn^2+^ |
| WP_008765563.1 |  |  |  | + |  |  |  | 2.A.6.1.17 | 0 | Q1LCE8 | Cations | Zn^2+^ |
| ABI39_01290 | + |  |  |  |  |  |  | 2.A.6.2.4 | -45 | Q67GM1 | Drugs | Amikacin, ceftazidime, chloramphenicol, ciprofloxacin, erythromycin, ethidium bromide, meropenem, rifampin, and tetracycline |
| WP_048696050.1 |  |  |  | + |  |  |  | 2.A.6.2.6 | 0 | P52002 | Drugs | Quinolones, Macrolides, Tetracycline |
| WP_011107941.1 |  |  |  | + |  |  |  | 2.A.6.2.11 | -18 | Q93PU5 | Organic solvents | Toluene/Styrene |
| ABI39_01690 | + |  |  |  |  |  |  | 2.A.6.2.16 | 0 | Q9I0Y8 | Drugs | Fluoroquinolones/Chloramphenicol |
| [WP_029428128.1](file:///Volumes/ESD-USB/Summer%202018/Hassan%20Submission%20PLosOne/content.html#lcl|NZ_CP012801.1_prot_WP_029428128.1_5133) |  |  | + |  |  |  |  | [2.A.6.2.16](http://tcdb.org/search/result.php?tc=2.A.6.2.16) | 0 | Q9I0Y8 | Unknown | Quinolones, Chloramphenicol |
| WP_061474265.1 |  |  |  | + |  |  |  | 2.A.6.2.16 | 0 | Q9I0Y8 | Drugs | Quinolones, Chloramphenicol |
| YP_099676.1 |  |  |  |  | + |  |  | 2.A.6.2.16 | 0 | Q9I0Y8 | Drugs | Quinolones/Chloramphenicol |
| [WP_013617881.1](file:///Volumes/ESD-USB/Summer%202018/Hassan%20Submission%20PLosOne/content.html#lcl|NC_015164.1_prot_WP_013617881.1_1854) |  | + |  |  |  |  |  | [2.A.6.2.17](http://tcdb.org/search/result.php?tc=2.A.6.2.17) | -106 | Q9HXW4 | Drugs | Fluoroquinolones, tetracycline, macrolides |
| [WP_029427895.1](file:///Volumes/ESD-USB/Summer%202018/Hassan%20Submission%20PLosOne/content.html#lcl|NZ_CP012801.1_prot_WP_029427895.1_1560) |  |  | + |  |  |  |  | [2.A.6.2.17](http://tcdb.org/search/result.php?tc=2.A.6.2.17) | -102 | Q9HXW4 | Unknown | Unknown |
| YP_099465.1 |  |  |  |  | + |  |  | 2.A.6.1.17 | 0 | Q1LCE8 | Cations | Zn^2+^ |
| ABI39_16570 | + |  |  |  |  |  |  | 2.A.6.2.20 | -07 | Q4VSJ3 | Drugs | Toxoflavin |
| [WP_013617664.1](file:///Volumes/ESD-USB/Summer%202018/Hassan%20Submission%20PLosOne/content.html#lcl|NC_015164.1_prot_WP_013617664.1_1632) |  | + |  |  |  |  |  | [2.A.6.2.20](http://tcdb.org/search/result.php?tc=2.A.6.2.20) | -34 | Q4VSJ3 | Drugs | Toxoflavin |
| [WP_013617327.1](file:///Volumes/ESD-USB/Summer%202018/Hassan%20Submission%20PLosOne/content.html#lcl|NC_015164.1_prot_WP_013617327.1_1295) |  | + |  |  |  |  |  | [2.A.6.2.20](http://tcdb.org/search/result.php?tc=2.A.6.2.20) | -14 | Q4VSJ3 | Drugs | Toxoflavin |
| [WP_007213328.1](file:///Volumes/ESD-USB/Summer%202018/Hassan%20Submission%20PLosOne/content.html#lcl|NZ_CP012801.1_prot_WP_007213328.1_3661) |  |  | + |  |  |  |  | [2.A.6.2.20](http://tcdb.org/search/result.php?tc=2.A.6.2.20) | -37 | Q4VSJ3 | Drugs | Toxoflavin |
| [WP_026367244.1](file:///Volumes/ESD-USB/Summer%202018/Hassan%20Submission%20PLosOne/content.html#lcl|NZ_CP012801.1_prot_WP_026367244.1_3294) |  |  | + |  |  |  |  | [2.A.6.2.20](http://tcdb.org/search/result.php?tc=2.A.6.2.20) | -20 | Q4VSJ3 | Drugs | Toxoflavin |
| WP_062694297.1 |  |  |  | + |  |  |  | 2.A.6.2.20 | -22 | Q4VSJ3 | Drugs | Toxoflavin |
| WP_008760595.1 |  |  |  | + |  |  |  | 2.A.6.2.20 | -19 | Q4VSJ3 | Drugs | Toxoflavin |
| [WP_004296311.1](file:///Volumes/ESD-USB/Summer%202018/Hassan%20Submission%20PLosOne/content.html#lcl|NZ_CP012938.1_prot_WP_004296311.1_2613) |  |  |  |  |  | + |  | [2.A.6.2.20](http://tcdb.org/search/result.php?tc=2.A.6.2.20) | -21 | Q4VSJ3 | Drugs | Toxoflavin |
| [WP_005845555.1](file:///Volumes/ESD-USB/Summer%202018/Library/Application%20Support/Microsoft/Office/Office%202011%20AutoRecovery/content.html#lcl|NC_009614.1_prot_WP_005845555.1_233) |  |  |  |  |  |  | + | [2.A.6.2.20](http://tcdb.org/search/result.php?tc=2.A.6.2.20) | -19 | Q4VSJ3 | Drugs | Toxoflavin |
| [WP_012055571.1](file:///Volumes/ESD-USB/Summer%202018/Library/Application%20Support/Microsoft/Office/Office%202011%20AutoRecovery/content.html#lcl|NC_009614.1_prot_WP_012055571.1_3115) |  |  |  |  |  |  | + | [2.A.6.2.20](http://tcdb.org/search/result.php?tc=2.A.6.2.20) | -06 | Q4VSJ3 | Drugs | Toxoflavin |
| [WP_013617557.1](file:///Volumes/ESD-USB/Summer%202018/Hassan%20Submission%20PLosOne/content.html#lcl|NC_015164.1_prot_WP_013617557.1_1523) |  | + |  |  |  |  |  | [2.A.6.2.21](http://tcdb.org/search/result.php?tc=2.A.6.2.21) | -54 | Q51487 | Drugs | fluoroquinolones, tetracycline, macrolides |
| [WP_029426692.1](file:///Volumes/ESD-USB/Summer%202018/Hassan%20Submission%20PLosOne/content.html#lcl|NZ_CP012801.1_prot_WP_029426692.1_3315) |  |  | + |  |  |  |  | [2.A.6.2.21](http://tcdb.org/search/result.php?tc=2.A.6.2.21) | 55 | Q51487 | Drugs | Fluoroquinolones/Macrolides/Chloramphenicol |
| [WP_007211003.1](file:///Volumes/ESD-USB/Summer%202018/Hassan%20Submission%20PLosOne/content.html#lcl|NZ_CP012801.1_prot_WP_007211003.1_2386) |  |  | + |  |  |  |  | [2.A.6.2.21](http://tcdb.org/search/result.php?tc=2.A.6.2.21) | -55 | Q51487 | Drugs | Fluoroquinolones/Macrolides/Chloramphenicol |
| WP_008767546.1 |  |  |  | + |  |  |  | 2.A.6.2.21 | -53 | Q51487 | Drugs | Aminoglycosides/Lactams/Fluoroquinolones |
| WP_062694194.1 |  |  |  | + |  |  |  | 2.A.6.2.21 | -48 | Q51487 | Drugs | Aminoglycosides/Lactams/Fluoroquinolones |
| WP_011107843.1 |  |  |  | + |  |  |  | 2.A.6.2.21 | -07 | Q51487 | Drugs | Aminoglycosides/Lactams/Fluoroquinolones |
| [WP_004324284.1](file:///Volumes/ESD-USB/Summer%202018/Hassan%20Submission%20PLosOne/content.html#lcl|NZ_CP012938.1_prot_WP_004324284.1_3300) |  |  |  |  |  | + |  | [2.A.6.2.21](http://tcdb.org/search/result.php?tc=2.A.6.2.21) | -49 | Q51487 | Drugs | Aminoglycosides/Macrolides/Fluoroquinolones |
| [WP_052587944.1](file:///Volumes/ESD-USB/Summer%202018/Hassan%20Submission%20PLosOne/content.html#lcl|NZ_CP012938.1_prot_WP_052587944.1_3102) |  |  |  |  |  | + |  | [2.A.6.2.21](http://tcdb.org/search/result.php?tc=2.A.6.2.21) | -48 | Q51487 | Drugs | Aminoglycosides/Macrolides/Fluoroquinolones |
| ABI39_01285 | + |  |  |  |  |  |  | 2.A.6.2.22 | 0 | Q8RTE4 | Drugs | Fluoroquinolones/Aminoglycosides/Ethidium |
| ABI39_08285 | + |  |  |  |  |  |  | 2.A.6.2.22 | 0 | Q8RTE4 | Drugs | Fluoroquinolones/Aminoglycosides/Ethidium |
| ABI39_18005 | + |  |  |  |  |  |  | 2.A.6.2.22 | 0 | Q8RTE4 | Drugs | Fluoroquinolones/Aminoglycosides/Ethidium |
| ABI39_18725 | + |  |  |  |  |  |  | 2.A.6.2.22 | 0 | Q8RTE4 | Drugs | Fluoroquinolones/Aminoglycosides/Ethidium |
| [WP_013617937.1](file:///Volumes/ESD-USB/Summer%202018/Hassan%20Submission%20PLosOne/content.html#lcl|NC_015164.1_prot_WP_013617937.1_1913) |  | + |  |  |  |  |  | [2.A.6.2.22](http://tcdb.org/search/result.php?tc=2.A.6.2.22) | 0 | Q8RTE4 | Drugs | Fluoroquinoloes/Tetracylines/Macrolides |
| [WP_013617558.1](file:///Volumes/ESD-USB/Summer%202018/Hassan%20Submission%20PLosOne/content.html#lcl|NC_015164.1_prot_WP_013617558.1_1524) |  | + |  |  |  |  |  | [2.A.6.2.22](http://tcdb.org/search/result.php?tc=2.A.6.2.22) | 0 | Q8RTE4 | Drugs | Fluoroquinoloes/Tetracylines/Macrolides |
| [WP_013617334.1](file:///Volumes/ESD-USB/Summer%202018/Hassan%20Submission%20PLosOne/content.html#lcl|NC_015164.1_prot_WP_013617334.1_1302) |  | + |  |  |  |  |  | [2.A.6.2.22](http://tcdb.org/search/result.php?tc=2.A.6.2.22) | 0 | Q8RTE4 | Drugs | Fluoroquinoloes/Tetracylines/Macrolides |
| [WP_029426171.1](file:///Volumes/ESD-USB/Summer%202018/Hassan%20Submission%20PLosOne/content.html#lcl|NZ_CP012801.1_prot_WP_029426171.1_2385) |  |  | + |  |  |  |  | [2.A.6.2.22](http://tcdb.org/search/result.php?tc=2.A.6.2.22) | 0 | Q8RTE4 | Drugs | Fluroquinolones/Aminoglycosides |
| [WP_029427108.1](file:///Volumes/ESD-USB/Summer%202018/Hassan%20Submission%20PLosOne/content.html#lcl|NZ_CP012801.1_prot_WP_029427108.1_3973) |  |  | + |  |  |  |  | [2.A.6.2.22](http://tcdb.org/search/result.php?tc=2.A.6.2.22) | 0 | Q8RTE4 | Drugs | Fluroquinolones/Aminoglycosides |
| [WP_007219592.1](file:///Volumes/ESD-USB/Summer%202018/Hassan%20Submission%20PLosOne/content.html#lcl|NZ_CP012801.1_prot_WP_007219592.1_3314) |  |  | + |  |  |  |  | [2.A.6.2.22](http://tcdb.org/search/result.php?tc=2.A.6.2.22) | 0 | Q8RTE4 | Drugs | Fluroquinolones/Aminoglycosides |
| [WP_025725834.1](file:///Volumes/ESD-USB/Summer%202018/Hassan%20Submission%20PLosOne/content.html#lcl|NZ_CP012801.1_prot_WP_025725834.1_1836) |  |  | + |  |  |  |  | [2.A.6.2.22](http://tcdb.org/search/result.php?tc=2.A.6.2.22) | 0 | Q8RTE4 | Drugs | Fluroquinolones/Aminoglycosides |
| [WP_029428048.1](file:///Volumes/ESD-USB/Summer%202018/Hassan%20Submission%20PLosOne/content.html#lcl|NZ_CP012801.1_prot_WP_029428048.1_5256) |  |  | + |  |  |  |  | [2.A.6.2.22](http://tcdb.org/search/result.php?tc=2.A.6.2.22) | 0 | Q8RTE4 | Drugs | Fluroquinolones/Aminoglycosides |
| [WP_007212270.1](file:///Volumes/ESD-USB/Summer%202018/Hassan%20Submission%20PLosOne/content.html#lcl|NZ_CP012801.1_prot_WP_007212270.1_3688) |  |  | + |  |  |  |  | [2.A.6.2.22](http://tcdb.org/search/result.php?tc=2.A.6.2.22) | 0 | Q8RTE4 | Drugs | Fluroquinolones/Aminoglycosides |
| WP_008767547.1 |  |  |  | + |  |  |  | 2.A.6.2.22 | 0 | Q8RTE4 | Drugs | Tetracycline, fluoroquinolones |
| WP_016269621.1 |  |  |  | + |  |  |  | 2.A.6.2.22 | 0 | Q8RTE4 | Drugs | Tetracycline, fluoroquinolones |
| WP_062695211.1 |  |  |  | + |  |  |  | 2.A.6.2.22 | 0 | Q8RTE4 | Drugs | Fluorouinolones,Tetracylines, Cephalosporins |
| WP_062694612.1 |  |  |  | + |  |  |  | 2.A.6.2.22 | -41 | Q8RTE5 | Drugs | Fluoroquinolones,Tetracylines, Cephalosporins |
| YP_097473.1_190 |  |  |  |  | + |  |  | 2.A.6.2.22 | 0 | Q8RTE4 | Drugs | Fluoroquinolones/Macrolides |
| YP_100820.1_3537 |  |  |  |  | + |  |  | 2.A.6.2.22 | 0 | Q8RTE4 | Drugs | Fluoroquinolones/Macrolides |
| YP_100388.1_3105 |  |  |  |  | + |  |  | 2.A.6.2.22 | 0 | Q8RTE4 | Drugs | Fluoroquinolones/Macrolides |
| [WP_011964673.1](file:///Volumes/ESD-USB/Summer%202018/Library/Application%20Support/Microsoft/Office/Office%202011%20AutoRecovery/content.html#lcl|NC_009614.1_prot_WP_011964673.1_238) |  |  |  |  |  |  | + | [2.A.6.2.22](http://tcdb.org/search/result.php?tc=2.A.6.2.22) | 0 | Q8RTE4 | Drugs | Toxoflavin |
| [WP_011965258.1](file:///Volumes/ESD-USB/Summer%202018/Library/Application%20Support/Microsoft/Office/Office%202011%20AutoRecovery/content.html#lcl|NC_009614.1_prot_WP_011965258.1_1659) |  |  |  |  |  |  | + | [2.A.6.2.22](http://tcdb.org/search/result.php?tc=2.A.6.2.22) | 0 | Q8RTE4 | Drugs | Toxoflavin |
| [WP_008669550.1](file:///Volumes/ESD-USB/Summer%202018/Library/Application%20Support/Microsoft/Office/Office%202011%20AutoRecovery/content.html#lcl|NC_009614.1_prot_WP_008669550.1_3511) |  |  |  |  |  |  | + | [2.A.6.2.22](http://tcdb.org/search/result.php?tc=2.A.6.2.22) | 0 | Q8RTE4 | Drugs | Toxoflavin |
| [WP_012055900.1](file:///Volumes/ESD-USB/Summer%202018/Library/Application%20Support/Microsoft/Office/Office%202011%20AutoRecovery/content.html#lcl|NC_009614.1_prot_WP_012055900.1_3695) |  |  |  |  |  |  | + | [2.A.6.2.22](http://tcdb.org/search/result.php?tc=2.A.6.2.22) | 0 | Q8RTE4 | Drugs | Toxoflavin |
| [WP_032945784.1](file:///Volumes/ESD-USB/Summer%202018/Library/Application%20Support/Microsoft/Office/Office%202011%20AutoRecovery/content.html#lcl|NC_009614.1_prot_WP_032945784.1_1658) |  |  |  |  |  |  | + | [2.A.6.2.22](http://tcdb.org/search/result.php?tc=2.A.6.2.22) | -45 | Q8RTE5 | Drugs | Ethidium, ciprofloxacin, norfloxacin, tetracycline, |
| WP_011107656.1 |  |  |  | + |  |  |  | 2.A.6.2.23 | -19 | Q6VV67 | Drugs | Fluoroquinolones,Tetracylines, Cephalosporins |
| [WP_029428127.1](file:///Volumes/ESD-USB/Summer%202018/Hassan%20Submission%20PLosOne/content.html#lcl|NZ_CP012801.1_prot_WP_029428127.1_5134) |  |  | + |  |  |  |  | [2.A.6.2.25](http://tcdb.org/search/result.php?tc=2.A.6.2.25) | -50 | Q8ZRG8 | Cations | Au^2+^ |
| [WP_012055770.1](file:///Volumes/ESD-USB/Summer%202018/Library/Application%20Support/Microsoft/Office/Office%202011%20AutoRecovery/content.html#lcl|NC_009614.1_prot_WP_012055770.1_3514) |  |  |  |  |  |  | + | [2.A.6.2.25](http://tcdb.org/search/result.php?tc=2.A.6.2.25) | -15 | Q8ZRG8 | Cations | Au^2+^ |
| [WP_029427894.1](file:///Volumes/ESD-USB/Summer%202018/Hassan%20Submission%20PLosOne/content.html#lcl|NZ_CP012801.1_prot_WP_029427894.1_1561) |  |  | + |  |  |  |  | [2.A.6.2.27](http://tcdb.org/search/result.php?tc=2.A.6.2.27) | -20 | Q9I6X5 | Drugs | Triclosan |
| [WP_007210149.1](file:///Volumes/ESD-USB/Summer%202018/Hassan%20Submission%20PLosOne/content.html#lcl|NZ_CP012801.1_prot_WP_007210149.1_1630) |  |  | + |  |  |  |  | [2.A.6.2.27](http://tcdb.org/search/result.php?tc=2.A.6.2.27) | -11 | Q9I6X5 | Drugs | Triclosan |
| WP_008766605.1 |  |  |  | + |  |  |  | 2.A.6.2.27 | -23 | Q9I6X5 | Drugs | Triclosan |
| ABI39_09430 | + |  |  |  |  |  |  | 2.A.6.2.28 | -32 | A0Q8A4 | Unknown | Unknown |
| WP_008764016.1 |  |  |  | + |  |  |  | 2.A.6.2.28 | -38 | A0Q8A4 | Unknown | Unknown |
| WP_062694974.1 |  |  |  | + |  |  |  | 2.A.6.2.28 | -12 | A0Q8A4 | Unknown | Unknown |
| ABI39_00280 | + |  |  |  |  |  |  | 2.A.6.2.32 | -09 | Q9HWH5 | Unknown | Unknown |
| [WP_029428105.1](file:///Volumes/ESD-USB/Summer%202018/Hassan%20Submission%20PLosOne/content.html#lcl|NZ_CP012801.1_prot_WP_029428105.1_5169) |  |  | + |  |  |  |  | [2.A.6.2.32](http://tcdb.org/search/result.php?tc=2.A.6.2.32) | -06 | Q9HWH5 | Drugs | Fluoroquinolones |
| [WP_032946435.1](file:///Volumes/ESD-USB/Summer%202018/Library/Application%20Support/Microsoft/Office/Office%202011%20AutoRecovery/content.html#lcl|NC_009614.1_prot_WP_032946435.1_63) |  |  |  |  |  |  | + | [2.A.6.2.32](http://tcdb.org/search/result.php?tc=2.A.6.2.32) | -09 | Q9HWH5 | Drugs | Fluoroquinolones |
| ABI39_09425 | + |  |  |  |  |  |  | 2.A.6.2.33 | 0 | Q9HW27 | Drugs | Fluoroquinolones/Aminoglycosides/Macrolides |
| [WP_013617665.1](file:///Volumes/ESD-USB/Summer%202018/Hassan%20Submission%20PLosOne/content.html#lcl|NC_015164.1_prot_WP_013617665.1_1633) |  | + |  |  |  |  |  | [2.A.6.2.33](http://tcdb.org/search/result.php?tc=2.A.6.2.33) | 0 | Q9HW27 | Drugs | Fluoroquinoloes/Tetracylines/Macrolides |
| [WP_029427295.1](file:///Volumes/ESD-USB/Summer%202018/Hassan%20Submission%20PLosOne/content.html#lcl|NZ_CP012801.1_prot_WP_029427295.1_3660) |  |  | + |  |  |  |  | [2.A.6.2.33](http://tcdb.org/search/result.php?tc=2.A.6.2.33) | 0 | Q9HW27 | Drugs | Fluruquinolones/Aminoglycosides |
| WP_008764017.1 |  |  |  | + |  |  |  | 2.A.6.2.33 | 0 | Q9HW27 | Drugs | Fluoroquinolones, Macrolides, Tetracycline |
| YP_097964.1_681 |  |  |  |  | + |  |  | 2.A.6.2.33 | 0 | Q9HW27 | Drugs | Macrolides/Fluoroquinolones |
| ABI39_21235 | + |  |  |  |  |  |  | 2.A.6.2.37 | 0 | A6P7H1 | Drugs | Detergents |
| [WP_029428309.1](file:///Volumes/ESD-USB/Summer%202018/Hassan%20Submission%20PLosOne/content.html#lcl|NZ_CP012801.1_prot_WP_029428309.1_1237) |  |  | + |  |  |  |  | [2.A.6.2.37](http://tcdb.org/search/result.php?tc=2.A.6.2.37) | 0 | A6P7H1 | Drugs | Detergent |
| [WP_029428353.1](file:///Volumes/ESD-USB/Summer%202018/Hassan%20Submission%20PLosOne/content.html#lcl|NZ_CP012801.1_prot_WP_029428353.1_1159) |  |  | + |  |  |  |  | [2.A.6.2.37](http://tcdb.org/search/result.php?tc=2.A.6.2.37) | 0 | A6P7H1 | Drugs | Detergent |
| WP_008764996.1 |  |  |  | + |  |  |  | 2.A.6.2.37 | 0 | A6P7H1 | Drugs | Detergent |
| YP_100639.1 |  |  |  |  | + |  |  | 2.A.6.2.37 | 0 | A6P7H1 | Drugs | Detergent |
| YP_101792.1 |  |  |  |  | + |  |  | 2.A.6.2.37 | 0 | A6P7H1 | Drugs | Detergent |
| YP_100590.1 |  |  |  |  | + |  |  | 2.A.6.2.37 | 0 | A6P7H1 | Drugs | Detergent |
| [WP_012056022.1](file:///Volumes/ESD-USB/Summer%202018/Library/Application%20Support/Microsoft/Office/Office%202011%20AutoRecovery/content.html#lcl|NC_009614.1_prot_WP_012056022.1_4027) |  |  |  |  |  |  | + | [2.A.6.2.37](http://tcdb.org/search/result.php?tc=2.A.6.2.37) | 0 | A6P7H1 | Drugs | Detergents |
| ABI39_21010 | + |  |  |  |  |  |  | 2.A.6.2.38 | 0 | Q9KRG9 | Drugs | Detergents |
| WP_032841184.1 |  |  |  | + |  |  |  | 2.A.6.2.38 | 0 | Q9KRG9 | Drugs | Detergent |
| [WP_005847070.1](file:///Volumes/ESD-USB/Summer%202018/Library/Application%20Support/Microsoft/Office/Office%202011%20AutoRecovery/content.html#lcl|NC_009614.1_prot_WP_005847070.1_3979) |  |  |  |  |  |  | + | [2.A.6.2.38](http://tcdb.org/search/result.php?tc=2.A.6.2.38) | 0 | Q9KRG9 | Drugs | Detergents |
| ABI39_14365 | + |  |  |  |  |  |  | 2.A.6.2.39 | -21 | Q9I0V8 | Drugs | Macrolides/Monobactams/Tetracyclines |
| [WP_065757466.1](file:///Volumes/ESD-USB/Summer%202018/Hassan%20Submission%20PLosOne/content.html#lcl|NC_015164.1_prot_WP_065757466.1_2678) |  | + |  |  |  |  |  | [2.A.6.2.40](http://tcdb.org/search/result.php?tc=2.A.6.2.40) | -09 | Q2FD69 | Unknown | Chloramphenicol / tetracycline |
| WP_008760594.1 |  |  |  | + |  |  |  | 2.A.6.2.40 | -09 | Q2FD69 | Drugs | Chloramphenicol/Tetracycline |
| WP_008761527.1 |  |  |  | + |  |  |  | 2.A.6.2.40 | -08 | Q2FD71 | Drugs | Chloramphenicol/Tetracycline |
| ABI39_18720 | + |  |  |  |  |  |  | 2.A.6.2.42 | -56 | Q9F241 | Drugs | Fluoroquinolones/Ethidium bromide |
| WP_008766159.1 |  |  |  | + |  |  |  | 2.A.6.2.42 | -56 | Q9F241 | Drugs | Chloramphenicol/Tetracycline |
| [WP_004297379.1](file:///Volumes/ESD-USB/Summer%202018/Hassan%20Submission%20PLosOne/content.html#lcl|NZ_CP012938.1_prot_WP_004297379.1_144) |  |  |  |  |  | + |  | [2.A.6.2.42](http://tcdb.org/search/result.php?tc=2.A.6.2.42) | -56 | Q9F241 | Drugs | Ethidium bromide/Norfloxacin |
| WP_061474266.1 |  |  |  | + |  |  |  | 2.A.6.2.44 | -52 | Q2FD82 | Drugs | Chloramphenicol/Tetracycline |
| [WP_005852982.1](file:///Volumes/ESD-USB/Summer%202018/Library/Application%20Support/Microsoft/Office/Office%202011%20AutoRecovery/content.html#lcl|NC_009614.1_prot_WP_005852982.1_316) |  |  |  |  |  |  | + | [2.A.6.2.44](http://tcdb.org/search/result.php?tc=2.A.6.2.44) | -56 | Q2FD82 | Drugs | Fluoroquinolones, microlides, chloramphenicol, and tetracycline |
| WP_062695209.1 |  |  |  | + |  |  |  | 2.A.6.2.46 | -69 | Q0VQY7 | Drugs | Detergent |
| WP_062695093.1 |  |  |  | + |  |  |  | 2.A.6.2.46 | -65 | Q0VQY7 | Drugs | Detergent |
| YP_100430.1 |  |  |  |  | + |  |  | 2.A.6.3.4 | -76 | Q1DEX6 | Unknown | Unknown |
| ABI39_01255 | + |  |  |  |  |  |  | 2.A.6.3.6 | -176 | Q8CX78 | Cations | Cations |
| ABI39_00275 | + |  |  |  |  |  |  | 2.A.6.3.6 | -101 | Q8CX78 | Cations | Cations |
| ABI39_19360 | + |  |  |  |  |  |  | 2.A.6.3.6 | -80 | Q8CX78 | Cations | Cations |
| ABI39_00265 | + |  |  |  |  |  |  | 2.A.6.3.6 | -47 | Q8CX78 | Cations | Cations |
| [WP_029426684.1](file:///Volumes/ESD-USB/Summer%202018/Hassan%20Submission%20PLosOne/content.html#lcl|NZ_CP012801.1_prot_WP_029426684.1_3295) |  |  | + |  |  |  |  | [2.A.6.3.6](http://tcdb.org/search/result.php?tc=2.A.6.3.6) | -177 | Q8CX78 | Cations | Cations |
| [WP_029427845.1](file:///Volumes/ESD-USB/Summer%202018/Hassan%20Submission%20PLosOne/content.html#lcl|NZ_CP012801.1_prot_WP_029427845.1_1629) |  |  | + |  |  |  |  | [2.A.6.3.6](http://tcdb.org/search/result.php?tc=2.A.6.3.6) | -101 | Q8CX78 | Cations | Cations |
| WP_008762065.1 |  |  |  | + |  |  |  | 2.A.6.3.6 | -179 | Q8CX78 | Unknown | Unknown |
| WP_008766163.1 |  |  |  | + |  |  |  | 2.A.6.3.6 | -145 | Q8CX78 | Unknown | Unknown |
| WP_062694976.1 |  |  |  | + |  |  |  | 2.A.6.3.6 | -103 | Q8CX78 | Unknown | Unknown |
| WP_008760597.1 |  |  |  | + |  |  |  | 2.A.6.3.6 | -101 | Q8CX78 | Unknown | Unknown |
| WP_062694978.1 |  |  |  | + |  |  |  | 2.A.6.3.6 | -48 | Q8CX78 | Unknown | Unknown |
| YP_101416.1 |  |  |  |  | + |  |  | 2.A.6.3.6 | 0 | Q8CX78 | Unknown | Unknown |
| [WP_004302026.1](file:///Volumes/ESD-USB/Summer%202018/Hassan%20Submission%20PLosOne/content.html#lcl|NZ_CP012938.1_prot_WP_004302026.1_4571) |  |  |  |  |  | + |  | [2.A.6.3.6](http://tcdb.org/search/result.php?tc=2.A.6.3.6) | -113 | Q8CX78 | Drugs | Acriflavine |
| [WP_004302025.1](file:///Volumes/ESD-USB/Summer%202018/Hassan%20Submission%20PLosOne/content.html#lcl|NZ_CP012938.1_prot_WP_004302025.1_4570) |  |  |  |  |  | + |  | [2.A.6.3.6](http://tcdb.org/search/result.php?tc=2.A.6.3.6) | -41 | Q8CX78 | Drugs | Acriflavine |
| [WP_005845553.1](file:///Volumes/ESD-USB/Summer%202018/Library/Application%20Support/Microsoft/Office/Office%202011%20AutoRecovery/content.html#lcl|NC_009614.1_prot_WP_005845553.1_232) |  |  |  |  |  |  | + | [2.A.6.3.6](http://tcdb.org/search/result.php?tc=2.A.6.3.6) | -177 | Q8CX78 | Drugs | Acriflavine |
| [WP_011964605.1](file:///Volumes/ESD-USB/Summer%202018/Library/Application%20Support/Microsoft/Office/Office%202011%20AutoRecovery/content.html#lcl|NC_009614.1_prot_WP_011964605.1_62) |  |  |  |  |  |  | + | [2.A.6.3.6](http://tcdb.org/search/result.php?tc=2.A.6.3.6) | -98 | Q8CX78 | Drugs | Acriflavine |
| [WP_005851161.1](file:///Volumes/ESD-USB/Summer%202018/Library/Application%20Support/Microsoft/Office/Office%202011%20AutoRecovery/content.html#lcl|NC_009614.1_prot_WP_005851161.1_60) |  |  |  |  |  |  | + | [2.A.6.3.6](http://tcdb.org/search/result.php?tc=2.A.6.3.6) | -47 | Q8CX78 | Drugs | Acriflavine |
| YP_100428.1 |  |  |  |  | + |  |  | 2.A.6.3.11 | -46 | 763427793 | Unknown | Unknown |
| ABI39_06645 | + |  |  |  |  |  |  | 2.A.6.4.3 | -93 | Q5SKE6 | Unknown | Unknown |
| [WP_013618465.1](file:///Volumes/ESD-USB/Summer%202018/Hassan%20Submission%20PLosOne/content.html#lcl|NC_015164.1_prot_WP_013618465.1_2461) |  | + |  |  |  |  |  | [2.A.6.4.3](http://tcdb.org/search/result.php?tc=2.A.6.4.3) | -91 | Q5SKE6 | Unknown | Unknown |
| 4WP_062694255.1 |  |  |  | + |  |  |  | 2.A.6.4.3 | -98 | Q5SKE6 | Unknown | Unknown |
| [WP_004296487.1](file:///Volumes/ESD-USB/Summer%202018/Hassan%20Submission%20PLosOne/content.html#lcl|NZ_CP012938.1_prot_WP_004296487.1_2774) |  |  |  |  |  | + |  | [2.A.6.4.3](http://tcdb.org/search/result.php?tc=2.A.6.4.3) | -96 | Q5SKE6 | Unknown | Unknown |
| [WP_005844398.1](file:///Volumes/ESD-USB/Summer%202018/Library/Application%20Support/Microsoft/Office/Office%202011%20AutoRecovery/content.html#lcl|NC_009614.1_prot_WP_005844398.1_974) |  |  |  |  |  |  | + | [2.A.6.4.3](http://tcdb.org/search/result.php?tc=2.A.6.4.3) | -93 | Q5SKE6 | Unknown | Unknown |
| WP_055173478.1 |  |  |  | + |  |  |  | 2.A.6.7.7 | -164 | F4LLK3 | Lipids | Lipids |
| WP_062695657.1 |  |  |  | + |  |  |  | 2.A.6.7.7 | -148 | F4LLK3 | Lipids | Lipids |
| [WP_004297280.1](file:///Volumes/ESD-USB/Summer%202018/Hassan%20Submission%20PLosOne/content.html#lcl|NZ_CP012938.1_prot_WP_004297280.1_3896) |  |  |  |  |  | + |  | [2.A.6.8.1](http://tcdb.org/search/result.php?tc=2.A.6.8.1) | -17 | Q9EY29 | Unknown | Unknown |
| [WP_013619157.1](file:///Volumes/ESD-USB/Summer%202018/Hassan%20Submission%20PLosOne/content.html#lcl|NC_015164.1_prot_WP_013619157.1_3210) |  | + |  |  |  |  |  | [2.A.7.1.4](http://tcdb.org/search/result.php?tc=2.A.7.1.4) | -19 | P69937 | Quaternary ammonium compounds | Cetylpyridinium, cetyldimethyl ethylammonium, hexadecyltrimethyl ammonium |
| [WP_007213213.1](file:///Volumes/ESD-USB/Summer%202018/Hassan%20Submission%20PLosOne/content.html#lcl|NZ_CP012801.1_prot_WP_007213213.1_4451) |  |  | + |  |  |  |  | [2.A.7.1.4](http://tcdb.org/search/result.php?tc=2.A.7.1.4) | -18 | P69937 | Quaternary ammonium compounds | Cetylpyridinium, cetyldimethyl ethylammonium, hexadecyltrimethyl ammonium |
| WP_008764901.1 |  |  |  | + |  |  |  | 2.A.7.1.5 | -23 | P49857 | Drugs | Ethidium bromide, proflavin, tetraphenylarsonium chloride, crystal violet, pyronin, methylviologen, cetylperdinium chloride, streptomycin, tetracycline, chloramphenicol, phosphonomycin |
| [WP_004299159.1](file:///Volumes/ESD-USB/Summer%202018/Hassan%20Submission%20PLosOne/content.html#lcl|NZ_CP012938.1_prot_WP_004299159.1_2250) |  |  |  |  |  | + |  | [2.A.7.1.10](http://tcdb.org/search/result.php?tc=2.A.7.1.10) | -19 | D5CES3 | Unknown | Unknown |
| ABI39_19355 | + |  |  |  |  |  |  | 2.A.7.2.1 | -32 | P29939 | Unknown | Unknown |
| WP_011107871.1 |  |  |  | + |  |  |  | 2.A.7.2.4 | -17 | K9FTF6 | Drugs | Ethidium bromide, proflavin, tetraphenylarsonium chloride, crystal violet, pyronin, methylviologen, cetylperdinium chloride, streptomycin, tetracycline, chloramphenicol, phosphonomycin |
| ABI39_19240 | + |  |  |  |  |  |  | 2.A.7.3.22 | -27 | p0aa70 | Amino Acids | Amino Acids |
| WP_008764892.1 |  |  |  | + |  |  |  | 2.A.7.3.22 | E-23 | p0aa70 | Unknown | Unknown |
| ABI39_09980 | + |  |  |  |  |  |  | 2.A.7.3.28 | -09 | O29973 | Unknown | Unknown |
| [WP_007215250.1](file:///Volumes/ESD-USB/Summer%202018/Hassan%20Submission%20PLosOne/content.html#lcl|NZ_CP012801.1_prot_WP_007215250.1_3819) |  |  | + |  |  |  |  | [2.A.7.3.28](http://tcdb.org/search/result.php?tc=2.A.7.3.28) | -12 | O29973 | Unknown | Unknown |
| [WP_007211083.1](file:///Volumes/ESD-USB/Summer%202018/Hassan%20Submission%20PLosOne/content.html#lcl|NZ_CP012801.1_prot_WP_007211083.1_2460) |  |  | + |  |  |  |  | [2.A.7.3.28](http://tcdb.org/search/result.php?tc=2.A.7.3.28) | -08 | O29973 | Unknown | Unknown |
| [WP_022209352.1](file:///Volumes/ESD-USB/Summer%202018/Hassan%20Submission%20PLosOne/content.html#lcl|NZ_CP012801.1_prot_WP_022209352.1_3446) |  |  | + |  |  |  |  | [2.A.7.3.28](http://tcdb.org/search/result.php?tc=2.A.7.3.28) | -06 | O29973 | Unknown | Unknown |
| YP_098075.1_792 |  |  |  |  | + |  |  | 2.A.7.3.28 | -13 | O29973 | Unknown | Unknown |
| WP_008764292.1 |  |  |  | + |  |  |  | 2.A.7.3.28 | -11 | O29973 | Unknown | Unknown |
| [WP_004301430.1](file:///Volumes/ESD-USB/Summer%202018/Hassan%20Submission%20PLosOne/content.html#lcl|NZ_CP012938.1_prot_WP_004301430.1_4130) |  |  |  |  |  | + |  | [2.A.7.3.28](http://tcdb.org/search/result.php?tc=2.A.7.3.28) | -12 | O29973 | Unknown | Unknown |
| [WP_012055930.1](file:///Volumes/ESD-USB/Summer%202018/Library/Application%20Support/Microsoft/Office/Office%202011%20AutoRecovery/content.html#lcl|NC_009614.1_prot_WP_012055930.1_3796) |  |  |  |  |  |  | + | [2.A.7.3.28](http://tcdb.org/search/result.php?tc=2.A.7.3.28) | -09 | O29973 | Unknown | Unknown |
| [WP_011965397.1](file:///Volumes/ESD-USB/Summer%202018/Library/Application%20Support/Microsoft/Office/Office%202011%20AutoRecovery/content.html#lcl|NC_009614.1_prot_WP_011965397.1_1941) |  |  |  |  |  |  | + | [2.A.7.3.28](http://tcdb.org/search/result.php?tc=2.A.7.3.28) | -09 | O29973 | Unknown | Unknown |
| WP_008760419.1 |  |  |  | + |  |  |  | 2.A.7.3.29 | -07 | O34416 | Unknown | Unknown |
| [WP_029428281.1](file:///Volumes/ESD-USB/Summer%202018/Hassan%20Submission%20PLosOne/content.html#lcl|NZ_CP012801.1_prot_WP_029428281.1_1307) |  |  | + |  |  |  |  | [2.A.7.3.31](http://tcdb.org/search/result.php?tc=2.A.7.3.31) | -05 | P96661 | Unknown | Unknown |
| [WP_005853396.1](file:///Volumes/ESD-USB/Summer%202018/Library/Application%20Support/Microsoft/Office/Office%202011%20AutoRecovery/content.html#lcl|NC_009614.1_prot_WP_005853396.1_3109) |  |  |  |  |  |  | + | [2.A.7.3.31](http://tcdb.org/search/result.php?tc=2.A.7.3.31) | -05 | P96661 | Unknown | Unknown |
| ABI39_19335 | + |  |  |  |  |  |  | 2.A.7.3.32 | -09 | P96680 | Unknown | Unknown |
| [WP_013618822.1](file:///Volumes/ESD-USB/Summer%202018/Hassan%20Submission%20PLosOne/content.html#lcl|NC_015164.1_prot_WP_013618822.1_2862) |  | + |  |  |  |  |  | [2.A.7.3.35](http://tcdb.org/search/result.php?tc=2.A.7.3.35) | -10 | O31540 | Unknown | Unknown |
| ABI39_17075 | + |  |  |  |  |  |  | 2.A.7.3.36 | -22 | O29740 | Unknown | Unknown |
| ABI39_20120 | + |  |  |  |  |  |  | 2.A.7.3.36 | -08 | O29740 | Unknown | Unknown |
| [WP_013617484.1](file:///Volumes/ESD-USB/Summer%202018/Hassan%20Submission%20PLosOne/content.html#lcl|NC_015164.1_prot_WP_013617484.1_1450) |  | + |  |  |  |  |  | [2.A.7.3.36](http://tcdb.org/search/result.php?tc=2.A.7.3.36) | -21 | O29740 | Unknown | Unknown |
| [WP_029426331.1](file:///Volumes/ESD-USB/Summer%202018/Hassan%20Submission%20PLosOne/content.html#lcl|NZ_CP012801.1_prot_WP_029426331.1_2653) |  |  | + |  |  |  |  | [2.A.7.3.36](http://tcdb.org/search/result.php?tc=2.A.7.3.36) | -20 | O29740 | Unknown | Unknown |
| WP_008767386.1 |  |  |  | + |  |  |  | 2.A.7.3.36 | -22 | O29740 | Unknown | Unknown |
| WP_008765200.1 |  |  |  | + |  |  |  | 2.A.7.3.36 | -09 | O29740 | Unknown | Unknown |
| YP_099181.1_1898 |  |  |  |  | + |  |  | 2.A.7.3.36 | -28 | O29740 | Unknown | Unknown |
| YP_097895.1_612 |  |  |  |  | + |  |  | 2.A.7.3.36 | -09 | O29740 | Unknown | Unknown |
| [WP_004298693.1](file:///Volumes/ESD-USB/Summer%202018/Hassan%20Submission%20PLosOne/content.html#lcl|NZ_CP012938.1_prot_WP_004298693.1_3064) |  |  |  |  |  | + |  | [2.A.7.3.36](http://tcdb.org/search/result.php?tc=2.A.7.3.36) | -20 | O29740 | Unknown | Unknown |
| [WP_004296266.1](file:///Volumes/ESD-USB/Summer%202018/Hassan%20Submission%20PLosOne/content.html#lcl|NZ_CP012938.1_prot_WP_004296266.1_2580) |  |  |  |  |  | + |  | [2.A.7.3.36](http://tcdb.org/search/result.php?tc=2.A.7.3.36) | -09 | O29740 | Unknown | Unknown |
| [WP_012055621.1](file:///Volumes/ESD-USB/Summer%202018/Library/Application%20Support/Microsoft/Office/Office%202011%20AutoRecovery/content.html#lcl|NC_009614.1_prot_WP_012055621.1_3207) |  |  |  |  |  |  | + | [2.A.7.3.36](http://tcdb.org/search/result.php?tc=2.A.7.3.36) | -22 | O29740 | Unknown | Unknown |
| ABI39_18820 | + |  |  |  |  |  |  | 2.A.7.3.57 | -174 | Q89ZY3 | Unknown | Unknown |
| [WP_013617417.1](file:///Volumes/ESD-USB/Summer%202018/Hassan%20Submission%20PLosOne/content.html#lcl|NC_015164.1_prot_WP_013617417.1_1384) |  | + |  |  |  |  |  | [2.A.7.3.57](http://tcdb.org/search/result.php?tc=2.A.7.3.57) | -169 | Q89ZY3 | Unknown | Unknown |
| [WP_022209235.1](file:///Volumes/ESD-USB/Summer%202018/Hassan%20Submission%20PLosOne/content.html#lcl|NZ_CP012801.1_prot_WP_022209235.1_3984) |  |  | + |  |  |  |  | [2.A.7.3.57](http://tcdb.org/search/result.php?tc=2.A.7.3.57) | 0 | Q89ZY3 | Unknown | Unknown |
| WP_011109171.1 |  |  |  | + |  |  |  | 2.A.7.3.57 | 0 | Q89ZY3 | Unknown | Unknown |
| YP_098211.1_928 |  |  |  |  | + |  |  | 2.A.7.3.57 | 0 | Q89ZY3 | Unknown | Unknown |
| [WP_005841579.1](file:///Volumes/ESD-USB/Summer%202018/Library/Application%20Support/Microsoft/Office/Office%202011%20AutoRecovery/content.html#lcl|NC_009614.1_prot_WP_005841579.1_3715) |  |  |  |  |  |  | + | [2.A.7.3.57](http://tcdb.org/search/result.php?tc=2.A.7.3.57) | -173 | Q89ZY3 | Drugs | Drugs |
| [WP_013616294.1](file:///Volumes/ESD-USB/Summer%202018/Hassan%20Submission%20PLosOne/content.html#lcl|NC_015164.1_prot_WP_013616294.1_217) |  | + |  |  |  |  |  | [2.A.7.3.65](http://tcdb.org/search/result.php?tc=2.A.7.3.65) | -08 | M4V8N5 | Unknown | Unknown |
| [WP_029427425.1](file:///Volumes/ESD-USB/Summer%202018/Hassan%20Submission%20PLosOne/content.html#lcl|NZ_CP012801.1_prot_WP_029427425.1_3508) |  |  | + |  |  |  |  | [2.A.7.3.65](http://tcdb.org/search/result.php?tc=2.A.7.3.65) | -12 | M4V8N5 | Unknown | Unknown |
| WP_048693993.1 |  |  |  | + |  |  |  | 2.A.7.3.65 | -12 | M4V8N5 | Unknown | Unknown |
| WP_016269238.1 |  |  |  | + |  |  |  | 2.A.7.3.65 | -09 | M4V8N5 | Unknown | Unknown |
| YP_097450.1 |  |  |  |  | + |  |  | 2.A.7.3.65 | -12 | M4V8N5 | Unknown | Unknown |
| YP_100366.1 |  |  |  |  | + |  |  | 2.A.7.3.65 | -10 | M4V8N5 | Unknown | Unknown |
| YP_098571.1 |  |  |  |  | + |  |  | 2.A.7.3.65 | -07 | M4V8N5 | Unknown | Unknown |
| [WP_004299199.1](file:///Volumes/ESD-USB/Summer%202018/Hassan%20Submission%20PLosOne/content.html#lcl|NZ_CP012938.1_prot_WP_004299199.1_2215) |  |  |  |  |  | + |  | [2.A.7.3.65](http://tcdb.org/search/result.php?tc=2.A.7.3.65) | -12 | M4V8N5 | Unknown | Unknown |
| [WP_004298047.1](file:///Volumes/ESD-USB/Summer%202018/Hassan%20Submission%20PLosOne/content.html#lcl|NZ_CP012938.1_prot_WP_004298047.1_3227) |  |  |  |  |  | + |  | [2.A.7.3.65](http://tcdb.org/search/result.php?tc=2.A.7.3.65) | -09 | M4V8N5 | Unknown | Unknown |
| [WP_005843539.1](file:///Volumes/ESD-USB/Summer%202018/Library/Application%20Support/Microsoft/Office/Office%202011%20AutoRecovery/content.html#lcl|NC_009614.1_prot_WP_005843539.1_3179) |  |  |  |  |  |  | + | [2.A.7.3.65](http://tcdb.org/search/result.php?tc=2.A.7.3.65) | -14 | M4V8N5 | unknown | unknown |
| ABI39_03005 | + |  |  |  |  |  |  | 2.A.7.6.2 | -76 | M5UGY9 | Sugars | Rhamnose |
| [WP_013618543.1](file:///Volumes/ESD-USB/Summer%202018/Hassan%20Submission%20PLosOne/content.html#lcl|NC_015164.1_prot_WP_013618543.1_2588) |  | + |  |  |  |  |  | [2.A.7.6.2](http://tcdb.org/search/result.php?tc=2.A.7.6.2) | -61 | M5UGY9 | Sugars | Rhamnose |
| [WP_025725753.1](file:///Volumes/ESD-USB/Summer%202018/Hassan%20Submission%20PLosOne/content.html#lcl|NZ_CP012801.1_prot_WP_025725753.1_1975) |  |  | + |  |  |  |  | [2.A.7.6.2](http://tcdb.org/search/result.php?tc=2.A.7.6.2) | -78 | M5UGY9 | Sugars | Rhamnose |
| [WP_004299877.1](file:///Volumes/ESD-USB/Summer%202018/Hassan%20Submission%20PLosOne/content.html#lcl|NZ_CP012938.1_prot_WP_004299877.1_3724) |  |  |  |  |  | + |  | [2.A.7.6.2](http://tcdb.org/search/result.php?tc=2.A.7.6.2) | -78 | M5UGY9 | Unknown | Unknown |
| WP_008762702.1 |  |  |  | + |  |  |  | 2.A.7.6.2 | -76 | M5UGY9 | Sugars | Rhamnose |
| [WP_005850740.1](file:///Volumes/ESD-USB/Summer%202018/Library/Application%20Support/Microsoft/Office/Office%202011%20AutoRecovery/content.html#lcl|NC_009614.1_prot_WP_005850740.1_588) |  |  |  |  |  |  | + | [2.A.7.6.2](http://tcdb.org/search/result.php?tc=2.A.7.6.2) | -75 | M5UGY9 | Sugars | Rhamnose |
| [WP_013617561.1](file:///Volumes/ESD-USB/Summer%202018/Hassan%20Submission%20PLosOne/content.html#lcl|NC_015164.1_prot_WP_013617561.1_1528) |  | + |  |  |  |  |  | [2.A.7.30.1](http://tcdb.org/search/result.php?tc=2.A.7.30.1) | -43 | Q7URM2 | Unknown | Unknown |
| ABI39_18305 | + |  |  |  |  |  |  | 2.A.7.34.3 | -50 | Q64X46 | Unknown | Unknown |
| [WP_013616592.1](file:///Volumes/ESD-USB/Summer%202018/Hassan%20Submission%20PLosOne/content.html#lcl|NC_015164.1_prot_WP_013616592.1_528) |  | + |  |  |  |  |  | [2.A.7.34.3](http://tcdb.org/search/result.php?tc=2.A.7.34.3) | -51 | Q64X46 | Unknown | Unknown |
| [WP_007215884.1](file:///Volumes/ESD-USB/Summer%202018/Hassan%20Submission%20PLosOne/content.html#lcl|NZ_CP012801.1_prot_WP_007215884.1_4412) |  |  | + |  |  |  |  | [2.A.7.34.3](http://tcdb.org/search/result.php?tc=2.A.7.34.3) | -71 | Q64X46 | Unknown | Unknown |
| WP_062695320.1 |  |  |  | + |  |  |  | 2.A.7.34.3 | -65 | Q64X46 | Unknown | Unknown |
| WP_005821429.1 |  |  |  | + |  |  |  | 2.A.7.34.3 | -63 | Q64X46 | Unknown | Unknown |
| WP_062695315.1 |  |  |  | + |  |  |  | 2.A.7.34.3 | -43 | Q64X46 | Unknown | Unknown |
| YP_098464.1_1181 |  |  |  |  | + |  |  | 2.A.7.34.3 | -87 | Q64X46 | Unknown | Unknown |
| [WP_004302024.1](file:///Volumes/ESD-USB/Summer%202018/Hassan%20Submission%20PLosOne/content.html#lcl|NZ_CP012938.1_prot_WP_004302024.1_4569) |  |  |  |  |  | + |  | [2.A.7.34.3](http://tcdb.org/search/result.php?tc=2.A.7.34.3) | -65 | Q64X46 | Unknown | Unknown |
| [WP_005851774.1](file:///Volumes/ESD-USB/Summer%202018/Library/Application%20Support/Microsoft/Office/Office%202011%20AutoRecovery/content.html#lcl|NC_009614.1_prot_WP_005851774.1_3556) |  |  |  |  |  |  | + | [2.A.7.34.3](http://tcdb.org/search/result.php?tc=2.A.7.34.3) | 50 | Q64X46 | Unknown | Unknown |
| [WP_004296444.1](file:///Volumes/ESD-USB/Summer%202018/Hassan%20Submission%20PLosOne/content.html#lcl|NZ_CP012938.1_prot_WP_004296444.1_2735) |  |  |  |  |  | + |  | [2.A.8.1.9](http://tcdb.org/search/result.php?tc=2.A.8.1.9) | -142 | Q57493 | Unknown | Unknown |
| WP_008763174.1 |  |  |  | + |  |  |  | 2.A.9.3.1 | -33 | P25714 | Proteins | Proteins |
| ABI39_14305 | + |  |  |  |  |  |  | 2.A.13.1.3 | -134 | Q8ZLD2 | Carboxylates | Succinate/Fumarate |
| WP_008762009.1 |  |  |  | + |  |  |  | 2.A.13.1.3 | -136 | Q8ZLD2 | Carboxylates | Succinate/Fumarate/Aspartate/ |
| YP_101491.1_4208 |  |  |  |  | + |  |  | 2.A.13.1.3 | -136 | Q8ZLD2 | Carboxylates | Succinate, fumarate, aspartate |
| [WP_004323663.1](file:///Volumes/ESD-USB/Summer%202018/Hassan%20Submission%20PLosOne/content.html#lcl|NZ_CP012938.1_prot_WP_004323663.1_2686) |  |  |  |  |  | + |  | [2.A.13.1.3](http://tcdb.org/search/result.php?tc=2.A.13.1.3) | -137 | Q8ZLD2 | Carboxylates | Succinate/Fumarate/Aspartate |
| [WP_011965736.1](file:///Volumes/ESD-USB/Summer%202018/Library/Application%20Support/Microsoft/Office/Office%202011%20AutoRecovery/content.html#lcl|NC_009614.1_prot_WP_011965736.1_2740) |  |  |  |  |  |  | + | [2.A.13.1.3](http://tcdb.org/search/result.php?tc=2.A.13.1.3) | -133 | Q8ZLD2 | Carboxylates | Succinate/Fumarate/Aspartate |
| WP_016267344.1 |  |  |  | + |  |  |  | 2.A.17.1.2 | -34 | P77304 | Peptides | Peptides |
| WP_011107468.1 |  |  |  | + |  |  |  | 2.A.17.1.4 | -09 | P75742 | Peptides | Peptides |
| [WP_013617431.1](file:///Volumes/ESD-USB/Summer%202018/Hassan%20Submission%20PLosOne/content.html#lcl|NC_015164.1_prot_WP_013617431.1_1397) |  | + |  |  |  |  |  | [2.A.17.1.6](http://tcdb.org/search/result.php?tc=2.A.17.1.6) | -46 | Q5M4H8 | Unknown | Unknown |
| [WP_004301900.1](file:///Volumes/ESD-USB/Summer%202018/Hassan%20Submission%20PLosOne/content.html#lcl|NZ_CP012938.1_prot_WP_004301900.1_4460) |  |  |  |  |  | + |  | [2.A.17.1.6](http://tcdb.org/search/result.php?tc=2.A.17.1.6) | -70 | Q5M4H8 | Unknown | Unknown |
| [WP_029427118.1](file:///Volumes/ESD-USB/Summer%202018/Hassan%20Submission%20PLosOne/content.html#lcl|NZ_CP012801.1_prot_WP_029427118.1_3966) |  |  | + |  |  |  |  | [2.A.17.1.7](http://tcdb.org/search/result.php?tc=2.A.17.1.7) | -68 | Q5KYD1 | Peptides | Peptides |
| WP_008764544.1 |  |  |  | + |  |  |  | 2.A.17.1.7 | -69 | Q5KYD1 | Peptides | Peptides |
| YP_101814.1 |  |  |  |  | + |  |  | 2.A.19.5.2 | -53 | Q0ZAI3 | Cations | Ca^2+^/Na^2+^ |
| WP_008767297.1 |  |  |  | + |  |  |  | 2.A.19.5.2 | -52 | Q0ZAI3 | Cations | Ca^2+^/Na^+^ |
| [WP_004298386.1](file:///Volumes/ESD-USB/Summer%202018/Hassan%20Submission%20PLosOne/content.html#lcl|NZ_CP012938.1_prot_WP_004298386.1_2891) |  |  |  |  |  | + |  | [2.A.19.5.2](http://tcdb.org/search/result.php?tc=2.A.19.5.2) | -55 | Q0ZAI3 | Unknown | Unknown |
| [WP_005849851.1](file:///Volumes/ESD-USB/Summer%202018/Library/Application%20Support/Microsoft/Office/Office%202011%20AutoRecovery/content.html#lcl|NC_009614.1_prot_WP_005849851.1_1215) |  |  |  |  |  |  | + | [2.A.19.5.2](http://tcdb.org/search/result.php?tc=2.A.19.5.2) | -46 | Q0ZAI3 | Unknown | Unknown |
| [WP_007217446.1](file:///Volumes/ESD-USB/Summer%202018/Hassan%20Submission%20PLosOne/content.html#lcl|NZ_CP012801.1_prot_WP_007217446.1_1190) |  |  | + |  |  |  |  | [2.A.20.1.4](http://tcdb.org/search/result.php?tc=2.A.20.1.4) | -86 | O30499 | Cations | PO_4_^3-^ |
| WP_008766890.1 |  |  |  | + |  |  |  | 2.A.20.1.4 | -89 | O30499 | Anions | PO_4_^3-^ |
| [WP_004302159.1](file:///Volumes/ESD-USB/Summer%202018/Hassan%20Submission%20PLosOne/content.html#lcl|NZ_CP012938.1_prot_WP_004302159.1_4658) |  |  |  |  |  | + |  | [2.A.20.1.4](http://tcdb.org/search/result.php?tc=2.A.20.1.4) | -90 | O30499 | Cations | PO_4_^3-^ |
| [WP_004296811.1](file:///Volumes/ESD-USB/Summer%202018/Hassan%20Submission%20PLosOne/content.html#lcl|NZ_CP012938.1_prot_WP_004296811.1_1865) |  |  |  |  |  | + |  | [2.A.20.2.9](http://tcdb.org/search/result.php?tc=2.A.20.2.9) | -05 | O26024 | Cations | PO_4_^3-^ |
| WP_008760499.1 |  |  |  | + |  |  |  | 2.A.21.2.4 | -37 | J7JIE8 | Amino Acids | Proline |
| YP_100476.1 |  |  |  |  | + |  |  | 2.A.21.2.4 | -35 | J7JIE8 | Unknown | Unknown |
| [WP_004296930.1](file:///Volumes/ESD-USB/Summer%202018/Hassan%20Submission%20PLosOne/content.html#lcl|NZ_CP012938.1_prot_WP_004296930.1_72) |  |  |  |  |  | + |  | [2.A.21.2.4](http://tcdb.org/search/result.php?tc=2.A.21.2.4) | -36 | J7JIE8 | Unknown | Unknown |
| ABI39_14295 | + |  |  |  |  |  |  | 2.A.21.3.7 | -76 | Q5E733 | Sugars | Glucose/Galactose |
| WP_011108495.1 |  |  |  | + |  |  |  | 2.A.21.3.7 | -75 | Q5E733 | Sugars | Sialic acid |
| WP_008767085.1 |  |  |  | + |  |  |  | 2.A.21.3.7 | -28 | Q5E733 | Sugars | Sialic acid |
| YP_097720.1_437 |  |  |  |  | + |  |  | 2.A.21.3.7 | -25 | Q5E733 | Sugars | Sialic acid |
| [WP_004299696.1](file:///Volumes/ESD-USB/Summer%202018/Hassan%20Submission%20PLosOne/content.html#lcl|NZ_CP012938.1_prot_WP_004299696.1_3591) |  |  |  |  |  | + |  | [2.A.21.3.7](http://tcdb.org/search/result.php?tc=2.A.21.3.7) | -27 | Q5E733 | Unknown | Unknown |
| [WP_004318555.1](file:///Volumes/ESD-USB/Summer%202018/Hassan%20Submission%20PLosOne/content.html#lcl|NZ_CP012938.1_prot_WP_004318555.1_519) |  |  |  |  |  | + |  | [2.A.21.3.7](http://tcdb.org/search/result.php?tc=2.A.21.3.7) | -21 | Q5E733 | Unknown | Unknown |
| [WP_005843381.1](file:///Volumes/ESD-USB/Summer%202018/Library/Application%20Support/Microsoft/Office/Office%202011%20AutoRecovery/content.html#lcl|NC_009614.1_prot_WP_005843381.1_2738) |  |  |  |  |  |  | + | [2.A.21.3.7](http://tcdb.org/search/result.php?tc=2.A.21.3.7) | -77 | Q5E733 | Sugars | Glucose/Galactose |
| WP_062694156.1 |  |  |  | + |  |  |  | 2.A.21.3.8 | 0 | A1S2A8 | Sugars | Mannose |
| [WP_011964813.1](file:///Volumes/ESD-USB/Summer%202018/Library/Application%20Support/Microsoft/Office/Office%202011%20AutoRecovery/content.html#lcl|NC_009614.1_prot_WP_011964813.1_571) |  |  |  |  |  |  | + | [2.A.21.3.8](http://tcdb.org/search/result.php?tc=2.A.21.3.8) | 0 | A1S2A8 | Unknown | Unknown |
| [WP_029426598.1](file:///Volumes/ESD-USB/Summer%202018/Hassan%20Submission%20PLosOne/content.html#lcl|NZ_CP012801.1_prot_WP_029426598.1_3114) |  |  | + |  |  |  |  | [2.A.21.3.11](http://tcdb.org/search/result.php?tc=2.A.21.3.11) | -37 | A3UWQ1 | Sugars | Oligosaccharides |
| [WP_029426470.1](file:///Volumes/ESD-USB/Summer%202018/Hassan%20Submission%20PLosOne/content.html#lcl|NZ_CP012801.1_prot_WP_029426470.1_2890) |  |  | + |  |  |  |  | [2.A.21.3.18](http://tcdb.org/search/result.php?tc=2.A.21.3.18) | -84 | Q9NY91 | Sugars | Oligosaccharides |
| WP_062694370.1 |  |  |  | + |  |  |  | 2.A.21.3.18 | -90 | Q9NY91 | Sugars | Glucose |
| ABI39_11630 | + |  |  |  |  |  |  | 2.A.21.3.19 | -146 | Q8AAV7 | Sugars | Arabinose |
| ABI39_14675 | + |  |  |  |  |  |  | 2.A.21.3.19 | -80 | Q8AAV7 | Sugars | Glucose |
| [WP_013616768.1](file:///Volumes/ESD-USB/Summer%202018/Hassan%20Submission%20PLosOne/content.html#lcl|NC_015164.1_prot_WP_013616768.1_710) |  | + |  |  |  |  |  | [2.A.21.3.19](http://tcdb.org/search/result.php?tc=2.A.21.3.19) | -158 | Q8AAV7 | Sugars | Arabinose |
| [WP_029428502.1](file:///Volumes/ESD-USB/Summer%202018/Hassan%20Submission%20PLosOne/content.html#lcl|NZ_CP012801.1_prot_WP_029428502.1_875) |  |  | + |  |  |  |  | [2.A.21.3.19](http://tcdb.org/search/result.php?tc=2.A.21.3.19) | 0 | Q8AAV7 | Sugars | Arabinose |
| WP_008766122.1 |  |  |  | + |  |  |  | 2.A.21.3.19 | 0 | Q8AAV7 | Sugars | Arabinose |
| WP_008766132.1 |  |  |  | + |  |  |  | 2.A.21.3.19 | -77 | Q8AAV7 | Sugars | Arabinose |
| [WP_004297444.1](file:///Volumes/ESD-USB/Summer%202018/Hassan%20Submission%20PLosOne/content.html#lcl|NZ_CP012938.1_prot_WP_004297444.1_194) |  |  |  |  |  | + |  | [2.A.21.3.19](http://tcdb.org/search/result.php?tc=2.A.21.3.19) | -79 | Q8AAV7 | Sugars | Arabinose |
| [WP_004297444.1](file:///Volumes/ESD-USB/Summer%202018/Hassan%20Submission%20PLosOne/content.html#lcl|NZ_CP012938.1_prot_WP_004297444.1_194) |  |  |  |  |  | + |  | [2.A.21.3.19](http://tcdb.org/search/result.php?tc=2.A.21.3.19) | -79 | Q8AAV7 | Sugars | Arabinose |
| [WP_005846197.1](file:///Volumes/ESD-USB/Summer%202018/Library/Application%20Support/Microsoft/Office/Office%202011%20AutoRecovery/content.html#lcl|NC_009614.1_prot_WP_005846197.1_2274) |  |  |  |  |  |  | + | [2.A.21.3.19](http://tcdb.org/search/result.php?tc=2.A.21.3.19) | -146 | Q8AAV7 | Sugars | Glucose |
| [WP_011965818.1](file:///Volumes/ESD-USB/Summer%202018/Library/Application%20Support/Microsoft/Office/Office%202011%20AutoRecovery/content.html#lcl|NC_009614.1_prot_WP_011965818.1_2876) |  |  |  |  |  |  | + | [2.A.21.3.19](http://tcdb.org/search/result.php?tc=2.A.21.3.19) | -80 | Q8AAV7 | Sugars | Glucose |
| [WP_007210716.1](file:///Volumes/ESD-USB/Summer%202018/Hassan%20Submission%20PLosOne/content.html#lcl|NZ_CP012801.1_prot_WP_007210716.1_2114) |  |  | + |  |  |  |  | [2.A.21.5.1](http://tcdb.org/search/result.php?tc=2.A.21.5.1) | -24 | Q92911 | Cations | Na^+^ |
| ABI39_07830 | + |  |  |  |  |  |  | 2.A.22.5.3 | -117 | Q9KDT3 | Cations | Na^+^ |
| [WP_013618027.1](file:///Volumes/ESD-USB/Summer%202018/Hassan%20Submission%20PLosOne/content.html#lcl|NC_015164.1_prot_WP_013618027.1_2003) |  | + |  |  |  |  |  | [2.A.22.5.3](http://tcdb.org/search/result.php?tc=2.A.22.5.3) | -116 | Q9KDT3 | Unknown | Unknown |
| [WP_022209674.1](file:///Volumes/ESD-USB/Summer%202018/Hassan%20Submission%20PLosOne/content.html#lcl|NZ_CP012801.1_prot_WP_022209674.1_2093) |  |  | + |  |  |  |  | [2.A.22.5.3](http://tcdb.org/search/result.php?tc=2.A.22.5.3) | -124 | Q9KDT3 | Cations | Na^+^ |
| WP_008762587.1 |  |  |  | + |  |  |  | 2.A.22.5.3 | -122 | Q9KDT3 | Unknown | Unknown |
| YP_097732.1 |  |  |  |  | + |  |  | 2.A.22.5.3 | -122 | Q9KDT3 | Cations | Na^+^ |
| [WP_004299713.1](file:///Volumes/ESD-USB/Summer%202018/Hassan%20Submission%20PLosOne/content.html#lcl|NZ_CP012938.1_prot_WP_004299713.1_3607) |  |  |  |  |  | + |  | [2.A.22.5.3](http://tcdb.org/search/result.php?tc=2.A.22.5.3) | -120 | Q9KDT3 | Unknown | Unknown |
| [WP_008668319.1](file:///Volumes/ESD-USB/Summer%202018/Library/Application%20Support/Microsoft/Office/Office%202011%20AutoRecovery/content.html#lcl|NC_009614.1_prot_WP_008668319.1_1473) |  |  |  |  |  |  | + | [2.A.22.5.3](http://tcdb.org/search/result.php?tc=2.A.22.5.3) | -118 | Q9KDT3 | Unknown | Unknown |
| ABI39_14330 | **+** |  |  |  |  |  |  | 2.A.23.2.11 | -19 | B0W0K4 | Amino acids | Glutamate |
| [WP_029328277.1](file:///Volumes/ESD-USB/Summer%202018/Hassan%20Submission%20PLosOne/content.html#lcl|NZ_CP012801.1_prot_WP_029328277.1_639) |  |  | + |  |  |  |  | [2.A.23.2.11](http://tcdb.org/search/result.php?tc=2.A.23.2.11) | -21 | B0W0K4 | Amino acids | Glutamate |
| WP_008763417.1 |  |  |  | + |  |  |  | 2.A.23.2.11 | -21 | B0W0K4 | Amino Acids | Glutamate/Aspartate |
| YP_099134.1 |  |  |  |  | **+** |  |  | 2.A.23.2.11 | -22 | B0W0K4 | Amino acids | Glutamate |
| [WP_004297835.1](file:///Volumes/ESD-USB/Summer%202018/Hassan%20Submission%20PLosOne/content.html#lcl|NZ_CP012938.1_prot_WP_004297835.1_344) |  |  |  |  |  | + |  | [2.A.23.2.11](http://tcdb.org/search/result.php?tc=2.A.23.2.11) | -20 | B0W0K4 | Amino acids | Glutamate |
| [WP_005843390.1](file:///Volumes/ESD-USB/Summer%202018/Library/Application%20Support/Microsoft/Office/Office%202011%20AutoRecovery/content.html#lcl|NC_009614.1_prot_WP_005843390.1_2743) |  |  |  |  |  |  | + | [2.A.23.2.11](http://tcdb.org/search/result.php?tc=2.A.23.2.11) | -19 | B0W0K4 | Amino acids | Glutamate |
| [WP_007213064.1](file:///Volumes/ESD-USB/Summer%202018/Hassan%20Submission%20PLosOne/content.html#lcl|NZ_CP012801.1_prot_WP_007213064.1_4347) |  |  | + |  |  |  |  | [2.A.25.1.5](http://tcdb.org/search/result.php?tc=2.A.25.1.5) | -162 | Q45068 | Amino acids | Alanine |
| WP_055300519.1 |  |  |  | + |  |  |  | 2.A.25.1.5 | -159 | Q45068 | Amino acids | Alanine |
| YP_100853.1 |  |  |  |  | **+** |  |  | 2.A.25.1.5 | -159 | Q45068 | Amino acids | Amino acids |
| [WP_004301963.1](file:///Volumes/ESD-USB/Summer%202018/Hassan%20Submission%20PLosOne/content.html#lcl|NZ_CP012938.1_prot_WP_004301963.1_4515) |  |  |  |  |  | + |  | [2.A.25.1.5](http://tcdb.org/search/result.php?tc=2.A.25.1.5) | -162 | Q45068 | Amino acids | Alanine |
| [WP_005839028.1](file:///Volumes/ESD-USB/Summer%202018/Library/Application%20Support/Microsoft/Office/Office%202011%20AutoRecovery/content.html#lcl|NC_009614.1_prot_WP_005839028.1_2053) |  |  |  |  |  |  | + | [2.A.25.1.5](http://tcdb.org/search/result.php?tc=2.A.25.1.5) | -153 | Q45068 | Amino acids | Amino Acids |
| [WP_005847452.1](file:///Volumes/ESD-USB/Summer%202018/Library/Application%20Support/Microsoft/Office/Office%202011%20AutoRecovery/content.html#lcl|NC_009614.1_prot_WP_005847452.1_2470) |  |  |  |  |  |  | + | [2.A.27.2.1](http://tcdb.org/search/result.php?tc=2.A.27.2.1) | -45 | B1XKD9 | Amino acids | Glutamate |
| [WP_029428290.1](file:///Volumes/ESD-USB/Summer%202018/Hassan%20Submission%20PLosOne/content.html#lcl|NZ_CP012801.1_prot_WP_029428290.1_1294) |  |  | + |  |  |  |  | [2.A.28.2.6](http://tcdb.org/search/result.php?tc=2.A.28.2.6) | -30 | B7GU58 | Drugs | Macrolides |
| WP_008765514.1 |  |  |  | + |  |  |  | 2.A.33.1.1 | -89 | P13738 | Unknown | Unknown |
| [WP_013618234.1](file:///Volumes/ESD-USB/Summer%202018/Hassan%20Submission%20PLosOne/content.html#lcl|NC_015164.1_prot_WP_013618234.1_2215) |  | + |  |  |  |  |  | [2.A.33.1.2](http://tcdb.org/search/result.php?tc=2.A.33.1.2) | -93 | Q56725 | Unknown | Unknown |
| [WP_007218001.1](file:///Volumes/ESD-USB/Summer%202018/Hassan%20Submission%20PLosOne/content.html#lcl|NZ_CP012801.1_prot_WP_007218001.1_490) |  |  | + |  |  |  |  | [2.A.33.1.2](http://tcdb.org/search/result.php?tc=2.A.33.1.2) | -90 | Q56725 | Cations | Na^+^/K^+^/H^+^ |
| [WP_041583809.1](file:///Volumes/ESD-USB/Summer%202018/Hassan%20Submission%20PLosOne/content.html#lcl|NC_015164.1_prot_WP_041583809.1_132) |  | + |  |  |  |  |  | [2.A.35.1.1](http://tcdb.org/search/result.php?tc=2.A.35.1.1) | -78 | P27611 | Unknown | Unknown |
| ABI39_15345 | **+** |  |  |  |  |  |  | 2.A.35.1.5 | -104 | Q8EHX2 | Amino acids | Methionine |
| [WP_013617054.1](file:///Volumes/ESD-USB/Summer%202018/Hassan%20Submission%20PLosOne/content.html#lcl|NC_015164.1_prot_WP_013617054.1_1001) |  | + |  |  |  |  |  | [2.A.35.1.5](http://tcdb.org/search/result.php?tc=2.A.35.1.5) | -103 | Q8EHX2 | Unknown | Unknown |
| [WP_007213446.1](file:///Volumes/ESD-USB/Summer%202018/Hassan%20Submission%20PLosOne/content.html#lcl|NZ_CP012801.1_prot_WP_007213446.1_4980) |  |  | + |  |  |  |  | [2.A.35.1.5](http://tcdb.org/search/result.php?tc=2.A.35.1.5) | -98 | Q8EHX2 | Amino acids | Methionine |
| WP_008764994.1 |  |  |  | + |  |  |  | 2.A.35.1.5 | -101 | Q8EHX2 | Unknown | Unknown |
| ABI39_16710 | **+** |  |  |  |  |  |  | 2.A.35.1.6 | -81 | O07553 | Cations | Na^+^/H^+^ |
| [WP_033160637.1](file:///Volumes/ESD-USB/Summer%202018/Hassan%20Submission%20PLosOne/content.html#lcl|NZ_CP012801.1_prot_WP_033160637.1_1089) |  |  | + |  |  |  |  | [2.A.35.1.6](http://tcdb.org/search/result.php?tc=2.A.35.1.6) | -87 | O07553 | Amino acids | Methionine |
| WP_062695248.1 |  |  |  | + |  |  |  | 2.A.35.1.6 | -88 | O07553 | Unknown | Unknown |
| YP_098695.1 |  |  |  |  | **+** |  |  | 2.A.35.1.6 | -86 | O07553 | Cations | Na^+^/H^+^ |
| [WP_004296953.1](file:///Volumes/ESD-USB/Summer%202018/Hassan%20Submission%20PLosOne/content.html#lcl|NZ_CP012938.1_prot_WP_004296953.1_54) |  |  |  |  |  | + |  | [2.A.35.1.6](http://tcdb.org/search/result.php?tc=2.A.35.1.6) | -86 | O07553 | Unknown | Unknown |
| ABI39_13995 | **+** |  |  |  |  |  |  | 2.A.36.6.3 | -98 | Q87KV8 | Cations | K^+^/H^+^ |
| [WP_007218583.1](file:///Volumes/ESD-USB/Summer%202018/Hassan%20Submission%20PLosOne/content.html#lcl|NZ_CP012801.1_prot_WP_007218583.1_5092) |  |  | + |  |  |  |  | [2.A.36.6.3](http://tcdb.org/search/result.php?tc=2.A.36.6.3) | -94 | Q87KV8 | Cations | K^+^/H^+^ |
| WP_008761361.1 |  |  |  | + |  |  |  | 2.A.36.6.3 | -97 | Q87KV8 | Cations | K^+^/H^+^ |
| YP_099426.1 |  |  |  |  | **+** |  |  | 2.A.36.6.3 | -96 | Q87KV8 | Cations | K^+^/H^+^ |
| [WP_004295729.1](file:///Volumes/ESD-USB/Summer%202018/Hassan%20Submission%20PLosOne/content.html#lcl|NZ_CP012938.1_prot_WP_004295729.1_921) |  |  |  |  |  | + |  | [2.A.36.6.3](http://tcdb.org/search/result.php?tc=2.A.36.6.3) | -97 | Q87KV8 | Cations | K^+^ |
| [WP_005843270.1](file:///Volumes/ESD-USB/Summer%202018/Library/Application%20Support/Microsoft/Office/Office%202011%20AutoRecovery/content.html#lcl|NC_009614.1_prot_WP_005843270.1_2691) |  |  |  |  |  |  | + | [2.A.36.6.3](http://tcdb.org/search/result.php?tc=2.A.36.6.3) | -97 | Q87KV8 | Unknown | Unknown |
| [WP_013616941.1](file:///Volumes/ESD-USB/Summer%202018/Hassan%20Submission%20PLosOne/content.html#lcl|NC_015164.1_prot_WP_013616941.1_886) |  | + |  |  |  |  |  | [2.A.36.6.5](http://tcdb.org/search/result.php?tc=2.A.36.6.5) |  | Q9KNM9 | Unknown | Unknown |
| [WP_007213284.1](file:///Volumes/ESD-USB/Summer%202018/Hassan%20Submission%20PLosOne/content.html#lcl|NZ_CP012801.1_prot_WP_007213284.1_4537) |  |  | + |  |  |  |  | [2.A.37.1.1](http://tcdb.org/search/result.php?tc=2.A.37.1.1) | -25 | P0A754 | Amino acids | Glutathione |
| [WP_029428064.1](file:///Volumes/ESD-USB/Summer%202018/Hassan%20Submission%20PLosOne/content.html#lcl|NZ_CP012801.1_prot_WP_029428064.1_5232) |  |  | + |  |  |  |  | [2.A.37.1.1](http://tcdb.org/search/result.php?tc=2.A.37.1.1) | -22 | P0A754 | Amino acids | Glutathione |
| WP_008761351.1 |  |  |  | + |  |  |  | 2.A.37.1.2 | -23 | P0A756 | Cations | K^+^ |
| [WP_004295716.1](file:///Volumes/ESD-USB/Summer%202018/Hassan%20Submission%20PLosOne/content.html#lcl|NZ_CP012938.1_prot_WP_004295716.1_933) |  |  |  |  |  | + |  | [2.A.37.1.2](http://tcdb.org/search/result.php?tc=2.A.37.1.2) | -25 | P0A756 | Unknown | Unknown |
| ABI39_01105 | **+** |  |  |  |  |  |  | 2.A.37.2.5 | 0 | B3EE21 | Unknown | Unknown |
| [WP_013619215.1](file:///Volumes/ESD-USB/Summer%202018/Hassan%20Submission%20PLosOne/content.html#lcl|NC_015164.1_prot_WP_013619215.1_3270) |  | + |  |  |  |  |  | [2.A.37.2.5](http://tcdb.org/search/result.php?tc=2.A.37.2.5) | 0 | B3EE21 | Cations | Na^+^/H^+^ |
| [WP_007210690.1](file:///Volumes/ESD-USB/Summer%202018/Hassan%20Submission%20PLosOne/content.html#lcl|NZ_CP012801.1_prot_WP_007210690.1_2099) |  |  | + |  |  |  |  | [2.A.37.2.5](http://tcdb.org/search/result.php?tc=2.A.37.2.5) | 0 | B3EE21 | Cations | Na^+^/H^+^ |
| WP_062695794.1 |  |  |  | + |  |  |  | 2.A.37.2.5 | 0 | B3EE21 | Cations | Na^+^/H^+^ |
| [WP_011964666.1](file:///Volumes/ESD-USB/Summer%202018/Library/Application%20Support/Microsoft/Office/Office%202011%20AutoRecovery/content.html#lcl|NC_009614.1_prot_WP_011964666.1_206) |  |  |  |  |  |  | + | [2.A.37.2.5](http://tcdb.org/search/result.php?tc=2.A.37.2.5) | 0 | B3EE21 | Unknown | Unknown |
| YP_099838.1 |  |  |  |  | **+** |  |  | 2.A.37.2.6 | -95 | Q6ML49 | Cations | Na^+^/H^+^ |
| WP_048692293.1 |  |  |  | + |  |  |  | 2.A.37.2.6 | -92 | Q6ML49 | Cations | Na+/H+ |
| YP_100632.1 |  |  |  |  | **+** |  |  | 2.A.37.4.2 | -87 | Q9SUQ7 | Cations | K^+^/H^+^ |
| WP_011107975.1 |  |  |  | + |  |  |  | 2.A.37.4.2 | -82 | Q9SUQ7 | Cations | K+ |
| ABI39_08555 | **+** |  |  |  |  |  |  | 2.A.38.1.1 | -48 | P0AGI8 | Cations | K+ |
| [WP_013617756.1](file:///Volumes/ESD-USB/Summer%202018/Hassan%20Submission%20PLosOne/content.html#lcl|NC_015164.1_prot_WP_013617756.1_1729) |  | + |  |  |  |  |  | [2.A.38.1.1](http://tcdb.org/search/result.php?tc=2.A.38.1.1) | -48 | P0AGI8 | Cations | K^+^ |
| [WP_007212463.1](file:///Volumes/ESD-USB/Summer%202018/Hassan%20Submission%20PLosOne/content.html#lcl|NZ_CP012801.1_prot_WP_007212463.1_3855) |  |  | + |  |  |  |  | [2.A.38.1.1](http://tcdb.org/search/result.php?tc=2.A.38.1.1) | -56 | P0AGI8 | Cations | K^+^ |
| WP_008764647.1 |  |  |  | + |  |  |  | 2.A.38.1.1 | -92 | P0AFZ7 | Cations | K^+^ |
| WP_008764324.1 |  |  |  | + |  |  |  | 2.A.38.1.1 | -52 | P0AGI8 | Cations | K^+^ |
| YP_100369.1 |  |  |  |  | **+** |  |  | 2.A.38.1.1 | -92 | P0AFZ7 | Cations | K^+^/H^+^ |
| YP_098156.1 |  |  |  |  | **+** |  |  | 2.A.38.1.1 | -92 | P0AFZ7 | Cations | K^+^/H^+^ |
| YP_098157.1 |  |  |  |  | **+** |  |  | 2.A.38.1.1 | -52 | P0AGI8 | Cations | K^+^/H^+^ |
| [WP_004301980.1](file:///Volumes/ESD-USB/Summer%202018/Hassan%20Submission%20PLosOne/content.html#lcl|NZ_CP012938.1_prot_WP_004301980.1_4531) |  |  |  |  |  | + |  | [2.A.38.1.1](http://tcdb.org/search/result.php?tc=2.A.38.1.1) | -92 | P0AFZ7 | Cations | K^+^ |
| [WP_004301492.1](file:///Volumes/ESD-USB/Summer%202018/Hassan%20Submission%20PLosOne/content.html#lcl|NZ_CP012938.1_prot_WP_004301492.1_4173) |  |  |  |  |  | + |  | [2.A.38.1.1](http://tcdb.org/search/result.php?tc=2.A.38.1.1) | -52 | P0AGI8 | Cations | K^+^ |
| [WP_005842854.1](file:///Volumes/ESD-USB/Summer%202018/Library/Application%20Support/Microsoft/Office/Office%202011%20AutoRecovery/content.html#lcl|NC_009614.1_prot_WP_005842854.1_1742) |  |  |  |  |  |  | + | [2.A.38.1.1](http://tcdb.org/search/result.php?tc=2.A.38.1.1) | -49 | P0AGI8 | Unknown | Unknown |
| ABI39_08550 | **+** |  |  |  |  |  |  | 2.A.38.1.3 | -99 | Q6T3V7 | Cations | K^+^ |
| ABI39_18715 | **+** |  |  |  |  |  |  | 2.A.38.1.3 | -94 | Q6T3V7 | Cations | K^+^ |
| [WP_013619510.1](file:///Volumes/ESD-USB/Summer%202018/Hassan%20Submission%20PLosOne/content.html#lcl|NC_015164.1_prot_WP_013619510.1_3558) |  | + |  |  |  |  |  | [2.A.38.1.3](http://tcdb.org/search/result.php?tc=2.A.38.1.3) | -100 | Q6T3V7 | Cations | K^+^ |
| [WP_007212462.1](file:///Volumes/ESD-USB/Summer%202018/Hassan%20Submission%20PLosOne/content.html#lcl|NZ_CP012801.1_prot_WP_007212462.1_3854) |  |  | + |  |  |  |  | [2.A.38.1.3](http://tcdb.org/search/result.php?tc=2.A.38.1.3) | -96 | Q6T3V7 | Cations | K^+^ |
| WP_008764323.1 |  |  |  | + |  |  |  | 2.A.38.1.3 | -95 | Q6T3V7 | Cations | K^+^ |
| [WP_004320973.1](file:///Volumes/ESD-USB/Summer%202018/Hassan%20Submission%20PLosOne/content.html#lcl|NZ_CP012938.1_prot_WP_004320973.1_4172) |  |  |  |  |  | + |  | [2.A.38.1.3](http://tcdb.org/search/result.php?tc=2.A.38.1.3) | -98 | Q6T3V7 | Cations | K^+^ |
| [WP_005842856.1](file:///Volumes/ESD-USB/Summer%202018/Library/Application%20Support/Microsoft/Office/Office%202011%20AutoRecovery/content.html#lcl|NC_009614.1_prot_WP_005842856.1_1741) |  |  |  |  |  |  | + | [2.A.38.1.3](http://tcdb.org/search/result.php?tc=2.A.38.1.3) | -100 | Q6T3V7 | Unknown | Unknown |
| [WP_012055899.1](file:///Volumes/ESD-USB/Summer%202018/Library/Application%20Support/Microsoft/Office/Office%202011%20AutoRecovery/content.html#lcl|NC_009614.1_prot_WP_012055899.1_3693) |  |  |  |  |  |  | + | [2.A.38.1.3](http://tcdb.org/search/result.php?tc=2.A.38.1.3) | -94 | Q6T3V7 | Unknown | Unknown |
| [WP_029427034.1](file:///Volumes/ESD-USB/Summer%202018/Hassan%20Submission%20PLosOne/content.html#lcl|NZ_CP012801.1_prot_WP_029427034.1_180) |  |  | + |  |  |  |  | [2.A.38.4.5](http://tcdb.org/search/result.php?tc=2.A.38.4.5) | -06 | P73948 | Cations | K^+^ |
| [WP_026367077.1](file:///Volumes/ESD-USB/Summer%202018/Hassan%20Submission%20PLosOne/content.html#lcl|NZ_CP012801.1_prot_WP_026367077.1_2943) |  |  | + |  |  |  |  | [2.A.38.4.5](http://tcdb.org/search/result.php?tc=2.A.38.4.5) | -05 | P73948 | Cations | K^+^ |
| [WP_029426133.1](file:///Volumes/ESD-USB/Summer%202018/Hassan%20Submission%20PLosOne/content.html#lcl|NZ_CP012801.1_prot_WP_029426133.1_2335) |  |  | + |  |  |  |  | [2.A.38.4.5](http://tcdb.org/search/result.php?tc=2.A.38.4.5) | -05 | P73948 | Cations | K^+^ |
| WP_055300240.1 |  |  |  | + |  |  |  | 2.A.38.4.5 | -09 | P73948 | Cations | K^+^ |
| WP_008759975.1 |  |  |  | + |  |  |  | 2.A.38.4.5 | -06 | P73948 | Cations | K^+^ |
| WP_062694200.1 |  |  |  | + |  |  |  | 2.A.38.4.5 | -06 | P73948 | Cations | K^+^ |
| WP_008767823.1 |  |  |  | + |  |  |  | 2.A.38.4.5 | -05 | P73948 | Cations | K^+^ |
| WP_062694180.1 |  |  |  | + |  |  |  | 2.A.38.4.5 | -05 | P73948 | Cations | K^+^ |
| WP_062695837.1 |  |  |  | + |  |  |  | 2.A.38.4.5 | -05 | P73948 | Cations | K^+^ |
| WP_062695838.1 |  |  |  | + |  |  |  | 2.A.38.4.5 | -05 | P73948 | Cations | K^+^ |
| [WP_004302240.1](file:///Volumes/ESD-USB/Summer%202018/Hassan%20Submission%20PLosOne/content.html#lcl|NZ_CP012938.1_prot_WP_004302240.1_4729) |  |  |  |  |  | + |  | [2.A.38.4.5](http://tcdb.org/search/result.php?tc=2.A.38.4.5) | -09 | P73948 | Unknown | Unknown |
| [WP_004297498.1](file:///Volumes/ESD-USB/Summer%202018/Hassan%20Submission%20PLosOne/content.html#lcl|NZ_CP012938.1_prot_WP_004297498.1_236) |  |  |  |  |  | + |  | [2.A.38.4.5](http://tcdb.org/search/result.php?tc=2.A.38.4.5) | -08 | P73948 | Unknown | Unknown |
| ABI39_12220 | **+** |  |  |  |  |  |  | 2.A.38.4.6 | -30 | G8V089 | Cations | K^+^ |
| [WP_029427676.1](file:///Volumes/ESD-USB/Summer%202018/Hassan%20Submission%20PLosOne/content.html#lcl|NZ_CP012801.1_prot_WP_029427676.1_4474) |  |  | + |  |  |  |  | [2.A.38.4.6](http://tcdb.org/search/result.php?tc=2.A.38.4.6) | -29 | G8V089 | Cations | K^+^ |
| WP_008766869.1 |  |  |  | + |  |  |  | 2.A.38.4.6 | -25 | G8V089 | Cations | K^+^ |
| YP_098528.1 |  |  |  |  | **+** |  |  | 2.A.38.4.6 | -27 | G8V089 | Cations | K^+^ |
| [WP_004302197.1](file:///Volumes/ESD-USB/Summer%202018/Hassan%20Submission%20PLosOne/content.html#lcl|NZ_CP012938.1_prot_WP_004302197.1_4689) |  |  |  |  |  | + |  | [2.A.38.4.6](http://tcdb.org/search/result.php?tc=2.A.38.4.6) | -25 | G8V089 | Unknown | Unknown |
| WP_008765567.1 |  |  |  | + |  |  |  | 2.A.40.1.4 | -91 | P39766 | Nucleobases | Uracil |
| YP_099427.1 |  |  |  |  | **+** |  |  | 2.A.40.1.4 | -92 | P39766 | Nucleobases | Uracil |
| [WP_008777334.1](file:///Volumes/ESD-USB/Summer%202018/Hassan%20Submission%20PLosOne/content.html#lcl|NZ_CP012938.1_prot_WP_008777334.1_920) |  |  |  |  |  | + |  | [2.A.40.1.4](http://tcdb.org/search/result.php?tc=2.A.40.1.4) | -91 | P39766 | Nucleobases | Uracil |
| ABI39_12605 | **+** |  |  |  |  |  |  | 2.A.40.4.2 | -149 | P0AGM9 | Nucleobases | Xanthine |
| [WP_013618031.1](file:///Volumes/ESD-USB/Summer%202018/Hassan%20Submission%20PLosOne/content.html#lcl|NC_015164.1_prot_WP_013618031.1_2007) |  | + |  |  |  |  |  | [2.A.40.4.2](http://tcdb.org/search/result.php?tc=2.A.40.4.2) | -146 | P0AGM9 | Nucleobases | Purines |
| WP_062695536.1 |  |  |  | + |  |  |  | 2.A.40.4.2 | -147 | P0AGM9 | Nucleobases | Adenine/Guanine |
| YP_100856.1 |  |  |  |  | **+** |  |  | 2.A.40.4.2 | -147 | P0AGM9 | Nucleobases | Xanthine |
| [WP_004301959.1](file:///Volumes/ESD-USB/Summer%202018/Hassan%20Submission%20PLosOne/content.html#lcl|NZ_CP012938.1_prot_WP_004301959.1_4512) |  |  |  |  |  | + |  | [2.A.40.4.2](http://tcdb.org/search/result.php?tc=2.A.40.4.2) | -149 | P0AGM9 | Nucleosides | Xantosine |
| [WP_011965637.1](file:///Volumes/ESD-USB/Summer%202018/Library/Application%20Support/Microsoft/Office/Office%202011%20AutoRecovery/content.html#lcl|NC_009614.1_prot_WP_011965637.1_2484) |  |  |  |  |  |  | + | [2.A.40.4.2](http://tcdb.org/search/result.php?tc=2.A.40.4.2) | -148 | P0AGM9 | Nucleobases | Purines |
| WP_008762675.1 |  |  |  | + |  |  |  | 2.A.47.1.1 | -41 | Q2FMC1 | Anions | Anions |
| ABI39_02270 | **+** |  |  |  |  |  |  | 2.A.47.4.5 | -42 | Q9K7H7 | Cations | Ions |
| [WP_013618923.1](file:///Volumes/ESD-USB/Summer%202018/Hassan%20Submission%20PLosOne/content.html#lcl|NC_015164.1_prot_WP_013618923.1_2960) |  | + |  |  |  |  |  | [2.A.47.4.5](http://tcdb.org/search/result.php?tc=2.A.47.4.5) | -47 | Q9K7H7 | Cations | K^+^ |
| WP_008766078.1 |  |  |  | + |  |  |  | 2.A.47.4.6 | -63 | D5AQ60 | Anions |  |
| [WP_004297521.1](file:///Volumes/ESD-USB/Summer%202018/Hassan%20Submission%20PLosOne/content.html#lcl|NZ_CP012938.1_prot_WP_004297521.1_253) |  |  |  |  |  | + |  | [2.A.47.4.6](http://tcdb.org/search/result.php?tc=2.A.47.4.6) | -66 | D5AQ60 | Anions | MoO^2−^_4_ |
| ABI39_12145 | **+** |  |  |  |  |  |  | 2.A.47.4.8 | -130 | P0AFU2 | Unknown | Unknown |
| [WP_013616222.1](file:///Volumes/ESD-USB/Summer%202018/Hassan%20Submission%20PLosOne/content.html#lcl|NC_015164.1_prot_WP_013616222.1_144) |  | + |  |  |  |  |  | [2.A.47.4.8](http://tcdb.org/search/result.php?tc=2.A.47.4.8) | -132 | P0AFU2 | Unknown | Unknown |
| [WP_007213356.1](file:///Volumes/ESD-USB/Summer%202018/Hassan%20Submission%20PLosOne/content.html#lcl|NZ_CP012801.1_prot_WP_007213356.1_4590) |  |  | + |  |  |  |  | [2.A.47.4.8](http://tcdb.org/search/result.php?tc=2.A.47.4.8) | -132 | P0AFU2 | Unknown | Unknown |
| WP_011107313.1 |  |  |  | + |  |  |  | 2.A.47.4.8 | -132 | P0AFU2 | Unknown | Unknown |
| [WP_004300098.1](file:///Volumes/ESD-USB/Summer%202018/Hassan%20Submission%20PLosOne/content.html#lcl|NZ_CP012938.1_prot_WP_004300098.1_1164) |  |  |  |  |  | + |  | [2.A.47.4.8](http://tcdb.org/search/result.php?tc=2.A.47.4.8) | -133 | P0AFU2 | Cations | K^+^ |
| [WP_007213237.1](file:///Volumes/ESD-USB/Summer%202018/Hassan%20Submission%20PLosOne/content.html#lcl|NZ_CP012801.1_prot_WP_007213237.1_4471) |  |  | + |  |  |  |  | [2.A.49.5.5](http://tcdb.org/search/result.php?tc=2.A.49.5.5) | -31 | A8ALD3 | Anions | Cl^-^ |
| WP_008760366.1 |  |  |  | + |  |  |  | 2.A.49.5.5 | -30 | A8ALD3 | Unknown | Unknown |
| YP_098524.1 |  |  |  |  | + |  |  | 2.A.49.5.5 | -28 | A8ALD3 | Anions | Cl^-^ |
| [WP_007210121.1](file:///Volumes/ESD-USB/Summer%202018/Hassan%20Submission%20PLosOne/content.html#lcl|NZ_CP012801.1_prot_WP_007210121.1_1648) |  |  | + |  |  |  |  | [2.A.49.6.1](http://tcdb.org/search/result.php?tc=2.A.49.6.1) | -34 | P74477 | Anions | Cl^-^ |
| YP_101180.1 |  |  |  |  | + |  |  | 2.A.49.6.1 | -34 | P74477 | Anions | Cl- |
| [WP_013617640.1](file:///Volumes/ESD-USB/Summer%202018/Hassan%20Submission%20PLosOne/content.html#lcl|NC_015164.1_prot_WP_013617640.1_1608) |  | + |  |  |  |  |  | [2.A.50.2.1](http://tcdb.org/search/result.php?tc=2.A.50.2.1) | -37 | P39580 | Amino Acids | Alanine |
| ABI39_00415 | **+** |  |  |  |  |  |  | 2.A.51.1.6 | -31 | Q73LW8 | Unknown | Unknown |
| ABI39_00420 | **+** |  |  |  |  |  |  | 2.A.51.1.6 | -26 | Q73LW9 | Unknown | Unknown |
| [WP_013616820.1](file:///Volumes/ESD-USB/Summer%202018/Hassan%20Submission%20PLosOne/content.html#lcl|NC_015164.1_prot_WP_013616820.1_762) |  | + |  |  |  |  |  | [2.A.51.1.6](http://tcdb.org/search/result.php?tc=2.A.51.1.6) | -31 | Q73LW8 | Anions | CrO^2−^_4_ |
| [WP_013616821.1](file:///Volumes/ESD-USB/Summer%202018/Hassan%20Submission%20PLosOne/content.html#lcl|NC_015164.1_prot_WP_013616821.1_763) |  | + |  |  |  |  |  | [2.A.51.1.6](http://tcdb.org/search/result.php?tc=2.A.51.1.6) | -28 | Q73LW9 | Anions | CrO^2−^_4_ |
| [WP_007217394.1](file:///Volumes/ESD-USB/Summer%202018/Hassan%20Submission%20PLosOne/content.html#lcl|NZ_CP012801.1_prot_WP_007217394.1_1249) |  |  | + |  |  |  |  | [2.A.51.1.6](http://tcdb.org/search/result.php?tc=2.A.51.1.6) | -36 | Q73LW8 | Anions | CrO^2−^_4_ |
| [WP_007214814.1](file:///Volumes/ESD-USB/Summer%202018/Hassan%20Submission%20PLosOne/content.html#lcl|NZ_CP012801.1_prot_WP_007214814.1_1250) |  |  | + |  |  |  |  | [2.A.51.1.6](http://tcdb.org/search/result.php?tc=2.A.51.1.6) | -28 | Q73LW8 | Anions | CrO^2−^_4_ |
| WP_055221371.1 |  |  |  | + |  |  |  | 2.A.51.1.6 | -35 | Q73LW8 | Anions | CrO^2−^_4_ |
| WP_011108481.1 |  |  |  | + |  |  |  | 2.A.51.1.6 | -29 | Q73LW8 | Anions | CrO^2−^_4_ |
| WP_008763311.1 |  |  |  | + |  |  |  | 2.A.51.1.6 | -27 | Q73LW8 | Anions | CrO^2−^_4_ |
| WP_008761975.1 |  |  |  | + |  |  |  | 2.A.51.1.6 | -21 | Q73LW8 | Anions | CrO^2−^_4_ |
| YP_100595.1 |  |  |  |  | + |  |  | 2.A.51.1.6 | -37 | Q73LW8 | Anions | Chromate |
| YP_100596.1 |  |  |  |  | + |  |  | 2.A.51.1.6 | -26 | Q73LW9 | Anions | Chromate |
| [WP_004300957.1](file:///Volumes/ESD-USB/Summer%202018/Hassan%20Submission%20PLosOne/content.html#lcl|NZ_CP012938.1_prot_WP_004300957.1_1717) |  |  |  |  |  | + |  | [2.A.51.1.6](http://tcdb.org/search/result.php?tc=2.A.51.1.6) | -35 | Q73LW8 | Cations | CrO^2−^_4_ |
| [WP_004296411.1](file:///Volumes/ESD-USB/Summer%202018/Hassan%20Submission%20PLosOne/content.html#lcl|NZ_CP012938.1_prot_WP_004296411.1_2709) |  |  |  |  |  | + |  | [2.A.51.1.6](http://tcdb.org/search/result.php?tc=2.A.51.1.6) | -28 | Q73LW8 | Cations | CrO^2−^_4_ |
| [WP_005843001.1](file:///Volumes/ESD-USB/Summer%202018/Library/Application%20Support/Microsoft/Office/Office%202011%20AutoRecovery/content.html#lcl|NC_009614.1_prot_WP_005843001.1_82) |  |  |  |  |  |  | + | [2.A.51.1.6](http://tcdb.org/search/result.php?tc=2.A.51.1.6) | -31 | Q73LW8 | Cations | CrO^2−^_4_ |
| [WP_005843003.1](file:///Volumes/ESD-USB/Summer%202018/Library/Application%20Support/Microsoft/Office/Office%202011%20AutoRecovery/content.html#lcl|NC_009614.1_prot_WP_005843003.1_83) |  |  |  |  |  |  | + | [2.A.51.1.6](http://tcdb.org/search/result.php?tc=2.A.51.1.6) | -25 | Q73LW9 | Cations | CrO^2−^_4_ |
| [WP_007216501.1](file:///Volumes/ESD-USB/Summer%202018/Hassan%20Submission%20PLosOne/content.html#lcl|NZ_CP012801.1_prot_WP_007216501.1_2681) |  |  | + |  |  |  |  | [2.A.53.3.9](http://tcdb.org/search/result.php?tc=2.A.53.3.9) | -105 | A1JRS3 | Anions | SO^2-^_4_ |
| WP_008767420.1 |  |  |  | + |  |  |  | 2.A.53.3.9 | -104 | A1JRS3 | Unknown | Unknown |
| [WP_004298804.1](file:///Volumes/ESD-USB/Summer%202018/Hassan%20Submission%20PLosOne/content.html#lcl|NZ_CP012938.1_prot_WP_004298804.1_3103) |  |  |  |  |  | + |  | [2.A.53.3.9](http://tcdb.org/search/result.php?tc=2.A.53.3.9) | -103 | A1JRS3 | Anions | SO^2-^_4_ |
| [WP_005844072.1](file:///Volumes/ESD-USB/Summer%202018/Library/Application%20Support/Microsoft/Office/Office%202011%20AutoRecovery/content.html#lcl|NC_009614.1_prot_WP_005844072.1_1120) |  |  |  |  |  |  | + | [2.A.53.3.9](http://tcdb.org/search/result.php?tc=2.A.53.3.9) | -104 | A1JRS3 | Unknown | Unknown |
| [WP_007213741.1](file:///Volumes/ESD-USB/Summer%202018/Hassan%20Submission%20PLosOne/content.html#lcl|NZ_CP012801.1_prot_WP_007213741.1_499) |  |  | + |  |  |  |  | [2.A.55.2.6](http://tcdb.org/search/result.php?tc=2.A.55.2.6) | -67 | Q89K67 | Cations | Mn^2+^ |
| ABI39_13990 | **+** |  |  |  |  |  |  | 2.A.55.3.3 | -63 | P96593 | Cations | Mg^2+^ |
| WP_032840969.1 |  |  |  | + |  |  |  | 2.A.55.3.3 | -64 | P96593 | Cations | Mn^2+^ |
| YP_099844.1_2561 |  |  |  |  | + |  |  | 2.A.55.3.3 | -65 | P96593 | Cations | Mn^2+^ |
| [WP_004295597.1](file:///Volumes/ESD-USB/Summer%202018/Hassan%20Submission%20PLosOne/content.html#lcl|NZ_CP012938.1_prot_WP_004295597.1_1038) |  |  |  |  |  | + |  | [2.A.55.3.3](http://tcdb.org/search/result.php?tc=2.A.55.3.3) | -64 | P96593 | Cations | Mn^2+^ |
| [WP_005843268.1](file:///Volumes/ESD-USB/Summer%202018/Library/Application%20Support/Microsoft/Office/Office%202011%20AutoRecovery/content.html#lcl|NC_009614.1_prot_WP_005843268.1_2690) |  |  |  |  |  |  | + | [2.A.55.3.3](http://tcdb.org/search/result.php?tc=2.A.55.3.3) | -62 | P96593 | Cations | Mn^2+^ |
| [WP_007219478.1](file:///Volumes/ESD-USB/Summer%202018/Hassan%20Submission%20PLosOne/content.html#lcl|NZ_CP012801.1_prot_WP_007219478.1_3122) |  |  | + |  |  |  |  | [2.A.58.2.2](http://tcdb.org/search/result.php?tc=2.A.58.2.2) | -82 | M7AKZ4 | Unknown | Unknown |
| WP_008765170.1 |  |  |  | + |  |  |  | 2.A.58.2.2 | -78 | M7AKZ4 | Unknown | Unknown |
| [WP_012056055.1](file:///Volumes/ESD-USB/Summer%202018/Library/Application%20Support/Microsoft/Office/Office%202011%20AutoRecovery/content.html#lcl|NC_009614.1_prot_WP_012056055.1_4087) |  |  |  |  |  |  | + | [2.A.59.1.5](http://tcdb.org/search/result.php?tc=2.A.59.1.5) | -175 | A6TP80 | Anions | AsO_3_^-3^ |
| WP_062695061.1 |  |  |  | + |  |  |  | 2.A.59.1.6 | -170 | A6TLY3 | Anions | AsO_3_^-3^ |
| ABI39_18845 | **+** |  |  |  |  |  |  | 2.A.64.1.1 | -33 | P69423 | Unknown | Unknown |
| [WP_007212471.1](file:///Volumes/ESD-USB/Summer%202018/Hassan%20Submission%20PLosOne/content.html#lcl|NZ_CP012801.1_prot_WP_007212471.1_3863) |  |  | + |  |  |  |  | [2.A.64.1.1](http://tcdb.org/search/result.php?tc=2.A.64.1.1) | -31 | P69423 | Unknown | Unknown |
| ABI39_19200 | **+** |  |  |  |  |  |  | 2.A.64.1.3 | -10 | Q6ML26 | unknown | unknown |
| ABI39_19195 | **+** |  |  |  |  |  |  | 2.A.64.1.3 | -13 | Q6MGQ9 | unknown | unknown |
| WP_041584029.1 |  | + |  |  |  |  |  | 2.A.64.1.3 | -34 | Q6MGQ9 | Unknown | Unknown |
| [WP_005852112.1](file:///Volumes/ESD-USB/Summer%202018/Library/Application%20Support/Microsoft/Office/Office%202011%20AutoRecovery/content.html#lcl|NC_009614.1_prot_WP_005852112.1_3720) |  |  |  |  |  |  | + | [2.A.64.1.3](http://tcdb.org/search/result.php?tc=2.A.64.1.3) | -34 | Q6MGQ9 | Unknown | Unknown |
| WP_013618491.1 |  | + |  |  |  |  |  | 2.A.64.2.1 | -09 | Q9LKU2 | Unknown | Unknown |
| YP_098176.1 |  |  |  |  | + |  |  | 2.A.64.2.1 | -06 | Q9LKU2 | Unknown | Unknown |
| YP_098175.1 |  |  |  |  | + |  |  | 2.A.64.3.2 | -30 | O05523 | Unknown | Unknown |
| [WP_026367288.1](file:///Volumes/ESD-USB/Summer%202018/Hassan%20Submission%20PLosOne/content.html#lcl|NZ_CP012801.1_prot_WP_026367288.1_3382) |  |  | + |  |  |  |  | [2.A.66.1.1](http://tcdb.org/search/result.php?tc=2.A.66.1.1) | -58 | O82855 | Drugs | Fluruquinolones/Aminoglycosides |
| WP_062695760.1 |  |  |  | + |  |  |  | 2.A.66.1.1 | -59 | O82855 | Drugs | Fluoroquinolones/Ethidium |
| [WP_004299933.1](file:///Volumes/ESD-USB/Summer%202018/Hassan%20Submission%20PLosOne/content.html#lcl|NZ_CP012938.1_prot_WP_004299933.1_3768) |  |  |  |  |  | + |  | [2.A.66.1.1](http://tcdb.org/search/result.php?tc=2.A.66.1.1) | -60 | O82855 | Drugs | Aminoglycosides/Macrolides/Fluoroquinolones |
| [WP_013616441.1](file:///Volumes/ESD-USB/Summer%202018/Hassan%20Submission%20PLosOne/content.html#lcl|NC_015164.1_prot_WP_013616441.1_369) |  | + |  |  |  |  |  | [2.A.66.1.2](http://tcdb.org/search/result.php?tc=2.A.66.1.2) | -13 | Q9KRU4 | Drugs | Fluoroquinolones/Acriflavin/Aminoglycosides |
| [WP_008667662.1](file:///Volumes/ESD-USB/Summer%202018/Library/Application%20Support/Microsoft/Office/Office%202011%20AutoRecovery/content.html#lcl|NC_009614.1_prot_WP_008667662.1_377) |  |  |  |  |  |  | + | [2.A.66.1.2](http://tcdb.org/search/result.php?tc=2.A.66.1.2) | -56 | Q9KRU4 | Drugs | Fluoroquinolones/Streptomycin/Ethidium Bromide |
| [WP_012055883.1](file:///Volumes/ESD-USB/Summer%202018/Library/Application%20Support/Microsoft/Office/Office%202011%20AutoRecovery/content.html#lcl|NC_009614.1_prot_WP_012055883.1_3670) |  |  |  |  |  |  | + | [2.A.66.1.2](http://tcdb.org/search/result.php?tc=2.A.66.1.2) | -14 | Q9KRU4 | Drugs | Fluoroquinolones/Streptomycin/Ethidium Bromide |
| [WP_013617287.1](file:///Volumes/ESD-USB/Summer%202018/Hassan%20Submission%20PLosOne/content.html#lcl|NC_015164.1_prot_WP_013617287.1_1256) |  | + |  |  |  |  |  | [2.A.66.1.6](http://tcdb.org/search/result.php?tc=2.A.66.1.36) | -73 | Q8XT32 | Drugs | Drugs/Detergents |
| [WP_029429147.1](file:///Volumes/ESD-USB/Summer%202018/Hassan%20Submission%20PLosOne/content.html#lcl|NZ_CP012801.1_prot_WP_029429147.1_1412) |  |  | + |  |  |  |  | [2.A.66.1.7](http://tcdb.org/search/result.php?tc=2.A.66.1.7) | -14 | Q93HR7 | Drugs | Acriflavin/TTP |
| YP_100778.1 |  |  |  |  | + |  |  | 2.A.66.1.10 | -10 | Q9JV27 | Drugs | Fluoroquinolones |
| ABI39_08035 | **+** |  |  |  |  |  |  | 2.A.66.1.28 | -41 | Q9WZS2 | Unknown | Unknown |
| ABI39_17420 | **+** |  |  |  |  |  |  | 2.A.66.1.28 | -34 | Q9WZS2 | Unknown | Unknown |
| [WP_029428933.1](file:///Volumes/ESD-USB/Summer%202018/Hassan%20Submission%20PLosOne/content.html#lcl|NZ_CP012801.1_prot_WP_029428933.1_647) |  |  | + |  |  |  |  | [2.A.66.1.28](http://tcdb.org/search/result.php?tc=2.A.66.1.28) | -40 | Q9WZS2 | Unknown | Unknown |
| [WP_007213592.1](file:///Volumes/ESD-USB/Summer%202018/Hassan%20Submission%20PLosOne/content.html#lcl|NZ_CP012801.1_prot_WP_007213592.1_620) |  |  | + |  |  |  |  | [2.A.66.1.28](http://tcdb.org/search/result.php?tc=2.A.66.1.28) | -38 | Q9WZS2 | Unknown | Unknown |
| [WP_007213690.1](file:///Volumes/ESD-USB/Summer%202018/Hassan%20Submission%20PLosOne/content.html#lcl|NZ_CP012801.1_prot_WP_007213690.1_542) |  |  | + |  |  |  |  | [2.A.66.1.28](http://tcdb.org/search/result.php?tc=2.A.66.1.28) | -30 | Q9WZS2 | Unknown | Unknown |
| WP_008763445.1 |  |  |  | + |  |  |  | 2.A.66.1.28 | -35 | Q9WZS2 | Unknown | Unknown |
| YP_099098.1 |  |  |  |  | + |  |  | 2.A.66.1.28 | -38 | Q9WZS2 | Drugs | Drugs |
| YP_099180.1 |  |  |  |  | + |  |  | 2.A.66.1.28 | -36 | Q9WZS2 | Drugs | Drugs |
| [WP_004300349.1](file:///Volumes/ESD-USB/Summer%202018/Hassan%20Submission%20PLosOne/content.html#lcl|NZ_CP012938.1_prot_WP_004300349.1_454) |  |  |  |  |  | + |  | [2.A.66.1.28](http://tcdb.org/search/result.php?tc=2.A.66.1.28) | -35 | Q9WZS2 | Unknown | Unknown |
| [WP_004299975.1](file:///Volumes/ESD-USB/Summer%202018/Hassan%20Submission%20PLosOne/content.html#lcl|NZ_CP012938.1_prot_WP_004299975.1_1091) |  |  |  |  |  | + |  | [2.A.66.1.28](http://tcdb.org/search/result.php?tc=2.A.66.1.28) | -32 | Q9WZS2 | Unknown | Unknown |
| ABI39_01435 | **+** |  |  |  |  |  |  | 2.A.66.1.32 | -87 | Q9KAX3 | Unknown | Unknown |
| ABI39_14000 | **+** |  |  |  |  |  |  | 2.A.66.1.32 | -83 | Q9KAX3 | Unknown | Unknown |
| [WP_013617412.1](file:///Volumes/ESD-USB/Summer%202018/Hassan%20Submission%20PLosOne/content.html#lcl|NC_015164.1_prot_WP_013617412.1_1380) |  | + |  |  |  |  |  | [2.A.66.1.32](http://tcdb.org/search/result.php?tc=2.A.66.1.32) | -88 | Q9KAX3 | Dyes | Rhodamine |
| [WP_013619203.1](file:///Volumes/ESD-USB/Summer%202018/Hassan%20Submission%20PLosOne/content.html#lcl|NC_015164.1_prot_WP_013619203.1_3257) |  | + |  |  |  |  |  | [2.A.66.1.32](http://tcdb.org/search/result.php?tc=2.A.66.1.32) | -87 | Q9KAX3 | Dyes | Rhodamine |
| [WP_052306028.1](file:///Volumes/ESD-USB/Summer%202018/Hassan%20Submission%20PLosOne/content.html#lcl|NC_015164.1_prot_WP_052306028.1_1785) |  | + |  |  |  |  |  | [2.A.66.1.32](http://tcdb.org/search/result.php?tc=2.A.66.1.32) | -54 | Q9KAX3 | Dyes | Rhodamine |
| [WP_025726037.1](file:///Volumes/ESD-USB/Summer%202018/Hassan%20Submission%20PLosOne/content.html#lcl|NZ_CP012801.1_prot_WP_025726037.1_1489) |  |  | + |  |  |  |  | [2.A.66.1.32](http://tcdb.org/search/result.php?tc=2.A.66.1.32) | -91 | Q9KAX3 | Sugars | Oligosaccharides |
| [WP_022209294.1](file:///Volumes/ESD-USB/Summer%202018/Hassan%20Submission%20PLosOne/content.html#lcl|NZ_CP012801.1_prot_WP_022209294.1_307) |  |  | + |  |  |  |  | [2.A.66.1.32](http://tcdb.org/search/result.php?tc=2.A.66.1.32) | -89 | Q9KAX3 | Sugars | Oligosaccharides |
| [WP_029427422.1](file:///Volumes/ESD-USB/Summer%202018/Hassan%20Submission%20PLosOne/content.html#lcl|NZ_CP012801.1_prot_WP_029427422.1_3511) |  |  | + |  |  |  |  | [2.A.66.1.32](http://tcdb.org/search/result.php?tc=2.A.66.1.32) | -67 | Q9KAX3 | Sugars | Oligosaccharides |
| WP_007218302.1 |  |  | + |  |  |  |  | [2.A.66.1.32](http://tcdb.org/search/result.php?tc=2.A.66.1.32) | -59 | Q9KAX3 | Sugars | Oligosaccharides |
| WP_008761372.1 |  |  |  | + |  |  |  | 2.A.66.1.32 | -85 | Q9KAX3 | Dye | Rhodamine |
| WP_008766607.1 |  |  |  | + |  |  |  | 2.A.66.1.32 | -84 | Q9KAX3 | Dye | Rhodamine |
| WP_054959726.1 |  |  |  | + |  |  |  | 2.A.66.1.32 | -59 | Q9KAX3 | Dye | Rhodamine |
| YP_099440.1 |  |  |  |  | + |  |  | 2.A.66.1.32 | -87 | Q9KAX3 | Drugs | Rhodamine |
| YP_101085.1 |  |  |  |  | + |  |  | 2.A.66.1.32 | -86 | Q9KAX3 | Drugs | Rhodamine |
| [WP_004295740.1](file:///Volumes/ESD-USB/Summer%202018/Hassan%20Submission%20PLosOne/content.html#lcl|NZ_CP012938.1_prot_WP_004295740.1_910) |  |  |  |  |  | + |  | [2.A.66.1.32](http://tcdb.org/search/result.php?tc=2.A.66.1.32) | 6.88E-84 | Q9KAX3 | unknown | Unknown |
| [WP_004299380.1](file:///Volumes/ESD-USB/Summer%202018/Hassan%20Submission%20PLosOne/content.html#lcl|NZ_CP012938.1_prot_WP_004299380.1_2090) |  |  |  |  |  | + |  | [2.A.66.1.32](http://tcdb.org/search/result.php?tc=2.A.66.1.32) | -82 | Q9KAX3 | unknown | unknown |
| [WP_004299139.1](file:///Volumes/ESD-USB/Summer%202018/Hassan%20Submission%20PLosOne/content.html#lcl|NZ_CP012938.1_prot_WP_004299139.1_2264) |  |  |  |  |  | + |  | [2.A.66.1.32](http://tcdb.org/search/result.php?tc=2.A.66.1.32) | -62 | Q9KAX3 | unknown | unknown |
| [WP_005845631.1](file:///Volumes/ESD-USB/Summer%202018/Library/Application%20Support/Microsoft/Office/Office%202011%20AutoRecovery/content.html#lcl|NC_009614.1_prot_WP_005845631.1_268) |  |  |  |  |  |  | + | [2.A.66.1.32](http://tcdb.org/search/result.php?tc=2.A.66.1.32) | -84 | Q9KAX3 | unknown | unknown |
| [WP_005843272.1](file:///Volumes/ESD-USB/Summer%202018/Library/Application%20Support/Microsoft/Office/Office%202011%20AutoRecovery/content.html#lcl|NC_009614.1_prot_WP_005843272.1_2692) |  |  |  |  |  |  | + | [2.A.66.1.32](http://tcdb.org/search/result.php?tc=2.A.66.1.32) | -84 | Q9KAX3 | unknown | unknown |
| ABI39_18710 | **+** |  |  |  |  |  |  | 2.A.66.1.33 | -84 | I6L8P4 | Drugs | Fluoroquinolones |
| ABI39_12150 | **+** |  |  |  |  |  |  | 2.A.66.1.33 | -60 | I6L8P4 | Drugs | Fluoroquinolones |
| [WP_013618703.1](file:///Volumes/ESD-USB/Summer%202018/Hassan%20Submission%20PLosOne/content.html#lcl|NC_015164.1_prot_WP_013618703.1_2742) |  | + |  |  |  |  |  | [2.A.66.1.33](http://tcdb.org/search/result.php?tc=2.A.66.1.33) | -81 | I6L8P4 | Drugs | Fluoroquinolones/Acriflavin/Aminoglycosides |
| [WP_007213011.1](file:///Volumes/ESD-USB/Summer%202018/Hassan%20Submission%20PLosOne/content.html#lcl|NZ_CP012801.1_prot_WP_007213011.1_4298) |  |  | + |  |  |  |  | [2.A.66.1.33](http://tcdb.org/search/result.php?tc=2.A.66.1.33) | -82 | I6L8P4 | Drugs | Fluruquinolones |
| WP_029427670.1 |  |  | + |  |  |  |  | [2.A.66.1.33](http://tcdb.org/search/result.php?tc=2.A.66.1.33) | -44 | I6L8P4 | Drugs | Fluruquinolones |
| WP_062695515.1 |  |  |  | + |  |  |  | 2.A.66.1.33 | -83 | I6L8P4 | Drugs | Fluoroquinolones |
| WP_062694849.1 |  |  |  | + |  |  |  | 2.A.66.1.33 | -45 | I6L8P4 | Drugs | Fluoroquinolones |
| WP_004301982.1 |  |  |  |  |  | + |  | [2.A.66.1.33](http://tcdb.org/search/result.php?tc=2.A.66.1.33) | -84 | I6L8P4 | Drugs | Fluoroquinolones |
| [WP_004297055.1](file:///Volumes/ESD-USB/Summer%202018/Hassan%20Submission%20PLosOne/content.html#lcl|NZ_CP012938.1_prot_WP_004297055.1_1362) |  |  |  |  |  | + |  | [2.A.66.1.33](http://tcdb.org/search/result.php?tc=2.A.66.1.33) | -43 | I6L8P4 | Drugs | Fluoroquinolones |
| [WP_012055898.1](file:///Volumes/ESD-USB/Summer%202018/Library/Application%20Support/Microsoft/Office/Office%202011%20AutoRecovery/content.html#lcl|NC_009614.1_prot_WP_012055898.1_3692) |  |  |  |  |  |  | + | [2.A.66.1.33](http://tcdb.org/search/result.php?tc=2.A.66.1.33) | -83 | I6L8P4 | Drugs | Fluoroquinolones |
| [WP_011965597.1](file:///Volumes/ESD-USB/Summer%202018/Library/Application%20Support/Microsoft/Office/Office%202011%20AutoRecovery/content.html#lcl|NC_009614.1_prot_WP_011965597.1_2376) |  |  |  |  |  |  | + | [2.A.66.1.33](http://tcdb.org/search/result.php?tc=2.A.66.1.33) | -59 | I6L8P4 | Drugs | Fluoroquinolones |
| ABI39_09475 | **+** |  |  |  |  |  |  | 2.A.66.1.36 | -74 | Q8XT32 | Drugs | Antibiotics/Detergents |
| [WP_029427294.1](file:///Volumes/ESD-USB/Summer%202018/Hassan%20Submission%20PLosOne/content.html#lcl|NZ_CP012801.1_prot_WP_029427294.1_3665) |  |  | + |  |  |  |  | [2.A.66.1.36](http://tcdb.org/search/result.php?tc=2.A.66.1.36) | -76 | Q8XT32 | Drugs | Antibiotics/detergents/Phytoalexins |
| WP_011108187.1 |  |  |  | + |  |  |  | 2.A.66.1.36 | -82 | Q8XT32 | Drugs | antibiotics/detergents |
| YP_097979.1 |  |  |  |  | + |  |  | 2.A.66.1.36 | -74 | Q8XT32 | Drugs | Antibiotics/Detergents |
| WP_004299208.1 |  |  |  |  |  | + |  | [2.A.66.1.36](http://tcdb.org/search/result.php?tc=2.A.66.1.36) | -82 | Q8XT32 | Drugs | Fluoroquinolones |
| [WP_005839861.1](file:///Volumes/ESD-USB/Summer%202018/Library/Application%20Support/Microsoft/Office/Office%202011%20AutoRecovery/content.html#lcl|NC_009614.1_prot_WP_005839861.1_1849) |  |  |  |  |  |  | + | [2.A.66.1.36](http://tcdb.org/search/result.php?tc=2.A.66.1.36) | -75 | Q8XT32 | Drugs | Antibiotics/Detergents |
| ABI39_01880 | **+** |  |  |  |  |  |  | 2.A.66.1.37 | -66 | Q7WZ38 | Drugs | Ethidium bromide, fluoroquinolones and Acriflavin |
| [WP_013617940.1](file:///Volumes/ESD-USB/Summer%202018/Hassan%20Submission%20PLosOne/content.html#lcl|NC_015164.1_prot_WP_013617940.1_1916) |  | + |  |  |  |  |  | [2.A.66.1.37](http://tcdb.org/search/result.php?tc=2.A.66.1.37) | -69 | Q7WZ38 | Drugs | Fluruquinolones/ acriflavin |
| [WP_013617935.1](file:///Volumes/ESD-USB/Summer%202018/Hassan%20Submission%20PLosOne/content.html#lcl|NC_015164.1_prot_WP_013617935.1_1911) |  | + |  |  |  |  |  | [2.A.66.1.37](http://tcdb.org/search/result.php?tc=2.A.66.1.37) | -49 | Q7WZ38 | Drugs | Fluruquinolones/ acriflavin |
| [WP_029427691.1](file:///Volumes/ESD-USB/Summer%202018/Hassan%20Submission%20PLosOne/content.html#lcl|NZ_CP012801.1_prot_WP_029427691.1_4454) |  |  | + |  |  |  |  | [2.A.66.1.37](http://tcdb.org/search/result.php?tc=2.A.66.1.37) | -63 | Q7WZ38 | Drugs | Fluruquinolones/ acriflavin |
| [WP_029426693.1](file:///Volumes/ESD-USB/Summer%202018/Hassan%20Submission%20PLosOne/content.html#lcl|NZ_CP012801.1_prot_WP_029426693.1_3317) |  |  | + |  |  |  |  | [2.A.66.1.37](http://tcdb.org/search/result.php?tc=2.A.66.1.37) | -49 | Q7WZ38 | Drugs | Fluruquinolones/ acriflavin |
| WP_008766587.1 |  |  |  | + |  |  |  | 2.A.66.1.37 | -69 | Q7WZ38 | Drugs | Fluruquinolones/acriflavin |
| YP_101080.1_3797 |  |  |  |  | + |  |  | 2.A.66.1.37 | -70 | Q7WZ38 | Drugs | Fluoroquinolones/Aminoglycosides |
| [WP_004299405.1](file:///Volumes/ESD-USB/Summer%202018/Hassan%20Submission%20PLosOne/content.html#lcl|NZ_CP012938.1_prot_WP_004299405.1_2067) |  |  |  |  |  | + |  | [2.A.66.1.37](http://tcdb.org/search/result.php?tc=2.A.66.1.37) | -74 | Q7WZ38 | Drugs | Fluoroquinolones |
| [WP_008782062.1](file:///Volumes/ESD-USB/Summer%202018/Library/Application%20Support/Microsoft/Office/Office%202011%20AutoRecovery/content.html#lcl|NC_009614.1_prot_WP_008782062.1_342) |  |  |  |  |  |  | + | [2.A.66.1.37](http://tcdb.org/search/result.php?tc=2.A.66.1.37) | -65 | Q7WZ38 | Drugs | Ethidium bromide, fluoroquinolones and acriflavin |
| ABI39_10850 | **+** |  |  |  |  |  |  | 2.A.66.1.45 | -30 | Q8TZX5 | Unknown | Unknown |
| ABI39_19295 | **+** |  |  |  |  |  |  | 2.A.66.1.45 | -27 | Q8TZX5 | Unknown | Unknown |
| ABI39_03365 | **+** |  |  |  |  |  |  | 2.A.66.1.45 | -13 | Q8TZX5 | Unknown | Unknown |
| [WP_013616115.1](file:///Volumes/ESD-USB/Summer%202018/Hassan%20Submission%20PLosOne/content.html#lcl|NC_015164.1_prot_WP_013616115.1_38) |  | + |  |  |  |  |  | [2.A.66.1.45](http://tcdb.org/search/result.php?tc=2.A.66.1.45) | -33 | Q8TZX5 | Unknown | Unknown |
| [WP_029428287.1](file:///Volumes/ESD-USB/Summer%202018/Hassan%20Submission%20PLosOne/content.html#lcl|NZ_CP012801.1_prot_WP_029428287.1_1298) |  |  | + |  |  |  |  | [2.A.66.1.45](http://tcdb.org/search/result.php?tc=2.A.66.1.45) | -27 | Q8TZX5 | Unknown | Unknown |
| WP_062694928.1 |  |  |  | + |  |  |  | 2.A.66.1.45 | -33 | Q8TZX5 | Unknown | Unknown |
| WP_008767919.1 |  |  |  | + |  |  |  | 2.A.66.1.45 | -30 | Q8TZX5 | Unknown | Unknown |
| WP_062694639.1 |  |  |  | + |  |  |  | 2.A.66.1.45 | -16 | Q8TZX5 | Unknown | Unknown |
| YP_100495.1 |  |  |  |  | + |  |  | 2.A.66.1.45 | -31 | Q8TZX5 | Unknown | Unknown |
| YP_099888.1 |  |  |  |  | + |  |  | 2.A.66.1.45 | -28 | Q8TZX5 | Unknown | Unknown |
| YP_099361.1 |  |  |  |  | + |  |  | 2.A.66.1.45 | -27 | Q8TZX5 | Unknown | Unknown |
| [WP_004300795.1](file:///Volumes/ESD-USB/Summer%202018/Hassan%20Submission%20PLosOne/content.html#lcl|NZ_CP012938.1_prot_WP_004300795.1_1612) |  |  |  |  |  | + |  | [2.A.66.1.45](http://tcdb.org/search/result.php?tc=2.A.66.1.45) | -31 | Q8TZX5 | Unknown | Unknown |
| [WP_004296185.1](file:///Volumes/ESD-USB/Summer%202018/Hassan%20Submission%20PLosOne/content.html#lcl|NZ_CP012938.1_prot_WP_004296185.1_2510) |  |  |  |  |  | + |  | [2.A.66.1.45](http://tcdb.org/search/result.php?tc=2.A.66.1.45) | -14 | Q8TZX5 | Unknown | Unknown |
| [WP_005839098.1](file:///Volumes/ESD-USB/Summer%202018/Library/Application%20Support/Microsoft/Office/Office%202011%20AutoRecovery/content.html#lcl|NC_009614.1_prot_WP_005839098.1_2167) |  |  |  |  |  |  | + | [2.A.66.1.45](http://tcdb.org/search/result.php?tc=2.A.66.1.45) | -31 | Q8TZX5 | Unknown | Unknown |
| [WP_013617731.1](file:///Volumes/ESD-USB/Summer%202018/Hassan%20Submission%20PLosOne/content.html#lcl|NC_015164.1_prot_WP_013617731.1_1702) |  | + |  |  |  |  |  | [2.A.66.2.6](http://tcdb.org/search/result.php?tc=2.A.66.2.6) | -48 | O32273 | Sugar derivatives | Teichuronic acid |
| [WP_029428870.1](file:///Volumes/ESD-USB/Summer%202018/Hassan%20Submission%20PLosOne/content.html#lcl|NZ_CP012801.1_prot_WP_029428870.1_522) |  |  | + |  |  |  |  | [2.A.66.2.6](http://tcdb.org/search/result.php?tc=2.A.66.2.6) | -44 | O32273 | Sugar derivatives | Sugars |
| [WP_029428784.1](file:///Volumes/ESD-USB/Summer%202018/Hassan%20Submission%20PLosOne/content.html#lcl|NZ_CP012801.1_prot_WP_029428784.1_4746) |  |  | + |  |  |  |  | [2.A.66.2.6](http://tcdb.org/search/result.php?tc=2.A.66.2.6) | -43 | O32273 | Sugar derivatives | Sugars |
| [WP_029428945.1](file:///Volumes/ESD-USB/Summer%202018/Hassan%20Submission%20PLosOne/content.html#lcl|NZ_CP012801.1_prot_WP_029428945.1_666) |  |  | + |  |  |  |  | [2.A.66.2.6](http://tcdb.org/search/result.php?tc=2.A.66.2.6) | -36 | O32273 | Sugar derivatives | Sugars |
| YP_099866.1 |  |  |  |  | + |  |  | 2.A.66.2.6 | -45 | O32273 | Sugars | Teichuronic acid |
| YP_098093.1 |  |  |  |  | + |  |  | 2.A.66.2.6 | -44 | O32273 | Sugars | Teichuronic acid |
| YP_099283.1_2000 |  |  |  |  | + |  |  | 2.A.66.2.6 | -38 | O32273 | Sugars | Teichuronic acid |
| YP_101775.1_4492 |  |  |  |  | + |  |  | 2.A.66.2.6 | -37 | O32273 | Sugars | Teichuronic acid |
| YP_101199.1_3916 |  |  |  |  | + |  |  | 2.A.66.2.6 | -36 | O32273 | Sugars | Teichuronic acid |
| [WP_004300364.1](file:///Volumes/ESD-USB/Summer%202018/Hassan%20Submission%20PLosOne/content.html#lcl|NZ_CP012938.1_prot_WP_004300364.1_467) |  |  |  |  |  | + |  | [2.A.66.2.6](http://tcdb.org/search/result.php?tc=2.A.66.2.6) | -36 | O32273 | Sugar derivatives | Teichuronic acid |
| ABI39_06960 | **+** |  |  |  |  |  |  | 2.A.66.2.7 | -34 | P77377 | Sugars derivatives | Colanic acid |
| [WP_007211698.1](file:///Volumes/ESD-USB/Summer%202018/Hassan%20Submission%20PLosOne/content.html#lcl|NZ_CP012801.1_prot_WP_007211698.1_2951) |  |  | + |  |  |  |  | [2.A.66.2.7](http://tcdb.org/search/result.php?tc=2.A.66.2.7) | -37 | P77377 | Sugar derivatives | Colanic acid |
| WP_062694203.1 |  |  |  | + |  |  |  | 2.A.66.2.7 | -31 | P77377 | Sugar derivatives | Colanic acid |
| [WP_004296590.1](file:///Volumes/ESD-USB/Summer%202018/Hassan%20Submission%20PLosOne/content.html#lcl|NZ_CP012938.1_prot_WP_004296590.1_2865) |  |  |  |  |  | + |  | [2.A.66.2.7](http://tcdb.org/search/result.php?tc=2.A.66.2.7) | -39 | P77377 | Unknown | Unknown |
| WP_005850555.1 |  |  |  |  |  |  | + | [2.A.66.2.7](http://tcdb.org/search/result.php?tc=2.A.66.2.7) | -33 | P77377 | Sugar derivatives | Colanic acid |
| [WP_004299959.1](file:///Volumes/ESD-USB/Summer%202018/Hassan%20Submission%20PLosOne/content.html#lcl|NZ_CP012938.1_prot_WP_004299959.1_1076) |  |  |  |  |  | + |  | [2.A.66.2.8](http://tcdb.org/search/result.php?tc=2.A.66.2.8) | -49 | D4HW93 | Sugars | Polysaccharides |
| [WP_029428697.1](file:///Volumes/ESD-USB/Summer%202018/Hassan%20Submission%20PLosOne/content.html#lcl|NZ_CP012801.1_prot_WP_029428697.1_4174) |  |  | + |  |  |  |  | [2.A.66.2.15](http://tcdb.org/search/result.php?tc=2.A.66.2.15) | -41 | G3XD19 | Unknown | Unknown |
| ABI39_20325 | **+** |  |  |  |  |  |  | 2.A.66.2.16 | -66 | Q8KUK6 | Unknown | Unknown |
| ABI39_20810 | + |  |  |  |  |  |  | 2.A.66.2.16 |  | Q8KUK6 | Unknown | Unknown |
| [WP_013617226.1](file:///Volumes/ESD-USB/Summer%202018/Hassan%20Submission%20PLosOne/content.html#lcl|NC_015164.1_prot_WP_013617226.1_1194) |  | + |  |  |  |  |  | [2.A.66.2.16](http://tcdb.org/search/result.php?tc=2.A.66.2.16) | -67 | Q8KUK6 | Sugars | Polysaccharides |
| [WP_013618099.1](file:///Volumes/ESD-USB/Summer%202018/Hassan%20Submission%20PLosOne/content.html#lcl|NC_015164.1_prot_WP_013618099.1_2076) |  | + |  |  |  |  |  | [2.A.66.2.20](http://tcdb.org/search/result.php?tc=2.A.66.2.20) | -06 | F8KUW5 | Sugars | Polysaccharides |
| [WP_013616147.1](file:///Volumes/ESD-USB/Summer%202018/Hassan%20Submission%20PLosOne/content.html#lcl|NC_015164.1_prot_WP_013616147.1_71) |  | + |  |  |  |  |  | [2.A.66.2.20](http://tcdb.org/search/result.php?tc=2.A.66.2.20) | -05 | F8KUW5 | Sugars | Polysaccharides |
| [WP_007218034.1](file:///Volumes/ESD-USB/Summer%202018/Hassan%20Submission%20PLosOne/content.html#lcl|NZ_CP012801.1_prot_WP_007218034.1_473) |  |  | + |  |  |  |  | [2.A.66.2.20](http://tcdb.org/search/result.php?tc=2.A.66.2.20) | -10 | F8KUW5 | Unknown | Unknown |
| [WP_022209873.1](file:///Volumes/ESD-USB/Summer%202018/Hassan%20Submission%20PLosOne/content.html#lcl|NZ_CP012801.1_prot_WP_022209873.1_2487) |  |  | + |  |  |  |  | [2.A.66.2.20](http://tcdb.org/search/result.php?tc=2.A.66.2.20) | -05 | F8KUW5 | Unknown | Unknown |
| WP_011108742.1 |  |  |  | + |  |  |  | 2.A.66.2.20 | -10 | F8KUW5 | Unknown | Unknown |
| YP_097447.1 |  |  |  |  | + |  |  | 2.A.66.2.20 | -11 | F8KUW5 | Unknown | Unknown |
| [WP_004298043.1](file:///Volumes/ESD-USB/Summer%202018/Hassan%20Submission%20PLosOne/content.html#lcl|NZ_CP012938.1_prot_WP_004298043.1_3224) |  |  |  |  |  | + |  | [2.A.66.2.20](http://tcdb.org/search/result.php?tc=2.A.66.2.20) | -09 | F8KUW5 | Unknown | Unknown |
| [WP_005838743.1](file:///Volumes/ESD-USB/Summer%202018/Library/Application%20Support/Microsoft/Office/Office%202011%20AutoRecovery/content.html#lcl|NC_009614.1_prot_WP_005838743.1_1272) |  |  |  |  |  |  | + | [2.A.66.2.20](http://tcdb.org/search/result.php?tc=2.A.66.2.20) | -08 | F8KUW5 | Unknown | Unknown |
| ABI39_17575 | **+** |  |  |  |  |  |  | 2.A.66.10.1 | -52 | F1XYT9 | Unknown | Unknown |
| [WP_005636855.1](file:///Volumes/ESD-USB/Summer%202018/Library/Application%20Support/Microsoft/Office/Office%202011%20AutoRecovery/content.html#lcl|NC_009614.1_prot_WP_005636855.1_1524) |  |  |  |  |  |  | + | [2.A.66.10.1](http://tcdb.org/search/result.php?tc=2.A.66.10.1) | -24 | F1XYT9 | Unknown | Unknown |
| [WP_005636855.1](file:///Volumes/ESD-USB/Summer%202018/Library/Application%20Support/Microsoft/Office/Office%202011%20AutoRecovery/content.html#lcl|NC_009614.1_prot_WP_005636855.1_692) |  |  |  |  |  |  | + | [2.A.66.10.1](http://tcdb.org/search/result.php?tc=2.A.66.10.1) | -24 | F1XYT9 | Unknown | Unknown |
| [WP_012055955.1](file:///Volumes/ESD-USB/Summer%202018/Library/Application%20Support/Microsoft/Office/Office%202011%20AutoRecovery/content.html#lcl|NC_009614.1_prot_WP_012055955.1_3833) |  |  |  |  |  |  | + | [2.A.66.10.1](http://tcdb.org/search/result.php?tc=2.A.66.10.1) | -19 | F1XYT9 | Unknown | Unknown |
| [WP_011965287.1](file:///Volumes/ESD-USB/Summer%202018/Library/Application%20Support/Microsoft/Office/Office%202011%20AutoRecovery/content.html#lcl|NC_009614.1_prot_WP_011965287.1_1703) |  |  |  |  |  |  | + | [2.A.66.10.1](http://tcdb.org/search/result.php?tc=2.A.66.10.1) | -16 | F1XYT9 | Unknown | Unknown |
| WP_055300237.1 |  |  |  | + |  |  |  | 2.A.66.12.2 | -77 | B7B640 | Unknown | Unknown |
| [WP_007213898.1](file:///Volumes/ESD-USB/Summer%202018/Hassan%20Submission%20PLosOne/content.html#lcl|NZ_CP012801.1_prot_WP_007213898.1_365) |  |  | + |  |  |  |  | [2.A.67.4.2](http://tcdb.org/search/result.php?tc=2.A.67.4.2) | -76 | O58099 | Unknown | Unknown |
| WP_062695121.1 |  |  |  | + |  |  |  | 2.A.67.4.2 | -77 | O58099 | Unknown | Unknown |
| [WP_004300419.1](file:///Volumes/ESD-USB/Summer%202018/Hassan%20Submission%20PLosOne/content.html#lcl|NZ_CP012938.1_prot_WP_004300419.1_515) |  |  |  |  |  | + |  | [2.A.67.4.2](http://tcdb.org/search/result.php?tc=2.A.67.4.2) | -77 | O58099 | unknown | Unknown |
| [WP_007214039.1](file:///Volumes/ESD-USB/Summer%202018/Hassan%20Submission%20PLosOne/content.html#lcl|NZ_CP012801.1_prot_WP_007214039.1_45) |  |  | + |  |  |  |  | [2.A.68.1.2](http://tcdb.org/search/result.php?tc=2.A.68.1.2) | -96 | Q5F721 | Drugs | Sulfonamides |
| WP_011107428.1 |  |  |  | + |  |  |  | 2.A.68.1.2 | -85 | Q5F721 | Drugs | Drugs |
| [WP_013619465.1](file:///Volumes/ESD-USB/Summer%202018/Hassan%20Submission%20PLosOne/content.html#lcl|NC_015164.1_prot_WP_013619465.1_3511) |  | + |  |  |  |  |  | [2.A.69.4.5](http://tcdb.org/search/result.php?tc=2.A.69.4.5) | -15 | Q48797 | Carboxylates | Malate |
| ABI39_00400 | + |  |  |  |  |  |  | 2.A.76.1.6 | -18 | Q3J2V9 | Amino Acids | Amino Acids |
| [WP_013616816.1](file:///Volumes/ESD-USB/Summer%202018/Hassan%20Submission%20PLosOne/content.html#lcl|NC_015164.1_prot_WP_013616816.1_758) |  | + |  |  |  |  |  | [2.A.76.1.6](http://tcdb.org/search/result.php?tc=2.A.76.1.6) | -07 | Q3J2V9 | Amino Acids | Amino Acids |
| [WP_007214810.1](file:///Volumes/ESD-USB/Summer%202018/Hassan%20Submission%20PLosOne/content.html#lcl|NZ_CP012801.1_prot_WP_007214810.1_1246) |  |  | + |  |  |  |  | [2.A.76.1.6](http://tcdb.org/search/result.php?tc=2.A.76.1.6) | -07 | Q3J2V9 | Amino Acids |  |
| WP_008767802.1 |  |  |  | + |  |  |  | 2.A.76.1.6 | -07 | Q3J2V9 | Unknown | Unknown |
| [WP_008774798.1](file:///Volumes/ESD-USB/Summer%202018/Hassan%20Submission%20PLosOne/content.html#lcl|NZ_CP012938.1_prot_WP_008774798.1_1714) |  |  |  |  |  | + |  | [2.A.76.1.6](http://tcdb.org/search/result.php?tc=2.A.76.1.6) | -07 | Q3J2V9 | Amino acids | Amino acids |
| [WP_005842995.1](file:///Volumes/ESD-USB/Summer%202018/Library/Application%20Support/Microsoft/Office/Office%202011%20AutoRecovery/content.html#lcl|NC_009614.1_prot_WP_005842995.1_79) |  |  |  |  |  |  | + | [2.A.76.1.6](http://tcdb.org/search/result.php?tc=2.A.76.1.6) | -07 | Q3J2V9 | Amino Acids | Amino Acids |
| [WP_029426820.1](file:///Volumes/ESD-USB/Summer%202018/Hassan%20Submission%20PLosOne/content.html#lcl|NZ_CP012801.1_prot_WP_029426820.1_362) |  |  | + |  |  |  |  | [2.A.78.1.1](http://tcdb.org/search/result.php?tc=2.A.78.1.1) | -74 | O07942 | Amino acids | Amino Acids |
| YP_099982.1 |  |  |  |  | + |  |  | 2.A.79.1.7 | -07 | W4C0M8 | Unknown | Unknown |
| [WP_007213453.1](file:///Volumes/ESD-USB/Summer%202018/Hassan%20Submission%20PLosOne/content.html#lcl|NZ_CP012801.1_prot_WP_007213453.1_4974) |  |  | + |  |  |  |  | [2.A.79.2.1](http://tcdb.org/search/result.php?tc=2.A.79.2.1) | -21 | Q8FA71 | Unknown | Unknown |
| WP_008761612.1 |  |  |  | + |  |  |  | 2.A.79.2.1 | -24 | Q8FA71 | Unknown | Unknown |
| ABI39_01645 | + |  |  |  |  |  |  | 2.A.81.1.2 | -07 | Q5LCC7 | Cations | Co^+2^ |
| ABI39_15590 | + |  |  |  |  |  |  | 2.A.81.1.2 | 0 | Q5LCC7 | Cations | Co^+2^ |
| ABI39_15645 | + |  |  |  |  |  |  | 2.A.81.1.2 | 0 | Q5LCC7 | Cations | Co^+2^ |
| ABI39_14555 | + |  |  |  |  |  |  | 2.A.81.1.2 | 0 | Q5LCC7 | Cations | Co^+2^ |
| ABI39_13490 | + |  |  |  |  |  |  | 2.A.81.1.2 | -163 | Q5LCC7 | Cations | Co^+2^ |
| [WP_013616609.1](file:///Volumes/ESD-USB/Summer%202018/Hassan%20Submission%20PLosOne/content.html#lcl|NC_015164.1_prot_WP_013616609.1_544) |  | + |  |  |  |  |  | [2.A.81.1.2](http://tcdb.org/search/result.php?tc=2.A.81.1.2) |  | Q5LCC7 | Cations | Co^+2^ |
| [WP_013616736.1](file:///Volumes/ESD-USB/Summer%202018/Hassan%20Submission%20PLosOne/content.html#lcl|NC_015164.1_prot_WP_013616736.1_674) |  | + |  |  |  |  |  | [2.A.81.1.2](http://tcdb.org/search/result.php?tc=2.A.81.1.2) | -161 | Q5LCC7 | Cations | Co^+2^ |
| [WP_007210356.1](file:///Volumes/ESD-USB/Summer%202018/Hassan%20Submission%20PLosOne/content.html#lcl|NZ_CP012801.1_prot_WP_007210356.1_1487) |  |  | + |  |  |  |  | [2.A.81.1.2](http://tcdb.org/search/result.php?tc=2.A.81.1.2) | 0 | Q5LCC7 | Anions | Co^+2^ |
| [WP_022209060.1](file:///Volumes/ESD-USB/Summer%202018/Hassan%20Submission%20PLosOne/content.html#lcl|NZ_CP012801.1_prot_WP_022209060.1_4733) |  |  | + |  |  |  |  | [2.A.81.1.2](http://tcdb.org/search/result.php?tc=2.A.81.1.2) | 0 | Q5LCC7 | Anions | Co^+2^ |
| [WP_007213533.1](file:///Volumes/ESD-USB/Summer%202018/Hassan%20Submission%20PLosOne/content.html#lcl|NZ_CP012801.1_prot_WP_007213533.1_646) |  |  | + |  |  |  |  | [2.A.81.1.2](http://tcdb.org/search/result.php?tc=2.A.81.1.2) | -166 | Q5LCC7 | Anions | Co^+2^ |
| [WP_007219608.1](file:///Volumes/ESD-USB/Summer%202018/Hassan%20Submission%20PLosOne/content.html#lcl|NZ_CP012801.1_prot_WP_007219608.1_3708) |  |  | + |  |  |  |  | [2.A.81.1.2](http://tcdb.org/search/result.php?tc=2.A.81.1.2) | -100 | Q5LCC7 | Anions | Co^+2^ |
| WP_008766579.1 |  |  |  | + |  |  |  | 2.A.81.1.2 | 0 | Q5LCC7 | Cations | Co^+2^, K^+^ |
| WP_008763411.1 |  |  |  | + |  |  |  | 2.A.81.1.2 | -172 | Q5LCC7 | Cations | Co^+2^, K^+^ |
| WP_008765024.1 |  |  |  | + |  |  |  | 2.A.81.1.2 | -99 | Q5LCC7 | Cations | Co^+2^, K^+^ |
| YP_101078.1 |  |  |  |  | + |  |  | 2.A.81.1.2 | 0 | Q5LCC7 | Cations | Co^+2^ |
| YP_099790.1 |  |  |  |  | + |  |  | 2.A.81.1.2 | 0 | Q5LCC7 | Cations | Co^+2^ |
| YP_099099.1 |  |  |  |  | + |  |  | 2.A.81.1.2 | -171 | Q5LCC7 | Cations | Co^+2^ |
| YP_099964.1 |  |  |  |  | + |  |  | 2.A.81.1.2 | -101 | Q5LCC7 | Cations | Co^+2^ |
| [WP_004297845.1](file:///Volumes/ESD-USB/Summer%202018/Hassan%20Submission%20PLosOne/content.html#lcl|NZ_CP012938.1_prot_WP_004297845.1_337) |  |  |  |  |  | + |  | [2.A.81.1.2](http://tcdb.org/search/result.php?tc=2.A.81.1.2) | -172 | Q5LCC7 | Cations | Co^+2^ |
| [WP_004300141.1](file:///Volumes/ESD-USB/Summer%202018/Hassan%20Submission%20PLosOne/content.html#lcl|NZ_CP012938.1_prot_WP_004300141.1_1201) |  |  |  |  |  | + |  | [2.A.81.1.2](http://tcdb.org/search/result.php?tc=2.A.81.1.2) | -96 | Q5LCC7 | Cations | Co^+2^ |
| [WP_005845692.1](file:///Volumes/ESD-USB/Summer%202018/Library/Application%20Support/Microsoft/Office/Office%202011%20AutoRecovery/content.html#lcl|NC_009614.1_prot_WP_005845692.1_306) |  |  |  |  |  |  | + | [2.A.81.1.2](http://tcdb.org/search/result.php?tc=2.A.81.1.2) | 0 | Q5LCC7 | Cations | Co^+2^ |
| [WP_005846503.1](file:///Volumes/ESD-USB/Summer%202018/Library/Application%20Support/Microsoft/Office/Office%202011%20AutoRecovery/content.html#lcl|NC_009614.1_prot_WP_005846503.1_3044) |  |  |  |  |  |  | + | [2.A.81.1.2](http://tcdb.org/search/result.php?tc=2.A.81.1.2) | 0 | Q5LCC7 | Cations | Co^+2^ |
| [WP_005846477.1](file:///Volumes/ESD-USB/Summer%202018/Library/Application%20Support/Microsoft/Office/Office%202011%20AutoRecovery/content.html#lcl|NC_009614.1_prot_WP_005846477.1_3055) |  |  |  |  |  |  | + | [2.A.81.1.2](http://tcdb.org/search/result.php?tc=2.A.81.1.2) | 0 | Q5LCC7 | Cations | Co^+2^ |
| [WP_005847874.1](file:///Volumes/ESD-USB/Summer%202018/Library/Application%20Support/Microsoft/Office/Office%202011%20AutoRecovery/content.html#lcl|NC_009614.1_prot_WP_005847874.1_2848) |  |  |  |  |  |  | + | [2.A.81.1.2](http://tcdb.org/search/result.php?tc=2.A.81.1.2) | -163 | Q5LCC7 | Cations | Co^+2^ |
| [WP_005843762.1](file:///Volumes/ESD-USB/Summer%202018/Library/Application%20Support/Microsoft/Office/Office%202011%20AutoRecovery/content.html#lcl|NC_009614.1_prot_WP_005843762.1_2637) |  |  |  |  |  |  | + | [2.A.81.1.2](http://tcdb.org/search/result.php?tc=2.A.81.1.2) | -95 | Q5LCC7 | Cations | Co^+2^ |
| [WP_022210688.1](file:///Volumes/ESD-USB/Summer%202018/Hassan%20Submission%20PLosOne/content.html#lcl|NZ_CP012801.1_prot_WP_022210688.1_1009) |  |  | + |  |  |  |  | [2.A.81.1.4](http://tcdb.org/search/result.php?tc=2.A.81.1.4) | -83 | A7ZJR5 | Unknown | Unknown |
| WP_011107422.1 |  |  |  | + |  |  |  | 2.A.81.1.4 | -80 | A7ZJR5 | Unknown | Unknown |
| YP_098803.1 |  |  |  |  | + |  |  | 2.A.81.1.4 | -76 | A7ZJR5 | Cations | Cations |
| [WP_004295784.1](file:///Volumes/ESD-USB/Summer%202018/Hassan%20Submission%20PLosOne/content.html#lcl|NZ_CP012938.1_prot_WP_004295784.1_874) |  |  |  |  |  | + |  | [2.A.81.1.4](http://tcdb.org/search/result.php?tc=2.A.81.1.4) | -79 | A7ZJR5 | Unknown | Unknown |
| [WP_011965545.1](file:///Volumes/ESD-USB/Summer%202018/Library/Application%20Support/Microsoft/Office/Office%202011%20AutoRecovery/content.html#lcl|NC_009614.1_prot_WP_011965545.1_2217) |  |  |  |  |  |  | + | [2.A.81.1.4](http://tcdb.org/search/result.php?tc=2.A.81.1.4) | -70 | A7ZJR5 | Unknown | Unknown |
| WP_062694524.1 |  |  |  | + |  |  |  | 2.A.86.1.11 | -21 | F8FLY5 | Unknown | Unknown |
| ABI39_10520 | + |  |  |  |  |  |  | 2.A.86.2.3 | -95 | Q3IS50 | Unknown | Unknown |
| [WP_026367749.1](file:///Volumes/ESD-USB/Summer%202018/Hassan%20Submission%20PLosOne/content.html#lcl|NZ_CP012801.1_prot_WP_026367749.1_3904) |  |  | + |  |  |  |  | [2.A.86.2.3](http://tcdb.org/search/result.php?tc=2.A.86.2.3) | -17 | Q3IS50 | Unknown | Unknown |
| WP_011107805.1 |  |  |  | + |  |  |  | 2.A.86.2.3 | -21 | Q3IS50 | Unknown | Unknown |
| [WP_013617427.1](file:///Volumes/ESD-USB/Summer%202018/Hassan%20Submission%20PLosOne/content.html#lcl|NC_015164.1_prot_WP_013617427.1_1393) |  | + |  |  |  |  |  | [2.A.88.8.1](http://tcdb.org/search/result.php?tc=2.A.88.8.1) | -17 | D3QD12 | Nucleoside | Queuosine |
| WP_008762200.1 |  |  |  | + |  |  |  | 2.A.88.8.4 | -14 | Q8PW04 | Unknown | Unknown |
| YP_098749.1 |  |  |  |  | + |  |  | 2.A.88.8.5 | -16 | B2FPS5 | Unknown | Unknown |
| [WP_007213368.1](file:///Volumes/ESD-USB/Summer%202018/Hassan%20Submission%20PLosOne/content.html#lcl|NZ_CP012801.1_prot_WP_007213368.1_4601) |  |  | + |  |  |  |  | [2.A.88.8.5](http://tcdb.org/search/result.php?tc=2.A.88.8.5) | -17 | B2FPS5 | Vitamins | Vitamins |
| [WP_004300766.1](file:///Volumes/ESD-USB/Summer%202018/Hassan%20Submission%20PLosOne/content.html#lcl|NZ_CP012938.1_prot_WP_004300766.1_1586) |  |  |  |  |  | + |  | [2.A.88.8.5](http://tcdb.org/search/result.php?tc=2.A.88.8.5) | -14 | B2FPS5 | Vitamins | Vitamins |
| [WP_029429062.1](file:///Volumes/ESD-USB/Summer%202018/Hassan%20Submission%20PLosOne/content.html#lcl|NZ_CP012801.1_prot_WP_029429062.1_4779) |  |  | + |  |  |  |  | [2.A.89.3.10](http://tcdb.org/search/result.php?tc=2.A.89.3.10) | -16 | Q9UY73 | Cations | Fe^2+^ |
| WP_062695954.1 |  |  |  | + |  |  |  | 2.A.89.3.10 | -19 | Q9UY73 | Unknown | Unknown |
| [WP_025726054.1](file:///Volumes/ESD-USB/Summer%202018/Hassan%20Submission%20PLosOne/content.html#lcl|NZ_CP012801.1_prot_WP_025726054.1_1465) |  |  | + |  |  |  |  | [2.A.90.3.3](http://tcdb.org/search/result.php?tc=2.A.90.3.3) | -06 | K1QQE6 | Cations | Fe^2+^ |
| ABI39_01190 | + |  |  |  |  |  |  | 2.A.95.1.3 | -18 | O32244 | Unknown | Unknown |
| [WP_007215438.1](file:///Volumes/ESD-USB/Summer%202018/Hassan%20Submission%20PLosOne/content.html#lcl|NZ_CP012801.1_prot_WP_007215438.1_4055) |  |  | + |  |  |  |  | [2.A.95.1.3](http://tcdb.org/search/result.php?tc=2.A.95.1.3) | -16 | O32244 | Unknown | Unknown |
| WP_004308119.1 |  |  |  | + |  |  |  | 2.A.95.1.3 | -43 | O32244 | Unknown | Unknown |
| WP_008759972.1 |  |  |  | + |  |  |  | 2.A.95.1.3 | -16 | O32244 | Unknown | Unknown |
| WP_008760156.1 |  |  |  | + |  |  |  | 2.A.95.1.3 | -13 | O32244 | Unknown | Unknown |
| YP_097946.1 |  |  |  |  | + |  |  | 2.A.95.1.3 | -42 | O32244 | Unknown | Unknown |
| YP_098280.1 |  |  |  |  | + |  |  | 2.A.95.1.3 | -16 | O32244 | Unknown | Unknown |
| YP_099469.1 |  |  |  |  | + |  |  | 2.A.95.1.3 | -15 | O32244 | Unknown | Unknown |
| [WP_004323496.1](file:///Volumes/ESD-USB/Summer%202018/Hassan%20Submission%20PLosOne/content.html#lcl|NZ_CP012938.1_prot_WP_004323496.1_2232) |  |  |  |  |  | + |  | [2.A.95.1.3](http://tcdb.org/search/result.php?tc=2.A.95.1.3) | -43 | O32244 | Unknown | Unknown |
| [WP_004301781.1](file:///Volumes/ESD-USB/Summer%202018/Hassan%20Submission%20PLosOne/content.html#lcl|NZ_CP012938.1_prot_WP_004301781.1_4357) |  |  |  |  |  | + |  | [2.A.95.1.3](http://tcdb.org/search/result.php?tc=2.A.95.1.3) | -17 | O32244 | Unknown | Unknown |
| [WP_004304823.1](file:///Volumes/ESD-USB/Summer%202018/Hassan%20Submission%20PLosOne/content.html#lcl|NZ_CP012938.1_prot_WP_004304823.1_4539) |  |  |  |  |  | + |  | [2.A.95.1.3](http://tcdb.org/search/result.php?tc=2.A.95.1.3) | -11 | O32244 | Unknown | Unknown |
| [WP_007212579.1](file:///Volumes/ESD-USB/Summer%202018/Hassan%20Submission%20PLosOne/content.html#lcl|NZ_CP012801.1_prot_WP_007212579.1_3546) |  |  | + |  |  |  |  | [2.A.95.1.4](http://tcdb.org/search/result.php?tc=2.A.95.1.4) | -42 | Q8J305 | Unknown | Unknown |
| WP_008763832.1 |  |  |  | + |  |  |  | 2.A.95.1.4 | -42 | Q8J305 | Unknown | Unknown |
| [WP_013616174.1](file:///Volumes/ESD-USB/Summer%202018/Hassan%20Submission%20PLosOne/content.html#lcl|NC_015164.1_prot_WP_013616174.1_98) |  | + |  |  |  |  |  | [2.A.95.1.5](http://tcdb.org/search/result.php?tc=2.A.95.1.5) | -17 | P67143 | Unknown | Unknown |
| ABI39_13165 | + |  |  |  |  |  |  | 2.A.95.1.6 | -15 | D3P9M4 | Amino Acids | Amino acid |
| [WP_005844726.1](file:///Volumes/ESD-USB/Summer%202018/Library/Application%20Support/Microsoft/Office/Office%202011%20AutoRecovery/content.html#lcl|NC_009614.1_prot_WP_005844726.1_2580) |  |  |  |  |  |  | + | [2.A.95.1.6](http://tcdb.org/search/result.php?tc=2.A.95.1.6) | -15 | D3P9M4 | Amino Acids | Serine |
| [WP_013616920.1](file:///Volumes/ESD-USB/Summer%202018/Hassan%20Submission%20PLosOne/content.html#lcl|NC_015164.1_prot_WP_013616920.1_864) |  | + |  |  |  |  |  | [2.A.98.1.3](http://tcdb.org/search/result.php?tc=2.A.98.1.3) | -73 | P62723 | Anions | SO_4_^2-^ |
| WP_008766269.1 |  |  |  | + |  |  |  | 2.A.98.1.3 | -80 | P62723 | Anions | SO_4_^2-^ |
| YP_098579.1_1296 |  |  |  |  | + |  |  | 2.A.98.1.3 | -78 | P62723 | Anions | SO_4_^2-^ |
| [WP_007213204.1](file:///Volumes/ESD-USB/Summer%202018/Hassan%20Submission%20PLosOne/content.html#lcl|NZ_CP012801.1_prot_WP_007213204.1_4443) |  |  | + |  |  |  |  | [2.A.98.1.4](http://tcdb.org/search/result.php?tc=2.A.98.1.4) | -40 | F3LB58 | Unknown | Unknown |
| WP_008764761.1 |  |  |  | + |  |  |  | 2.A.98.1.4 | -40 | F3LB58 | Unknown | Unknown |
| YP_098492.1 |  |  |  |  | + |  |  | 2.A.98.1.4 | -40 | F3LB58 | Anions | SO_4_^2-^ |
| [WP_013618017.1](file:///Volumes/ESD-USB/Summer%202018/Hassan%20Submission%20PLosOne/content.html#lcl|NC_015164.1_prot_WP_013618017.1_1993) |  | + |  |  |  |  |  | [2.A.103.1.1](http://tcdb.org/search/result.php?tc=2.A.103.1.1) | -29 | P0ABG4 | Unknown | Unknown |
| WP_008767643.1 |  |  |  | + |  |  |  | 2.A.103.1.1 | -32 | P0ABG4 | Unknown | Unknown |
| YP_097590.1 |  |  |  |  | + |  |  | 2.A.103.1.1 | -31 | P0ABG4 | Unknown | Unknown |
| ABI39_09215 | + |  |  |  |  |  |  | 2.A.103.1.2 | -34 | P0ABG7 | Unknown | Unknown |
| [WP_013617392.1](file:///Volumes/ESD-USB/Summer%202018/Hassan%20Submission%20PLosOne/content.html#lcl|NC_015164.1_prot_WP_013617392.1_1361) |  | + |  |  |  |  |  | [2.A.103.1.2](http://tcdb.org/search/result.php?tc=2.A.103.1.2) | -38 | P0ABG7 | Unknown | Unknown |
| YP_101372.1 |  |  |  |  | + |  |  | 2.A.103.1.2 | -34 | P0ABG7 | Unknown | Unknown |
| [WP_013617349.1](file:///Volumes/ESD-USB/Summer%202018/Hassan%20Submission%20PLosOne/content.html#lcl|NC_015164.1_prot_WP_013617349.1_1317) |  | + |  |  |  |  |  | [2.A.107.1.1](http://tcdb.org/search/result.php?tc=2.A.107.1.1) | -36 | P76264 | Unknown | Unknown |
| [WP_007213160.1](file:///Volumes/ESD-USB/Summer%202018/Hassan%20Submission%20PLosOne/content.html#lcl|NZ_CP012801.1_prot_WP_007213160.1_4414) |  |  | + |  |  |  |  | [2.A.107.1.1](http://tcdb.org/search/result.php?tc=2.A.107.1.1) | -32 | P76264 | Cations | Mn2+ |
| WP_008764711.1 |  |  |  | + |  |  |  | 2.A.107.1.1 | -34 | P76264 | Cations | Mn2+ |
| ABI39_00465 | + |  |  |  |  |  |  | 2.A.114.1.4 | -156 | Q8XME6 | Unknown | Unknown |
| [WP_013616921.1](file:///Volumes/ESD-USB/Summer%202018/Hassan%20Submission%20PLosOne/content.html#lcl|NC_015164.1_prot_WP_013616921.1_865) |  | + |  |  |  |  |  | [2.A.114.1.4](http://tcdb.org/search/result.php?tc=2.A.114.1.4) | -147 | Q8XME6 | Unknown | Unknown |
| [WP_007217389.1](file:///Volumes/ESD-USB/Summer%202018/Hassan%20Submission%20PLosOne/content.html#lcl|NZ_CP012801.1_prot_WP_007217389.1_1256) |  |  | + |  |  |  |  | [2.A.114.1.4](http://tcdb.org/search/result.php?tc=2.A.114.1.4) | -146 | Q8XME6 | Unknown | Unknown |
| WP_008763305.1 |  |  |  | + |  |  |  | 2.A.114.1.4 | -156 | Q8XME6 | Unknown | Unknown |
| YP_100601.1 |  |  |  |  | + |  |  | 2.A.114.1.4 | -153 | Q8XME6 | Unknown | Unknown |
| [WP_004300963.1](file:///Volumes/ESD-USB/Summer%202018/Hassan%20Submission%20PLosOne/content.html#lcl|NZ_CP012938.1_prot_WP_004300963.1_1723) |  |  |  |  |  | + |  | [2.A.114.1.4](http://tcdb.org/search/result.php?tc=2.A.114.1.4) | -156 | Q8XME6 | Peptides | Peptides |
| [WP_005851068.1](file:///Volumes/ESD-USB/Summer%202018/Library/Application%20Support/Microsoft/Office/Office%202011%20AutoRecovery/content.html#lcl|NC_009614.1_prot_WP_005851068.1_90) |  |  |  |  |  |  | + | [2.A.114.1.4](http://tcdb.org/search/result.php?tc=2.A.114.1.4) | -157 | Q8XME6 | Unknown | Unknown |
| [WP_007212615.1](file:///Volumes/ESD-USB/Summer%202018/Hassan%20Submission%20PLosOne/content.html#lcl|NZ_CP012801.1_prot_WP_007212615.1_3989) |  |  | + |  |  |  |  | [2.A.115.1.2](http://tcdb.org/search/result.php?tc=2.A.115.1.2) | -43 | P94357 | Drugs | Novobiocin |
| WP_011109173.1 |  |  |  | + |  |  |  | 2.A.115.1.2 | -43 | P94357 | Drugs | Aminocoumarin |
| WP_008762766.1 |  |  |  | + |  |  |  | 2.A.115.1.2 | -28 | P94357 | Drugs | Aminocoumarin |
| YP_098215.1_932 |  |  |  |  | + |  |  | 2.A.115.1.2 | -46 | P94357 | Drugs | Novobiocin |
| YP_101858.1 |  |  |  |  | + |  |  | 2.A.115.1.2 | -31 | P94357 | Drugs | Novobiocin |
| [WP_004301761.1](file:///Volumes/ESD-USB/Summer%202018/Hassan%20Submission%20PLosOne/content.html#lcl|NZ_CP012938.1_prot_WP_004301761.1_4340) |  |  |  |  |  | + |  | [2.A.115.1.2](http://tcdb.org/search/result.php?tc=2.A.115.1.2) | -44 | P94357 | Drugs | Aminocoumarin |
| [WP_004298714.1](file:///Volumes/ESD-USB/Summer%202018/Hassan%20Submission%20PLosOne/content.html#lcl|NZ_CP012938.1_prot_WP_004298714.1_3070) |  |  |  |  |  | + |  | [2.A.115.1.2](http://tcdb.org/search/result.php?tc=2.A.115.1.2) | -28 | P94357 | Drugs | Aminocoumarin |
| [WP_007219113.1](file:///Volumes/ESD-USB/Summer%202018/Hassan%20Submission%20PLosOne/content.html#lcl|NZ_CP012801.1_prot_WP_007219113.1_2808) |  |  | + |  |  |  |  | [2.A.115.2.10](http://tcdb.org/search/result.php?tc=2.A.115.2.10) | -50 | A2SSB1 | Drugs | Novobiocin |
| WP_008764884.1 |  |  |  | + |  |  |  | 2.A.115.2.10 | -36 | A2SSB1 | Drugs | Aminocoumarin |
| [WP_004297076.1](file:///Volumes/ESD-USB/Summer%202018/Hassan%20Submission%20PLosOne/content.html#lcl|NZ_CP012938.1_prot_WP_004297076.1_1380) |  |  |  |  |  | + |  | [2.A.115.2.10](http://tcdb.org/search/result.php?tc=2.A.115.2.10) | -36 | A2SSB1 | Drugs | Aminocoumarin |
| WP_008766580.1 |  |  |  | + |  |  |  | 2.A.115.2.14 | -35 | C7N858 | Drugs | Aminocoumarin |
| YP_098418.1 |  |  |  |  | + |  |  | 2.A.118.1.3 | 0 | F9Z4G3 | Amino acids | Amino acids |
| ABI39_07350 | + |  |  |  |  |  |  | 2.A.119.1.2 | -41 | A8VTI4 | Unknown | Unknown |
| ABI39_07355 | + |  |  |  |  |  |  | 2.A.119.1.2 | -32 | A8VTI4 | Unknown | Unknown |
| [WP_013617720.1](file:///Volumes/ESD-USB/Summer%202018/Hassan%20Submission%20PLosOne/content.html#lcl|NC_015164.1_prot_WP_013617720.1_1690) |  | + |  |  |  |  |  | [2.A.122.1.1](http://tcdb.org/search/result.php?tc=2.A.122.1.1) | -54 | C6EAH0 | Unknown | Unknown |
| [WP_029425995.1](file:///Volumes/ESD-USB/Summer%202018/Hassan%20Submission%20PLosOne/content.html#lcl|NZ_CP012801.1_prot_WP_029425995.1_2078) |  |  | + |  |  |  |  | [2.A.122.1.1](http://tcdb.org/search/result.php?tc=2.A.122.1.1) | -51 | C6EAH0 | Unknown | Unknown |
| WP_008762629.1 |  |  |  | + |  |  |  | 2.A.122.1.1 | -52 | C6EAH0 | Unknown | Unknown |
| YP_097756.1 |  |  |  |  | + |  |  | 2.A.122.1.1 | -54 | C6EAH0 | Unknown | Unknown |
| YP_100719.1 |  |  |  |  | + |  |  | 2.A.123.4.1 | -63 | A0M0P7 | Unknown | Unknown |
| [WP_013618686.1](file:///Volumes/ESD-USB/Summer%202018/Hassan%20Submission%20PLosOne/content.html#lcl|NC_015164.1_prot_WP_013618686.1_2725) |  | + |  |  |  |  |  | [2.A.124.1.2](http://tcdb.org/search/result.php?tc=2.A.124.1.2) | -103 | S0F499 | Amino Acids | Lysine |
| [WP_007210896.1](file:///Volumes/ESD-USB/Summer%202018/Hassan%20Submission%20PLosOne/content.html#lcl|NZ_CP012801.1_prot_WP_007210896.1_2278) |  |  | + |  |  |  |  | [2.A.124.1.2](http://tcdb.org/search/result.php?tc=2.A.124.1.2) | -113 | S0F499 | Amino Acids | Lysine |
| [WP_022208430.1](file:///Volumes/ESD-USB/Summer%202018/Hassan%20Submission%20PLosOne/content.html#lcl|NZ_CP012801.1_prot_WP_022208430.1_2277) |  |  | + |  |  |  |  | [2.A.124.1.2](http://tcdb.org/search/result.php?tc=2.A.124.1.2) | -28 | S0F499 | Amino Acids | Lysine |
| [WP_007212699.1](file:///Volumes/ESD-USB/Summer%202018/Hassan%20Submission%20PLosOne/content.html#lcl|NZ_CP012801.1_prot_WP_007212699.1_4056) |  |  | + |  |  |  |  | [2.A.124.1.2](http://tcdb.org/search/result.php?tc=2.A.124.1.2) | -10 | S0F499 | Amino Acids | Lysine |
| WP_008759728.1 |  |  |  | + |  |  |  | 2.A.124.1.2 | -103 | S0F499 | Amino acids | Lysine |
| YP_101152.1 |  |  |  |  | + |  |  | 2.A.124.1.2 | -108 | S0F499 | Unknown | Unknown |
| [WP_004299314.1](file:///Volumes/ESD-USB/Summer%202018/Hassan%20Submission%20PLosOne/content.html#lcl|NZ_CP012938.1_prot_WP_004299314.1_2149) |  |  |  |  |  | + |  | [2.A.124.1.2](http://tcdb.org/search/result.php?tc=2.A.124.1.2) | -101 | S0F499 | Unknown | Unknown |
| [WP_005838876.1](file:///Volumes/ESD-USB/Summer%202018/Library/Application%20Support/Microsoft/Office/Office%202011%20AutoRecovery/content.html#lcl|NC_009614.1_prot_WP_005838876.1_1349) |  |  |  |  |  |  | + | [2.A.124.1.2](http://tcdb.org/search/result.php?tc=2.A.124.1.2) | -110 | S0F499 | Amino Acids | Lysine |
| ABI39_15330 | + |  |  |  |  |  |  | 2.A.124.1.4 | -130 | Q64U72 | Amino Acids | Alanine |
| ABI39_19975 | + |  |  |  |  |  |  | 2.A.124.1.4 | -92 | Q64U72 | Amino Acids | Alanine |
| [WP_013616687.1](file:///Volumes/ESD-USB/Summer%202018/Hassan%20Submission%20PLosOne/content.html#lcl|NC_015164.1_prot_WP_013616687.1_624) |  | + |  |  |  |  |  | [2.A.124.1.4](http://tcdb.org/search/result.php?tc=2.A.124.1.4) | -56 | Q64U72 | Amino Acids | Lysine |
| [WP_007212700.1](file:///Volumes/ESD-USB/Summer%202018/Hassan%20Submission%20PLosOne/content.html#lcl|NZ_CP012801.1_prot_WP_007212700.1_4057) |  |  | + |  |  |  |  | [2.A.124.1.4](http://tcdb.org/search/result.php?tc=2.A.124.1.4) | -111 | Q64U72 | Amino Acids | Lysine |
| WP_008761396.1 |  |  |  | + |  |  |  | 2.A.124.1.4 | -124 | Q64U72 | Amino acids | Lysine |
| YP_099491.1 |  |  |  |  | + |  |  | 2.A.124.1.4 | -148 | Q64U72 | Unknown | Unknown |
| [WP_004295766.1](file:///Volumes/ESD-USB/Summer%202018/Hassan%20Submission%20PLosOne/content.html#lcl|NZ_CP012938.1_prot_WP_004295766.1_888) |  |  |  |  |  | + |  | [2.A.124.1.4](http://tcdb.org/search/result.php?tc=2.A.124.1.4) | -123 | Q64U72 | Unknown | Unknown |
| [WP_005846620.1](file:///Volumes/ESD-USB/Summer%202018/Library/Application%20Support/Microsoft/Office/Office%202011%20AutoRecovery/content.html#lcl|NC_009614.1_prot_WP_005846620.1_2993) |  |  |  |  |  |  | + | [2.A.124.1.4](http://tcdb.org/search/result.php?tc=2.A.124.1.4) | -129 | Q64U72 | Unknown | Unknown |
| [WP_008782404.1](file:///Volumes/ESD-USB/Summer%202018/Library/Application%20Support/Microsoft/Office/Office%202011%20AutoRecovery/content.html#lcl|NC_009614.1_prot_WP_008782404.1_3767) |  |  |  |  |  |  | + | [2.A.124.1.4](http://tcdb.org/search/result.php?tc=2.A.124.1.4) | -92 | Q64U72 | Unknown | Unknown |
| [WP_008666399.1](file:///Volumes/ESD-USB/Summer%202018/Library/Application%20Support/Microsoft/Office/Office%202011%20AutoRecovery/content.html#lcl|NC_009614.1_prot_WP_008666399.1_1248) |  |  |  |  |  |  | + | [2.A.127.1.3](http://tcdb.org/search/result.php?tc=2.A.127.1.3) | -06 | V6AJ90 | Unknown | Unknown |
| [WP_049764434.1](file:///Volumes/ESD-USB/Summer%202018/Library/Application%20Support/Microsoft/Office/Office%202011%20AutoRecovery/content.html#lcl|NC_009614.1_prot_WP_049764434.1_1057) |  |  |  |  |  |  | + | [2.A.127.1.3](http://tcdb.org/search/result.php?tc=2.A.127.1.3) | -05 | V6AJ90 | Unknown | Unknown |
| ABI39_01965 | + |  |  |  |  |  |  | 3.A.1.2.6 | -30 | P39265 | Sugars | Allose |
| ABI39_03985 | + |  |  |  |  |  |  | 3.A.1.2.13 | -10 | A6VKT0 | Sugars | Monosaccharides |
| [WP_013618747.1](file:///Volumes/ESD-USB/Summer%202018/Hassan%20Submission%20PLosOne/content.html#lcl|NC_015164.1_prot_WP_013618747.1_2789) |  | + |  |  |  |  |  | [3.A.1.2.13](http://tcdb.org/search/result.php?tc=3.A.1.2.13) | -22 | A6VKT0 | Unknown | Unknown |
| [WP_007217410.1](file:///Volumes/ESD-USB/Summer%202018/Hassan%20Submission%20PLosOne/content.html#lcl|NZ_CP012801.1_prot_WP_007217410.1_1233) |  |  | + |  |  |  |  | [3.A.1.2.13](http://tcdb.org/search/result.php?tc=3.A.1.2.13) | -13 | A6VKT0 | Sugars | Monosaccharides |
| [WP_007219450.1](file:///Volumes/ESD-USB/Summer%202018/Hassan%20Submission%20PLosOne/content.html#lcl|NZ_CP012801.1_prot_WP_007219450.1_3099) |  |  | + |  |  |  |  | [3.A.1.2.13](http://tcdb.org/search/result.php?tc=3.A.1.2.13) | -10 | A6VKT0 | Sugars | Monosaccharides |
| [WP_022209766.1](file:///Volumes/ESD-USB/Summer%202018/Hassan%20Submission%20PLosOne/content.html#lcl|NZ_CP012801.1_prot_WP_022209766.1_4527) |  |  | + |  |  |  |  | [3.A.1.2.13](http://tcdb.org/search/result.php?tc=3.A.1.2.13) | -08 | A6VKT0 | Sugars | Monosaccharides |
| YP_101807.1 |  |  |  |  | + |  |  | 3.A.1.2.13 | -22 | A6VKT0 | Sugars | Monosaccharides |
| YP_100586.1 |  |  |  |  | + |  |  | 3.A.1.2.13 | -11 | A6VKT0 | Sugars | Monosaccharides |
| YP_100617.1 |  |  |  |  | + |  |  | 3.A.1.2.14 | -28 | Q0HIQ8 | Sugars | Arabinose |
| [WP_052588004.1](file:///Volumes/ESD-USB/Summer%202018/Hassan%20Submission%20PLosOne/content.html#lcl|NZ_CP012938.1_prot_WP_052588004.1_1743) |  |  |  |  |  | + |  | [3.A.1.2.14](http://tcdb.org/search/result.php?tc=3.A.1.2.14) | -27 | Q0HIQ8 | Sugars | Arabinose |
| [WP_013619440.1](file:///Volumes/ESD-USB/Summer%202018/Hassan%20Submission%20PLosOne/content.html#lcl|NC_015164.1_prot_WP_013619440.1_3488) |  | + |  |  |  |  |  | [3.A.1.2.20](http://tcdb.org/search/result.php?tc=3.A.1.2.20) | -14 | G4FGN5 | Unknown | Unknown |
| [WP_022210480.1](file:///Volumes/ESD-USB/Summer%202018/Hassan%20Submission%20PLosOne/content.html#lcl|NZ_CP012801.1_prot_WP_022210480.1_2909) |  |  | + |  |  |  |  | [3.A.1.2.20](http://tcdb.org/search/result.php?tc=3.A.1.2.20) | -13 | G4FGN5 | Sugars | Glucose |
| [WP_033160614.1](file:///Volumes/ESD-USB/Summer%202018/Hassan%20Submission%20PLosOne/content.html#lcl|NZ_CP012801.1_prot_WP_033160614.1_4982) |  |  | + |  |  |  |  | [3.A.1.2.20](http://tcdb.org/search/result.php?tc=3.A.1.2.20) | -11 | G4FGN5 | Sugars | Glucose |
| [WP_029428080.1](file:///Volumes/ESD-USB/Summer%202018/Hassan%20Submission%20PLosOne/content.html#lcl|NZ_CP012801.1_prot_WP_029428080.1_5209) |  |  | + |  |  |  |  | [3.A.1.2.20](http://tcdb.org/search/result.php?tc=3.A.1.2.20) | -11 | G4FGN5 | Sugars | Glucose |
| WP_062695076.1 |  |  |  | + |  |  |  | 3.A.1.2.20 | -12 | G4FGN5 | Sugars | Glucose/Xylose |
| WP_011107293.1 |  |  |  | + |  |  |  | 3.A.1.2.20 | -10 | G4FGN5 | Sugars | Glucose/Xylose |
| WP_008762342.1 |  |  |  | + |  |  |  | 3.A.1.2.20 | -07 | G4FGN5 | Sugars | Glucose/Xylose |
| [WP_033160653.1](file:///Volumes/ESD-USB/Summer%202018/Hassan%20Submission%20PLosOne/content.html#lcl|NZ_CP012801.1_prot_WP_033160653.1_1263) |  |  | + |  |  |  |  | [3.A.1.2.25](http://tcdb.org/search/result.php?tc=3.A.1.2.25) | -25 | P39325 | Sugars | Glucose |
| WP_025726485.1 |  |  | + |  |  |  |  | [3.A.1.3.15](http://tcdb.org/search/result.php?tc=3.A.1.3.15) | -07 | P54535 | Amino Acids | Arginine |
| YP_098925.1 |  |  |  |  | + |  |  | 3.A.1.3.15 | -10 | P54535 | Amino acids | Arginine |
| [WP_012055631.1](file:///Volumes/ESD-USB/Summer%202018/Library/Application%20Support/Microsoft/Office/Office%202011%20AutoRecovery/content.html#lcl|NC_009614.1_prot_WP_012055631.1_3249) |  |  |  |  |  |  | + | [3.A.1.3.21](http://tcdb.org/search/result.php?tc=3.A.1.3.21) | -30 | Q9I488 | Amino Acids | Proline |
| ABI39_17295 | + |  |  |  |  |  |  | 3.A.1.3.22 | -30 | Q9I405 | Amino Acids | Glutamine/Glutamic acid |
| [WP_029428125.1](file:///Volumes/ESD-USB/Summer%202018/Hassan%20Submission%20PLosOne/content.html#lcl|NZ_CP012801.1_prot_WP_029428125.1_5140) |  |  | + |  |  |  |  | [3.A.1.3.25](http://tcdb.org/search/result.php?tc=3.A.1.3.25) | -24 | Q9CES4 | Amino Acids | Glutamine |
| WP_011107201.1 |  |  |  | + |  |  |  | 3.A.1.3.27 | -11 | Q8RCC4 | Amino acids | Amino Acids |
| [WP_004297413.1](file:///Volumes/ESD-USB/Summer%202018/Hassan%20Submission%20PLosOne/content.html#lcl|NZ_CP012938.1_prot_WP_004297413.1_171) |  |  |  |  |  | + |  | [3.A.1.3.27](http://tcdb.org/search/result.php?tc=3.A.1.3.27) | -09 | Q8RCC4 | Amino Acids | Arginine/Lysine/Histidine |
| [WP_029427044.1](file:///Volumes/ESD-USB/Summer%202018/Hassan%20Submission%20PLosOne/content.html#lcl|NZ_CP012801.1_prot_WP_029427044.1_171) |  |  | + |  |  |  |  | [3.A.1.4.3](http://tcdb.org/search/result.php?tc=3.A.1.4.3) | -07 | Q9L3M3 | Unknown | Unknown |
| WP_029427043.1 |  |  | + |  |  |  |  | [3.A.1.4.6](http://tcdb.org/search/result.php?tc=3.A.1.4.6) | -48 | Q8YT15 | Unknown | Unknown |
| [WP_029427042.1](file:///Volumes/ESD-USB/Summer%202018/Hassan%20Submission%20PLosOne/content.html#lcl|NZ_CP012801.1_prot_WP_029427042.1_173) |  |  | + |  |  |  |  | [3.A.1.4.8](http://tcdb.org/search/result.php?tc=3.A.1.4.8) | -50 | P21629 | Amino acids | Alanine |
| [WP_029427041.1](file:///Volumes/ESD-USB/Summer%202018/Hassan%20Submission%20PLosOne/content.html#lcl|NZ_CP012801.1_prot_WP_029427041.1_174) |  |  | + |  |  |  |  | [3.A.1.4.8](http://tcdb.org/search/result.php?tc=3.A.1.4.8) | -34 | P21628 | Amino acids | Alanine |
| [WP_029427040.1](file:///Volumes/ESD-USB/Summer%202018/Hassan%20Submission%20PLosOne/content.html#lcl|NZ_CP012801.1_prot_WP_029427040.1_175) |  |  | + |  |  |  |  | [3.A.1.4.10](http://tcdb.org/search/result.php?tc=3.A.1.4.10) | -28 | Q8DQI0 | Amino Acids |  |
| ABI39_04585 | + |  |  |  |  |  |  | 3.A.1.5.3 | -19 | P33594 | Cations | Ni^2+^ |
| WP_008763669.1 |  |  |  | + |  |  |  | 3.A.1.5.3 | -17 | P33594 | Cations | Ni^2+^ |
| YP_097550.1_267 |  |  |  |  | + |  |  | 3.A.1.5.3 | -18 | P33594 | Metal | Ni^2+^ |
| [WP_005838860.1](file:///Volumes/ESD-USB/Summer%202018/Library/Application%20Support/Microsoft/Office/Office%202011%20AutoRecovery/content.html#lcl|NC_009614.1_prot_WP_005838860.1_1342) |  |  |  |  |  |  | + | [3.A.1.5.6](http://tcdb.org/search/result.php?tc=3.A.1.5.6) | -18 | Q8U1J5 | Sugar derivative | Cellobiose (β-1, 4), cellotriose, cellotetraose, cellopentaose, laminaribiose (β-1,3), laminaritriose, sophorose |
| ABI39_09985 | + |  |  |  |  |  |  | 3.A.1.5.24 | -05 | Q8ZNJ8 | Drugs | PolymyxinB/Protamine |
| [WP_007212442.1](file:///Volumes/ESD-USB/Summer%202018/Hassan%20Submission%20PLosOne/content.html#lcl|NZ_CP012801.1_prot_WP_007212442.1_3837) |  |  | + |  |  |  |  | [3.A.1.5.24](http://tcdb.org/search/result.php?tc=3.A.1.5.24) | -05 | Q8ZNJ8 | Peptides | Protamine/Melittin |
| WP_008764293.1 |  |  |  | + |  |  |  | 3.A.1.5.24 | -06 | Q8ZNJ8 | Drugs | Drugs |
| [WP_005843165.1](file:///Volumes/ESD-USB/Summer%202018/Library/Application%20Support/Microsoft/Office/Office%202011%20AutoRecovery/content.html#lcl|NC_009614.1_prot_WP_005843165.1_3841) |  |  |  |  |  |  | + | [3.A.1.5.24](http://tcdb.org/search/result.php?tc=3.A.1.5.24) | -05 | Q8ZNJ8 | Unknown | Unknown |
| [WP_007210912.1](file:///Volumes/ESD-USB/Summer%202018/Hassan%20Submission%20PLosOne/content.html#lcl|NZ_CP012801.1_prot_WP_007210912.1_2291) |  |  | + |  |  |  |  | [3.A.1.5.35](http://tcdb.org/search/result.php?tc=3.A.1.5.35) | -18 | Q9F350 | Unknown | Unknown |
| [WP_004298251.1](file:///Volumes/ESD-USB/Summer%202018/Hassan%20Submission%20PLosOne/content.html#lcl|NZ_CP012938.1_prot_WP_004298251.1_3388) |  |  |  |  |  | + |  | [3.A.1.5.35](http://tcdb.org/search/result.php?tc=3.A.1.5.35) | -18 | Q9F350 | Unknown | Unknown |
| ABI39_14285 | + |  |  |  |  |  |  | 3.A.1.6.3 | -21 | P0A4W2 | Anions | SO_4_^2-^ |
| ABI39_20365 | + |  |  |  |  |  |  | 3.A.1.7.1 | -109 | P0AAH0 | Anions | PO_4_^3-^ |
| [WP_013619503.1](file:///Volumes/ESD-USB/Summer%202018/Hassan%20Submission%20PLosOne/content.html#lcl|NC_015164.1_prot_WP_013619503.1_3551) |  | + |  |  |  |  |  | [3.A.1.7.1](http://tcdb.org/search/result.php?tc=3.A.1.7.1) | -113 | P0AAH0 | Anions | PO_4_^3-^ |
| [WP_007209709.1](file:///Volumes/ESD-USB/Summer%202018/Hassan%20Submission%20PLosOne/content.html#lcl|NZ_CP012801.1_prot_WP_007209709.1_4867) |  |  | + |  |  |  |  | [3.A.1.7.1](http://tcdb.org/search/result.php?tc=3.A.1.7.1) | -109 | P0AAH0 | Unknown | Unknown |
| WP_008764960.1 |  |  |  | + |  |  |  | 3.A.1.7.1 | -114 | P0AAH0 | Drugs | Protamine, melittin, polymyxin B, human defensin |
| YP_100038.1 |  |  |  |  | + |  |  | 3.A.1.7.1 | -111 | P0AAH0 | Anions | PO_4_^3-^ |
| WP_004296658.1 |  |  |  |  |  | + |  | [3.A.1.7.1](http://tcdb.org/search/result.php?tc=3.A.1.7.1) | -113 | P0AAH0 | Unknown | Unknown |
| [WP_005843166.1](file:///Volumes/ESD-USB/Summer%202018/Library/Application%20Support/Microsoft/Office/Office%202011%20AutoRecovery/content.html#lcl|NC_009614.1_prot_WP_005843166.1_3840) |  |  |  |  |  |  | + | [3.A.1.7.1](http://tcdb.org/search/result.php?tc=3.A.1.7.1) | -109 | P0AAH0 | Anions | PO_4_^3-^ |
| [WP_004301432.1](file:///Volumes/ESD-USB/Summer%202018/Hassan%20Submission%20PLosOne/content.html#lcl|NZ_CP012938.1_prot_WP_004301432.1_4131) |  |  |  |  |  | + |  | [3.A.1.7.4](http://tcdb.org/search/result.php?tc=3.A.1.7.4) | -05 | P95302 | Anions | PO_4_^3-^ |
| ABI39_20375 | **+** |  |  |  |  |  |  | 3.A.1.7.5 | -53 | O51234 | Anions | PO_4_^3-^ |
| ABI39_20370 | **+** |  |  |  |  |  |  | 3.A.1.7.5 | -42 | O51235 | Anions | PO_4_^3-^ |
| ABI39_20380 | **+** |  |  |  |  |  |  | 3.A.1.7.5 | -30 | O51233 | Anions | PO_4_^3-^ |
| ABI39_08005 | **+** |  |  |  |  |  |  | 3.A.1.7.5 | -09 | O51233 | Anions | PO_4_^3-^ |
| [WP_013619505.1](file:///Volumes/ESD-USB/Summer%202018/Hassan%20Submission%20PLosOne/content.html#lcl|NC_015164.1_prot_WP_013619505.1_3553) |  | + |  |  |  |  |  | [3.A.1.7.5](http://tcdb.org/search/result.php?tc=3.A.1.7.5) | -55 | O51234 | Anions | PO_4_^3-^ |
| [WP_013619504.1](file:///Volumes/ESD-USB/Summer%202018/Hassan%20Submission%20PLosOne/content.html#lcl|NC_015164.1_prot_WP_013619504.1_3552) |  | + |  |  |  |  |  | [3.A.1.7.5](http://tcdb.org/search/result.php?tc=3.A.1.7.5) | -44 | O51235 | Anions | PO_4_^3-^ |
| [WP_007209711.1](file:///Volumes/ESD-USB/Summer%202018/Hassan%20Submission%20PLosOne/content.html#lcl|NZ_CP012801.1_prot_WP_007209711.1_4865) |  |  | + |  |  |  |  | [3.A.1.7.5](http://tcdb.org/search/result.php?tc=3.A.1.7.5) | -55 | O51234 | Anions | PO_4_^3-^ |
| [WP_007216331.1](file:///Volumes/ESD-USB/Summer%202018/Hassan%20Submission%20PLosOne/content.html#lcl|NZ_CP012801.1_prot_WP_007216331.1_4866) |  |  | + |  |  |  |  | [3.A.1.7.5](http://tcdb.org/search/result.php?tc=3.A.1.7.5) | -42 | O51235 | Anions | PO_4_^3-^ |
| [WP_007216329.1](file:///Volumes/ESD-USB/Summer%202018/Hassan%20Submission%20PLosOne/content.html#lcl|NZ_CP012801.1_prot_WP_007216329.1_4864) |  |  | + |  |  |  |  | [3.A.1.7.5](http://tcdb.org/search/result.php?tc=3.A.1.7.5) | -30 | O51233 | Unknown | Unknown |
| [WP_026367266.1](file:///Volumes/ESD-USB/Summer%202018/Hassan%20Submission%20PLosOne/content.html#lcl|NZ_CP012801.1_prot_WP_026367266.1_3333) |  |  | + |  |  |  |  | [3.A.1.7.5](http://tcdb.org/search/result.php?tc=3.A.1.7.5) | -09 | O51233 | Unknown | Unknown |
| WP_008764958.1 |  |  |  | + |  |  |  | 3.A.1.7.5 | -55 | O51234 | Anions | PO_4_^3-^ |
| WP_008764959.1 |  |  |  | + |  |  |  | 3.A.1.7.5 | -41 | O51235 | Unknown | Unknown |
| WP_008762446.1 |  |  |  | + |  |  |  | 3.A.1.7.5 | -29 | O51233 | Anions | PO_4_^3-^ |
| YP_100040.1 |  |  |  |  | + |  |  | 3.A.1.7.5 | -55 | O51234 | Anions | PO_4_^3-^ |
| YP_100039.1 |  |  |  |  | + |  |  | 3.A.1.7.5 | -39 | O51235 | Anions | PO_4_^3-^ |
| WP_008763547.1 |  |  |  | + |  |  |  | 3.A.1.7.5 | -09 | O51233 | Anions | PO_4_^3-^ |
| [WP_008671314.1](file:///Volumes/ESD-USB/Summer%202018/Library/Application%20Support/Microsoft/Office/Office%202011%20AutoRecovery/content.html#lcl|NC_009614.1_prot_WP_008671314.1_3842) |  |  |  |  |  |  | + | [3.A.1.7.5](http://tcdb.org/search/result.php?tc=3.A.1.7.5) | -53 | O51234 | Anions | PO_4_^3-^ |
| [WP_005839165.1](file:///Volumes/ESD-USB/Summer%202018/Library/Application%20Support/Microsoft/Office/Office%202011%20AutoRecovery/content.html#lcl|NC_009614.1_prot_WP_005839165.1_1603) |  |  |  |  |  |  | + | [3.A.1.7.5](http://tcdb.org/search/result.php?tc=3.A.1.7.5) | -42 | O51235 | Anions | PO_4_^3-^ |
| [WP_012055762.1](file:///Volumes/ESD-USB/Summer%202018/Library/Application%20Support/Microsoft/Office/Office%202011%20AutoRecovery/content.html#lcl|NC_009614.1_prot_WP_012055762.1_3479) |  |  |  |  |  |  | + | [3.A.1.7.5](http://tcdb.org/search/result.php?tc=3.A.1.7.5) | -30 | O51233 | Anions | PO_4_^3-^ |
| [WP_005839542.1](file:///Volumes/ESD-USB/Summer%202018/Library/Application%20Support/Microsoft/Office/Office%202011%20AutoRecovery/content.html#lcl|NC_009614.1_prot_WP_005839542.1_3477) |  |  |  |  |  |  | + | [3.A.1.7.5](http://tcdb.org/search/result.php?tc=3.A.1.7.5) | -08 | O51233 | Anions | PO_4_^3-^ |
| [WP_029428192.1](file:///Volumes/ESD-USB/Summer%202018/Hassan%20Submission%20PLosOne/content.html#lcl|NZ_CP012801.1_prot_WP_029428192.1_5030) |  |  | + |  |  |  |  | [3.A.1.11.7](http://tcdb.org/search/result.php?tc=3.A.1.11.7) | -46 | Q97Q45 | Polyamines | Spermidine/Putrescine |
| WP_008762999.1 |  |  |  | + |  |  |  | 3.A.1.11.7 | -55 | Q97Q44 | Amines | Spermidine/Putrescine |
| WP_062695921.1 |  |  |  | + |  |  |  | 3.A.1.11.7 | -52 | Q97Q45 | Amines | Spermidine/Putrescine |
| YP_100004.1 |  |  |  |  | + |  |  | 3.A.1.11.7 | -48 | Q97Q45 | Amines | Spermidine/Putrescine |
| ABI39_17815 | **+** |  |  |  |  |  |  | 3.A.1.11.8 | -131 | Q5HGY5 | Amines | Spermidine |
| ABI39_17805 | **+** |  |  |  |  |  |  | 3.A.1.11.8 | -56 | Q7A677 | Amines | Spermidine |
| ABI39_17800 | **+** |  |  |  |  |  |  | 3.A.1.11.8 | -48 | Q7A676 | Amines | Spermidine |
| ABI39_17810 | **+** |  |  |  |  |  |  | 3.A.1.11.8 | -41 | Q7A678 | Amines | Spermidine |
| [WP_022210240.1](file:///Volumes/ESD-USB/Summer%202018/Hassan%20Submission%20PLosOne/content.html#lcl|NZ_CP012801.1_prot_WP_022210240.1_5033) |  |  | + |  |  |  |  | [3.A.1.11.8](http://tcdb.org/search/result.php?tc=3.A.1.11.8) | -133 | Q5HGY5 | Polyamines | Spermidine/Putrescine |
| [WP_007214337.1](file:///Volumes/ESD-USB/Summer%202018/Hassan%20Submission%20PLosOne/content.html#lcl|NZ_CP012801.1_prot_WP_007214337.1_5031) |  |  | + |  |  |  |  | [3.A.1.11.8](http://tcdb.org/search/result.php?tc=3.A.1.11.8) | -55 | Q7A677 | Polyamines | Spermine/Spermidine |
| [WP_033160611.1](file:///Volumes/ESD-USB/Summer%202018/Hassan%20Submission%20PLosOne/content.html#lcl|NZ_CP012801.1_prot_WP_033160611.1_5032) |  |  | + |  |  |  |  | [3.A.1.11.8](http://tcdb.org/search/result.php?tc=3.A.1.11.8) | -44 | Q7A678 | Polyamines | Spermine/Spermidine |
| WP_032813749.1 |  |  |  | + |  |  |  | 3.A.1.11.8 | -131 | Q5HGY5 | Amines | Spermidine/Putrescine |
| WP_008762998.1 |  |  |  | + |  |  |  | 3.A.1.11.8 | -44 | Q7A678 | Amines | Spermidine/Spermine |
| YP_100007.1 |  |  |  |  | + |  |  | 3.A.1.11.8 | -131 | Q5HGY5 | Amines | Spermidine |
| YP_100005.1 |  |  |  |  | + |  |  | 3.A.1.11.8 | -55 | Q7A677 | Amines | Spermidine |
| YP_100006.1 |  |  |  |  | + |  |  | 3.A.1.11.8 | -41 | Q7A678 | Amines | Spermidine |
| [WP_008999649.1](file:///Volumes/ESD-USB/Summer%202018/Hassan%20Submission%20PLosOne/content.html#lcl|NZ_CP012938.1_prot_WP_008999649.1_1252) |  |  |  |  |  | + |  | [3.A.1.11.8](http://tcdb.org/search/result.php?tc=3.A.1.11.8) | -130 | Q5HGY5 | Amines | Spermine/Spermidine |
| [WP_004297655.1](file:///Volumes/ESD-USB/Summer%202018/Hassan%20Submission%20PLosOne/content.html#lcl|NZ_CP012938.1_prot_WP_004297655.1_1250) |  |  |  |  |  | + |  | [3.A.1.11.8](http://tcdb.org/search/result.php?tc=3.A.1.11.8) | -54 | Q7A677 | Amines | Spermine/Spermidine |
| [WP_004297656.1](file:///Volumes/ESD-USB/Summer%202018/Hassan%20Submission%20PLosOne/content.html#lcl|NZ_CP012938.1_prot_WP_004297656.1_1249) |  |  |  |  |  | + |  | [3.A.1.11.8](http://tcdb.org/search/result.php?tc=3.A.1.11.8) | -49 | Q7A676 | Amines | Spermine/Spermidine |
| [WP_004308090.1](file:///Volumes/ESD-USB/Summer%202018/Hassan%20Submission%20PLosOne/content.html#lcl|NZ_CP012938.1_prot_WP_004308090.1_1251) |  |  |  |  |  | + |  | [3.A.1.11.8](http://tcdb.org/search/result.php?tc=3.A.1.11.8) | -42 | Q7A678 | Amines | Spermine/Spermidine |
| [WP_012055761.1](file:///Volumes/ESD-USB/Summer%202018/Library/Application%20Support/Microsoft/Office/Office%202011%20AutoRecovery/content.html#lcl|NC_009614.1_prot_WP_012055761.1_3476) |  |  |  |  |  |  | + | [3.A.1.11.8](http://tcdb.org/search/result.php?tc=3.A.1.11.8) | -131 | Q5HGY5 | Amines | Spermidine/Spermine |
| [WP_005839541.1](file:///Volumes/ESD-USB/Summer%202018/Library/Application%20Support/Microsoft/Office/Office%202011%20AutoRecovery/content.html#lcl|NC_009614.1_prot_WP_005839541.1_3478) |  |  |  |  |  |  | + | [3.A.1.11.8](http://tcdb.org/search/result.php?tc=3.A.1.11.8) | -55 | Q7A677 | Amines | Spermidine/Spermine |
| [WP_005841255.1](file:///Volumes/ESD-USB/Summer%202018/Library/Application%20Support/Microsoft/Office/Office%202011%20AutoRecovery/content.html#lcl|NC_009614.1_prot_WP_005841255.1_3205) |  |  |  |  |  |  | + | [3.A.1.11.8](http://tcdb.org/search/result.php?tc=3.A.1.11.8) | -48 | Q7A676 | Amines | Spermidine/Spermine |
| [WP_005852472.1](file:///Volumes/ESD-USB/Summer%202018/Library/Application%20Support/Microsoft/Office/Office%202011%20AutoRecovery/content.html#lcl|NC_009614.1_prot_WP_005852472.1_3204) |  |  |  |  |  |  | + | [3.A.1.11.8](http://tcdb.org/search/result.php?tc=3.A.1.11.8) | -42 | Q7A678 | Amines | Spermidine/Spermine |
| ABI39_17065 | + |  |  |  |  |  |  | 3.A.1.12.2 | -98 | P46921 | Amino Acids | Glycine/betaine |
| WP_008763296.1 |  |  |  | + |  |  |  | 3.A.1.12.2 | -98 | P46921 | Amino acids | Glycine/betaine |
| YP_100613.1 |  |  |  |  | + |  |  | 3.A.1.12.2 | -98 | P46921 | Amino acids | Glycine/betaine |
| [WP_004300983.1](file:///Volumes/ESD-USB/Summer%202018/Hassan%20Submission%20PLosOne/content.html#lcl|NZ_CP012938.1_prot_WP_004300983.1_1739) |  |  |  |  |  | + |  | [3.A.1.12.2](http://tcdb.org/search/result.php?tc=3.A.1.12.2) | -95 | P46921 | Amino Acids | Glycine/betaine |
| [WP_012055620.1](file:///Volumes/ESD-USB/Summer%202018/Library/Application%20Support/Microsoft/Office/Office%202011%20AutoRecovery/content.html#lcl|NC_009614.1_prot_WP_012055620.1_3206) |  |  |  |  |  |  | + | [3.A.1.12.2](http://tcdb.org/search/result.php?tc=3.A.1.12.2) | -98 | P46921 | Amino Acids | Glycine/betaine |
| ABI39_17060 | + |  |  |  |  |  |  | 3.A.1.12.9 | -143 | Q8U4S5 | Amino Acids | Glycine/betaine |
| ABI39_17070 | + |  |  |  |  |  |  | 3.A.1.12.9 | -67 | Q8U4S3 | Amino Acids | Glycine/betaine |
| WP_008767787.1 |  |  |  | + |  |  |  | 3.A.1.12.9 | -145 | Q8U4S5 | Amino acids | Glycine/betaine |
| YP_100614.1 |  |  |  |  | + |  |  | 3.A.1.12.9 | -141 | Q8U4S5 | Amino acids | Glycine/betaine |
| [WP_004326171.1](file:///Volumes/ESD-USB/Summer%202018/Hassan%20Submission%20PLosOne/content.html#lcl|NZ_CP012938.1_prot_WP_004326171.1_2059) |  |  |  |  |  | + |  | [3.A.1.12.9](http://tcdb.org/search/result.php?tc=3.A.1.12.9) | -141 | Q8U4S5 | Amino Acids | Glycine/betaine |
| [WP_008668681.1](file:///Volumes/ESD-USB/Summer%202018/Library/Application%20Support/Microsoft/Office/Office%202011%20AutoRecovery/content.html#lcl|NC_009614.1_prot_WP_008668681.1_3021) |  |  |  |  |  |  | + | [3.A.1.12.9](http://tcdb.org/search/result.php?tc=3.A.1.12.9) | -143 | Q8U4S5 | Amino Acids | Glycine/betaine |
| WP_055300297.1 |  |  |  | + |  |  |  | 3.A.1.12.12 | -64 | Q9HZ04 | Quaternary ammonium compounds | Choline/carnitine/betaine |
| YP_100612.1 |  |  |  |  | + |  |  | 3.A.1.12.12 | -54 | Q9HZ04 | Amino acids | Glycine/betaine |
| ABI39_15485 | + |  |  |  |  |  |  | 3.A.1.13.2 | -90 | A9WCZ6 | Unknown | Unknown |
| ABI39_10445 | + |  |  |  |  |  |  | 3.A.1.13.2 | -79 | A9WCZ6 | Unknown | Unknown |
| ABI39_15490 | + |  |  |  |  |  |  | 3.A.1.13.2 | -73 | A9WCZ7 | Vitamins | Cobalamin |
| ABI39_10440 | + |  |  |  |  |  |  | 3.A.1.13.2 | -71 | A9WCZ7 | Vitamins | Cobalamin |
| [WP_013617443.1](file:///Volumes/ESD-USB/Summer%202018/Hassan%20Submission%20PLosOne/content.html#lcl|NC_015164.1_prot_WP_013617443.1_1409) |  | + |  |  |  |  |  | [3.A.1.13.2](http://tcdb.org/search/result.php?tc=3.A.1.13.2) | -75 | A9WCZ7 | Vitamins | Cobalamin |
| [WP_041583871.1](file:///Volumes/ESD-USB/Summer%202018/Hassan%20Submission%20PLosOne/content.html#lcl|NC_015164.1_prot_WP_041583871.1_725) |  | + |  |  |  |  |  | [3.A.1.13.2](http://tcdb.org/search/result.php?tc=3.A.1.13.2) | -69 | A9WCZ6 | Vitamins | Cobalamin |
| [WP_013616786.1](file:///Volumes/ESD-USB/Summer%202018/Hassan%20Submission%20PLosOne/content.html#lcl|NC_015164.1_prot_WP_013616786.1_726) |  | + |  |  |  |  |  | [3.A.1.13.2](http://tcdb.org/search/result.php?tc=3.A.1.13.2) | -59 | A9WCZ4 | Vitamins | Cobalamin |
| [WP_007213911.1](file:///Volumes/ESD-USB/Summer%202018/Hassan%20Submission%20PLosOne/content.html#lcl|NZ_CP012801.1_prot_WP_007213911.1_356) |  |  | + |  |  |  |  | [3.A.1.13.2](http://tcdb.org/search/result.php?tc=3.A.1.13.2) | -91 | A9WCZ6 | Vitamins | Cobalamin |
| [WP_029426855.1](file:///Volumes/ESD-USB/Summer%202018/Hassan%20Submission%20PLosOne/content.html#lcl|NZ_CP012801.1_prot_WP_029426855.1_355) |  |  | + |  |  |  |  | [3.A.1.13.2](http://tcdb.org/search/result.php?tc=3.A.1.13.2) | -79 | A9WCZ7 | Vitamins | Cobalamin |
| [WP_029429160.1](file:///Volumes/ESD-USB/Summer%202018/Hassan%20Submission%20PLosOne/content.html#lcl|NZ_CP012801.1_prot_WP_029429160.1_1438) |  |  | + |  |  |  |  | [3.A.1.13.2](http://tcdb.org/search/result.php?tc=3.A.1.13.2) | -75 | A9WCZ7 | Vitamins | Cobalamin |
| [WP_029429159.1](file:///Volumes/ESD-USB/Summer%202018/Hassan%20Submission%20PLosOne/content.html#lcl|NZ_CP012801.1_prot_WP_029429159.1_1437) |  |  | + |  |  |  |  | [3.A.1.13.2](http://tcdb.org/search/result.php?tc=3.A.1.13.2) | -74 | A9WCZ6 | Vitamins | Cobalamin |
| [WP_029426853.1](file:///Volumes/ESD-USB/Summer%202018/Hassan%20Submission%20PLosOne/content.html#lcl|NZ_CP012801.1_prot_WP_029426853.1_357) |  |  | + |  |  |  |  | [3.A.1.13.2](http://tcdb.org/search/result.php?tc=3.A.1.13.2) | -63 | A9WCZ4 | Vitamins | Cobalamin |
| [WP_007210415.1](file:///Volumes/ESD-USB/Summer%202018/Hassan%20Submission%20PLosOne/content.html#lcl|NZ_CP012801.1_prot_WP_007210415.1_1436) |  |  | + |  |  |  |  | [3.A.1.13.2](http://tcdb.org/search/result.php?tc=3.A.1.13.2) | -57 | A9WCZ4 | Vitamins | Cobalamin |
| WP_004311292.1 |  |  |  | + |  |  |  | 3.A.1.13.2 | -75 | A9WCZ6 | Vitamins | Cobalamin |
| WP_062695662.1 |  |  |  | + |  |  |  | 3.A.1.13.2 | -75 | A9WCZ6 | Vitamins | Cobalamin |
| WP_008766585.1 |  |  |  | + |  |  |  | 3.A.1.13.2 | -71 | A9WCZ4 | Vitamins | Cobalamin |
| WP_004289233.1 |  |  |  | + |  |  |  | 3.A.1.13.2 | -71 | A9WCZ4 | Vitamins | Cobalamin |
| WP_008759785.1 |  |  |  | + |  |  |  | 3.A.1.13.2 | -71 | A9WCZ6 | Vitamins | Cobalamin |
| WP_062695659.1 |  |  |  | + |  |  |  | 3.A.1.13.2 | -68 | A9WCZ4 | Vitamins | Cobalamin |
| WP_004289235.1 |  |  |  | + |  |  |  | 3.A.1.13.2 | -65 | A9WCZ7 | Vitamins | Cobalamin |
| WP_062695663.1 |  |  |  | + |  |  |  | 3.A.1.13.2 | -60 | A9WCZ7 | Vitamins | Cobalamin |
| WP_008766586.1 |  |  |  | + |  |  |  | 3.A.1.13.2 | -60 | A9WCZ7 | Vitamins | Cobalamin |
| YP_099474.1 |  |  |  |  | + |  |  | 3.A.1.13.2 | -89 | A9WCZ6 | Vitamins | Cobalamin |
| YP_099475.1 |  |  |  |  | + |  |  | 3.A.1.13.2 | -81 | A9WCZ7 | Vitamins | Cobalamin |
| YP_100307.1 |  |  |  |  | + |  |  | 3.A.1.13.2 | -75 | A9WCZ6 | Vitamins | Cobalamin |
| YP_098503.1 |  |  |  |  | + |  |  | 3.A.1.13.2 | -70 | A9WCZ6 | Vitamins | Cobalamin |
| YP_100306.1 |  |  |  |  | + |  |  | 3.A.1.13.2 | -64 | A9WCZ7 | Vitamins | Cobalamin |
| [WP_004299413.1](file:///Volumes/ESD-USB/Summer%202018/Hassan%20Submission%20PLosOne/content.html#lcl|NZ_CP012938.1_prot_WP_004299413.1_2060) |  |  |  |  |  | + |  | [3.A.1.13.2](http://tcdb.org/search/result.php?tc=3.A.1.13.2) | -75 | A9WCZ6 | Vitamins | Cobalamin |
| [WP_004301256.1](file:///Volumes/ESD-USB/Summer%202018/Hassan%20Submission%20PLosOne/content.html#lcl|NZ_CP012938.1_prot_WP_004301256.1_4014) |  |  |  |  |  | + |  | [3.A.1.13.2](http://tcdb.org/search/result.php?tc=3.A.1.13.2) | -73 | A9WCZ4 | Vitamins | Cobalamin |
| [WP_004299412.1](file:///Volumes/ESD-USB/Summer%202018/Hassan%20Submission%20PLosOne/content.html#lcl|NZ_CP012938.1_prot_WP_004299412.1_2061) |  |  |  |  |  | + |  | [3.A.1.13.2](http://tcdb.org/search/result.php?tc=3.A.1.13.2) | -70 | A9WCZ6 | Vitamins | Cobalamin |
| [WP_004301252.1](file:///Volumes/ESD-USB/Summer%202018/Hassan%20Submission%20PLosOne/content.html#lcl|NZ_CP012938.1_prot_WP_004301252.1_4012) |  |  |  |  |  | + |  | [3.A.1.13.2](http://tcdb.org/search/result.php?tc=3.A.1.13.2) | -69 | A9WCZ4 | Vitamins | Cobalamin |
| [WP_004296695.1](file:///Volumes/ESD-USB/Summer%202018/Hassan%20Submission%20PLosOne/content.html#lcl|NZ_CP012938.1_prot_WP_004296695.1_3181) |  |  |  |  |  | + |  | [3.A.1.13.2](http://tcdb.org/search/result.php?tc=3.A.1.13.2) | -63 | A9WCZ7 | Vitamins | Cobalamin |
| [WP_004296696.1](file:///Volumes/ESD-USB/Summer%202018/Hassan%20Submission%20PLosOne/content.html#lcl|NZ_CP012938.1_prot_WP_004296696.1_3182) |  |  |  |  |  | + |  | [3.A.1.13.2](http://tcdb.org/search/result.php?tc=3.A.1.13.2) | -59 | A9WCZ7 | Vitamins | Cobalamin |
| [WP_011965427.1](file:///Volumes/ESD-USB/Summer%202018/Library/Application%20Support/Microsoft/Office/Office%202011%20AutoRecovery/content.html#lcl|NC_009614.1_prot_WP_011965427.1_2022) |  |  |  |  |  |  | + | [3.A.1.13.2](http://tcdb.org/search/result.php?tc=3.A.1.13.2) | -80 | A9WCZ6 | Vitamins | Cobalamin |
| [WP_012055535.1](file:///Volumes/ESD-USB/Summer%202018/Library/Application%20Support/Microsoft/Office/Office%202011%20AutoRecovery/content.html#lcl|NC_009614.1_prot_WP_012055535.1_3022) |  |  |  |  |  |  | + | [3.A.1.13.2](http://tcdb.org/search/result.php?tc=3.A.1.13.2) | -72 | A9WCZ7 | Vitamins | Cobalamin |
| [WP_011965426.1](file:///Volumes/ESD-USB/Summer%202018/Library/Application%20Support/Microsoft/Office/Office%202011%20AutoRecovery/content.html#lcl|NC_009614.1_prot_WP_011965426.1_2021) |  |  |  |  |  |  | + | [3.A.1.13.2](http://tcdb.org/search/result.php?tc=3.A.1.13.2) | -71 | A9WCZ7 | Vitamins | Cobalamin |
| WP_008761546.1 |  |  |  | + |  |  |  | 3.A.1.14.1 | -24 | P15031 | Unknown | Unknown |
| WP_032813763.1 |  |  |  | + |  |  |  | 3.A.1.14.3 | -09 | P07821 | Siderophores | Hydroxamate |
| [WP_004307944.1](file:///Volumes/ESD-USB/Summer%202018/Hassan%20Submission%20PLosOne/content.html#lcl|NZ_CP012938.1_prot_WP_004307944.1_2167) |  |  |  |  |  | + |  | [3.A.1.15.4](http://tcdb.org/search/result.php?tc=3.A.1.15.4) | -36 | Q56953 | Cations | Fe^2+/^Mn^2+^ |
| [WP_007211545.1](file:///Volumes/ESD-USB/Summer%202018/Hassan%20Submission%20PLosOne/content.html#lcl|NZ_CP012801.1_prot_WP_007211545.1_2797) |  |  | + |  |  |  |  | [3.A.1.15.6](http://tcdb.org/search/result.php?tc=3.A.1.15.6) | -25 | P0A4G4 | Cations | Fe^2+^/Zn^2+^ |
| WP_011108714.1 |  |  |  | + |  |  |  | 3.A.1.15.6 | -25 | P0A4G4 | Cations | Zn^2+^/Fe^3+^ |
| YP_097362.1 |  |  |  |  | + |  |  | 3.A.1.15.8 | -37 | P96117 | Cations | Mn^2+^/Zn^2+^/Fe^2+^ |
| [WP_004300133.1](file:///Volumes/ESD-USB/Summer%202018/Hassan%20Submission%20PLosOne/content.html#lcl|NZ_CP012938.1_prot_WP_004300133.1_1194) |  |  |  |  |  | + |  | [3.A.1.15.11](http://tcdb.org/search/result.php?tc=3.A.1.15.11) | -25 | O34966 | Cations | Zn^2+^ |
| ABI39_06020 | **+** |  |  |  |  |  |  | 3.A.1.15.12 | -41 | O84071 | Cations | Fe^2+^ |
| [WP_029426419.1](file:///Volumes/ESD-USB/Summer%202018/Hassan%20Submission%20PLosOne/content.html#lcl|NZ_CP012801.1_prot_WP_029426419.1_2796) |  |  | + |  |  |  |  | [3.A.1.15.12](http://tcdb.org/search/result.php?tc=3.A.1.15.12) | -37 | O84071 | Nucleotides |  |
| WP_062696006.1 |  |  |  | + |  |  |  | 3.A.1.15.12 | -35 | O84071 | Unknown | Unknown |
| [WP_029427287.1](file:///Volumes/ESD-USB/Summer%202018/Hassan%20Submission%20PLosOne/content.html#lcl|NZ_CP012801.1_prot_WP_029427287.1_3694) |  |  | + |  |  |  |  | [3.A.1.15.13](http://tcdb.org/search/result.php?tc=3.A.1.15.13) | -39 | B2IWT1 | Cations | Zn^2+^ |
| WP_008763988.1 |  |  |  | + |  |  |  | 3.A.1.15.13 | -34 | B2IWT1 | Unknown | Unknown |
| WP_004303643.1 |  |  |  |  |  | + |  | [3.A.1.15.13](http://tcdb.org/search/result.php?tc=3.A.1.15.13) | -35 | B2IWT1 | Cations | Zn^2+^ |
| ABI39_06025 | **+** |  |  |  |  |  |  | 3.A.1.15.14 | -24 | P0A4G2 | Cations | Mn^2+^ |
| [WP_013618403.1](file:///Volumes/ESD-USB/Summer%202018/Hassan%20Submission%20PLosOne/content.html#lcl|NC_015164.1_prot_WP_013618403.1_2397) |  | + |  |  |  |  |  | [3.A.1.15.14](http://tcdb.org/search/result.php?tc=3.A.1.15.14) | -34 | P0A4G2 | Cations | Mn^2+^ |
| YP_098006.1 |  |  |  |  | + |  |  | 3.A.1.15.14 | -35 | Q2MGF9 | Cations | Mn^2+^ |
| [WP_041584488.1](file:///Volumes/ESD-USB/Summer%202018/Hassan%20Submission%20PLosOne/content.html#lcl|NC_015164.1_prot_WP_041584488.1_3300) |  | + |  |  |  |  |  | [3.A.1.15.15](http://tcdb.org/search/result.php?tc=3.A.1.15.15) | -37 | G0LRW8 | Cations | Mn^2+^ |
| [WP_041584488.1](file:///Volumes/ESD-USB/Summer%202018/Hassan%20Submission%20PLosOne/content.html#lcl|NC_015164.1_prot_WP_041584488.1_3300) |  | + |  |  |  |  |  | [3.A.1.15.15](http://tcdb.org/search/result.php?tc=3.A.1.15.15) | -37 | G0LRW8 | Cations | Mn^2+^ |
| [WP_007213197.1](file:///Volumes/ESD-USB/Summer%202018/Hassan%20Submission%20PLosOne/content.html#lcl|NZ_CP012801.1_prot_WP_007213197.1_3655) |  |  | + |  |  |  |  | [3.A.1.17.14](http://tcdb.org/search/result.php?tc=3.A.1.17.14) | -18 | A9WGD2 | Vitamins | Riboflavin |
| [WP_029428842.1](file:///Volumes/ESD-USB/Summer%202018/Hassan%20Submission%20PLosOne/content.html#lcl|NZ_CP012801.1_prot_WP_029428842.1_4659) |  |  | + |  |  |  |  | [3.A.1.21.1](http://tcdb.org/search/result.php?tc=3.A.1.21.1) | -105 | Q9R7V3 | Cations | Fe^3+^ |
| [WP_029428843.1](file:///Volumes/ESD-USB/Summer%202018/Hassan%20Submission%20PLosOne/content.html#lcl|NZ_CP012801.1_prot_WP_029428843.1_4658) |  |  | + |  |  |  |  | [3.A.1.21.1](http://tcdb.org/search/result.php?tc=3.A.1.21.1) | -101 | Q9Z375 | Cations | Fe^3+^ |
| WP_029425494.1 |  |  |  | + |  |  |  | 3.A.1.21.1 | -82 | Q9Z375 | Unknown | Unknown |
| YP_099392.1 |  |  |  |  | + |  |  | 3.A.1.21.1 | -111 | Q9R7V3 | Cations | Fe^3+^ |
| YP_099393.1 |  |  |  |  | + |  |  | 3.A.1.21.1 | -96 | Q9R7V3 | Cations | Fe^3+^ |
| [WP_004297229.1](file:///Volumes/ESD-USB/Summer%202018/Hassan%20Submission%20PLosOne/content.html#lcl|NZ_CP012938.1_prot_WP_004297229.1_3850) |  |  |  |  |  | + |  | [3.A.1.21.1](http://tcdb.org/search/result.php?tc=3.A.1.21.1) | -109 | Q9R7V3 | Siderophores | Yersinibactin |
| [WP_029428331.1](file:///Volumes/ESD-USB/Summer%202018/Hassan%20Submission%20PLosOne/content.html#lcl|NZ_CP012801.1_prot_WP_029428331.1_1197) |  |  | + |  |  |  |  | [3.A.1.26.5](http://tcdb.org/search/result.php?tc=3.A.1.26.5) | -22 | A7NRG0 | Vitamins | Thiamine |
| [WP_007210210.1](file:///Volumes/ESD-USB/Summer%202018/Hassan%20Submission%20PLosOne/content.html#lcl|NZ_CP012801.1_prot_WP_007210210.1_1817) |  |  | + |  |  |  |  | [3.A.1.27.2](http://tcdb.org/search/result.php?tc=3.A.1.27.2) | -26 | Q8L4R0 | Unknown | Unknown |
| [WP_007210209.1](file:///Volumes/ESD-USB/Summer%202018/Hassan%20Submission%20PLosOne/content.html#lcl|NZ_CP012801.1_prot_WP_007210209.1_1818) |  |  | + |  |  |  |  | [3.A.1.27.4](http://tcdb.org/search/result.php?tc=3.A.1.27.4) | -52 | Q0SD37 | Lipids | Cholesterol |
| WP_008764109.1 |  |  |  | + |  |  |  | 3.A.1.27.4 | -52 | Q0SD37 | Lipids | Cholesterol |
| [WP_005840227.1](file:///Volumes/ESD-USB/Summer%202018/Library/Application%20Support/Microsoft/Office/Office%202011%20AutoRecovery/content.html#lcl|NC_009614.1_prot_WP_005840227.1_447) |  |  |  |  |  |  | + | [3.A.1.27.4](http://tcdb.org/search/result.php?tc=3.A.1.27.4) | -51 | Q0SD37 | Unknown | Unknown |
| WP_062694689.1 |  |  |  | + |  |  |  | 3.A.1.32.3 | -11 | A9WBR9 | Vitamins | Cobalamin |
| ABI39_13670 | **+** |  |  |  |  |  |  | 3.A.1.103.5 | -46 | P72880 | Sugars | Carbohydrates |
| ABI39_20530 | **+** |  |  |  |  |  |  | 3.A.1.103.5 | -42 | P72880 | Sugars | Carbohydrates |
| [WP_005843133.1](file:///Volumes/ESD-USB/Summer%202018/Library/Application%20Support/Microsoft/Office/Office%202011%20AutoRecovery/content.html#lcl|NC_009614.1_prot_WP_005843133.1_3871) |  |  |  |  |  |  | + | [3.A.1.103.5](http://tcdb.org/search/result.php?tc=3.A.1.103.5) | -96 | P72884 | Sugars | Polysaccharides |
| [WP_005843132.1](file:///Volumes/ESD-USB/Summer%202018/Library/Application%20Support/Microsoft/Office/Office%202011%20AutoRecovery/content.html#lcl|NC_009614.1_prot_WP_005843132.1_3872) |  |  |  |  |  |  | + | [3.A.1.103.5](http://tcdb.org/search/result.php?tc=3.A.1.103.5) | -40 | P72880 | Sugars | Polysaccharides |
| ABI39_17370 | **+** |  |  |  |  |  |  | 3.A.1.105.4 | -56 | Q4VWC7 | Drugs | Pyoluteorin |
| [WP_013618570.1](file:///Volumes/ESD-USB/Summer%202018/Hassan%20Submission%20PLosOne/content.html#lcl|NC_015164.1_prot_WP_013618570.1_2615) |  | + |  |  |  |  |  | [3.A.1.105.4](http://tcdb.org/search/result.php?tc=3.A.1.105.4) | -14 | Q4VWC7 | Drugs | Pyoluteorin |
| [WP_029428180.1](file:///Volumes/ESD-USB/Summer%202018/Hassan%20Submission%20PLosOne/content.html#lcl|NZ_CP012801.1_prot_WP_029428180.1_5055) |  |  | + |  |  |  |  | [3.A.1.105.4](http://tcdb.org/search/result.php?tc=3.A.1.105.4) | -21 | Q4VWD0 | Secondary metabolites | Pyoluteorin |
| [WP_025725882.1](file:///Volumes/ESD-USB/Summer%202018/Hassan%20Submission%20PLosOne/content.html#lcl|NZ_CP012801.1_prot_WP_025725882.1_1722) |  |  | + |  |  |  |  | [3.A.1.105.4](http://tcdb.org/search/result.php?tc=3.A.1.105.4) | -17 | Q4VWC7 | Secondary metabolites | Pyoluteorin |
| WP_062694913.1 |  |  |  | + |  |  |  | 3.A.1.105.4 | -57 | Q4VWC8 | Secondary metabolites | Pyoluteorin |
| WP_011109012.1 |  |  |  | + |  |  |  | 3.A.1.105.4 | -18 | Q4VWC7 | Secondary metabolites | Pyoluteorin |
| YP_099912.1 |  |  |  |  | + |  |  | 3.A.1.105.4 | -58 | Q4VWC8 | Secondary metabolite | Pyoluteorin |
| YP_101263.1 |  |  |  |  | + |  |  | 3.A.1.105.4 | -15 | Q4VWC7 | Secondary metabolite | Pyoluteorin |
| [WP_004296002.1](file:///Volumes/ESD-USB/Summer%202018/Hassan%20Submission%20PLosOne/content.html#lcl|NZ_CP012938.1_prot_WP_004296002.1_684) |  |  |  |  |  | + |  | [3.A.1.105.4](http://tcdb.org/search/result.php?tc=3.A.1.105.4) | -18 | Q4VWD0 | Drugs | Pyoluteorin |
| [WP_004303641.1](file:///Volumes/ESD-USB/Summer%202018/Hassan%20Submission%20PLosOne/content.html#lcl|NZ_CP012938.1_prot_WP_004303641.1_1115) |  |  |  |  |  | + |  | [3.A.1.105.4](http://tcdb.org/search/result.php?tc=3.A.1.105.4) | -17 | Q4VWC7 | Drugs | Pyoluteorin |
| [WP_004297231.1](file:///Volumes/ESD-USB/Summer%202018/Hassan%20Submission%20PLosOne/content.html#lcl|NZ_CP012938.1_prot_WP_004297231.1_3852) |  |  |  |  |  | + |  | [3.A.1.105.4](http://tcdb.org/search/result.php?tc=3.A.1.105.4) | -13 | Q4VWC7 | Drugs | Pyoluteorin |
| [WP_005842065.1](file:///Volumes/ESD-USB/Summer%202018/Library/Application%20Support/Microsoft/Office/Office%202011%20AutoRecovery/content.html#lcl|NC_009614.1_prot_WP_005842065.1_3262) |  |  |  |  |  |  | + | [3.A.1.105.4](http://tcdb.org/search/result.php?tc=3.A.1.105.4) | -40 | Q4VWC7 | Drugs | Pyoluteorin |
| [WP_005853065.1](file:///Volumes/ESD-USB/Summer%202018/Library/Application%20Support/Microsoft/Office/Office%202011%20AutoRecovery/content.html#lcl|NC_009614.1_prot_WP_005853065.1_257) |  |  |  |  |  |  | + | [3.A.1.105.4](http://tcdb.org/search/result.php?tc=3.A.1.105.4) | -13 | Q4VWC7 | Drugs | Pyoluteorin |
| ABI39_01380 | **+** |  |  |  |  |  |  | 3.A.1.105.9 | -13 | J7ZHK9 | Unknown | Unknown |
| ABI39_17365 | **+** |  |  |  |  |  |  | 3.A.1.105.15 | -121 | P0A9U1 | Drugs | Drugs |
| ABI39_17375 | **+** |  |  |  |  |  |  | 3.A.1.105.15 | -50 | P0AFP9 | Drugs | Drugs |
| ABI39_01370 | **+** |  |  |  |  |  |  | 3.A.1.105.15 | -16 | P75777 | Drugs | Drugs |
| [WP_013616806.1](file:///Volumes/ESD-USB/Summer%202018/Hassan%20Submission%20PLosOne/content.html#lcl|NC_015164.1_prot_WP_013616806.1_746) |  | + |  |  |  |  |  | [3.A.1.105.15](http://tcdb.org/search/result.php?tc=3.A.1.105.15) | -57 | P0A9U1 | Unknown | Unknown |
| [WP_013616803.1](file:///Volumes/ESD-USB/Summer%202018/Hassan%20Submission%20PLosOne/content.html#lcl|NC_015164.1_prot_WP_013616803.1_743) |  | + |  |  |  |  |  | [3.A.1.105.15](http://tcdb.org/search/result.php?tc=3.A.1.105.15) | -56 | C6EIY9 | Unknown | Unknown |
| [WP_013616802.1](file:///Volumes/ESD-USB/Summer%202018/Hassan%20Submission%20PLosOne/content.html#lcl|NC_015164.1_prot_WP_013616802.1_742) |  | + |  |  |  |  |  | [3.A.1.105.15](http://tcdb.org/search/result.php?tc=3.A.1.105.15) | -48 | P0AFP9 | Drugs | Drugs |
| [WP_029428177.1](file:///Volumes/ESD-USB/Summer%202018/Hassan%20Submission%20PLosOne/content.html#lcl|NZ_CP012801.1_prot_WP_029428177.1_5058) |  |  | + |  |  |  |  | [3.A.1.105.15](http://tcdb.org/search/result.php?tc=3.A.1.105.15) | -50 | P0AFP9 | Unknown | Unknown |
| YP_099911.1 |  |  |  |  | + |  |  | 3.A.1.105.15 | -45 | P0AFP9 | Unknown | Unknown |
| YP_099782.1 |  |  |  |  | + |  |  | 3.A.1.105.15 | -11 | P0AFP9 | Unknown | Unknown |
| [WP_004296000.1](file:///Volumes/ESD-USB/Summer%202018/Hassan%20Submission%20PLosOne/content.html#lcl|NZ_CP012938.1_prot_WP_004296000.1_686) |  |  |  |  |  | + |  | [3.A.1.105.15](http://tcdb.org/search/result.php?tc=3.A.1.105.15) | -45 | P0AFP9 | Unknown | Unknown |
| ABI39_17925 | **+** |  |  |  |  |  |  | 3.A.1.105.16 | -18 | P37626 | Unknown | Unknown |
| ABI39_17360 | **+** |  |  |  |  |  |  | 3.A.1.105.16 | -17 | P37626 | Unknown | Unknown |
| ABI39_17920 | **+** |  |  |  |  |  |  | 3.A.1.105.16 | -07 | P0AGH1 | Unknown | Unknown |
| [WP_029428179.1](file:///Volumes/ESD-USB/Summer%202018/Hassan%20Submission%20PLosOne/content.html#lcl|NZ_CP012801.1_prot_WP_029428179.1_5056) |  |  | + |  |  |  |  | [3.A.1.105.16](http://tcdb.org/search/result.php?tc=3.A.1.105.16) | -117 | C8TJS4 | Unknown | Unknown |
| [WP_007210522.1](file:///Volumes/ESD-USB/Summer%202018/Hassan%20Submission%20PLosOne/content.html#lcl|NZ_CP012801.1_prot_WP_007210522.1_1720) |  |  | + |  |  |  |  | [3.A.1.105.16](http://tcdb.org/search/result.php?tc=3.A.1.105.16) | -18 | P37626 | Unknown | Unknown |
| [WP_007218328.1](file:///Volumes/ESD-USB/Summer%202018/Hassan%20Submission%20PLosOne/content.html#lcl|NZ_CP012801.1_prot_WP_007218328.1_325) |  |  | + |  |  |  |  | [3.A.1.105.16](http://tcdb.org/search/result.php?tc=3.A.1.105.16) | -16 | P37626 | Unknown | Unknown |
| WP_008764160.1 |  |  |  | + |  |  |  | 3.A.1.105.16 | -20 | P37626 | Unknown | Unknown |
| WP_011107544.1 |  |  |  | + |  |  |  | 3.A.1.105.16 | -17 | P37626 | Unknown | Unknown |
| YP_101261.1 |  |  |  |  | + |  |  | 3.A.1.105.16 | -17 | P37626 | Unknown | Unknown |
| YP_099781.1 |  |  |  |  | + |  |  | 3.A.1.105.16 | -16 | P37626 | Unknown | Unknown |
| [WP_004297231.1](file:///Volumes/ESD-USB/Summer%202018/Hassan%20Submission%20PLosOne/content.html#lcl|NZ_CP012938.1_prot_WP_004297231.1_3852) |  |  |  |  |  | + |  | [3.A.1.105.16](http://tcdb.org/search/result.php?tc=3.A.1.105.16) | -22 | P37626 | Unknown | Unknown |
| [WP_004296000.1](file:///Volumes/ESD-USB/Summer%202018/Hassan%20Submission%20PLosOne/content.html#lcl|NZ_CP012938.1_prot_WP_004296000.1_686) |  |  |  |  |  | + |  | [3.A.1.105.16](http://tcdb.org/search/result.php?tc=3.A.1.105.16) | -15 | P37626 | Unknown | Unknown |
| [WP_004318255.1](file:///Volumes/ESD-USB/Summer%202018/Hassan%20Submission%20PLosOne/content.html#lcl|NZ_CP012938.1_prot_WP_004318255.1_685) |  |  |  |  |  | + |  | [3.A.1.105.16](http://tcdb.org/search/result.php?tc=3.A.1.105.16) | -09 | P0AGH1 | Unknown | Unknown |
| [WP_005839262.1](file:///Volumes/ESD-USB/Summer%202018/Library/Application%20Support/Microsoft/Office/Office%202011%20AutoRecovery/content.html#lcl|NC_009614.1_prot_WP_005839262.1_3495) |  |  |  |  |  |  | + | [3.A.1.105.16](http://tcdb.org/search/result.php?tc=3.A.1.105.16) | -18 | P37626 | Unknown | Unknown |
| [WP_007216834.1](file:///Volumes/ESD-USB/Summer%202018/Hassan%20Submission%20PLosOne/content.html#lcl|NZ_CP012801.1_prot_WP_007216834.1_2322) |  |  | + |  |  |  |  | [3.A.1.106.1](http://tcdb.org/search/result.php?tc=3.A.1.106.1) | -104 | P60752 | Drugs | Azidopine, daunomycin, vinblastine |
| YP_097512.1 |  |  |  |  | + |  |  | 3.A.1.106.1 | -103 | P60752 | Lipids | Lipid A |
| [WP_004298275.1](file:///Volumes/ESD-USB/Summer%202018/Hassan%20Submission%20PLosOne/content.html#lcl|NZ_CP012938.1_prot_WP_004298275.1_3364) |  |  |  |  |  | + |  | [3.A.1.106.1](http://tcdb.org/search/result.php?tc=3.A.1.106.1) | -107 | P60752 | Lipids | Lipid A/glycerophospholipid |
| ABI39_06190 | **+** |  |  |  |  |  |  | 3.A.1.106.2 | -111 | Q2G2M9 | Drugs | Drugs |
| [WP_029427515.1](file:///Volumes/ESD-USB/Summer%202018/Hassan%20Submission%20PLosOne/content.html#lcl|NZ_CP012801.1_prot_WP_029427515.1_3388) |  |  | + |  |  |  |  | [3.A.1.106.12](http://tcdb.org/search/result.php?tc=3.A.1.106.12) | -54 | Q7NL26 | Lipids | Lipids |
| ABI39_21395 | **+** |  |  |  |  |  |  | 3.A.1.106.15 | -58 | Q0P9C4 | Unknown | Unknown |
| [WP_008782176.1](file:///Volumes/ESD-USB/Summer%202018/Library/Application%20Support/Microsoft/Office/Office%202011%20AutoRecovery/content.html#lcl|NC_009614.1_prot_WP_008782176.1_4055) |  |  |  |  |  |  | + | [3.A.1.106.15](http://tcdb.org/search/result.php?tc=3.A.1.106.15) | -57 | Q0P9C4 | Unknown | Unknown |
| ABI39_01560 | **+** |  |  |  |  |  |  | 3.A.1.112.9 | -121 | A3DCU1 | Peptides | Bacteriocins |
| [WP_029428680.1](file:///Volumes/ESD-USB/Summer%202018/Hassan%20Submission%20PLosOne/content.html#lcl|NZ_CP012801.1_prot_WP_029428680.1_4205) |  |  | + |  |  |  |  | [3.A.1.112.9](http://tcdb.org/search/result.php?tc=3.A.1.112.9) | -118 | A3DCU1 | Proteins | Bacteriocins |
| [WP_011964696.1](file:///Volumes/ESD-USB/Summer%202018/Library/Application%20Support/Microsoft/Office/Office%202011%20AutoRecovery/content.html#lcl|NC_009614.1_prot_WP_011964696.1_288) |  |  |  |  |  |  | + | [3.A.1.112.9](http://tcdb.org/search/result.php?tc=3.A.1.112.9) | -121 | A3DCU1 | Peptides | Bacteriocins |
| ABI39_05835 | + |  |  |  |  |  |  | 3.A.1.112.10 | 0 | F0R9C2 | Peptides | Bacteriocins |
| ABI39_04755 | + |  |  |  |  |  |  | 3.A.1.112.10 | -109 | F0R9C2 | Peptides | Bacteriocins |
| [WP_029428169.1](file:///Volumes/ESD-USB/Summer%202018/Hassan%20Submission%20PLosOne/content.html#lcl|NZ_CP012801.1_prot_WP_029428169.1_5069) |  |  | + |  |  |  |  | [3.A.1.112.10](http://tcdb.org/search/result.php?tc=3.A.1.112.10) | -130 | F0R9C2 | Proteins | Bacteriocins |
| [WP_029426164.1](file:///Volumes/ESD-USB/Summer%202018/Hassan%20Submission%20PLosOne/content.html#lcl|NZ_CP012801.1_prot_WP_029426164.1_2376) |  |  | + |  |  |  |  | [3.A.1.112.10](http://tcdb.org/search/result.php?tc=3.A.1.112.10) | -121 | F0R9C2 | Proteins | Bacteriocins |
| [WP_044128793.1](file:///Volumes/ESD-USB/Summer%202018/Hassan%20Submission%20PLosOne/content.html#lcl|NZ_CP012801.1_prot_WP_044128793.1_3374) |  |  | + |  |  |  |  | [3.A.1.112.10](http://tcdb.org/search/result.php?tc=3.A.1.112.10) | -109 | F0R9C2 | Proteins | Bacteriocins |
| [WP_033160369.1](file:///Volumes/ESD-USB/Summer%202018/Hassan%20Submission%20PLosOne/content.html#lcl|NZ_CP012801.1_prot_WP_033160369.1_2962) |  |  | + |  |  |  |  | [3.A.1.112.10](http://tcdb.org/search/result.php?tc=3.A.1.112.10) | -64 | F0R9C2 | Proteins | Bacteriocins |
| [WP_029427526.1](file:///Volumes/ESD-USB/Summer%202018/Hassan%20Submission%20PLosOne/content.html#lcl|NZ_CP012801.1_prot_WP_029427526.1_3372) |  |  | + |  |  |  |  | [3.A.1.112.10](http://tcdb.org/search/result.php?tc=3.A.1.112.10) | -05 | F0R9C2 | Unknown | Unknown |
| WP_008759963.1 |  |  |  | + |  |  |  | 3.A.1.112.10 | -110 | F0R9C2 | Peptides | Bacteriocins |
| YP_101315.1 |  |  |  |  | + |  |  | 3.A.1.112.10 | -123 | F0R9C2 | Peptides | Bacteriocins |
| [WP_022210703.1](file:///Volumes/ESD-USB/Summer%202018/Hassan%20Submission%20PLosOne/content.html#lcl|NZ_CP012801.1_prot_WP_022210703.1_986) |  |  | + |  |  |  |  | [3.A.1.120.6](http://tcdb.org/search/result.php?tc=3.A.1.120.6) | -94 | P43672 | Unknown | Unknown |
| [WP_060550560.1](file:///Volumes/ESD-USB/Summer%202018/Hassan%20Submission%20PLosOne/content.html#lcl|NZ_CP012801.1_prot_WP_060550560.1_611) |  |  | + |  |  |  |  | [3.A.1.120.6](http://tcdb.org/search/result.php?tc=3.A.1.120.6) | -88 | P43672 | Unknown | Unknown |
| [WP_029427508.1](file:///Volumes/ESD-USB/Summer%202018/Hassan%20Submission%20PLosOne/content.html#lcl|NZ_CP012801.1_prot_WP_029427508.1_3399) |  |  | + |  |  |  |  | [3.A.1.120.6](http://tcdb.org/search/result.php?tc=3.A.1.120.6) | -84 | P43672 | Unknown | Unknown |
| WP_022470075.1 |  |  |  | + |  |  |  | 3.A.1.120.6 | -95 | P43672 | Unknown | Unknown |
| WP_008763451.1 |  |  |  | + |  |  |  | 3.A.1.120.6 | -84 | P43672 | Unknown | Unknown |
| WP_011108986.1 |  |  |  | + |  |  |  | 3.A.1.120.6 | -83 | P43672 | Unknown | Unknown |
| [WP_029429126.1](file:///Volumes/ESD-USB/Summer%202018/Hassan%20Submission%20PLosOne/content.html#lcl|NZ_CP012801.1_prot_WP_029429126.1_4923) |  |  | + |  |  |  |  | [3.A.1.121.2](http://tcdb.org/search/result.php?tc=3.A.1.121.2) | -44 | O54396 | Drugs | Streptogramins |
| ABI39_05670 | **+** |  |  |  |  |  |  | 3.A.1.121.3 | -53 | P39115 | Drugs | Streptogramins/Lincosamide |
| YP_098775.1 |  |  |  |  | + |  |  | 3.A.1.121.3 | -48 | P39115 | Drugs | Lincosamides/Streptogramin |
| [WP_008666477.1](file:///Volumes/ESD-USB/Summer%202018/Library/Application%20Support/Microsoft/Office/Office%202011%20AutoRecovery/content.html#lcl|NC_009614.1_prot_WP_008666477.1_1143) |  |  |  |  |  |  | + | [3.A.1.121.3](http://tcdb.org/search/result.php?tc=3.A.1.121.3) | -53 | P39115 | Unknown | Unknown |
| ABI39_11670 | **+** |  |  |  |  |  |  | 3.A.1.121.4 | 0 | Q83XH1 | Drugs | Fluoroquinolones/Ethidium |
| ABI39_01640 | **+** |  |  |  |  |  |  | 3.A.1.121.4 | -88 | Q83XH1 | Drugs | Fluoroquinolones/Ethidium |
| [WP_013616956.1](file:///Volumes/ESD-USB/Summer%202018/Hassan%20Submission%20PLosOne/content.html#lcl|NC_015164.1_prot_WP_013616956.1_901) |  | + |  |  |  |  |  | [3.A.1.121.4](http://tcdb.org/search/result.php?tc=3.A.1.121.4) | 0 | Q83XH1 | Drugs | Fluoroquinolones/Ethidium |
| [WP_007213487.1](file:///Volumes/ESD-USB/Summer%202018/Hassan%20Submission%20PLosOne/content.html#lcl|NZ_CP012801.1_prot_WP_007213487.1_683) |  |  | + |  |  |  |  | [3.A.1.121.4](http://tcdb.org/search/result.php?tc=3.A.1.121.4) | 0 | Q83XH1 | Drugs | Fluoroquinolones/Ethidium |
| [WP_007210433.1](file:///Volumes/ESD-USB/Summer%202018/Hassan%20Submission%20PLosOne/content.html#lcl|NZ_CP012801.1_prot_WP_007210433.1_1427) |  |  | + |  |  |  |  | [3.A.1.121.4](http://tcdb.org/search/result.php?tc=3.A.1.121.4) | -90 | Q83XH1 | Drugs | Fluoroquinolones/Ethidium |
| WP_008763401.1 |  |  |  | + |  |  |  | 3.A.1.121.4 | 0 | Q83XH1 | Drugs | Fluoroquinoloes/Ethidium |
| WP_008761073.1 |  |  |  | + |  |  |  | 3.A.1.121.4 | -87 | Q83XH1 | Drugs | Fluoroquinoloes/Ethidium |
| WP_008767969.1 |  |  |  | + |  |  |  | 3.A.1.121.4 | -49 | Q83XH1 | Drugs | Fluoroquinoloes/Ethidium |
| YP_099025.1 |  |  |  |  | + |  |  | 3.A.1.121.4 | 0 | Q83XH1 | Drugs | Ethidium/Flouroquinolones |
| YP_100994.1 |  |  |  |  | + |  |  | 3.A.1.121.4 | -88 | Q83XH1 | Drugs | Ethidium/Flouroquinolones |
| [WP_004297887.1](file:///Volumes/ESD-USB/Summer%202018/Hassan%20Submission%20PLosOne/content.html#lcl|NZ_CP012938.1_prot_WP_004297887.1_312) |  |  |  |  |  | + |  | [3.A.1.121.4](http://tcdb.org/search/result.php?tc=3.A.1.121.4) | 0 | Q83XH1 | Drugs | Fluruoquinolones/Ethidium |
| [WP_004297971.1](file:///Volumes/ESD-USB/Summer%202018/Hassan%20Submission%20PLosOne/content.html#lcl|NZ_CP012938.1_prot_WP_004297971.1_1974) |  |  |  |  |  | + |  | [3.A.1.121.4](http://tcdb.org/search/result.php?tc=3.A.1.121.4) | -88 | Q83XH1 | Drugs | Fluruoquinolones/Ethidium |
| [WP_004299938.1](file:///Volumes/ESD-USB/Summer%202018/Hassan%20Submission%20PLosOne/content.html#lcl|NZ_CP012938.1_prot_WP_004299938.1_3773) |  |  |  |  |  | + |  | [3.A.1.121.4](http://tcdb.org/search/result.php?tc=3.A.1.121.4) | -83 | Q83XH1 | Drugs | Fluruoquinolones/Ethidium |
| [WP_004300722.1](file:///Volumes/ESD-USB/Summer%202018/Hassan%20Submission%20PLosOne/content.html#lcl|NZ_CP012938.1_prot_WP_004300722.1_1562) |  |  |  |  |  | + |  | [3.A.1.121.4](http://tcdb.org/search/result.php?tc=3.A.1.121.4) | -48 | Q83XH1 | Drugs | Fluruoquinolones/Ethidium |
| [WP_005846213.1](file:///Volumes/ESD-USB/Summer%202018/Library/Application%20Support/Microsoft/Office/Office%202011%20AutoRecovery/content.html#lcl|NC_009614.1_prot_WP_005846213.1_2282) |  |  |  |  |  |  | + | [3.A.1.121.4](http://tcdb.org/search/result.php?tc=3.A.1.121.4) | 0 | Q83XH1 | Drugs | Ethidium/Fluoroquinolones |
| [WP_005845690.1](file:///Volumes/ESD-USB/Summer%202018/Library/Application%20Support/Microsoft/Office/Office%202011%20AutoRecovery/content.html#lcl|NC_009614.1_prot_WP_005845690.1_305) |  |  |  |  |  |  | + | [3.A.1.121.4](http://tcdb.org/search/result.php?tc=3.A.1.121.4) | -89 | Q83XH1 | Drugs | Ethidium/Fluoroquinolones |
| ABI39_14380 | **+** |  |  |  |  |  |  | 3.A.1.122.1 | -78 | P75831 | Drugs | Macrolides |
| ABI39_01060 | **+** |  |  |  |  |  |  | 3.A.1.122.1 | -27 | P75831 | Drugs | Macrolides |
| ABI39_01055 | **+** |  |  |  |  |  |  | 3.A.1.122.1 | -22 | P75831 | Drugs | Macrolides |
| ABI39_11745 | **+** |  |  |  |  |  |  | 3.A.1.122.1 | -15 | P75831 | Drugs | Macrolides |
| ABI39_11755 | **+** |  |  |  |  |  |  | 3.A.1.122.1 | -14 | P75831 | Drugs | Macrolides |
| [WP_013618551.1](file:///Volumes/ESD-USB/Summer%202018/Hassan%20Submission%20PLosOne/content.html#lcl|NC_015164.1_prot_WP_013618551.1_2596) |  | + |  |  |  |  |  | [3.A.1.122.1](http://tcdb.org/search/result.php?tc=3.A.1.122.1) | -76 | P75831 | Drugs | Macrolides |
| [WP_013618959.1](file:///Volumes/ESD-USB/Summer%202018/Hassan%20Submission%20PLosOne/content.html#lcl|NC_015164.1_prot_WP_013618959.1_2999) |  | + |  |  |  |  |  | [3.A.1.122.1](http://tcdb.org/search/result.php?tc=3.A.1.122.1) | -26 | P75831 | Drugs | Macrolides |
| [WP_013618960.1](file:///Volumes/ESD-USB/Summer%202018/Hassan%20Submission%20PLosOne/content.html#lcl|NC_015164.1_prot_WP_013618960.1_3000) |  | + |  |  |  |  |  | [3.A.1.122.1](http://tcdb.org/search/result.php?tc=3.A.1.122.1) | -20 | P75831 | Drugs | Macrolides |
| [WP_013618958.1](file:///Volumes/ESD-USB/Summer%202018/Hassan%20Submission%20PLosOne/content.html#lcl|NC_015164.1_prot_WP_013618958.1_2998) |  | + |  |  |  |  |  | [3.A.1.122.1](http://tcdb.org/search/result.php?tc=3.A.1.122.1) | -14 | P75830 | Drugs | Macrolides |
| [WP_013618635.1](file:///Volumes/ESD-USB/Summer%202018/Hassan%20Submission%20PLosOne/content.html#lcl|NC_015164.1_prot_WP_013618635.1_2674) |  | + |  |  |  |  |  | [3.A.1.122.1](http://tcdb.org/search/result.php?tc=3.A.1.122.1) | -07 | P75831 | Drugs | Macrolides |
| [WP_013618945.1](file:///Volumes/ESD-USB/Summer%202018/Hassan%20Submission%20PLosOne/content.html#lcl|NC_015164.1_prot_WP_013618945.1_2983) |  | + |  |  |  |  |  | [3.A.1.122.1](http://tcdb.org/search/result.php?tc=3.A.1.122.1) | -07 | P75831 | Drugs | Macrolides |
| [WP_013618636.1](file:///Volumes/ESD-USB/Summer%202018/Hassan%20Submission%20PLosOne/content.html#lcl|NC_015164.1_prot_WP_013618636.1_2675) |  | + |  |  |  |  |  | [3.A.1.122.1](http://tcdb.org/search/result.php?tc=3.A.1.122.1) | -05 | P75831 | Drugs | Macrolides |
| [WP_029428918.1](file:///Volumes/ESD-USB/Summer%202018/Hassan%20Submission%20PLosOne/content.html#lcl|NZ_CP012801.1_prot_WP_029428918.1_630) |  |  | + |  |  |  |  | [3.A.1.122.1](http://tcdb.org/search/result.php?tc=3.A.1.122.1) | -72 | P75831 | Drugs | Macrolides |
| [WP_007211959.1](file:///Volumes/ESD-USB/Summer%202018/Hassan%20Submission%20PLosOne/content.html#lcl|NZ_CP012801.1_prot_WP_007211959.1_1946) |  |  | + |  |  |  |  | [3.A.1.122.1](http://tcdb.org/search/result.php?tc=3.A.1.122.1) | -23 | P75831 | Drugs | Macrolides |
| [WP_007209975.1](file:///Volumes/ESD-USB/Summer%202018/Hassan%20Submission%20PLosOne/content.html#lcl|NZ_CP012801.1_prot_WP_007209975.1_4948) |  |  | + |  |  |  |  | [3.A.1.122.1](http://tcdb.org/search/result.php?tc=3.A.1.122.1) | -15 | P75831 | Drugs | Macrolides |
| YP_099152.1 |  |  |  |  | + |  |  | 3.A.1.122.1 | -75 | P75831 | Drugs | Macrolides |
| YP_097843.1 |  |  |  |  | + |  |  | 3.A.1.122.1 | -22 | P75831 | Drugs | Macrolides |
| YP_098795.1 |  |  |  |  | + |  |  | 3.A.1.122.1 | -15 | P75831 | Drugs | Macrolides |
| YP_098795.1 |  |  |  |  | + |  |  | 3.A.1.122.1 | -15 | P75831 | Drugs | Macrolides |
| YP_098791.1 |  |  |  |  | + |  |  | 3.A.1.122.1 | -14 | P75831 | Drugs | Macrolides |
| YP_099655.1 |  |  |  |  | + |  |  | 3.A.1.122.1 | -11 | P75831 | Drugs | Macrolides |
| YP_098792.1 |  |  |  |  | + |  |  | 3.A.1.122.1 | -05 | P75831 | Drugs | Macrolides |
| YP_099326.1 |  |  |  |  | + |  |  | 3.A.1.122.1 | -05 | P75831 | Drugs | Macrolides |
| [WP_004300329.1](file:///Volumes/ESD-USB/Summer%202018/Hassan%20Submission%20PLosOne/content.html#lcl|NZ_CP012938.1_prot_WP_004300329.1_435) |  |  |  |  |  | + |  | [3.A.1.122.1](http://tcdb.org/search/result.php?tc=3.A.1.122.1) | -74 | P75831 | Drugs | Macrolides |
| [WP_004296203.1](file:///Volumes/ESD-USB/Summer%202018/Hassan%20Submission%20PLosOne/content.html#lcl|NZ_CP012938.1_prot_WP_004296203.1_2524) |  |  |  |  |  | + |  | [3.A.1.122.1](http://tcdb.org/search/result.php?tc=3.A.1.122.1) | -21 | P75831 | Drugs | Macrolides |
| [WP_004300701.1](file:///Volumes/ESD-USB/Summer%202018/Hassan%20Submission%20PLosOne/content.html#lcl|NZ_CP012938.1_prot_WP_004300701.1_1552) |  |  |  |  |  | + |  | [3.A.1.122.1](http://tcdb.org/search/result.php?tc=3.A.1.122.1) | -16 | P75831 | Drugs | Macrolides |
| [WP_013618552.1](file:///Volumes/ESD-USB/Summer%202018/Hassan%20Submission%20PLosOne/content.html#lcl|NC_015164.1_prot_WP_013618552.1_2597) |  | + |  |  |  |  |  | [3.A.1.122.2](http://tcdb.org/search/result.php?tc=3.A.1.122.2) | -79 | O31711 | Unknown | Unknown |
| [WP_013618961.1](file:///Volumes/ESD-USB/Summer%202018/Hassan%20Submission%20PLosOne/content.html#lcl|NC_015164.1_prot_WP_013618961.1_3001) |  | + |  |  |  |  |  | [3.A.1.122.2](http://tcdb.org/search/result.php?tc=3.A.1.122.2) | -74 | O31711 | Unknown | Unknown |
| [WP_029429115.1](file:///Volumes/ESD-USB/Summer%202018/Hassan%20Submission%20PLosOne/content.html#lcl|NZ_CP012801.1_prot_WP_029429115.1_4943) |  |  | + |  |  |  |  | [3.A.1.122.1](http://tcdb.org/search/result.php?tc=3.A.1.122.1) | -14 | P75831 | Drugs | Macrolides |
| [WP_029429117.1](file:///Volumes/ESD-USB/Summer%202018/Hassan%20Submission%20PLosOne/content.html#lcl|NZ_CP012801.1_prot_WP_029429117.1_4940) |  |  | + |  |  |  |  | [3.A.1.122.1](http://tcdb.org/search/result.php?tc=3.A.1.122.1) | -13 | P75831 | Drugs | Macrolides |
| [WP_029429113.1](file:///Volumes/ESD-USB/Summer%202018/Hassan%20Submission%20PLosOne/content.html#lcl|NZ_CP012801.1_prot_WP_029429113.1_4946) |  |  | + |  |  |  |  | [3.A.1.122.1](http://tcdb.org/search/result.php?tc=3.A.1.122.1) | -12 | P75831 | Drugs | Macrolides |
| WP_008765923.1 |  |  |  | + |  |  |  | 3.A.1.122.1 | -72 | P75831 | Drugs | Macrolides |
| WP_008763621.1 |  |  |  | + |  |  |  | 3.A.1.122.1 | -20 | P75831 | Drugs | Macrolides |
| WP_008763619.1 |  |  |  | + |  |  |  | 3.A.1.122.1 | -13 | P75830 | Drugs | Macrolides |
| [WP_005843410.1](file:///Volumes/ESD-USB/Summer%202018/Library/Application%20Support/Microsoft/Office/Office%202011%20AutoRecovery/content.html#lcl|NC_009614.1_prot_WP_005843410.1_2753) |  |  |  |  |  |  | + | [3.A.1.122.1](http://tcdb.org/search/result.php?tc=3.A.1.122.1) | -78 | P75831 | Drugs | Macrolides |
| [WP_005853705.1](file:///Volumes/ESD-USB/Summer%202018/Library/Application%20Support/Microsoft/Office/Office%202011%20AutoRecovery/content.html#lcl|NC_009614.1_prot_WP_005853705.1_196) |  |  |  |  |  |  | + | [3.A.1.122.1](http://tcdb.org/search/result.php?tc=3.A.1.122.1) | -27 | P75831 | Drugs | Macrolides |
| [WP_005853707.1](file:///Volumes/ESD-USB/Summer%202018/Library/Application%20Support/Microsoft/Office/Office%202011%20AutoRecovery/content.html#lcl|NC_009614.1_prot_WP_005853707.1_195) |  |  |  |  |  |  | + | [3.A.1.122.1](http://tcdb.org/search/result.php?tc=3.A.1.122.1) | -22 | P75831 | Drugs | Macrolides |
| [WP_005846246.1](file:///Volumes/ESD-USB/Summer%202018/Library/Application%20Support/Microsoft/Office/Office%202011%20AutoRecovery/content.html#lcl|NC_009614.1_prot_WP_005846246.1_2300) |  |  |  |  |  |  | + | [3.A.1.122.1](http://tcdb.org/search/result.php?tc=3.A.1.122.1) | -15 | P75831 | Drugs | Macrolides |
| [WP_011965567.1](file:///Volumes/ESD-USB/Summer%202018/Library/Application%20Support/Microsoft/Office/Office%202011%20AutoRecovery/content.html#lcl|NC_009614.1_prot_WP_011965567.1_2298) |  |  |  |  |  |  | + | [3.A.1.122.1](http://tcdb.org/search/result.php?tc=3.A.1.122.1) | -14 | P75831 | Drugs | Macrolides |
| ABI39_14375 | **+** |  |  |  |  |  |  | 3.A.1.122.2 | -81 | O31711 | Unknown | Unknown |
| ABI39_11730 | **+** |  |  |  |  |  |  | 3.A.1.122.2 | -77 | O31711 | Unknown | Unknown |
| ABI39_16545 | **+** |  |  |  |  |  |  | 3.A.1.122.2 | -75 | O31711 | Unknown | Unknown |
| ABI39_17510 | **+** |  |  |  |  |  |  | 3.A.1.122.2 | -75 | O31711 | Unknown | Unknown |
| ABI39_01050 | **+** |  |  |  |  |  |  | 3.A.1.122.2 | -74 | O31711 | Unknown | Unknown |
| ABI39_01095 | **+** |  |  |  |  |  |  | 3.A.1.122.2 | -67 | O31711 | Unknown | Unknown |
| ABI39_11735 | **+** |  |  |  |  |  |  | 3.A.1.122.2 | -15 | O31712 | Unknown | Unknown |
| ABI39_11765 | **+** |  |  |  |  |  |  | 3.A.1.122.2 | -12 | O31712 | Unknown | Unknown |
| [WP_007213581.1](file:///Volumes/ESD-USB/Summer%202018/Hassan%20Submission%20PLosOne/content.html#lcl|NZ_CP012801.1_prot_WP_007213581.1_629) |  |  | + |  |  |  |  | [3.A.1.122.2](http://tcdb.org/search/result.php?tc=3.A.1.122.2) | -80 | O31711 | Unknown | Unknown |
| [WP_007211960.1](file:///Volumes/ESD-USB/Summer%202018/Hassan%20Submission%20PLosOne/content.html#lcl|NZ_CP012801.1_prot_WP_007211960.1_1947) |  |  | + |  |  |  |  | [3.A.1.122.2](http://tcdb.org/search/result.php?tc=3.A.1.122.2) | -75 | O31711 | Unknown | Unknown |
| [WP_007209985.1](file:///Volumes/ESD-USB/Summer%202018/Hassan%20Submission%20PLosOne/content.html#lcl|NZ_CP012801.1_prot_WP_007209985.1_4942) |  |  | + |  |  |  |  | [3.A.1.122.2](http://tcdb.org/search/result.php?tc=3.A.1.122.2) | -75 | O31711 | Unknown | Unknown |
| [WP_007218531.1](file:///Volumes/ESD-USB/Summer%202018/Hassan%20Submission%20PLosOne/content.html#lcl|NZ_CP012801.1_prot_WP_007218531.1_5159) |  |  | + |  |  |  |  | [3.A.1.122.2](http://tcdb.org/search/result.php?tc=3.A.1.122.2) | -74 | O31711 | Unknown | Unknown |
| [WP_007218342.1](file:///Volumes/ESD-USB/Summer%202018/Hassan%20Submission%20PLosOne/content.html#lcl|NZ_CP012801.1_prot_WP_007218342.1_310) |  |  | + |  |  |  |  | [3.A.1.122.2](http://tcdb.org/search/result.php?tc=3.A.1.122.2) | -73 | O31711 | Unknown | Unknown |
| [WP_025725698.1](file:///Volumes/ESD-USB/Summer%202018/Hassan%20Submission%20PLosOne/content.html#lcl|NZ_CP012801.1_prot_WP_025725698.1_2097) |  |  | + |  |  |  |  | [3.A.1.122.2](http://tcdb.org/search/result.php?tc=3.A.1.122.2) | -71 | O31711 | Unknown | Unknown |
| [WP_029429116.1](file:///Volumes/ESD-USB/Summer%202018/Hassan%20Submission%20PLosOne/content.html#lcl|NZ_CP012801.1_prot_WP_029429116.1_4941) |  |  | + |  |  |  |  | [3.A.1.122.2](http://tcdb.org/search/result.php?tc=3.A.1.122.2) | -06 | O31712 | Unknown | Unknown |
| WP_008763426.1 |  |  |  | + |  |  |  | 3.A.1.122.2 | -81 | O31711 | Unknown | Unknown |
| WP_008761370.1 |  |  |  | + |  |  |  | 3.A.1.122.2 | -75 | O31711 | Unknown | Unknown |
| WP_008761529.1 |  |  |  | + |  |  |  | 3.A.1.122.2 | -75 | O31711 | Unknown | Unknown |
| WP_016267112.1 |  |  |  | + |  |  |  | 3.A.1.122.2 | -74 | O31711 | Unknown | Unknown |
| WP_008762585.1 |  |  |  | + |  |  |  | 3.A.1.122.2 | -70 | O31711 | Unknown | Unknown |
| WP_008762308.1 |  |  |  | + |  |  |  | 3.A.1.122.2 | -69 | O31711 | Unknown | Unknown |
| WP_008764773.1 |  |  |  | + |  |  |  | 3.A.1.122.2 | -15 | O31712 | Unknown | Unknown |
| WP_062695089.1 |  |  |  | + |  |  |  | 3.A.1.122.2 | -05 | O31712 | Unknown | Unknown |
| YP_099153.1 |  |  |  |  | + |  |  | 3.A.1.122.2 | -81 | O31711 | Unknown | Unknown |
| YP_098790.1 |  |  |  |  | + |  |  | 3.A.1.122.2 | -74 | O31711 | Unknown | Unknown |
| YP_099658.1 |  |  |  |  | + |  |  | 3.A.1.122.2 | -74 | O31711 | Unknown | Unknown |
| YP_098793.1 |  |  |  |  | + |  |  | 3.A.1.122.2 | -74 | O31711 | Unknown | Unknown |
| YP_099435.1 |  |  |  |  | + |  |  | 3.A.1.122.2 | -74 | O31711 | Unknown | Unknown |
| YP_097844.1 |  |  |  |  | + |  |  | 3.A.1.122.2 | -73 | O31711 | Unknown | Unknown |
| YP_097730.1 |  |  |  |  | + |  |  | 3.A.1.122.2 | -70 | O31711 | Unknown | Unknown |
| YP_099327.1 |  |  |  |  | + |  |  | 3.A.1.122.2 | -68 | O31711 | Unknown | Unknown |
| YP_098785.1 |  |  |  |  | + |  |  | 3.A.1.122.2 | -13 | O31712 | Unknown | Unknown |
| YP_098783.1 |  |  |  |  | + |  |  | 3.A.1.122.2 | -12 | O31712 | Unknown | Unknown |
| YP_098787.1 |  |  |  |  | + |  |  | 3.A.1.122.2 | -11 | O31712 | Unknown | Unknown |
| YP_099438.1 |  |  |  |  | + |  |  | 3.A.1.122.2 | -09 | O31712 | Unknown | Unknown |
| YP_098881.1 |  |  |  |  | + |  |  | 3.A.1.122.2 | -08 | O31712 | Unknown | Unknown |
| YP_098879.1 |  |  |  |  | + |  |  | 3.A.1.122.2 | -07 | O31712 | Unknown | Unknown |
| YP_0996541 |  |  |  |  | + |  |  | 3.A.1.122.2 | -07 | O31712 | Unknown | Unknown |
| YP_099436.1 |  |  |  |  | + |  |  | 3.A.1.122.2 | -06 | O31712 | Unknown | Unknown |
| YP_098784.1 |  |  |  |  | + |  |  | 3.A.1.122.2 | -06 | O31712 | Unknown | Unknown |
| YP_099657.1 |  |  |  |  | + |  |  | 3.A.1.122.2 | 0 | O31712 | Unknown | Unknown |
| YP_099653.1 |  |  |  |  | + |  |  | 3.A.1.122.2 | 0 | O31712 | Unknown | Unknown |
| [WP_004296202.1](file:///Volumes/ESD-USB/Summer%202018/Hassan%20Submission%20PLosOne/content.html#lcl|NZ_CP012938.1_prot_WP_004296202.1_2523) |  |  |  |  |  | + |  | [3.A.1.122.2](http://tcdb.org/search/result.php?tc=3.A.1.122.2) | -74 | O31711 | Unknown | Unknown |
| [WP_004295738.1](file:///Volumes/ESD-USB/Summer%202018/Hassan%20Submission%20PLosOne/content.html#lcl|NZ_CP012938.1_prot_WP_004295738.1_912) |  |  |  |  |  | + |  | [3.A.1.122.2](http://tcdb.org/search/result.php?tc=3.A.1.122.2) | -74 | O31711 | Unknown | Unknown |
| [WP_004295924.1](file:///Volumes/ESD-USB/Summer%202018/Hassan%20Submission%20PLosOne/content.html#lcl|NZ_CP012938.1_prot_WP_004295924.1_752) |  |  |  |  |  | + |  | [3.A.1.122.2](http://tcdb.org/search/result.php?tc=3.A.1.122.2) | -74 | O31711 | Unknown | Unknown |
| [WP_004322218.1](file:///Volumes/ESD-USB/Summer%202018/Hassan%20Submission%20PLosOne/content.html#lcl|NZ_CP012938.1_prot_WP_004322218.1_3605) |  |  |  |  |  | + |  | [3.A.1.122.2](http://tcdb.org/search/result.php?tc=3.A.1.122.2) | -71 | O31711 | Unknown | Unknown |
| [WP_004300698.1](file:///Volumes/ESD-USB/Summer%202018/Hassan%20Submission%20PLosOne/content.html#lcl|NZ_CP012938.1_prot_WP_004300698.1_1550) |  |  |  |  |  | + |  | [3.A.1.122.2](http://tcdb.org/search/result.php?tc=3.A.1.122.2) | -14 | O31712 | Unknown | Unknown |
| [WP_004295925.1](file:///Volumes/ESD-USB/Summer%202018/Hassan%20Submission%20PLosOne/content.html#lcl|NZ_CP012938.1_prot_WP_004295925.1_751) |  |  |  |  |  | + |  | [3.A.1.122.2](http://tcdb.org/search/result.php?tc=3.A.1.122.2) | -06 | O31712 | Unknown | Unknown |
| [WP_005843409.1](file:///Volumes/ESD-USB/Summer%202018/Library/Application%20Support/Microsoft/Office/Office%202011%20AutoRecovery/content.html#lcl|NC_009614.1_prot_WP_005843409.1_2752) |  |  |  |  |  |  | + | [3.A.1.122.2](http://tcdb.org/search/result.php?tc=3.A.1.122.2) | -81 | O31711 | Unknown | Unknown |
| [WP_032934722.1](file:///Volumes/ESD-USB/Summer%202018/Library/Application%20Support/Microsoft/Office/Office%202011%20AutoRecovery/content.html#lcl|NC_009614.1_prot_WP_032934722.1_2297) |  |  |  |  |  |  | + | [3.A.1.122.2](http://tcdb.org/search/result.php?tc=3.A.1.122.2) | -76 | O31711 | Unknown | Unknown |
| [WP_005840005.1](file:///Volumes/ESD-USB/Summer%202018/Library/Application%20Support/Microsoft/Office/Office%202011%20AutoRecovery/content.html#lcl|NC_009614.1_prot_WP_005840005.1_3366) |  |  |  |  |  |  | + | [3.A.1.122.2](http://tcdb.org/search/result.php?tc=3.A.1.122.2) | -75 | O31711 | Unknown | Unknown |
| [WP_005853399.1](file:///Volumes/ESD-USB/Summer%202018/Library/Application%20Support/Microsoft/Office/Office%202011%20AutoRecovery/content.html#lcl|NC_009614.1_prot_WP_005853399.1_3111) |  |  |  |  |  |  | + | [3.A.1.122.2](http://tcdb.org/search/result.php?tc=3.A.1.122.2) | -75 | O31711 | Unknown | Unknown |
| [WP_005843634.1](file:///Volumes/ESD-USB/Summer%202018/Library/Application%20Support/Microsoft/Office/Office%202011%20AutoRecovery/content.html#lcl|NC_009614.1_prot_WP_005843634.1_194) |  |  |  |  |  |  | + | [3.A.1.122.2](http://tcdb.org/search/result.php?tc=3.A.1.122.2) | -74 | O31711 | Unknown | Unknown |
| [WP_005846236.1](file:///Volumes/ESD-USB/Summer%202018/Library/Application%20Support/Microsoft/Office/Office%202011%20AutoRecovery/content.html#lcl|NC_009614.1_prot_WP_005846236.1_2295) |  |  |  |  |  |  | + | [3.A.1.122.2](http://tcdb.org/search/result.php?tc=3.A.1.122.2) | -13 | O31712 | Unknown | Unknown |
| [WP_005848549.1](file:///Volumes/ESD-USB/Summer%202018/Library/Application%20Support/Microsoft/Office/Office%202011%20AutoRecovery/content.html#lcl|NC_009614.1_prot_WP_005848549.1_2302) |  |  |  |  |  |  | + | [3.A.1.122.2](http://tcdb.org/search/result.php?tc=3.A.1.122.2) | -12 | O31712 | Unknown | Unknown |
| YP_098880.1 |  |  |  |  | + |  |  | 3.A.1.122.3 | -68 | Q8RKC1 | Unknown | Unknown |
| [WP_029429112.1](file:///Volumes/ESD-USB/Summer%202018/Hassan%20Submission%20PLosOne/content.html#lcl|NZ_CP012801.1_prot_WP_029429112.1_4949) |  |  | + |  |  |  |  | [3.A.1.122.3](http://tcdb.org/search/result.php?tc=3.A.1.122.3) | -05 | Q8RKC0 | Unknown | Unknown |
| [WP_004300611.1](file:///Volumes/ESD-USB/Summer%202018/Hassan%20Submission%20PLosOne/content.html#lcl|NZ_CP012938.1_prot_WP_004300611.1_1487) |  |  |  |  |  | + |  | [3.A.1.122.3](http://tcdb.org/search/result.php?tc=3.A.1.122.3) | -69 | Q8RKC1 | Peptides | Enterocin |
| [WP_005842427.1](file:///Volumes/ESD-USB/Summer%202018/Library/Application%20Support/Microsoft/Office/Office%202011%20AutoRecovery/content.html#lcl|NC_009614.1_prot_WP_005842427.1_3452) |  |  |  |  |  |  | + | [3.A.1.122.3](http://tcdb.org/search/result.php?tc=3.A.1.122.3) | -68 | Q8RKC1 | Peptides | Bacteriocins |
| ABI39_14370 | **+** |  |  |  |  |  |  | 3.A.1.122.12 |  | Q84BQ3 | Drugs | Arthrofactin |
| [WP_013618553.1](file:///Volumes/ESD-USB/Summer%202018/Hassan%20Submission%20PLosOne/content.html#lcl|NC_015164.1_prot_WP_013618553.1_2598) |  | + |  |  |  |  |  | [3.A.1.122.12](http://tcdb.org/search/result.php?tc=3.A.1.122.12) | -37 | Q84BQ3 | Drugs | Arthrofactin |
| [WP_007217864.1](file:///Volumes/ESD-USB/Summer%202018/Hassan%20Submission%20PLosOne/content.html#lcl|NZ_CP012801.1_prot_WP_007217864.1_628) |  |  | + |  |  |  |  | [3.A.1.122.12](http://tcdb.org/search/result.php?tc=3.A.1.122.12) | -35 | Q84BQ3 | Drugs | Arthrofactin |
| [WP_007211958.1](file:///Volumes/ESD-USB/Summer%202018/Hassan%20Submission%20PLosOne/content.html#lcl|NZ_CP012801.1_prot_WP_007211958.1_1945) |  |  | + |  |  |  |  | [3.A.1.122.12](http://tcdb.org/search/result.php?tc=3.A.1.122.12) | -26 | A0ZUB1 | Unknown | Unknown |
| WP_011107657.1 |  |  |  | + |  |  |  | 3.A.1.122.12 | -34 | Q84BQ3 | Peptides | Arthrofactin |
| WP_011108314.1 |  |  |  | + |  |  |  | 3.A.1.122.12 | -29 | A0ZUB1 | Drugs | Arthrofactin |
| WP_008762307.1 |  |  |  | + |  |  |  | 3.A.1.122.12 | -13 | Q84BQ3 | Peptides | Arthrofactin |
| WP_008764774.1 |  |  |  | + |  |  |  | 3.A.1.122.12 | -05 | A0ZUB1 | Peptides | Arthrofactin |
| YP_097842.1 |  |  |  |  | + |  |  | 3.A.1.122.12 | -30 | A0ZUB1 | Peptides | Arthrofactin |
| YP_097841.1 |  |  |  |  | + |  |  | 3.A.1.122.12 | -15 | Q84BQ3 | Peptides | Arthrofactin |
| [WP_004300331.1](file:///Volumes/ESD-USB/Summer%202018/Hassan%20Submission%20PLosOne/content.html#lcl|NZ_CP012938.1_prot_WP_004300331.1_437) |  |  |  |  |  | + |  | [3.A.1.122.12](http://tcdb.org/search/result.php?tc=3.A.1.122.12) | -36 | Q84BQ3 | Drugs | Arthrofactin |
| [WP_005843408.1](file:///Volumes/ESD-USB/Summer%202018/Library/Application%20Support/Microsoft/Office/Office%202011%20AutoRecovery/content.html#lcl|NC_009614.1_prot_WP_005843408.1_2751) |  |  |  |  |  |  | + | [3.A.1.122.12](http://tcdb.org/search/result.php?tc=3.A.1.122.12) | -30 | Q84BQ3 | Drugs | Arthrofactin |
| ABI39_11740 | **+** |  |  |  |  |  |  | 3.A.1.122.13 | -05 | Q03RZ6 | Unknown | Unknown |
| [WP_013618944.1](file:///Volumes/ESD-USB/Summer%202018/Hassan%20Submission%20PLosOne/content.html#lcl|NC_015164.1_prot_WP_013618944.1_2982) |  | + |  |  |  |  |  | [3.A.1.122.14](http://tcdb.org/search/result.php?tc=3.A.1.122.14) | -72 | Q58206 | Unknown | Unknown |
| [WP_007218374.1](file:///Volumes/ESD-USB/Summer%202018/Hassan%20Submission%20PLosOne/content.html#lcl|NZ_CP012801.1_prot_WP_007218374.1_63) |  |  | + |  |  |  |  | [3.A.1.122.14](http://tcdb.org/search/result.php?tc=3.A.1.122.14) | -72 | Q58206 | Nucleotides | Nucleotides |
| [WP_025725680.1](file:///Volumes/ESD-USB/Summer%202018/Hassan%20Submission%20PLosOne/content.html#lcl|NZ_CP012801.1_prot_WP_025725680.1_2129) |  |  | + |  |  |  |  | [3.A.1.122.14](http://tcdb.org/search/result.php?tc=3.A.1.122.14) | -72 | Q58206 | Nucleotides | Nucleotides |
| WP_008765150.1 |  |  |  | + |  |  |  | 3.A.1.122.14 | -72 | Q58206 | Unknown | Unknown |
| YP_099437.1 |  |  |  |  | + |  |  | 3.A.1.122.14 | -71 | Q58206 | Unknown | Unknown |
| [WP_004300704.1](file:///Volumes/ESD-USB/Summer%202018/Hassan%20Submission%20PLosOne/content.html#lcl|NZ_CP012938.1_prot_WP_004300704.1_1554) |  |  |  |  |  | + |  | [3.A.1.122.14](http://tcdb.org/search/result.php?tc=3.A.1.122.14) | -74 | Q58206 | Unknown | Unknown |
| [WP_011965566.1](file:///Volumes/ESD-USB/Summer%202018/Library/Application%20Support/Microsoft/Office/Office%202011%20AutoRecovery/content.html#lcl|NC_009614.1_prot_WP_011965566.1_2296) |  |  |  |  |  |  | + | [3.A.1.122.14](http://tcdb.org/search/result.php?tc=3.A.1.122.14) | -05 | Q58207 | Unknown | Unknown |
| ABI39_18135 | **+** |  |  |  |  |  |  | 3.A.1.122.16 | -29 | D6ZUW6 | Drugs | Macrolides |
| [WP_007212806.1](file:///Volumes/ESD-USB/Summer%202018/Hassan%20Submission%20PLosOne/content.html#lcl|NZ_CP012801.1_prot_WP_007212806.1_4143) |  |  | + |  |  |  |  | [3.A.1.122.16](http://tcdb.org/search/result.php?tc=3.A.1.122.16) | -28 | D6ZUW6 | Drugs | Macrolides |
| WP_032840608.1 |  |  |  | + |  |  |  | 3.A.1.122.16 | -22 | D6ZUW6 | Drugs | Macrolides |
| [WP_004301808.1](file:///Volumes/ESD-USB/Summer%202018/Hassan%20Submission%20PLosOne/content.html#lcl|NZ_CP012938.1_prot_WP_004301808.1_4381) |  |  |  |  |  | + |  | [3.A.1.122.16](http://tcdb.org/search/result.php?tc=3.A.1.122.16) | -24 | D6ZUW6 | Drugs | Macrolides |
| [WP_012055780.1](file:///Volumes/ESD-USB/Summer%202018/Library/Application%20Support/Microsoft/Office/Office%202011%20AutoRecovery/content.html#lcl|NC_009614.1_prot_WP_012055780.1_3524) |  |  |  |  |  |  | + | [3.A.1.122.16](http://tcdb.org/search/result.php?tc=3.A.1.122.16) | -31 | D6ZUW6 | Drugs | Macrolides |
| ABI39_17660 | **+** |  |  |  |  |  |  | 3.A.1.125.1 | -68 | P75957 | Unknown | Unknown |
| ABI39_07025 | **+** |  |  |  |  |  |  | 3.A.1.125.1 | -25 | P75958 | Proteins | Lipoproteins |
| [WP_013619217.1](file:///Volumes/ESD-USB/Summer%202018/Hassan%20Submission%20PLosOne/content.html#lcl|NC_015164.1_prot_WP_013619217.1_3272) |  | + |  |  |  |  |  | [3.A.1.125.1](http://tcdb.org/search/result.php?tc=3.A.1.125.1) | -66 | P75957 | Unknown | Unknown |
| [WP_029426523.1](file:///Volumes/ESD-USB/Summer%202018/Hassan%20Submission%20PLosOne/content.html#lcl|NZ_CP012801.1_prot_WP_029426523.1_2995) |  |  | + |  |  |  |  | [3.A.1.125.1](http://tcdb.org/search/result.php?tc=3.A.1.125.1) | -23 | P75958 | Unknown | Unknown |
| [WP_007209883.1](file:///Volumes/ESD-USB/Summer%202018/Hassan%20Submission%20PLosOne/content.html#lcl|NZ_CP012801.1_prot_WP_007209883.1_4953) |  |  | + |  |  |  |  | [3.A.1.125.1](http://tcdb.org/search/result.php?tc=3.A.1.125.1) | -16 | P75958 | Unknown | Unknown |
| WP_062694253.1 |  |  |  | + |  |  |  | 3.A.1.125.1 | -28 | P75958 | Unknown | Unknown |
| WP_008764820.1 |  |  |  | + |  |  |  | 3.A.1.125.1 | -18 | P75958 | Unknown | Unknown |
| YP_101749.1 |  |  |  |  | + |  |  | 3.A.1.125.1 | -23 | P75958 | Unknown | Unknown |
| YP_098872.1 |  |  |  |  | + |  |  | 3.A.1.125.1 | -21 | P75958 | Unknown | Unknown |
| [WP_004296521.1](file:///Volumes/ESD-USB/Summer%202018/Hassan%20Submission%20PLosOne/content.html#lcl|NZ_CP012938.1_prot_WP_004296521.1_2803) |  |  |  |  |  | + |  | [3.A.1.125.1](http://tcdb.org/search/result.php?tc=3.A.1.125.1) | -26 | P75958 | Proteins | Proteins |
| [WP_004300622.1](file:///Volumes/ESD-USB/Summer%202018/Hassan%20Submission%20PLosOne/content.html#lcl|NZ_CP012938.1_prot_WP_004300622.1_1496) |  |  |  |  |  | + |  | [3.A.1.125.1](http://tcdb.org/search/result.php?tc=3.A.1.125.1) | -16 | P75958 | Proteins | Proteins |
| [WP_005843614.1](file:///Volumes/ESD-USB/Summer%202018/Library/Application%20Support/Microsoft/Office/Office%202011%20AutoRecovery/content.html#lcl|NC_009614.1_prot_WP_005843614.1_204) |  |  |  |  |  |  | + | [3.A.1.125.1](http://tcdb.org/search/result.php?tc=3.A.1.125.1) | -67 | P75957 | Unknown | Unknown |
| [WP_005844488.1](file:///Volumes/ESD-USB/Summer%202018/Library/Application%20Support/Microsoft/Office/Office%202011%20AutoRecovery/content.html#lcl|NC_009614.1_prot_WP_005844488.1_856) |  |  |  |  |  |  | + | [3.A.1.125.1](http://tcdb.org/search/result.php?tc=3.A.1.125.1) | -25 | P75958 | Unknown | Unknown |
| ABI39_19980 | **+** |  |  |  |  |  |  | 3.A.1.125.5 | -14 | B1ZMT9 | Unknown | Unknown |
| [WP_005841546.1](file:///Volumes/ESD-USB/Summer%202018/Library/Application%20Support/Microsoft/Office/Office%202011%20AutoRecovery/content.html#lcl|NC_009614.1_prot_WP_005841546.1_3768) |  |  |  |  |  |  | + | [3.A.1.125.5](http://tcdb.org/search/result.php?tc=3.A.1.125.5) | -14 | B1ZMT9 | Unknown | Unknown |
| ABI39_18250 | **+** |  |  |  |  |  |  | 3.A.1.128.6 | -41 | O58953 | Unknown | Unknown |
| ABI39_21265 | **+** |  |  |  |  |  |  | 3.A.1.128.6 | -28 | O58953 | Unknown | Unknown |
| [WP_013616732.1](file:///Volumes/ESD-USB/Summer%202018/Hassan%20Submission%20PLosOne/content.html#lcl|NC_015164.1_prot_WP_013616732.1_669) |  | + |  |  |  |  |  | [3.A.1.128.6](http://tcdb.org/search/result.php?tc=3.A.1.128.6) | -37 | O58953 | Unknown | Unknown |
| WP_008759988.1 |  |  |  | + |  |  |  | 3.A.1.128.6 | -39 | O58953 | Unknown | Unknown |
| YP_098300.1 |  |  |  |  | + |  |  | 3.A.1.128.6 | -38 | O58953 | Unknown | Unknown |
| [WP_004301798.1](file:///Volumes/ESD-USB/Summer%202018/Hassan%20Submission%20PLosOne/content.html#lcl|NZ_CP012938.1_prot_WP_004301798.1_4373) |  |  |  |  |  | + |  | [3.A.1.128.6](http://tcdb.org/search/result.php?tc=3.A.1.128.6) | -41 | O58953 | Unknown | Unknown |
| [WP_005839367.1](file:///Volumes/ESD-USB/Summer%202018/Library/Application%20Support/Microsoft/Office/Office%202011%20AutoRecovery/content.html#lcl|NC_009614.1_prot_WP_005839367.1_3545) |  |  |  |  |  |  | + | [3.A.1.128.6](http://tcdb.org/search/result.php?tc=3.A.1.128.6) | -41 | O58953 | Unknown | Unknown |
| ABI39_12535 | **+** |  |  |  |  |  |  | 3.A.1.131.1 | -35 | P42332 | Drugs | Bacitracin |
| [WP_007214173.1](file:///Volumes/ESD-USB/Summer%202018/Hassan%20Submission%20PLosOne/content.html#lcl|NZ_CP012801.1_prot_WP_007214173.1_5174) |  |  | + |  |  |  |  | [3.A.1.131.1](http://tcdb.org/search/result.php?tc=3.A.1.131.1) | -60 | P42332 | Drugs | Bacitracin |
| WP_011107522.1 |  |  |  | + |  |  |  | 3.A.1.131.1 | -62 | P42332 | Drugs | Bacitracin |
| [WP_007209583.1](file:///Volumes/ESD-USB/Summer%202018/Hassan%20Submission%20PLosOne/content.html#lcl|NZ_CP012801.1_prot_WP_007209583.1_3357) |  |  | + |  |  |  |  | [3.A.1.132.1](http://tcdb.org/search/result.php?tc=3.A.1.132.1) | -34 | O30489 | Unknown | Unknown |
| WP_008767082.1 |  |  |  | + |  |  |  | 3.A.1.132.1 | -34 | O30489 | Unknown | Unknown |
| YP_100503.1 |  |  |  |  | + |  |  | 3.A.1.131.1 | -34 | P42332 | Unknown | Unknown |
| [WP_004295990.1](file:///Volumes/ESD-USB/Summer%202018/Hassan%20Submission%20PLosOne/content.html#lcl|NZ_CP012938.1_prot_WP_004295990.1_693) |  |  |  |  |  | + |  | [3.A.1.131.1](http://tcdb.org/search/result.php?tc=3.A.1.131.1) | -65 | P42332 | Drugs | Bacitracin |
| [WP_005847446.1](file:///Volumes/ESD-USB/Summer%202018/Library/Application%20Support/Microsoft/Office/Office%202011%20AutoRecovery/content.html#lcl|NC_009614.1_prot_WP_005847446.1_2464) |  |  |  |  |  |  | + | [3.A.1.131.1](http://tcdb.org/search/result.php?tc=3.A.1.131.1) | -35 | P42332 | Drugs | Bacitracin |
| [WP_007214172.1](file:///Volumes/ESD-USB/Summer%202018/Hassan%20Submission%20PLosOne/content.html#lcl|NZ_CP012801.1_prot_WP_007214172.1_5175) |  |  | + |  |  |  |  | [3.A.1.132.2](http://tcdb.org/search/result.php?tc=3.A.1.132.2) | -09 | O07330 | Cations | Cu^2+^ |
| [WP_004295992.1](file:///Volumes/ESD-USB/Summer%202018/Hassan%20Submission%20PLosOne/content.html#lcl|NZ_CP012938.1_prot_WP_004295992.1_692) |  |  |  |  |  | + |  | [3.A.1.132.2](http://tcdb.org/search/result.php?tc=3.A.1.132.2) | -08 | O07330 | Unknown | Unknown |
| ABI39_19885 | **+** |  |  |  |  |  |  | 3.A.1.132.3 | -32 | A0L4L0 | Unknown | Unknown |
| ABI39_01400 | **+** |  |  |  |  |  |  | 3.A.1.132.3 | 0 | A0L4L0 | Unknown | Unknown |
| ABI39_19885 | + |  |  |  |  |  |  | 3.A.1.132.3 | 0 | A0L4L0 | Unknown | Unknown |
| [WP_013619209.1](file:///Volumes/ESD-USB/Summer%202018/Hassan%20Submission%20PLosOne/content.html#lcl|NC_015164.1_prot_WP_013619209.1_3263) |  | + |  |  |  |  |  | [3.A.1.132.3](http://tcdb.org/search/result.php?tc=3.A.1.132.3) | -59 | A0L4L0 | Unknown | Unknown |
| [WP_007218763.1](file:///Volumes/ESD-USB/Summer%202018/Hassan%20Submission%20PLosOne/content.html#lcl|NZ_CP012801.1_prot_WP_007218763.1_1728) |  |  | + |  |  |  |  | [3.A.1.132.3](http://tcdb.org/search/result.php?tc=3.A.1.132.3) | -59 | A0L4L0 | Unknown | Unknown |
| WP_062694342.1 |  |  |  | + |  |  |  | 3.A.1.132.3 | -57 | A0L4L0 | Unknown | Unknown |
| YP_101270.1 |  |  |  |  | + |  |  | 3.A.1.132.3 | -58 | A0L4L0 | Unknown | Unknown |
| [WP_004297220.1](file:///Volumes/ESD-USB/Summer%202018/Hassan%20Submission%20PLosOne/content.html#lcl|NZ_CP012938.1_prot_WP_004297220.1_3844) |  |  |  |  |  | + |  | [3.A.1.132.3](http://tcdb.org/search/result.php?tc=3.A.1.132.3) | -58 | A0L4L0 | Unknown | Unknown |
| [WP_011964682.1](file:///Volumes/ESD-USB/Summer%202018/Library/Application%20Support/Microsoft/Office/Office%202011%20AutoRecovery/content.html#lcl|NC_009614.1_prot_WP_011964682.1_261) |  |  |  |  |  |  | + | [3.A.1.132.3](http://tcdb.org/search/result.php?tc=3.A.1.132.3) | -60 | A0L4L0 | Unknown | Unknown |
| [WP_004299701.1](file:///Volumes/ESD-USB/Summer%202018/Hassan%20Submission%20PLosOne/content.html#lcl|NZ_CP012938.1_prot_WP_004299701.1_3595) |  |  |  |  |  | + |  | [3.A.1.132.4](http://tcdb.org/search/result.php?tc=3.A.1.132.4) | -34 | Q2SDB1 | Unknown | Unknown |
| ABI39_08015 | **+** |  |  |  |  |  |  | 3.A.1.132.6 | -47 | Q99ZC8 | Unknown | Unknown |
| [WP_007209416.1](file:///Volumes/ESD-USB/Summer%202018/Hassan%20Submission%20PLosOne/content.html#lcl|NZ_CP012801.1_prot_WP_007209416.1_3335) |  |  | + |  |  |  |  | [3.A.1.132.6](http://tcdb.org/search/result.php?tc=3.A.1.132.6) | -28 | Q99ZC8 | Unknown | Unknown |
| [WP_022208698.1](file:///Volumes/ESD-USB/Summer%202018/Hassan%20Submission%20PLosOne/content.html#lcl|NZ_CP012801.1_prot_WP_022208698.1_557) |  |  | + |  |  |  |  | [3.A.1.132.6](http://tcdb.org/search/result.php?tc=3.A.1.132.6) | -28 | Q99ZC8 | Unknown | Unknown |
| WP_008764965.1 |  |  |  | + |  |  |  | 3.A.1.132.6 | -27 | Q99ZC8 | Unknown | Unknown |
| [WP_004296281.1](file:///Volumes/ESD-USB/Summer%202018/Hassan%20Submission%20PLosOne/content.html#lcl|NZ_CP012938.1_prot_WP_004296281.1_2591) |  |  |  |  |  | + |  | [3.A.1.132.6](http://tcdb.org/search/result.php?tc=3.A.1.132.6) | -26 | Q99ZC8 | Unknown | Unknown |
| [WP_005839170.1](file:///Volumes/ESD-USB/Summer%202018/Library/Application%20Support/Microsoft/Office/Office%202011%20AutoRecovery/content.html#lcl|NC_009614.1_prot_WP_005839170.1_1605) |  |  |  |  |  |  | + | [3.A.1.132.6](http://tcdb.org/search/result.php?tc=3.A.1.132.6) | -30 | Q99ZC8 | Unknown | Unknown |
| [WP_005839170.1](file:///Volumes/ESD-USB/Summer%202018/Library/Application%20Support/Microsoft/Office/Office%202011%20AutoRecovery/content.html#lcl|NC_009614.1_prot_WP_005839170.1_1605) |  |  |  |  |  |  | + | [3.A.1.132.6](http://tcdb.org/search/result.php?tc=3.A.1.132.6) | -30 | Q99ZC8 | Unknown | Unknown |
| ABI39_17290 | **+** |  |  |  |  |  |  | 3.A.1.139.3 | -12 | H9UM46 | Unknown | Unknown |
| [WP_007214215.1](file:///Volumes/ESD-USB/Summer%202018/Hassan%20Submission%20PLosOne/content.html#lcl|NZ_CP012801.1_prot_WP_007214215.1_5139) |  |  | + |  |  |  |  | [3.A.1.139.3](http://tcdb.org/search/result.php?tc=3.A.1.139.3) | -33 | H9UM46 | Unknown | Unknown |
| WP_008761547.1 |  |  |  | + |  |  |  | 3.A.1.139.3 | -33 | H9UM46 | Unknown | Unknown |
| YP_099668.1 |  |  |  |  | + |  |  | 3.A.1.139.3 | -33 | H9UM46 | Unknown | Unknown |
| [WP_004295936.1](file:///Volumes/ESD-USB/Summer%202018/Hassan%20Submission%20PLosOne/content.html#lcl|NZ_CP012938.1_prot_WP_004295936.1_741) |  |  |  |  |  | + |  | [3.A.1.139.3](http://tcdb.org/search/result.php?tc=3.A.1.139.3) | -34 | H9UM46 | Unknown | Unknown |
| [WP_005842037.1](file:///Volumes/ESD-USB/Summer%202018/Library/Application%20Support/Microsoft/Office/Office%202011%20AutoRecovery/content.html#lcl|NC_009614.1_prot_WP_005842037.1_3248) |  |  |  |  |  |  | + | [3.A.1.139.3](http://tcdb.org/search/result.php?tc=3.A.1.139.3) | -31 | H9UM46 | Unknown | Unknown |
| ABI39_19940 | **+** |  |  |  |  |  |  | 3.A.1.140.1 | -16 | P0A9R7 | Unknown | Unknown |
| ABI39_05630 | **+** |  |  |  |  |  |  | 3.A.1.140.1 | 0 | P0AC31 | Unknown | Unknown |
| [WP_013616983.1](file:///Volumes/ESD-USB/Summer%202018/Hassan%20Submission%20PLosOne/content.html#lcl|NC_015164.1_prot_WP_013616983.1_931) |  | + |  |  |  |  |  | [3.A.1.140.1](http://tcdb.org/search/result.php?tc=3.A.1.140.1) | -47 | P0A9R7 | Unknown | Unknown |
| [WP_007209750.1](file:///Volumes/ESD-USB/Summer%202018/Hassan%20Submission%20PLosOne/content.html#lcl|NZ_CP012801.1_prot_WP_007209750.1_4832) |  |  | + |  |  |  |  | [3.A.1.140.1](http://tcdb.org/search/result.php?tc=3.A.1.140.1) | -47 | P0A9R7 | Unknown | Unknown |
| [WP_007216444.1](file:///Volumes/ESD-USB/Summer%202018/Hassan%20Submission%20PLosOne/content.html#lcl|NZ_CP012801.1_prot_WP_007216444.1_2739) |  |  | + |  |  |  |  | [3.A.1.140.1](http://tcdb.org/search/result.php?tc=3.A.1.140.1) | -12 | P0AC31 | Unknown | Unknown |
| WP_008762817.1 |  |  |  | + |  |  |  | 3.A.1.140.1 | -07 | P0AC31 | Unknown | Unknown |
| WP_008762817.1 |  |  |  | + |  |  |  | 3.A.1.140.1 | -07 | P0AC31 | Unknown | Unknown |
| YP_100331.1 |  |  |  |  | + |  |  | 3.A.1.140.1 | -45 | P0A9R7 | Unknown | Unknown |
| YP_097337.1 |  |  |  |  | + |  |  | 3.A.1.140.1 | -09 | P0AC31 | Unknown | Unknown |
| [WP_004297044.1](file:///Volumes/ESD-USB/Summer%202018/Hassan%20Submission%20PLosOne/content.html#lcl|NZ_CP012938.1_prot_WP_004297044.1_1352) |  |  |  |  |  | + |  | [3.A.1.140.1](http://tcdb.org/search/result.php?tc=3.A.1.140.1) | -44 | P0A9R7 | Unknown | Unknown |
| [WP_005841212.1](file:///Volumes/ESD-USB/Summer%202018/Library/Application%20Support/Microsoft/Office/Office%202011%20AutoRecovery/content.html#lcl|NC_009614.1_prot_WP_005841212.1_3759) |  |  |  |  |  |  | + | [3.A.1.140.1](http://tcdb.org/search/result.php?tc=3.A.1.140.1) | -47 | P0A9R7 | Unknown | Unknown |
| [WP_029428733.1](file:///Volumes/ESD-USB/Summer%202018/Hassan%20Submission%20PLosOne/content.html#lcl|NZ_CP012801.1_prot_WP_029428733.1_4099) |  |  | + |  |  |  |  | [3.A.1.147.2](http://tcdb.org/search/result.php?tc=3.A.1.147.2) | -37 | F8FLY7 | Unknown | Unknown |
| YP_101404.1 |  |  |  |  | + |  |  | 3.A.1.148.2 | -25 | R4ZCJ4 | Unknown | Unknown |
| WP_008760592.1 |  |  |  | + |  |  |  | 3.A.1.148.3 | -87 | D6TT54 | Unknown | Unknown |
| [WP_013616286.1](file:///Volumes/ESD-USB/Summer%202018/Hassan%20Submission%20PLosOne/content.html#lcl|NC_015164.1_prot_WP_013616286.1_209) |  | + |  |  |  |  |  | [3.A.2.1.1](http://tcdb.org/search/result.php?tc=3.A.2.1.1) | -11 | P0ABA0 | Cations | H^+^ |
| [WP_029427942.1](file:///Volumes/ESD-USB/Summer%202018/Hassan%20Submission%20PLosOne/content.html#lcl|NZ_CP012801.1_prot_WP_029427942.1_79) |  |  | + |  |  |  |  | [3.A.2.1.1](http://tcdb.org/search/result.php?tc=3.A.2.1.1) | -10 | P0ABA0 | Cations | H^+^ |
| WP_008761391.1 |  |  |  | + |  |  |  | 3.A.2.1.1 | -09 | P0ABA0 | Cations | H^+^ |
| YP_099457.1 |  |  |  |  | + |  |  | 3.A.2.1.1 | -09 | P0ABA0 | Cations | H^+^ |
| [WP_004295759.1](file:///Volumes/ESD-USB/Summer%202018/Hassan%20Submission%20PLosOne/content.html#lcl|NZ_CP012938.1_prot_WP_004295759.1_893) |  |  |  |  |  | + |  | [3.A.2.1.1](http://tcdb.org/search/result.php?tc=3.A.2.1.1) | -10 | P0ABA0 | Unknown | Unknown |
| [WP_005846830.1](file:///Volumes/ESD-USB/Summer%202018/Library/Application%20Support/Microsoft/Office/Office%202011%20AutoRecovery/content.html#lcl|NC_009614.1_prot_WP_005846830.1_2944) |  |  |  |  |  |  | + | [3.A.2.1.1](http://tcdb.org/search/result.php?tc=3.A.2.1.1) | -16 | P0ABA0 | Unknown | Unknown |
| ABI39_14955 | **+** |  |  |  |  |  |  | 3.A.2.1.2 | 0 | P29707 | Unknown | Unknown |
| ABI39_14995 | **+** |  |  |  |  |  |  | 3.A.2.1.2 | -12 | P29710 | Cations | Na^+^ |
| [WP_013616281.1](file:///Volumes/ESD-USB/Summer%202018/Hassan%20Submission%20PLosOne/content.html#lcl|NC_015164.1_prot_WP_013616281.1_204) |  | + |  |  |  |  |  | [3.A.2.1.2](http://tcdb.org/search/result.php?tc=3.A.2.1.2) | 0 | P29707 | Cations | Na^+^ |
| WP_062695034.1 |  |  |  | + |  |  |  | 3.A.2.1.2 | -49 | P29710 | Cations | Na^+^ |
| [WP_029328561.1](file:///Volumes/ESD-USB/Summer%202018/Hassan%20Submission%20PLosOne/content.html#lcl|NZ_CP012801.1_prot_WP_029328561.1_76) |  |  | + |  |  |  |  | [3.A.2.1.2](http://tcdb.org/search/result.php?tc=3.A.2.1.2) | -50 | P29710 | Cations | Na^+^ |
| YP_099460.1 |  |  |  |  | + |  |  | 3.A.2.1.2 | -54 | P29710 | Cations | Na^+^ |
| [WP_004295762.1](file:///Volumes/ESD-USB/Summer%202018/Hassan%20Submission%20PLosOne/content.html#lcl|NZ_CP012938.1_prot_WP_004295762.1_890) |  |  |  |  |  | + |  | [3.A.2.1.2](http://tcdb.org/search/result.php?tc=3.A.2.1.2) | -49 | P29710 | Anions | Anions |
| [WP_005846820.1](file:///Volumes/ESD-USB/Summer%202018/Library/Application%20Support/Microsoft/Office/Office%202011%20AutoRecovery/content.html#lcl|NC_009614.1_prot_WP_005846820.1_2939) |  |  |  |  |  |  | + | [3.A.2.1.2](http://tcdb.org/search/result.php?tc=3.A.2.1.2) | 0 | P29707 | Unknown | Unknown |
| [WP_005846837.1](file:///Volumes/ESD-USB/Summer%202018/Library/Application%20Support/Microsoft/Office/Office%202011%20AutoRecovery/content.html#lcl|NC_009614.1_prot_WP_005846837.1_2947) |  |  |  |  |  |  | + | [3.A.2.1.2](http://tcdb.org/search/result.php?tc=3.A.2.1.2) | -54 | P29710 | Unknown | Unknown |
| ABI39_14990 | **+** |  |  |  |  |  |  | 3.A.2.1.3 | -12 | P07251 | Cations | Na^+^ |
| [WP_013616288.1](file:///Volumes/ESD-USB/Summer%202018/Hassan%20Submission%20PLosOne/content.html#lcl|NC_015164.1_prot_WP_013616288.1_211) |  | + |  |  |  |  |  | [3.A.2.1.3](http://tcdb.org/search/result.php?tc=3.A.2.1.3) | 0 | P07251 | Cations | H^+^ |
| [WP_007218361.1](file:///Volumes/ESD-USB/Summer%202018/Hassan%20Submission%20PLosOne/content.html#lcl|NZ_CP012801.1_prot_WP_007218361.1_77) |  |  | + |  |  |  |  | [3.A.2.1.3](http://tcdb.org/search/result.php?tc=3.A.2.1.3) | 0 | P07251 | Cations | H^+^ |
| [WP_029427943.1](file:///Volumes/ESD-USB/Summer%202018/Hassan%20Submission%20PLosOne/content.html#lcl|NZ_CP012801.1_prot_WP_029427943.1_78) |  |  | + |  |  |  |  | [3.A.2.1.3](http://tcdb.org/search/result.php?tc=3.A.2.1.3) | -11 | P09457 | Unknown | Unknown |
| WP_008761392.1 |  |  |  | + |  |  |  | 3.A.2.1.3 | -11 | P09457 | Cations | H^+^ |
| YP_099459.1 |  |  |  |  | + |  |  | 3.A.2.1.3 | 0 | P07251 | Cations | H^+^ |
| [WP_005846835.1](file:///Volumes/ESD-USB/Summer%202018/Library/Application%20Support/Microsoft/Office/Office%202011%20AutoRecovery/content.html#lcl|NC_009614.1_prot_WP_005846835.1_2946) |  |  |  |  |  |  | + | [3.A.2.1.3](http://tcdb.org/search/result.php?tc=3.A.2.1.3) | 0 | P07251 | Unknown | Unknown |
| WP_008761393.1 |  |  |  | + |  |  |  | 3.A.2.1.4 | 0 | Q24HY8 | Unknown | Unknown |
| ABI39_14975 | + |  |  |  |  |  |  | 3.A.2.1.6 | -18 | F8L1Z6 | Unknown | Unknown |
| ABI39_14960 | + |  |  |  |  |  |  | 3.A.2.1.6 | -07 | F8L1Z0 | Unknown | Unknown |
| [WP_013616285.1](file:///Volumes/ESD-USB/Summer%202018/Hassan%20Submission%20PLosOne/content.html#lcl|NC_015164.1_prot_WP_013616285.1_208) |  | + |  |  |  |  |  | [3.A.2.1.6](http://tcdb.org/search/result.php?tc=3.A.2.1.6) | -13 | F8L1Z6 | Unknown | Unknown |
| [WP_013616282.1](file:///Volumes/ESD-USB/Summer%202018/Hassan%20Submission%20PLosOne/content.html#lcl|NC_015164.1_prot_WP_013616282.1_205) |  | + |  |  |  |  |  | [3.A.2.1.6](http://tcdb.org/search/result.php?tc=3.A.2.1.6) | -13 | F8L1Z0 | Unknown | Unknown |
| ABI39_14970 | + |  |  |  |  |  |  | 3.A.2.1.7 | -47 | A1B619 | Unknown | Unknown |
| [WP_041584153.1](file:///Volumes/ESD-USB/Summer%202018/Hassan%20Submission%20PLosOne/content.html#lcl|NC_015164.1_prot_WP_041584153.1_207) |  | + |  |  |  |  |  | [3.A.2.1.7](http://tcdb.org/search/result.php?tc=3.A.2.1.7) | -16 | A1B619 | Unknown | Unknown |
| WP_008761388.1 |  |  |  | + |  |  |  | 3.A.2.1.7 | 0 | A1B8P0 | Unknown | Unknown |
| [WP_007213983.1](file:///Volumes/ESD-USB/Summer%202018/Hassan%20Submission%20PLosOne/content.html#lcl|NZ_CP012801.1_prot_WP_007213983.1_83) |  |  | + |  |  |  |  | [3.A.2.1.6](http://tcdb.org/search/result.php?tc=3.A.2.1.6) | -16 | F8L1Z0 | Cations | Na^+^/H^+^ |
| [WP_007213986.1](file:///Volumes/ESD-USB/Summer%202018/Hassan%20Submission%20PLosOne/content.html#lcl|NZ_CP012801.1_prot_WP_007213986.1_80) |  |  | + |  |  |  |  | [3.A.2.1.6](http://tcdb.org/search/result.php?tc=3.A.2.1.6) | -15 | F8L1Z6 | Cations | Na^+^/H^+^ |
| WP_004295758.1 |  |  |  | + |  |  |  | 3.A.2.1.6 | -15 | F8L1Z6 | Unknown | Unknown |
| WP_011107410.1 |  |  |  | + |  |  |  | 3.A.2.1.6 | -13 | F8L1Z0 | Cations | H^+^/Na^+^ |
| YP_099456.1 |  |  |  |  | + |  |  | 3.A.2.1.6 | -15 | F8L1Z6 | Unknown | Unknown |
| YP_099453.1 |  |  |  |  | + |  |  | 3.A.2.1.6 | -13 | F8L1Z0 | Unknown | Unknown |
| [WP_005846828.1](file:///Volumes/ESD-USB/Summer%202018/Library/Application%20Support/Microsoft/Office/Office%202011%20AutoRecovery/content.html#lcl|NC_009614.1_prot_WP_005846828.1_2943) |  |  |  |  |  |  | + | [3.A.2.1.6](http://tcdb.org/search/result.php?tc=3.A.2.1.6) | -12 | F8L1Z6 | Unknown | Unknown |
| [WP_005846822.1](file:///Volumes/ESD-USB/Summer%202018/Library/Application%20Support/Microsoft/Office/Office%202011%20AutoRecovery/content.html#lcl|NC_009614.1_prot_WP_005846822.1_2940) |  |  |  |  |  |  | + | [3.A.2.1.6](http://tcdb.org/search/result.php?tc=3.A.2.1.6) | -12 | F8L1Z0 | Unknown | Unknown |
| [WP_007213982.1](file:///Volumes/ESD-USB/Summer%202018/Hassan%20Submission%20PLosOne/content.html#lcl|NZ_CP012801.1_prot_WP_007213982.1_84) |  |  | + |  |  |  |  | [3.A.2.1.7](http://tcdb.org/search/result.php?tc=3.A.2.1.7) | 0 | A1B8P0 | Unknown | Unknown |
| [WP_007213985.1](file:///Volumes/ESD-USB/Summer%202018/Hassan%20Submission%20PLosOne/content.html#lcl|NZ_CP012801.1_prot_WP_007213985.1_81) |  |  | + |  |  |  |  | [3.A.2.1.7](http://tcdb.org/search/result.php?tc=3.A.2.1.7) | -14 | A1B619 | Unknown | Unknown |
| [WP_004295754.1](file:///Volumes/ESD-USB/Summer%202018/Hassan%20Submission%20PLosOne/content.html#lcl|NZ_CP012938.1_prot_WP_004295754.1_898) |  |  |  |  |  | + |  | [3.A.2.1.7](http://tcdb.org/search/result.php?tc=3.A.2.1.7) | 0 | A1B8P0 | Unknown | Unknown |
| [WP_012055505.1](file:///Volumes/ESD-USB/Summer%202018/Library/Application%20Support/Microsoft/Office/Office%202011%20AutoRecovery/content.html#lcl|NC_009614.1_prot_WP_012055505.1_2942) |  |  |  |  |  |  | + | [3.A.2.1.7](http://tcdb.org/search/result.php?tc=3.A.2.1.7) | -18 | A1B619 | Unknown | Unknown |
| ABI39_14985 | + |  |  |  |  |  |  | 3.A.2.1.8 | -16 | Q9RMB3 | Unknown | Unknown |
| ABI39_17860 | + |  |  |  |  |  |  | 3.A.2.2.2 | 0 | P43439 | Unknown | Unknown |
| ABI39_17855 | **+** |  |  |  |  |  |  | 3.A.2.2.2 | -23 | P43457 | Unknown | Unknown |
| [WP_029428227.1](file:///Volumes/ESD-USB/Summer%202018/Hassan%20Submission%20PLosOne/content.html#lcl|NZ_CP012801.1_prot_WP_029428227.1_4967) |  |  | + |  |  |  |  | [3.A.2.2.2](http://tcdb.org/search/result.php?tc=3.A.2.2.2) | -47 | P43439 | Cations | Na^+^ |
| [WP_007213461.1](file:///Volumes/ESD-USB/Summer%202018/Hassan%20Submission%20PLosOne/content.html#lcl|NZ_CP012801.1_prot_WP_007213461.1_4968) |  |  | + |  |  |  |  | [3.A.2.2.2](http://tcdb.org/search/result.php?tc=3.A.2.2.2) | -19 | P43457 | Cations | Na^+^ |
| YP_100013.1 |  |  |  |  | + |  |  | 3.A.2.2.2 | -45 | P43439 | Cations | Na^+^ |
| YP_100012.1 |  |  |  |  | + |  |  | 3.A.2.2.2 | -18 | P43457 | Cations | Na^+^ |
| [WP_004297644.1](file:///Volumes/ESD-USB/Summer%202018/Hassan%20Submission%20PLosOne/content.html#lcl|NZ_CP012938.1_prot_WP_004297644.1_1256) |  |  |  |  |  | + |  | [3.A.2.2.2](http://tcdb.org/search/result.php?tc=3.A.2.2.2) | -18 | P43457 | Cations | Na^+^ |
| ABI39_17875 | **+** |  |  |  |  |  |  | 3.A.2.3.3 | 0 | O83441 | Unknown | Unknown |
| ABI39_17865 | **+** |  |  |  |  |  |  | 3.A.2.3.3 | 0 | O83443 | Unknown | Unknown |
| [WP_007213465.1](file:///Volumes/ESD-USB/Summer%202018/Hassan%20Submission%20PLosOne/content.html#lcl|NZ_CP012801.1_prot_WP_007213465.1_4964) |  |  | + |  |  |  |  | [3.A.2.3.3](http://tcdb.org/search/result.php?tc=3.A.2.3.3) | 0 | O83441 | Unknown | Unknown |
| [WP_029428228.1](file:///Volumes/ESD-USB/Summer%202018/Hassan%20Submission%20PLosOne/content.html#lcl|NZ_CP012801.1_prot_WP_029428228.1_4965) |  |  | + |  |  |  |  | [3.A.2.3.3](http://tcdb.org/search/result.php?tc=3.A.2.3.3) | -139 | O83442 | Unknown | Unknown |
| [WP_007213463.1](file:///Volumes/ESD-USB/Summer%202018/Hassan%20Submission%20PLosOne/content.html#lcl|NZ_CP012801.1_prot_WP_007213463.1_4966) |  |  | + |  |  |  |  | [3.A.2.3.3](http://tcdb.org/search/result.php?tc=3.A.2.3.3) | -22 | O83443 | Unknown | Unknown |
| WP_008764971.1 |  |  |  | + |  |  |  | 3.A.2.3.3 | 0 | O83441 | Unknown | Unknown |
| WP_008762991.1 |  |  |  | + |  |  |  | 3.A.2.3.3 | -139 | O83442 | Unknown | Unknown |
| WP_008762992.1 |  |  |  | + |  |  |  | 3.A.2.3.3 | -25 | O83443 | Unknown | Unknown |
| [WP_004297638.1](file:///Volumes/ESD-USB/Summer%202018/Hassan%20Submission%20PLosOne/content.html#lcl|NZ_CP012938.1_prot_WP_004297638.1_1260) |  |  |  |  |  | + |  | [3.A.2.3.3](http://tcdb.org/search/result.php?tc=3.A.2.3.3) | 0 | O83441 | Unknown | Unknown |
| [WP_004297639.1](file:///Volumes/ESD-USB/Summer%202018/Hassan%20Submission%20PLosOne/content.html#lcl|NZ_CP012938.1_prot_WP_004297639.1_1259) |  |  |  |  |  | + |  | [3.A.2.3.3](http://tcdb.org/search/result.php?tc=3.A.2.3.3) | -140 | O83442 | Unknown | Unknown |
| [WP_004297640.1](file:///Volumes/ESD-USB/Summer%202018/Hassan%20Submission%20PLosOne/content.html#lcl|NZ_CP012938.1_prot_WP_004297640.1_1258) |  |  |  |  |  | + |  | [3.A.2.3.3](http://tcdb.org/search/result.php?tc=3.A.2.3.3) | -25 | O83443 | Unknown | Unknown |
| [WP_005846833.1](file:///Volumes/ESD-USB/Summer%202018/Library/Application%20Support/Microsoft/Office/Office%202011%20AutoRecovery/content.html#lcl|NC_009614.1_prot_WP_005846833.1_2945) |  |  |  |  |  |  | + | [3.A.2.1.8](http://tcdb.org/search/result.php?tc=3.A.2.1.8) | -07 | Q9RMB3 | Unknown | Unknown |
| [WP_005839531.1](file:///Volumes/ESD-USB/Summer%202018/Library/Application%20Support/Microsoft/Office/Office%202011%20AutoRecovery/content.html#lcl|NC_009614.1_prot_WP_005839531.1_3485) |  |  |  |  |  |  | + | [3.A.2.2.2](http://tcdb.org/search/result.php?tc=3.A.2.2.2) | -16 | P43457 | Unknown | Unknown |
| [WP_012055763.1](file:///Volumes/ESD-USB/Summer%202018/Library/Application%20Support/Microsoft/Office/Office%202011%20AutoRecovery/content.html#lcl|NC_009614.1_prot_WP_012055763.1_3489) |  |  |  |  |  |  | + | [3.A.2.3.3](http://tcdb.org/search/result.php?tc=3.A.2.3.3) | 0 | O83441 | Unknown | Unknown |
| [WP_005839525.1](file:///Volumes/ESD-USB/Summer%202018/Library/Application%20Support/Microsoft/Office/Office%202011%20AutoRecovery/content.html#lcl|NC_009614.1_prot_WP_005839525.1_3488) |  |  |  |  |  |  | + | [3.A.2.3.3](http://tcdb.org/search/result.php?tc=3.A.2.3.3) | -136 | O83442 | Unknown | Unknown |
| [WP_004295758.1](file:///Volumes/ESD-USB/Summer%202018/Hassan%20Submission%20PLosOne/content.html#lcl|NZ_CP012938.1_prot_WP_004295758.1_894) |  |  |  |  |  | + |  | [3.A.2.1.6](http://tcdb.org/search/result.php?tc=3.A.2.1.6) | -15 | F8L1Z6 | Cations | H+ |
| [WP_004303467.1](file:///Volumes/ESD-USB/Summer%202018/Hassan%20Submission%20PLosOne/content.html#lcl|NZ_CP012938.1_prot_WP_004303467.1_897) |  |  |  |  |  | + |  | [3.A.2.1.6](http://tcdb.org/search/result.php?tc=3.A.2.1.6) | -12 | F8L1Z0 | Unknown | Unknown |
| [WP_029426603.1](file:///Volumes/ESD-USB/Summer%202018/Hassan%20Submission%20PLosOne/content.html#lcl|NZ_CP012801.1_prot_WP_029426603.1_3125) |  |  | + |  |  |  |  | [3.A.3.2.17](http://tcdb.org/search/result.php?tc=3.A.3.2.17) | -151 | P54678 | Cations | Ca^2+^ |
| [WP_013618113.1](file:///Volumes/ESD-USB/Summer%202018/Hassan%20Submission%20PLosOne/content.html#lcl|NC_015164.1_prot_WP_013618113.1_2090) |  | + |  |  |  |  |  | [3.A.3.2.39](http://tcdb.org/search/result.php?tc=3.A.3.2.39) | -157 | Q6RXX1 | Cations | Ca^2+^ |
| [WP_029427623.1](file:///Volumes/ESD-USB/Summer%202018/Hassan%20Submission%20PLosOne/content.html#lcl|NZ_CP012801.1_prot_WP_029427623.1_4554) |  |  | + |  |  |  |  | [3.A.3.4.1](http://tcdb.org/search/result.php?tc=3.A.3.4.1) | 0 | P36640 | Cations | Mg^2+/^Ni^2+^ |
| YP_097494.1 |  |  |  |  | + |  |  | 3.A.3.4.1 | 0 | P36640 | Cations | Mg^2+/^Ni^2+^ |
| [WP_013618580.1](file:///Volumes/ESD-USB/Summer%202018/Hassan%20Submission%20PLosOne/content.html#lcl|NC_015164.1_prot_WP_013618580.1_2625) |  | + |  |  |  |  |  | [3.A.3.5.1](http://tcdb.org/search/result.php?tc=3.A.3.5.1) | 0 | P32113 | Cations | Cu^2+^ |
| [WP_005846887.1](file:///Volumes/ESD-USB/Summer%202018/Library/Application%20Support/Microsoft/Office/Office%202011%20AutoRecovery/content.html#lcl|NC_009614.1_prot_WP_005846887.1_2968) |  |  |  |  |  |  | + | [3.A.3.5.1](http://tcdb.org/search/result.php?tc=3.A.3.5.1) | 0 | P32113 | Cations | Cu^2+^ |
| [WP_005846887.1](file:///Volumes/ESD-USB/Summer%202018/Library/Application%20Support/Microsoft/Office/Office%202011%20AutoRecovery/content.html#lcl|NC_009614.1_prot_WP_005846887.1_2968) |  |  |  |  |  |  | + | [3.A.3.5.1](http://tcdb.org/search/result.php?tc=3.A.3.5.1) | 0 | P32113 | Cations | Cu^2+^ |
| [WP_029328329.1](file:///Volumes/ESD-USB/Summer%202018/Hassan%20Submission%20PLosOne/content.html#lcl|NZ_CP012801.1_prot_WP_029328329.1_546) |  |  | + |  |  |  |  | [3.A.3.5.15](http://tcdb.org/search/result.php?tc=3.A.3.5.15) | 0 | Q3MNJ6 | Cations | Cu^2+^ |
| WP_008765859.1 |  |  |  | + |  |  |  | 3.A.3.5.15 | 0 | Q3MNJ6 | Cations | Cu^2+^ |
| ABI39_12380 | **+** |  |  |  |  |  |  | 3.A.3.6.5 | -138 | Q8L158 | Cations | Cu^2+^ |
| WP_062695744.1 |  |  |  | + |  |  |  | 3.A.3.6.5 | 0 | Q8L158 | Cations | Cu^+^, Ag^+^, Zn^2+^, Cd^2+^ |
| YP_098580.1 |  |  |  |  | + |  |  | 3.A.3.6.5 | 0 | Q8L158 | Metals | Cu^2+^/Ag^2+/^Zn^2+^ |
| [WP_029427613.1](file:///Volumes/ESD-USB/Summer%202018/Hassan%20Submission%20PLosOne/content.html#lcl|NZ_CP012801.1_prot_WP_029427613.1_4565) |  |  | + |  |  |  |  | [3.A.3.6.10](http://tcdb.org/search/result.php?tc=3.A.3.6.10) | 0 | O32219 | Unknown | Unknown |
| ABI39_17040 | **+** |  |  |  |  |  |  | 3.A.3.7.2 | -50 | Q1M606 | Cations | Cations |
| ABI39_17045 | **+** |  |  |  |  |  |  | 3.A.3.7.2 | -32 | Q1M605 | Cations | K^+^ |
| ABI39_17035 | **+** |  |  |  |  |  |  | 3.A.3.7.2 | -47 | Q1M607 | Cations | K^+^ |
| [WP_022208853.1](file:///Volumes/ESD-USB/Summer%202018/Hassan%20Submission%20PLosOne/content.html#lcl|NZ_CP012801.1_prot_WP_022208853.1_1911) |  |  | + |  |  |  |  | [3.A.3.7.2](http://tcdb.org/search/result.php?tc=3.A.3.7.2) | 0 | Q1M606 | Cations | K^+^ |
| [WP_029429001.1](file:///Volumes/ESD-USB/Summer%202018/Hassan%20Submission%20PLosOne/content.html#lcl|NZ_CP012801.1_prot_WP_029429001.1_1912) |  |  | + |  |  |  |  | [3.A.3.7.2](http://tcdb.org/search/result.php?tc=3.A.3.7.2) | -138 | Q1M605 | Cations | K^+^ |
| [WP_007211343.1](file:///Volumes/ESD-USB/Summer%202018/Hassan%20Submission%20PLosOne/content.html#lcl|NZ_CP012801.1_prot_WP_007211343.1_1910) |  |  | + |  |  |  |  | [3.A.3.7.2](http://tcdb.org/search/result.php?tc=3.A.3.7.2) | -51 | Q1M607 | Unknown | Unknown |
| YP_097862.1 |  |  |  |  | + |  |  | 3.A.3.7.2 | 0 | Q1M606 | Cations | K^+^ |
| YP_097861.1 |  |  |  |  | + |  |  | 3.A.3.7.2 | -142 | Q1M605 | Cations | K^+^ |
| YP_097863.1 |  |  |  |  | + |  |  | 3.A.3.7.2 | -49 | Q1M607 | Cations | K^+^ |
| [WP_004323566.1](file:///Volumes/ESD-USB/Summer%202018/Hassan%20Submission%20PLosOne/content.html#lcl|NZ_CP012938.1_prot_WP_004323566.1_2310) |  |  |  |  |  | + |  | [3.A.3.7.2](http://tcdb.org/search/result.php?tc=3.A.3.7.2) | -53 | Q1M607 | Unknown | Unknown |
| [WP_005841244.1](file:///Volumes/ESD-USB/Summer%202018/Library/Application%20Support/Microsoft/Office/Office%202011%20AutoRecovery/content.html#lcl|NC_009614.1_prot_WP_005841244.1_3200) |  |  |  |  |  |  | + | [3.A.3.7.2](http://tcdb.org/search/result.php?tc=3.A.3.7.2) | 0 | Q1M606 | Cations | K^+^ |
| [WP_005841245.1](file:///Volumes/ESD-USB/Summer%202018/Library/Application%20Support/Microsoft/Office/Office%202011%20AutoRecovery/content.html#lcl|NC_009614.1_prot_WP_005841245.1_3201) |  |  |  |  |  |  | + | [3.A.3.7.2](http://tcdb.org/search/result.php?tc=3.A.3.7.2) | -138 | Q1M605 | Cations | K^+^ |
| [WP_005841244.1](file:///Volumes/ESD-USB/Summer%202018/Library/Application%20Support/Microsoft/Office/Office%202011%20AutoRecovery/content.html#lcl|NC_009614.1_prot_WP_005841244.1_3200) |  |  |  |  |  |  | + | [3.A.3.7.2](http://tcdb.org/search/result.php?tc=3.A.3.7.2) | 0 | Q1M606 | Cations | K^+^ |
| [WP_005841245.1](file:///Volumes/ESD-USB/Summer%202018/Library/Application%20Support/Microsoft/Office/Office%202011%20AutoRecovery/content.html#lcl|NC_009614.1_prot_WP_005841245.1_3201) |  |  |  |  |  |  | + | [3.A.3.7.2](http://tcdb.org/search/result.php?tc=3.A.3.7.2) | -138 | Q1M605 | Cations | K^+^ |
| WP_062695059.1 |  |  |  | + |  |  |  | 3.A.4.1.1 | -178 | P08690 | Anions | Arsenite |
| WP_062695059.1 |  |  |  | + |  |  |  | 3.A.4.1.1 | -178 | P08690 | Anions | Arsenite |
| ABI39_12785 | **+** |  |  |  |  |  |  | 3.A.5.1.1 | -161 | P10408 | Proteins | Proteins |
| ABI39_10855 | **+** |  |  |  |  |  |  | 3.A.5.1.1 | -150 | P0AGD7 | Unknown | Unknown |
| ABI39_17115 | **+** |  |  |  |  |  |  | 3.A.5.1.1 | -95 | P10121 | Unknown | Unknown |
| ABI39_18910 | **+** |  |  |  |  |  |  | 3.A.5.1.1 | -10 | P0ADZ7 | Unknown | Unknown |
| [WP_013616117.1](file:///Volumes/ESD-USB/Summer%202018/Hassan%20Submission%20PLosOne/content.html#lcl|NC_015164.1_prot_WP_013616117.1_40) |  | + |  |  |  |  |  | [3.A.5.1.1](http://tcdb.org/search/result.php?tc=3.A.5.1.1) | -149 | P0AGD7 | Unknown | Unknown |
| [WP_013616633.1](file:///Volumes/ESD-USB/Summer%202018/Hassan%20Submission%20PLosOne/content.html#lcl|NC_015164.1_prot_WP_013616633.1_567) |  | + |  |  |  |  |  | [3.A.5.1.1](http://tcdb.org/search/result.php?tc=3.A.5.1.1) | -96 | P10121 | Unknown | Unknown |
| [WP_013617256.1](file:///Volumes/ESD-USB/Summer%202018/Hassan%20Submission%20PLosOne/content.html#lcl|NC_015164.1_prot_WP_013617256.1_1224) |  | + |  |  |  |  |  | [3.A.5.1.1](http://tcdb.org/search/result.php?tc=3.A.5.1.1) | -10 | P0ADZ7 | Unknown | Unknown |
| [WP_029428648.1](file:///Volumes/ESD-USB/Summer%202018/Hassan%20Submission%20PLosOne/content.html#lcl|NZ_CP012801.1_prot_WP_029428648.1_4257) |  |  | + |  |  |  |  | [3.A.5.1.1](http://tcdb.org/search/result.php?tc=3.A.5.1.1) | -156 | P10408 | Peptides | Peptides |
| [WP_007213341.1](file:///Volumes/ESD-USB/Summer%202018/Hassan%20Submission%20PLosOne/content.html#lcl|NZ_CP012801.1_prot_WP_007213341.1_4576) |  |  | + |  |  |  |  | [3.A.5.1.1](http://tcdb.org/search/result.php?tc=3.A.5.1.1) | -153 | P0AGD7 | Unknown | Unknown |
| [WP_007214254.1](file:///Volumes/ESD-USB/Summer%202018/Hassan%20Submission%20PLosOne/content.html#lcl|NZ_CP012801.1_prot_WP_007214254.1_5104) |  |  | + |  |  |  |  | [3.A.5.1.1](http://tcdb.org/search/result.php?tc=3.A.5.1.1) | -92 | P10121 | Unknown | Unknown |
| [WP_007213184.1](file:///Volumes/ESD-USB/Summer%202018/Hassan%20Submission%20PLosOne/content.html#lcl|NZ_CP012801.1_prot_WP_007213184.1_4435) |  |  | + |  |  |  |  | [3.A.5.1.1](http://tcdb.org/search/result.php?tc=3.A.5.1.1) | -09 | P0ADZ7 | Unknown | Unknown |
| WP_008767918.1 |  |  |  | + |  |  |  | 3.A.5.1.1 | -153 | P0AGD7 | Unknown | Unknown |
| WP_011107516.1 |  |  |  | + |  |  |  | 3.A.5.1.1 | -92 | P10121 | Unknown | Unknown |
| WP_008764747.1 |  |  |  | + |  |  |  | 3.A.5.1.1 | -09 | P0ADZ7 | Unknown | Unknown |
| YP_098352.1 |  |  |  |  | + |  |  | 3.A.5.1.1 | -157 | P10408 | Proteins | Proteins |
| YP_100496.1 |  |  |  |  | + |  |  | 3.A.5.1.1 | -149 | P0AGD7 | Proteins | Proteins |
| YP_099709.1 |  |  |  |  | + |  |  | 3.A.5.1.1 | -93 | P10121 | Proteins | Proteins |
| YP_098484.1 |  |  |  |  | + |  |  | 3.A.5.1.1 | -08 | P0ADZ7 | Proteins | Proteins |
| [WP_004300796.1](file:///Volumes/ESD-USB/Summer%202018/Hassan%20Submission%20PLosOne/content.html#lcl|NZ_CP012938.1_prot_WP_004300796.1_1613) |  |  |  |  |  | + |  | [3.A.5.1.1](http://tcdb.org/search/result.php?tc=3.A.5.1.1) | -153 | P0AGD7 | Unknown | Unknown |
| [WP_004295981.1](file:///Volumes/ESD-USB/Summer%202018/Hassan%20Submission%20PLosOne/content.html#lcl|NZ_CP012938.1_prot_WP_004295981.1_702) |  |  |  |  |  | + |  | [3.A.5.1.1](http://tcdb.org/search/result.php?tc=3.A.5.1.1) | -91 | P10121 | Unknown | Unknown |
| [WP_004302063.1](file:///Volumes/ESD-USB/Summer%202018/Hassan%20Submission%20PLosOne/content.html#lcl|NZ_CP012938.1_prot_WP_004302063.1_4602) |  |  |  |  |  | + |  | [3.A.5.1.1](http://tcdb.org/search/result.php?tc=3.A.5.1.1) | -08 | P0ADZ7 | Unknown | Unknown |
| [WP_005839100.1](file:///Volumes/ESD-USB/Summer%202018/Library/Application%20Support/Microsoft/Office/Office%202011%20AutoRecovery/content.html#lcl|NC_009614.1_prot_WP_005839100.1_2168) |  |  |  |  |  |  | + | [3.A.5.1.1](http://tcdb.org/search/result.php?tc=3.A.5.1.1) | -150 | P0AGD7 | Unknown | Unknown |
| [WP_005852483.1](file:///Volumes/ESD-USB/Summer%202018/Library/Application%20Support/Microsoft/Office/Office%202011%20AutoRecovery/content.html#lcl|NC_009614.1_prot_WP_005852483.1_3215) |  |  |  |  |  |  | + | [3.A.5.1.1](http://tcdb.org/search/result.php?tc=3.A.5.1.1) | -95 | P10121 | Unknown | Unknown |
| [WP_005841619.1](file:///Volumes/ESD-USB/Summer%202018/Library/Application%20Support/Microsoft/Office/Office%202011%20AutoRecovery/content.html#lcl|NC_009614.1_prot_WP_005841619.1_3733) |  |  |  |  |  |  | + | [3.A.5.1.1](http://tcdb.org/search/result.php?tc=3.A.5.1.1) | -10 | P0ADZ7 | Unknown | Unknown |
| ABI39_03630 | + |  |  |  |  |  |  | 3.A.5.2.2 | -110 | P0A5Z2 | Unknown | Unknown |
| [WP_013617986.1](file:///Volumes/ESD-USB/Summer%202018/Hassan%20Submission%20PLosOne/content.html#lcl|NC_015164.1_prot_WP_013617986.1_1962) |  | + |  |  |  |  |  | [3.A.5.2.2](http://tcdb.org/search/result.php?tc=3.A.5.2.2) | -109 | P0A5Z2 | Unknown | Unknown |
| [WP_007212085.1](file:///Volumes/ESD-USB/Summer%202018/Hassan%20Submission%20PLosOne/content.html#lcl|NZ_CP012801.1_prot_WP_007212085.1_3275) |  |  | + |  |  |  |  | [3.A.5.2.2](http://tcdb.org/search/result.php?tc=3.A.5.2.2) | -113 | P0A5Z2 | Unknown | Unknown |
| WP_062694285.1 |  |  |  | + |  |  |  | 3.A.5.2.2 | -111 | P0A5Z2 | Unknown | Unknown |
| YP_101438.1 |  |  |  |  | + |  |  | 3.A.5.2.2 | -115 | P0A5Z2 | Proteins | Proteins |
| [WP_004296330.1](file:///Volumes/ESD-USB/Summer%202018/Hassan%20Submission%20PLosOne/content.html#lcl|NZ_CP012938.1_prot_WP_004296330.1_2632) |  |  |  |  |  | + |  | [3.A.5.2.2](http://tcdb.org/search/result.php?tc=3.A.5.2.2) | -111 | P0A5Z2 | Unknown | Unknown |
| [WP_005844891.1](file:///Volumes/ESD-USB/Summer%202018/Library/Application%20Support/Microsoft/Office/Office%202011%20AutoRecovery/content.html#lcl|NC_009614.1_prot_WP_005844891.1_778) |  |  |  |  |  |  | + | [3.A.5.2.2](http://tcdb.org/search/result.php?tc=3.A.5.2.2) | -110 | P0A5Z2 | unknown | unknown |
| YP_100655.1 |  |  |  |  | + |  |  | 3.A.5.8.1 | -08 | P14906 | Proteins | Proteins |
| ABI39_14695 | + |  |  |  |  |  |  | 3.A.6.1.2 | -13 | P0A1C1 | Unknown | Unknown |
| ABI39_09925 | + |  |  |  |  |  |  | 3.A.7.7.1 | -87 | Q07711 | Nucleotides | DNA |
| ABI39_08715 | + |  |  |  |  |  |  | 3.A.7.7.1 | -79 | Q07711 | Nucleotides | DNA |
| ABI39_16115 | + |  |  |  |  |  |  | 3.A.7.7.1 | -74 | Q07711 | Nucleotides | DNA |
| ABI39_04700 | + |  |  |  |  |  |  | 3.A.7.7.1 | -21 | Q07711 | Nucleotides | DNA |
| [WP_013617264.1](file:///Volumes/ESD-USB/Summer%202018/Hassan%20Submission%20PLosOne/content.html#lcl|NC_015164.1_prot_WP_013617264.1_1232) |  | + |  |  |  |  |  | [3.A.7.7.1](http://tcdb.org/search/result.php?tc=3.A.7.7.1) | -92 | Q07711 | Nucleotides | DNA |
| [WP_008672316.1](file:///Volumes/ESD-USB/Summer%202018/Hassan%20Submission%20PLosOne/content.html#lcl|NC_015164.1_prot_WP_008672316.1_2488) |  | + |  |  |  |  |  | [3.A.7.7.1](http://tcdb.org/search/result.php?tc=3.A.7.7.1) | -80 | Q07711 | Nucleotides | DNA |
| [WP_013617089.1](file:///Volumes/ESD-USB/Summer%202018/Hassan%20Submission%20PLosOne/content.html#lcl|NC_015164.1_prot_WP_013617089.1_1039) |  | + |  |  |  |  |  | [3.A.7.7.1](http://tcdb.org/search/result.php?tc=3.A.7.7.1) | -61 | Q07711 | Nucleotides | DNA |
| [WP_041584091.1](file:///Volumes/ESD-USB/Summer%202018/Hassan%20Submission%20PLosOne/content.html#lcl|NC_015164.1_prot_WP_041584091.1_3184) |  | + |  |  |  |  |  | [3.A.7.7.1](http://tcdb.org/search/result.php?tc=3.A.7.7.1) | -52 | Q07711 | Nucleotides | DNA |
| [WP_013618934.1](file:///Volumes/ESD-USB/Summer%202018/Hassan%20Submission%20PLosOne/content.html#lcl|NC_015164.1_prot_WP_013618934.1_2972) |  | + |  |  |  |  |  | [3.A.7.7.1](http://tcdb.org/search/result.php?tc=3.A.7.7.1) | -19 | Q07711 | Nucleotides | DNA |
| [WP_029429174.1](file:///Volumes/ESD-USB/Summer%202018/Hassan%20Submission%20PLosOne/content.html#lcl|NZ_CP012801.1_prot_WP_029429174.1_1483) |  |  | + |  |  |  |  | [3.A.7.7.1](http://tcdb.org/search/result.php?tc=3.A.7.7.1) | -85 | Q07711 | Nucleotides | DNA |
| [WP_029427372.1](file:///Volumes/ESD-USB/Summer%202018/Hassan%20Submission%20PLosOne/content.html#lcl|NZ_CP012801.1_prot_WP_029427372.1_3618) |  |  | + |  |  |  |  | [3.A.7.7.1](http://tcdb.org/search/result.php?tc=3.A.7.7.1) | -82 | Q07711 | Nucleotides | DNA |
| [WP_004291456.1](file:///Volumes/ESD-USB/Summer%202018/Hassan%20Submission%20PLosOne/content.html#lcl|NZ_CP012801.1_prot_WP_004291456.1_2922) |  |  | + |  |  |  |  | [3.A.7.7.1](http://tcdb.org/search/result.php?tc=3.A.7.7.1) | -78 | Q07711 | Nucleotides | DNA |
| [WP_029427001.1](file:///Volumes/ESD-USB/Summer%202018/Hassan%20Submission%20PLosOne/content.html#lcl|NZ_CP012801.1_prot_WP_029427001.1_207) |  |  | + |  |  |  |  | [3.A.7.7.1](http://tcdb.org/search/result.php?tc=3.A.7.7.1) | -76 | Q07711 | Nucleotides | DNA |
| [WP_007851815.1](file:///Volumes/ESD-USB/Summer%202018/Hassan%20Submission%20PLosOne/content.html#lcl|NZ_CP012801.1_prot_WP_007851815.1_152) |  |  | + |  |  |  |  | [3.A.7.7.1](http://tcdb.org/search/result.php?tc=3.A.7.7.1) | -74 | Q07711 | Nucleotides | DNA |
| [WP_022210546.1](file:///Volumes/ESD-USB/Summer%202018/Hassan%20Submission%20PLosOne/content.html#lcl|NZ_CP012801.1_prot_WP_022210546.1_3061) |  |  | + |  |  |  |  | [3.A.7.7.1](http://tcdb.org/search/result.php?tc=3.A.7.7.1) | -18 | Q07711 | Nucleotides | DNA |
| WP_008766576.1 |  |  |  | + |  |  |  | 3.A.7.7.1 | -84 | Q07711 | Nucleotides | DNA |
| WP_007485367.1 |  |  |  | + |  |  |  | 3.A.7.7.1 | -80 | Q07711 | Nucleotides | DNA |
| WP_005680137.1 |  |  |  | + |  |  |  | 3.A.7.7.1 | -65 | Q07711 | Nucleotides | DNA |
| WP_008765309.1 |  |  |  | + |  |  |  | 3.A.7.7.1 | -19 | Q07711 | Nucleotides | DNA |
| [WP_004299459.1](file:///Volumes/ESD-USB/Summer%202018/Hassan%20Submission%20PLosOne/content.html#lcl|NZ_CP012938.1_prot_WP_004299459.1_2042) |  |  |  |  |  | + |  | [3.A.7.7.1](http://tcdb.org/search/result.php?tc=3.A.7.7.1) | -84 | Q07711 | Nucleotides | DNA |
| [WP_004296118.1](file:///Volumes/ESD-USB/Summer%202018/Hassan%20Submission%20PLosOne/content.html#lcl|NZ_CP012938.1_prot_WP_004296118.1_2460) |  |  |  |  |  | + |  | [3.A.7.7.1](http://tcdb.org/search/result.php?tc=3.A.7.7.1) | -50 | Q07711 | Nucleotides | DNA |
| [WP_052587840.1](file:///Volumes/ESD-USB/Summer%202018/Hassan%20Submission%20PLosOne/content.html#lcl|NZ_CP012938.1_prot_WP_052587840.1_2765) |  |  |  |  |  | + |  | [3.A.7.7.1](http://tcdb.org/search/result.php?tc=3.A.7.7.1) | -19 | Q07711 | Nucleotides | DNA |
| [WP_011965390.1](file:///Volumes/ESD-USB/Summer%202018/Library/Application%20Support/Microsoft/Office/Office%202011%20AutoRecovery/content.html#lcl|NC_009614.1_prot_WP_011965390.1_1929) |  |  |  |  |  |  | + | [3.A.7.7.1](http://tcdb.org/search/result.php?tc=3.A.7.7.1) | -87 | Q07711 | Nucleotides | DNA |
| [WP_005828774.1](file:///Volumes/ESD-USB/Summer%202018/Library/Application%20Support/Microsoft/Office/Office%202011%20AutoRecovery/content.html#lcl|NC_009614.1_prot_WP_005828774.1_703) |  |  |  |  |  |  | + | [3.A.7.7.1](http://tcdb.org/search/result.php?tc=3.A.7.7.1) | -52 | Q07711 | Nucleotides | DNA |
| [WP_005636823.1](file:///Volumes/ESD-USB/Summer%202018/Library/Application%20Support/Microsoft/Office/Office%202011%20AutoRecovery/content.html#lcl|NC_009614.1_prot_WP_005636823.1_1510) |  |  |  |  |  |  | + | [3.A.7.7.1](http://tcdb.org/search/result.php?tc=3.A.7.7.1) | -49 | Q07711 | Nucleotides | DNA |
| [WP_005838811.1](file:///Volumes/ESD-USB/Summer%202018/Library/Application%20Support/Microsoft/Office/Office%202011%20AutoRecovery/content.html#lcl|NC_009614.1_prot_WP_005838811.1_1319) |  |  |  |  |  |  | + | [3.A.7.7.1](http://tcdb.org/search/result.php?tc=3.A.7.7.1) | -21 | Q07711 | Nucleotides | DNA |
| ABI39_08805 | + |  |  |  |  |  |  | 3.A.7.11.1 | -22 | Q5EPB6 | Nucleotides | DNA |
| [WP_013616556.1](file:///Volumes/ESD-USB/Summer%202018/Hassan%20Submission%20PLosOne/content.html#lcl|NC_015164.1_prot_WP_013616556.1_489) |  | + |  |  |  |  |  | [3.A.7.11.1](http://tcdb.org/search/result.php?tc=3.A.7.11.1) | -25 | Q5EPB6 | Nucleotides | DNA |
| [WP_013619311.1](file:///Volumes/ESD-USB/Summer%202018/Hassan%20Submission%20PLosOne/content.html#lcl|NC_015164.1_prot_WP_013619311.1_3361) |  | + |  |  |  |  |  | [3.A.7.11.1](http://tcdb.org/search/result.php?tc=3.A.7.11.1) | -21 | Q5EPB6 | Nucleotides | DNA |
| [WP_004291515.1](file:///Volumes/ESD-USB/Summer%202018/Hassan%20Submission%20PLosOne/content.html#lcl|NC_015164.1_prot_WP_004291515.1_2507) |  | + |  |  |  |  |  | [3.A.7.11.1](http://tcdb.org/search/result.php?tc=3.A.7.11.1) | -19 | Q5EPB6 | Nucleotides | DNA |
| [WP_013619038.1](file:///Volumes/ESD-USB/Summer%202018/Hassan%20Submission%20PLosOne/content.html#lcl|NC_015164.1_prot_WP_013619038.1_3083) |  | + |  |  |  |  |  | [3.A.7.11.1](http://tcdb.org/search/result.php?tc=3.A.7.11.1) | -18 | Q5EPB6 | Nucleotides | DNA |
| [WP_013617144.1](file:///Volumes/ESD-USB/Summer%202018/Hassan%20Submission%20PLosOne/content.html#lcl|NC_015164.1_prot_WP_013617144.1_1096) |  | + |  |  |  |  |  | [3.A.7.11.1](http://tcdb.org/search/result.php?tc=3.A.7.11.1) | -18 | Q5EPB6 | Nucleotides | DNA |
| [WP_044128866.1](file:///Volumes/ESD-USB/Summer%202018/Hassan%20Submission%20PLosOne/content.html#lcl|NZ_CP012801.1_prot_WP_044128866.1_120) |  |  | + |  |  |  |  | [3.A.7.11.1](http://tcdb.org/search/result.php?tc=3.A.7.11.1) | -22 | Q5EPB6 | Nucleotides | DNA |
| [WP_029426972.1](file:///Volumes/ESD-USB/Summer%202018/Hassan%20Submission%20PLosOne/content.html#lcl|NZ_CP012801.1_prot_WP_029426972.1_231) |  |  | + |  |  |  |  | [3.A.7.11.1](http://tcdb.org/search/result.php?tc=3.A.7.11.1) | -19 | Q5EPB6 | Nucleotides | DNA |
| WP_005944216.1 |  |  |  | + |  |  |  | 3.A.7.11.1 | -23 | Q5EPB6 | Nucleotides | DNA |
| WP_062695482.1 |  |  |  | + |  |  |  | 3.A.7.11.1 | -11 | Q5EP68 | Nucleotides | DNA |
| WP_062694956.1 |  |  |  | + |  |  |  | 3.A.7.11.1 | -10 | Q5EPB6 | Nucleotides | DNA |
| WP_005944236.1 |  |  |  | + |  |  |  | 3.A.7.11.1 | -10 | Q5EP68 | Nucleotides | DNA |
| WP_016269488.1 |  |  |  | + |  |  |  | 3.A.7.11.1 | -09 | Q5EP68 | Nucleotides | DNA |
| YP_100081.1 |  |  |  |  | + |  |  | 3.A.7.11.1 | -23 | Q5EPB6 | Nucleotides | DNA |
| YP_097406.1 |  |  |  |  | + |  |  | 3.A.7.11.1 | -19 | Q5EPB6 | Nucleotides | DNA |
| YP_098640.1 |  |  |  |  | + |  |  | 3.A.7.11.1 | -18 | Q5EPB6 | Nucleotides | DNA |
| YP_100110.1 |  |  |  |  | + |  |  | 3.A.7.11.1 | -09 | Q5EP68 | Nucleotides | DNA |
| YP_098053.1 |  |  |  |  | + |  |  | 3.A.7.11.1 | -08 | Q5EP68 | Nucleotides | DNA |
| [WP_007219059.1](file:///Volumes/ESD-USB/Summer%202018/Library/Application%20Support/Microsoft/Office/Office%202011%20AutoRecovery/content.html#lcl|NC_009614.1_prot_WP_007219059.1_1553) |  |  |  |  |  |  | + | [3.A.7.11.1](http://tcdb.org/search/result.php?tc=3.A.7.11.1) | -18 | Q5EPB6 | Nucleotides | DNA |
| [WP_011964859.1](file:///Volumes/ESD-USB/Summer%202018/Library/Application%20Support/Microsoft/Office/Office%202011%20AutoRecovery/content.html#lcl|NC_009614.1_prot_WP_011964859.1_660) |  |  |  |  |  |  | + | [3.A.7.11.1](http://tcdb.org/search/result.php?tc=3.A.7.11.1) | -17 | Q5EPB6 | Nucleotides | DNA |
| [WP_012055652.1](file:///Volumes/ESD-USB/Summer%202018/Library/Application%20Support/Microsoft/Office/Office%202011%20AutoRecovery/content.html#lcl|NC_009614.1_prot_WP_012055652.1_3301) |  |  |  |  |  |  | + | [3.A.7.11.1](http://tcdb.org/search/result.php?tc=3.A.7.11.1) | -11 | Q5EPB6 | Nucleotides | DNA |
| [WP_012055682.1](file:///Volumes/ESD-USB/Summer%202018/Library/Application%20Support/Microsoft/Office/Office%202011%20AutoRecovery/content.html#lcl|NC_009614.1_prot_WP_012055682.1_3333) |  |  |  |  |  |  | + | [3.A.7.11.1](http://tcdb.org/search/result.php?tc=3.A.7.11.1) | -10 | Q5EP68 | Nucleotides | DNA |
[truncated: 265,144 more chars]
